# Characterisation of the developing heart in a pressure overloaded model utilising RNA sequencing to direct functional analysis

**DOI:** 10.1111/joa.13112

**Published:** 2019-11-14

**Authors:** Matthew Parnall, Chrysostomos Perdios, Kar Lai Pang, Sophie Rochette, Siobhan Loughna

**Affiliations:** ^1^ School of Life Sciences Medical School University of Nottingham Nottingham UK; ^2^ Present address: RDM Cardiovascular Medicine Wellcome Centre for Human Genetics Oxford UK

**Keywords:** haemodynamics, heart development, chick embryo, RNA sequencing, differential gene expression

## Abstract

Cardiogenesis is influenced by both environmental and genetic factors, with blood flow playing a critical role in cardiac remodelling. Perturbation of any of these factors could lead to abnormal heart development and hence the formation of congenital heart defects. Although abnormal blood flow has been associated with a number of heart defects, the effects of abnormal pressure load on the developing heart gene expression profile have to date not clearly been defined. To determine the heart transcriptional response to haemodynamic alteration during development, outflow tract (OFT) banding was employed in the chick embryo at Hamburger and Hamilton stage (HH) 21. Stereological and expression studies, including the use of global expression analysis by RNA sequencing with an optimised procedure for effective globin depletion, were subsequently performed on HH29 OFT‐banded hearts and compared with sham control hearts, with further targeted expression investigations at HH35. The OFT‐banded hearts were found to have an abnormal morphology with a rounded appearance and left‐sided dilation in comparison with controls. Internal analysis showed they typically had a ventricular septal defect and reductions in the myocardial wall and trabeculae, with an increase in the lumen on the left side of the heart. There was also a significant reduction in apoptosis. The differentially expressed genes were found to be predominately involved in contraction, metabolism, apoptosis and neural development, suggesting a cardioprotective mechanism had been induced. Therefore, altered haemodynamics during development leads to left‐sided dilation and differential expression of genes that may be associated with stress and maintaining cardiac output.

## Graphical abstract

The pressure overloaded developing chick heart was found to have left-sided dilation and decreased apoptosis. Global gene expression profiling found changes in gene expression relating to metabolism, apoptosis, neural development and contractility, suggesting a cardioprotective response. This study provides insights into the effect that altered haemodynamics has on structure and function during cardiogenesis, increasing the understanding of the pressure overloaded developing heart.

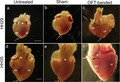

## Introduction

According to the British Heart Foundation, congenital heart defects are found in at least one in every 180 births in the UK (http://www.bhf.org.uk), with cardiomyopathies accounting for 8–11% of those diagnosed with a defect (Pedra et al. 2002). Both environmental and genetic factors affect embryonic heart formation, with blood flow playing a critical role in cardiac remodelling (Midgett & Rugonyi, 2014; Midgett et al. 2017; Courchaine et al. 2019). Animal models of haemodynamic alteration have been found to have phenotypes similar to those seen in human heart disorders, with features of cardiomyopathy (Sedmera et al. 1999; Midgett & Rugonyi, 2014; Midgett et al. 2017). The outflow tract banding (OFT‐banding) haemodynamic alteration model has been found to give a wide spectrum of cardiac malformations, such as pharyngeal arch anomalies, ventricular septal defects (VSDs), valve defects and double outlet right ventricle accompanied by increased stress–strain relations and myocardial stiffness (Sedmera et al. 1999; Tomanek et al. 1999; Miller et al. 2003; Buffinton et al. 2013; Midgett et al. 2017; Pang et al. 2017). Further, OFT‐banding in the chick leads to heart dilation (Clark et al. 1989; Tobita et al. 2002; Buffinton et al. 2013) and a reduction in compact myocardium (Tomanek et al. 1999). Myofiber alignment was also affected (Tobita et al. 2005). With regard to haemodynamic consequences of banding, chick embryos with banded hearts show an immediate and sustained increase of both peak systolic and end‐diastolic ventricular pressure (Clark et al. 1989; Tobita et al. 2002), with an increase in blood flow velocity and wall shear stress (Chivukula et al. 2016). Despite a marked increase of ventricular pressure seen in OFT‐banded embryos (Shi et al. 2013), heart rate and cardiac output/ejection fraction were not affected (Clark et al. 1989), suggesting preservation of ventricular function. Therefore, OFT‐banding is a model for a heart with a complex congenital heart defect phenotype that is prone to abnormal haemodynamics and pressure overload, and hence heart enlargement (Sanchez‐Gomez et al. 2017). Expression analysis in such hearts could pinpoint molecular pathways that may have protective or conversely debilitating long‐term effects. Hence, the elucidation of expression mechanisms could help direct potential therapeutic studies or even aid the diagnosis of a heart under stress. However, such gene pathways are currently poorly defined.

In the study described here, the OFT‐banded procedure was carried out at Hamburger–Hamilton stage (HH) 21, as the heart undergoes looping and chamber specification (Sedmera et al. 1999; Shi et al. 2013). Analysis was performed at key stages of development: at HH29 [Carnegie stage (CS) 19 for humans and embryonic day (E) 13.5 for mice], as chamber septation has just completed (Martinsen, 2005), and at HH35 (CS22 for humans and E15 for mice), when a fully septated heart with a mature apex to base conduction pattern is seen (Krishnan et al. 2014). Global gene expression analysis by RNA sequencing, together with stereological analysis, was performed on OFT‐banded chick hearts and controls at HH29. The RNA sequencing was performed with an optimised procedure described herein for globin depletion in the chick. Furthermore, targeted expression analysis on genes identified by sequencing was performed by qPCR at HH35. Findings from these studies were then used to guide further functional studies regarding apoptosis and glycogen deposition. Expression analysis highlighted genes involved in energy regulation such as *PRKAG3* (a regulatory subunit of the key metabolic regulatory enzyme AMPK) and LDHFB (involved in glucose metabolism) to be differentially regulated. Furthermore, genes such as S100A11 and PVALB (associated with improved contractility by increasing Ca^2+^ sequestering during repolarisation) were upregulated. Less active AMPK and increased *S100A11* expression can potentially lead to reduced apoptosis (Kanamori et al. 2004; Hwang et al. 2010; Law et al. 2017); a decrease in apoptosis was seen in the OFT‐banded hearts. Altered AMPK regulation has the potential to increase glycogen storage; however, no increase in glycogen storage by the periodic acid‐Schiff with diastase (PAS‐D) assay was seen in OFT‐banded hearts, suggesting a secondary mechanism. This study has identified left‐sided dilation in the OFT‐banded heart and showed gene expression that could provide a cardioprotective response to stress by attempting to maintain energy metabolism needs and contractility, together with a decrease in apoptosis.

## Methods

### Outflow tract banding, embryo isolation and histological analysis

White fertile chicken eggs (*Gallus gallus*; Dekalb white strain; Henry Stewart, UK) were incubated at 38 °C in a humidified atmosphere under constant rotation for 4 days until HH21 (Hamburger & Hamilton, 1992). After windowing, the inner shell membrane was removed to expose the heart. OFT‐banding was performed using 10‐0 nylon suture to create a snug double knot around the OFT, which was removed upon harvesting. The suture was passed through and removed immediately on sham controls. Untreated (UT) control embryos were opened and staged but no further procedures performed (Sedmera et al. 1999; Pang et al. 2017). All eggs were sealed and incubated for an additional 3–5 days until HH29‐35. Animal work was performed in accordance with national (UK Home Office) and institutional regulations and ethical guidelines. OFT‐banded and control (sham and UT) embryos were isolated and externally analysed. Hearts were then fixed in 4% paraformaldehyde, processed and wax‐embedded in a transverse orientation. Serial 8‐μm sections were taken (DSC1 microtome, Leica, Germany), dewaxed and rehydrated. For histological studies, sections were stained with Alcian blue (Sigma, UK) for 15 min at room temperature (RT) followed by Mayer’s haemalum (Raymond Lamb, UK). Images were acquired with a slide scanner (Nanozoomer 2.0‐HT, Hamamatsu, Japan).

### Stereology and morphometric measurement

Systematic random sampling (Mayhew, 1991) was used to assess tissue proportions throughout HH29 hearts (*n* = 6 sham, *n* = 7 OFT‐banded). A 96‐point grid was placed over every fifth section throughout the heart, and the tissue region and type on each point was identified (4479 points for sham, 7868 points for banded). Tissue regions consisted of right (RA) and left atrium (LA), right (RV) and left ventricle (LV). In addition, the tissue types counted included myocardial wall, extracellular matrix and lumen in the regions of RV and LV, and myocardial wall and lumen for RA and LA. Average tissue proportions of each group were calculated and tested for statistical significance.

### Apoptosis

ApopTag Peroxidase *In Situ* Apoptosis Detection Kit S7100 (Millipore, USA) was used to indicate apoptotic cells in accordance with manufacturer's instructions on 5‐μm serial sections. Imaging was performed using Zeiss Axio Scan Z1. Systematic random sampling (Mayhew, 1991) was utilised to count positive cells against total cell count on the left and right ventricular region of the heart, including the ventricular compact myocardium and trabeculae, and the base and the myocardial crest of the IVS to calculate proportions of apoptotic cells for statistical analysis. A total of 303 299 cells were counted.

### Periodic acid‐Schiff stain with diastase (PAS‐D)

Sections from HH35 hearts were prepared as described for histological studies and then subjected to diastase (Sigma), periodic acid‐Schiff (Merck) and Haemalum (Haematoxylin; Sigma) as per manufacturers’ instructions. Quantitative analysis was performed in imagej using colour deconvoluted images (Ruifork & Johnston 2001). Images were taken using an AxioPlan (Zeiss). Statistics were performed using a two‐way analysis of variance (anova).

### RNA isolation and cDNA synthesis for RNA sequencing and qPCR

RNA isolation on all hearts within direct comparison experiments were performed simultaneously by the same handler. Total RNA was extracted using TRIzol reagent (Sigma) and treated with RNase‐free DNase I (Qiagen). RNA purity was checked by NanoDrop 2000c UV/IV spectrophotometer (Thermo Fisher Scientific) at 260 nm absorbance for RNA. A260/280 ratios were 1.90–2.20 and A260/230 ratios were 2.0–2.20. R.I.N values of 9 were consistently seen following RNA purification. The values of 6.9–7.4 used for sequencing was due to sample degradation following multiple freeze thaws and globin depletion treatment. A single‐stranded template was used for standard qPCR. cDNA synthesis for RNA sequencing was double‐stranded, purified and checked for concentration via Qubit assay (Thermo Fisher Scientific). Reverse transcription reactions were performed using SuperScript® II Reverse Transcriptase (Invitrogen, UK) following the manufacturer’s instructions. All cDNA synthesis was carried out with 1 µg purified RNA in a 20‐µL reaction. cDNA from hearts within the same experiment were always synthesised in the same run, with no reverse transcriptase controls for assessment of gDNA. Hexamer primers were used at 1.25 µm. Following the reaction, samples were instantly placed on ice and then stored at −20 **°**C**.**

### Quantitative PCR

Quantitative PCR (qPCR) was run using the Applied Biosystems 7500 Fast Real‐time PCR system using SYBR Select master mix (Bio‐Rad). Gene‐specific primers were designed to give fragments of 90–210 bp and 62 °C T_m_ (± 1 °C) (Supporting Information Table S2). Primers were designed to exon/exon boundaries or to include introns > 1000 bp, except for ENS‐1/ERNI, which contained no introns. For these genes, no template controls showed minimal gDNA expression (≥ 10 Cq later than cDNA samples with Cq values > 38). Primers were optimised with cDNA diluted four times, leading to a dilution series using six points at a ratio of 1 : 5 or 1 : 3, depending on the level of gene expression. Final template dilutions for relative expression analysis were elucidated from a midway point on the linear portion of the range for each gene. All primer standard curve *R* ^2^ values were > 0.98 with efficiencies of 96–108%, with reference genes *GAPDH*, *EEF1A1* and *TBP* having efficiencies of 101–105%. A single heat‐denaturing step at 95 °C for 25 s, followed by 40 cycles of denaturation (95 °C for 20 s), annealing and extension (62 °C for 1 min) was used. Each 20‐µL qPCR reaction mixture consisted of 10 µL of iTaq™ universal SYBR® Green Supermix (1×), 0.5–0.75 µL (250–375 nm, respectively) primer combinations and optimised template concentrations. All samples were run in triplicate. Following the qPCR reaction, a melt curve was performed. Reference genes were shown to be unaffected by the OFT‐banded experimental conditions. Two reference genes were used for analysis: *GAPDH* and *EFF1A1* at HH29 and *GAPDH* and *TBP* at HH35. The threshold cycle number for product detection (ΔT value) was used to calculate the relative gene expression against reference genes using the Pfafll adjusted efficiency 2^−∆∆Ct^ method (Pfaffl, 2001). Statistics were carried out on normalized change in Cq values between OFT‐banded and sham.

### Globin depletion oligonucleotide design

Throughout all globin depletion/library preparation procedures, all OFT‐banded and sham preparations were performed simultaneously by the same handler. Globin depletion (GD) was performed via enzymatic methods on hybridised RNA/DNA complexes. Before the procedure could be carried out, specific oligonucleotides had to be designed for each globin gene. qPCR confirmed high relative expression of *HBAA*, *HBAD*, *HBB*, *HBG1*, *HBG2* and *HBZ* genes and oligonucleotides were designed using primer 3 (v. 4.1.0) and BLAST against these globin genes for depletion (Supporting Information Table S1). Oligonucleotides were designed to the 3′ end and had a T_m_ 60–70 °C.

### Globin depletion procedure

The chick‐specific designed oligonucleotides were used for GD using a modified Affymetrix protocol (Wu, 2007). A final concentration of 0.75 µm for each oligonucleotide was used for hybridisation to globin transcripts with 3 µg of total RNA. The hybridisation procedure time and temperature were optimised and a hybridisation protocol of 95 °C for 2 min, cooled down at 5 °C intervals in 50‐s steps to 50 °C, with longer 1.5‐min steps at 65 °C and 60 °C (the closest temperatures to oligonucleotide T_m_) was devised that allowed all oligos to anneal to their targets. Reaction buffer consisted of 500 mm Tris‐HCL, 1 m NaCl 5× stock with 1× used in the final hybridisation solution made up to 15 µL with DNase‐free water as necessary. Following the 50 °C step, samples were placed immediately on ice to help reduce secondary structure formation. Hybridised RNA/DNA was degraded by 4 U RNase H (0.2 U µL^−1^ in the total 20‐µL hybridisation solution) at 37 °C in RNase H buffer (NEB) containing SUPERase‐In. A 2‐U aliquot of DNase (0.07 U µL^−1^ in 30 µL) was then added to the mix after 10 min, and following a further 10 min, samples were placed on ice and the reaction was instantly stopped with ethylenediaminetetraacetic acid (EDTA) to a final concentration of 30 mm. The additional DNase step helps to degrade the DNA hybridised oligonucleotides and any genomic DNA contamination. Samples were then purified using Purelink RNA columns to allow GD total RNA to be quantified by Qubit Fluorometric assay. Statistical analysis of pre‐ and post‐GD treatment was performed by paired Student’s *t*‐test.

### Library preparation and RNA sequencing

RNA concentration was quantified using the Qubit RNA HS Assay Kit (Thermo Fisher Scientific). RNA integrity was assessed using Agilent 2100 Bioanalyzer (Agilent, USA) with an RNA integrity number (RIN) of 6.9–7.3 used across OFT‐banded and sham hearts for sequencing. Following GD, 1 µg RNA from each heart (OFT‐banded *n* = 3, sham *n* = 3) was used for RNA sequencing library preparation according to manufacturer’s instructions of the NEBNext Ultra Directional RNA Library Prep Kit for Illumina (New England Biolabs Inc.; NEB UK #E7420S/L). Twelve cycles were used for PCR enrichment. The barcoded strand‐specific libraries created were sequenced on the Illumina NextSeq 500 platform. Eight‐tenths of a full high output sequencing run was performed according to the manufacturer’s instructions. Forty million total reads came from two flow cell reads with each read having 75 bp paired‐end reads.

### RNA sequencing bioinformatic analysis

Following sequencing, compressed FASTQ files were trimmed to filter reads for adapter and low‐quality bases. sythe was used to remove adaptor sequences (https://github.com/vsbuffalo/scythe), with reads of low sequencing scores then trimmed using sickle. Reads were then aligned to the galGal4 reference genome (ICGSC Gallus_gallus‐4.0/galGal4) in the UCSC database Nov.2011 (http://genome.ucsc.edu) in the context of known gene exon coordinates by TopHat (https://ccb.jhu.edu/software/tophat/index.shtml). Alignment counts were recorded in BAM format with only uniquely mapped read alignment hits (47–90 × 106) having a quality score of MAPQ ≥ 20 used. Htseq count was used for generation of count tables. Read counts were then normalised using FPKM and TPM with resultant figures from both used for PCA using R software. As both normalisation techniques revealed nearly identical PCA plots, FPKM values were used for differential gene regulation analysis by DESeq with corrected *P*‐values of < 0.05 set as the DESeq threshold (Anders & Huber, 2010). Genes with average FPKM values < 1 were filtered from the FPKM data before gene regulation was assessed using DESeq adjusted *P*‐values (FDRs).

### Statistical analysis

All data are expressed as ± SEM, but a confidence interval of 95% was also checked. Statistical significance of the differences between OFT‐banded and sham hearts was analysed by independent *t*‐test assuming equal or unequal variances; ^*^ *P* < 0.05, ^**^ *P* < 0.01, ^***^ *P* < 0.001, ^****^ *P* < 0.0001. Assumption of normality was tested using a Shapiro–Wilk normality test. For assumption of variance, Levene’s test for homogeneity of variance was used. Tests were carried out using R, SPSS and Microsoft excel statistical analysis software.

## Results

### Alteration of haemodynamics results in enlarged hearts with ventricular septal defect

**Figure 1:**
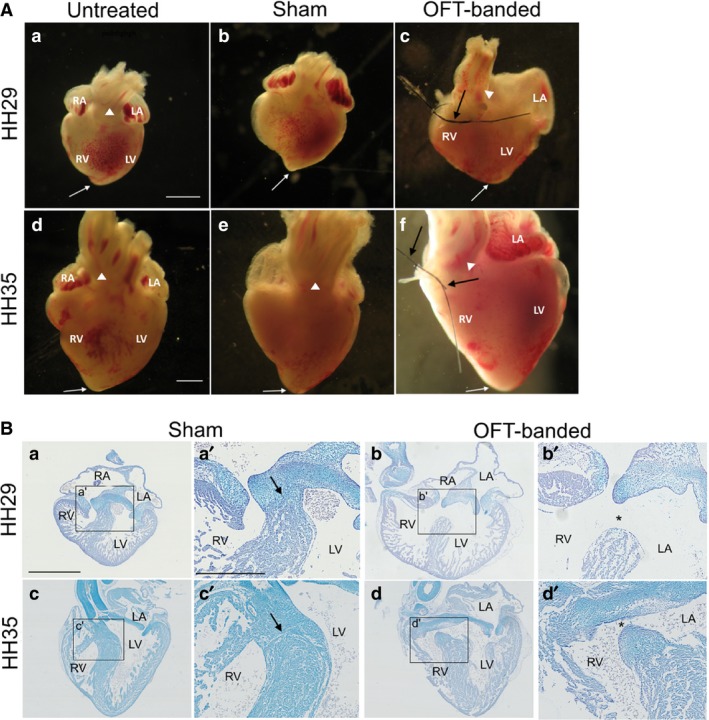
Phenotype of OFT‐banded hearts. (A) At HH29 (*n* = 97) (a,b) and HH35 (*n* = 35) (d,e), all controls displayed a normal external phenotype: the medial position of the outflow tract (denoted by arrowhead), a pointed apex (denoted by arrow) presented in both untreated (a,d) and sham (b,e). The OFT‐banded hearts (c,f) exhibited a right‐shifted position of the outflow tract (arrowhead) with a rounded ventricular apex (white arrow) and a less pronounced indentation (*n* = 33/60 at HH29, *n* = 8/9 at HH35). In addition, a thickened epicardium was seen in a subset of OFT‐banded hearts at both stages (double white arrow in c and f). The suture can be seen still attached around the OFT upon harvesting (c,f; black arrows). Scale bars: 1000 μm. (B) In controls at HH29 (*n* = 37) (a,a’) and HH35 (*n* = 8) (c,c’), the interventricular septum which grows in a superior direction has fused with the cushion in the control embryos (denoted by arrow). However, this fusion failed to occur in the OFT‐banded hearts which led to a formation of an opening (asterisk) and thus a communication between the ventricles (a ventricular septal defect) at both HH29 (*n* = 36/31; b,b’) and HH35 (*n* = 7/7; d,d’). Scale bars: 1000 μm (a,b,d), 500 μm (a’,b’‐d’). LA, left atrium; LV, left ventricle; RA, right atrium; RV, right ventricle.

Upon harvesting at HH29 and HH35, 33/60 OFT‐banded hearts appeared to display dilation and a more rounded ventricular apex at HH29 (Fig. 1Ac) and 8/9 at HH35 (Fig. 1Af) compared with controls (Fig. 1Aa,b,d,e). These features are in line with previously reported characteristics of pressure overloaded embryonic hearts (Sedmera et al. 1999; Tobita et al. 2002; Hall et al. 2004). Consistent with the literature (Pang et al. 2017), phenotypic analysis revealed a VSD at HH29 (n = 26/31) (Fig. 1Bb,b’; denoted by an asterisk in Fig. 1Bb’) and HH35 (n = 7/7) (Fig. 1Bd,d’; denoted by an asterisk in Fig. 1Bd’) compared with controls (Fig. 1Ba,a’,c,c’; denoted by an arrow in Fig. 1Ba’ and c’).

### Stereology reveals enlarged OFT‐banded hearts with changes in the chamber components representing left‐sided dilation

**Figure 2:**
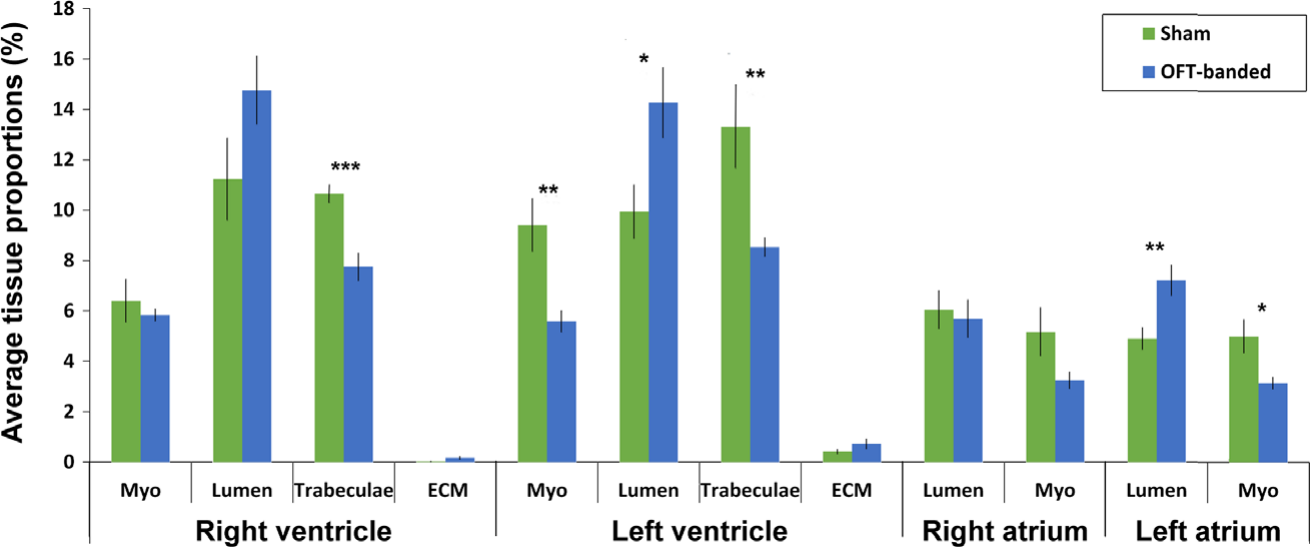
Stereological analysis of tissue types contributing to different heart regions upon OFT‐banding. Reduction of trabeculae was found in both right and left ventricle of the OFT‐banded hearts, with an increase in left ventricular lumen. In addition, the myocardium was thinner in the left ventricle but normal in the right ventricle. Also, banding led to an increase of the lumen and a decrease of myocardium in the left atrium. ECM, extracellular matrix; Myo, myocardium. Sham, *n* = 6; banded, *n* = 7. Significant differences are indicated: *P < 0.05; **P < 0.01; ***P < 0.001. Error bars indicate SEM.

To define the morphological phenotype of the OFT‐banded model, stereological analysis was performed at HH29, confirming a phenotype of left‐sided dilation (Fig. 2). Whole OFT‐banded hearts were found to be 1.77 times larger than sham controls. A significant reduction of the trabeculae was identified within both ventricular chambers of the OFT‐banded hearts. In the right ventricle (RV) a 27.3% decrease was seen (10.66 ± 0.36% sham, 7.75 ± 0.55% OFT‐banded; *P* < 0.001). Decreases were more prominent in the left ventricle (LV) with a 35.9% reduction seen (13.30 ± 1.65% sham, 8.53 ± 0.37% OFT‐banded; *P* < 0.001). Consistent with this, a simultaneous increase of LV lumen was found in OFT‐banded hearts (9.94 ± 1.06% and 14.27 ± 1.40% in sham and OFT‐banded hearts respectively, an increase of 43.6%; *P* < 0.05; Fig. 2). This coincided with a thinner myocardium in the LV by 40.7% upon banding (9.41 ± 1.05% sham, 5.58 ± 0.43% OFT‐banded; *P* < 0.01); however, no difference was seen in the RV (*P *> 0.05; Fig. 2). With regard to the atrial myocardium, a reduction of 37.07% was identified in the myocardium of the left atrium (LA) in OFT‐banded hearts (4.99 ± 0.67% sham, 3.14 ± 0.24% OFT‐banded; *P* < 0.01). However, decreases in the right atrium (RA) were found to be insignificant (5.16 ± 0.94% sham, 3.24 ± 0.34% OFT‐banded; *P *> 0.05; Fig. 2). A significant increase of the LA lumen by 47.4% was found in OFT‐banded hearts (4.9 ± 0.45% sham, 7.22 ± 0.62% OFT‐banded *P* < 0.01; Fig. 2). In contrast, no significant differences were seen in the lumen of the RA (6.04 ± 0.76% sham, 5.68 ± 0.74% OFT‐banded; *P* < 0.05).

### Globin depletion in the chick embryo

Following the identification of morphological phenotypes at HH29, the effect of OFT‐banding on gene expression was assessed by global RNA sequencing of whole HH29 hearts. To enhance discovery of possible transcripts influenced by OFT‐banding, particularly those of low reads, a library preparation procedure was adapted (Choi et al. 2014). RNase H was used to remove the polyA tail of globin transcripts prior to polyA selection. Expression of globin genes *HBAA*, *HBAD*, *HBB*, *HBG1*, *HBG2* and *HBZ* in the chick embryo was confirmed by qPCR. Then, to enable their removal, specific oligonucleotides were designed for each globin gene at their 3′ end (oligonucleotide sequences in Table S1). The RIN was 7.1–7.9 before globin depletion (GD) treatment for all samples, and 6.9–7.3 following GD.

**Figure 3:**
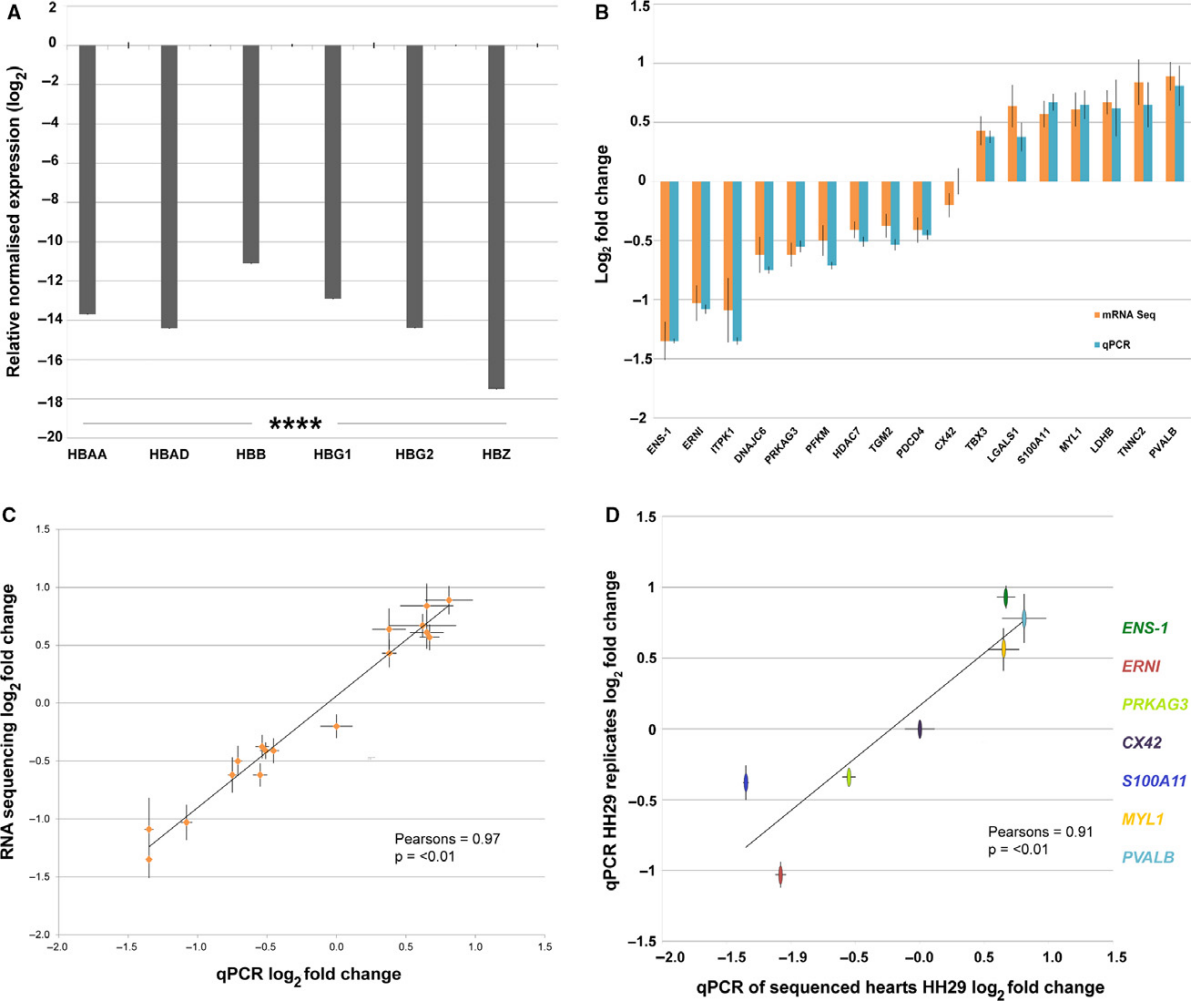
Gene expression of globin‐depleted and sequenced hearts. (A) Relative expression of each globin‐depleted gene HBAA, HBAD, HBB, HBG1, HBG2 and HBZ in pooled globin‐depleted and non‐globin‐depleted hearts. Globin depleted samples significantly different to non‐globin‐depleted. Values are ±1 SEM (*n* = 6; ****P < 0.0001). (B) Gene expression of globin‐depleted and sequenced hearts, compared with the same hearts non‐globin‐depleted by qPCR. The lack of statistical differences shows that globin depletion/sequencing preparation does not effect gene fold change (FC) values: t(32) = 0.145, *P* = 0.78 (*n* = 17). RNA sequencing non‐corrected log_2_ FC was used for direct comparison with qPCR log_2_ FC. (C) Relationship of corrected (DESeq) RNA sequencing to qPCR. Results are highly correlated and show that qPCR generally gives a small increase in log_2_ FC when compared with RNA sequencing: r(16) = 0.97, *P ≤ *0.0001) (*n* = 17). RNA sequencing DESeq corrected log_2_ FC compared with qPCR log_2_ FC. A closer parallel can be seen between non‐corrected RNA sequencing and qPCR log_2_ FC as seen in Supporting Information Fig. S5. (D) Relationship of gene expression of RNA‐sequenced hearts (*n* = 3 OFT‐banded, *n* = 3 sham) and new biological replicates (*n* = 3 OFT‐banded, *n* = 3 sham) at HH29 by qPCR. Statistically significant correlation of biologically independent samples is shown: *r*(5) = 0.91 (*P ≤ *0.01).

Globin transcripts in GD‐treated hearts showed a significant reduction of −11.10 to −17.49 log_2_ fold change (FC) by qPCR. This represents values of 0.045–0.0005% that of the original amount in the same hearts before treatment (*t* = 16.003, df = 5, *P* < 0.0001; Fig. 3A). Variation in gene expression comes from the variation between the biological replicates, as qPCR readouts show GD‐treated quantitation cycles (Cq) were consistent (see Supporting Information Table S3). This demonstrates that GD treatment removed globin genes consistently to a low point but did not remove all transcripts. It also shows that using the specific GD oligonucleotide concentration of 0.75 µm, the initial expression level of the globin gene was not a limiting factor for the number of transcripts remaining post‐GD treatment.

### Sequencing of chick globin‐depleted RNA is highly reproducible

Globin depletion has been shown to lead to reduced RNA concentrations of 33–95% to that of pre‐treatment values (Choi et al. 2014). To assess whether gene expression results were affected by the GD procedure, technical verification was required post‐RNA sequencing. This necessitated the use of qPCR to confirm that gene regulation identified by RNA sequencing in GD hearts was also seen in the same hearts without GD. A profile of 17 genes with an expression range of −1.35 to 0.81 log_2_ FC (OFT‐banded *n* = 3, sham *n* = 3) was used. No significant differences in expression were seen between GD sequenced hearts and the same hearts prior to treatment by qPCR, thus confirming sequencing expression (*t* = 0.145, df = 32, *P* = 0.78; Fig. 3B). Harvesting, RNA extraction, and library preparation were performed simultaneously across all OFT‐banded and sham samples to mitigate any batch effects. Housekeeping and qPCR reference genes *GAPDH*, *EEF1A1* and *TBP* showed consistent expression across all sham and OFT‐banded hearts by RNA sequencing at HH29. This demonstrated that library preparation was consistent across samples and confirmed the Genorm, BestKeeper and normFinder data, demonstrating stable expression in HH29 biological repeats and HH35 OFT‐banded hearts by qPCR (Fig. S3, Table S3; Supporting Information Fig. S4 also shows efficiency calibration and electrophoresis traces). The consistency of gene expression following GD was further confirmed in studies where independent untreated hearts pre‐ and post‐GD treatment (HH29 hearts, *n* = 3 per group) were used. Gene FC values remained unchanged when normalised to *GAPDH* and *EEF1A1* by qPCR (Supporting Information Figs S1 and S2).

The high consistency across gene regulation shows that the GD library preparation procedure in the chick does not lead to altered gene regulation, and also that the technical repeatability of the RNA sequencing result. The RNA sequencing and qPCR results are highly correlated (*r* = 0.97, df = 16, *P *= < 0.01; Fig. 3C) with a high level of sensitivity between small differences in fold change.

### OFT‐banded heart model shows biological repeatability of gene regulation

Biological repeatability of the OFT‐banding model was also determined at HH29. Gene regulation in RNA used for sequencing (*n* = 3 OFT‐banded, *n* = 3 sham) was compared with RNA of newly harvested hearts using qPCR (*n* = 3 OFT‐banded, *n* = 3 sham). Results showed similar gene regulation log_2_ FC results that were significantly correlated (*r* = 0.91, df = 5, *P *= < 0.01; Fig. 3D). Expression of selected genes ranged from −1.35 to 0.81 log_2_ FC. These data demonstrate the consistency of gene expression within the OFT‐banded hearts.

### Differential gene expression in OFT‐banded hearts

**Figure 4:**
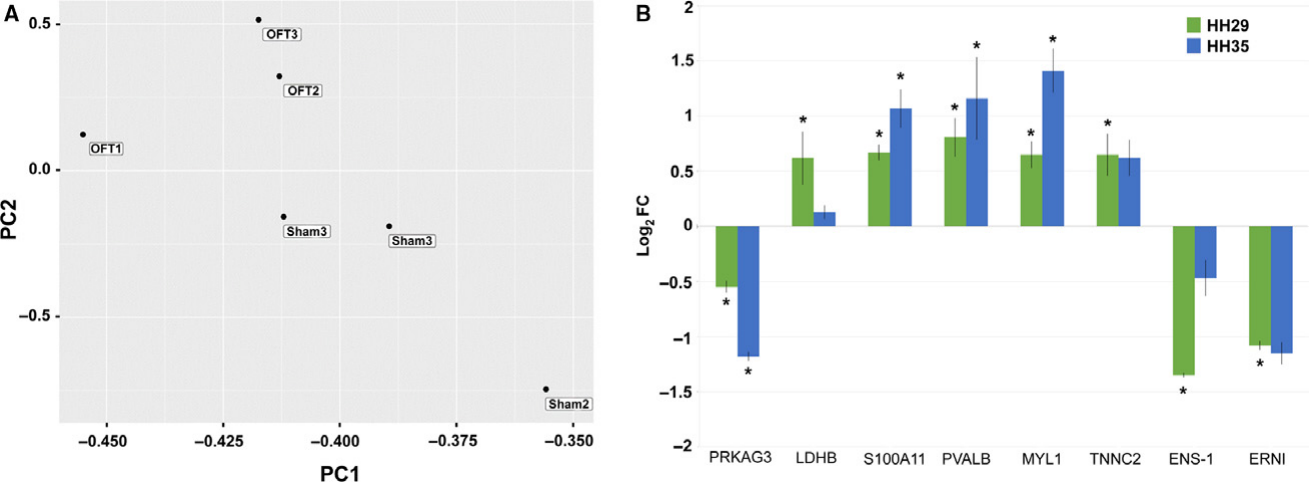
OFT‐banded hearts show overall consistency of gene expression with targeted gene analysis showing differential expression at HH29 and HH35. (A) PCA of RNA sequencing FPKM shows consistency of expression within OFT‐banded (OFT) and sham groups, with a distinction seen between groups. (B) Expression analysis of targeted genes at HH29 and HH35 by qPCR shows a general increase in differential expression in OFT‐banded hearts, with the exception of LDHB and ENS‐1, where decreases are seen (*P < 0.05; significantly differentially regulated in OFT‐banded hearts by qPCR). HH29 data are from hearts used for RNA sequencing compared with data from isolated HH35 hearts (*n* = 3 OFT‐banded, *n* = 3 sham).

Gene regulation in OFT‐banded hearts was assessed by RNA sequencing using HH29 OFT‐banded (*n* = 3) and sham (*n* = 3) hearts. In all, 40 million paired‐end reads were performed on these samples to create a global expression profile. Reads were matched to the galGal4 reference genome, identifying 6920 genes. Principal component analysis (PCA) revealed within‐group hearts were clustered together, showing consistency of expression, but that between groups the OFT‐banded and sham hearts were distinct (Fig. 4A). PCA was performed with read values normalised to total fragments (FPKM), and for comparison with total transcripts (TPM) with genes with expression levels < 1 FPKM/TPM discounted. However, no proportional differences were found in the PCA between the two normalisation methods. K‐means clustering was also performed on FPKM values on a subset of 45 genes linked to cardiac development and stress. This revealed that, without bias, all OFT‐banded hearts mapped a distinct centre from that of all sham hearts (Supporting Information Fig. S6).

Differential gene expression was analysed using DESeq with 28 genes being identified as differentially expressed in OFT‐banded hearts (using a *P*‐value cut‐off of 0.05; Table 1). To ensure differentially regulated genes of biological interest were not missed, significant genes had no minimum fold change cut‐off; however, qPCR was used to confirm differential regulation in all genes analysed further. Genes of interest highlighted as significant had corrected fold changes of 1.38–1.87 by RNA‐seq and fold changes of 1.46–2.56 by qPCR, confirming their differential regulation (Fig. 3B,C, Supporting Information Table S5), with all genes showing expression levels > 3.5 FPKM before any differential regulation.

**Table 1:** Significant differential gene expression by RNA sequencing following OFT‐banding.

Gene symbol/name	Biological function/involvement	Corrected log_2_ FC	SEM	Corrected *P*‐value
*ENS‐1/*Embryonic normal stem cell 1	Developmental regulation	−0.91	0.16	2.98E‐05
*TIMM10/*Translocase of inner mitochondrial membrane 10	Protein transport	0.72	0.13	2.98E‐05
*ERNI/*Early response to neural induction ERNI	Developmental regulation	−0.76	0.15	4.79E‐04
*GBX2/*Gastrulation brain homeobox 2	Transcription factor	0.83	0.17	8.27E‐04
*LDHB/*Lactate dehydrogenase B	Metabolic catalytic activity	0.64	0.13	1.23E‐03
*TNNC2/*Troponin C2, fast skeletal type	Regulation of muscle contraction	0.75	0.16	1.34E‐03
*PNO1/*Partner of NOB1 homolog	RNA binding	0.59	0.13	3.62E‐03
*PVALB/*Parvalbumin	Calcium ion binding/ contractility	0.7	0.17	1.91E‐02
*ADORA2B/*Adenosine A2b receptor	G protein‐coupled adenosine receptor	−0.74	0.18	2.08E‐02
*ITPK1/*Inositol‐tetrakisphosphate 1‐kinase	ATP binding kinase	−0.68	0.17	2.34E‐02
*GLRX/*Glutaredoxin	Enzyme, antioxidant defence system	−0.71	0.18	3.33E‐02
*PDCD4/*Programmed cell death 4	RNA binding inhibitor	−0.41	0.11	3.52E‐02
*PIAS2/*Protein inhibitor of activated STAT 2	Transcriptional coregulator	−0.36	0.1	4.37E‐02
*MYL10/*Myosin, light chain 10, regulatory	Calcium ion binding	0.67	0.18	4.37E‐02
*TGM2/*Transglutaminase 2	Crosslinks proteins	−0.38	0.1	4.37E‐02
*DNAJC6/*DnaJ heat shock protein family (Hsp40) member C6	Regulate molecular chaperone	−0.48	0.13	4.40E‐02
*RAP1GAP2 ‐* RAP1 GTPase‐activating protein 2	GTPase activator	−0.57	0.15	4.46E‐02
*MYL1/*Myosin, light chain 1	Calcium ion binding, structural muscle	0.54	0.15	4.46E‐02
*H1F0/*H1 histone family, member 0	Compacts histones	−0.64	0.18	4.46E‐02
*IFNGR1/*Interferon gamma receptor 1	Cytokine binding	0.55	0.15	4.46E‐02
*ARFGAP1/*ADP ribosylation factor GTPase‐activating protein 1	GTPase‐activating	−0.47	0.13	4.46E‐02
*S100A11/*S100 calcium binding protein A11	Calcium ion binding/ contractility	0.52	0.14	4.46E‐02
*LBH/*Limb bud and heart development	Modulator of transcription factors	0.6	0.17	4.86E‐02
*CDH8/*Cadherin 8	Cell adhesion	−0.6	0.16	4.86E‐02
*GSTA4/*Glutathione S‐transferase alpha 4‐like	Cellular defence against toxicity	−0.65	0.18	4.88E‐02
*LGALS1/*Galectin 1	Modulates cell‐cell/cell‐matrix interactions	0.64	0.18	4.88E‐02
*LLPH/*LLP homolog, long‐term synaptic facilitation	Transcription machinery binding	0.58	0.16	4.97E‐02

DESeq uses corrected false discovery rates (FDR) *P*‐values. Genes identified by this method have been shown to be highly verified (Anders & Huber, 2010). However, DESeq is a conservative estimator (Soneson & Delorenzi, 2013), particularly with small experiment sizes. To test whether sequencing results could be used to drive further research in genes of near significance, two genes, *PRKAG3* and *HDAC7*, with *P*‐values of 0.06 and 0.07, respectively, were further analysed by qPCR. *PRKAG3* returned a significant *P ≤ *0.05 by qPCR; however, *HDAC7* remained insignificant. The qPCR results show that the sequencing data (full gene regulation results provided in Supporting Information Table S8, and the sequencing data are accessible through GEO Series accession number http://www.ncbi.nlm.nih.gov/geo/query/acc.cgi?acc=GSE120498) can be used to target genes for further investigation and that genes just outside of significance could still potentially be targets.

### Targeted gene expression study at HH29 and HH35 highlights potential biological mechanisms

To elucidate the effect of OFT‐banding beyond HH29, observation of differential expression in genes of interest at HH29 led to their further study at HH35 by qPCR. Genes were selected based on their possible cardiac biological significance together with RNA sequencing pathway data (Table S6). Genes had potential roles in metabolism, contraction, apoptosis and neural development. PRKAG3, a gamma regulatory unit of key metabolic regulator AMPK showed a downregulation at HH29 in OFT‐banded hearts (−0.68 log_2_ FC); this differential regulation increased by 104% at HH35 (−1.18 log_2_ FC; Fig. 4B, Table S5). Sequencing also revealed that there is no compensatory response from *PRKAG3* isoforms at HH29, with *PRKAG2* expressed at very low levels of 0.1–0.4 FPKM across all samples (Supporting Information Table S7). LDHB promotes oxygen metabolism in the heart and at HH29 shows significant upregulation (0.62 log_2_ FC); however, differential regulation is eliminated at HH35 (Fig. 4B). Genes which play roles in calcium sequestering and hence are potentially important in contraction, namely S100A11, PVALB, MYL1 and TNNC2, all showed mRNA upregulation at HH29 (Fig. 4B). At HH35, intercellular membrane protein S100A11, cytoplasmic protein PVALB and structural protein MYL1 showed further increases in gene regulation of 43%, 60% and 114%, respectively; however, the structural protein TNNC2 showed a slight reduction in expression (Fig. 4). Neural developmental genes *ERNI* and *ENS‐1* show a significant downregulation of −1.08 and −1.35 log_2_ FC, respectively, at HH29. *ERNI* differential expression was maintained at HH35; however, the downregulation of *ENS‐1* was reduced to insignificant levels (Fig. 4B).

### Reduced apoptosis in HH29 OFT‐banded hearts

**Figure 5:**
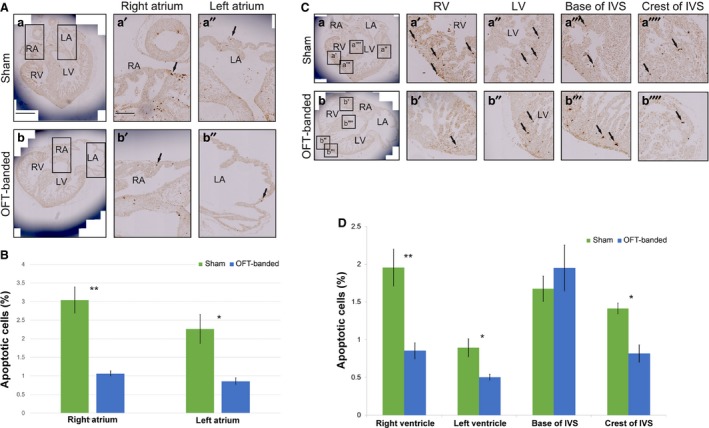
Apoptosis was decreased in both ventricles and in the myocardial crest of the interventricular septum in HH29‐banded hearts. (A,B) Apoptosis was decreased in both right and left atria upon OFT‐banding. Apoptotic cells were seen in the myocardium of both right and left atria of sham controls (a,a’,a’’) and OFT‐banded hearts (b,b’,b’’); arrows denote apoptotic cells. A decrease of apoptotic cells was revealed in both atria, with *P* < 0.01 in the right and *P* < 0.05 in left atrium. Boxed areas in (a) and (b) are shown in higher magnification in (a’,a’’) and (b,b’’), respectively. LA, left atrium; RA, right atrium. Scale bars: 300 μm (a,b), 150 μm (a’,a’’,b’,b’’). *n* = 3 for each group. (C,D) Apoptotic cells (denoted by arrows) were seen in the compact myocardium and ventricular trabeculae of both right and left ventricles in controls (a’,a’’) and OFT‐banded hearts (b’,b’’). Apoptotic cells were also seen in the base of interventricular septum and in the myocardial crest of the ventricular septum of both controls (a’’’,a’’’’) and banded (b’’’,b’’’’) hearts. A decrease of apoptotic cells was revealed in both the right (*P* < 0.01) and left ventricle (*P* < 0.05). The level of apoptosis was not found significantly different in the base of the septum but a significant reduction was revealed in the crest (*P* < 0.05). Boxed areas in (a) and (b) are shown in higher magnification in (a’‐a’’’’) and (b‐b’’’’), respectively. IVS, interventricular septum; LA, left atrium; LV, left ventricle; RA, right atrium; RV, right ventricle. *n* = 3 for each group.

Apoptosis has been shown to play a significant role in the ischaemic heart (Teringova & Tousek, 2017), and our sequencing and further qPCR analysis revealed genes that have been shown to affect apoptosis to be differentially regulated. Two such genes are the downregulated *S100A11* and upregulated *PDCD4*. Suppression of *S100A11* (Kanamori et al. 2004) and expression of *PDCD4* (Cheng et al. 2010; Liu et al. 2012) are associated with apoptosis, and the respective up‐ and downregulation of these genes could be indicative of reduced apoptosis. Therefore, apoptosis was analysed in OFT‐banded and sham control hearts. Cells positive for apoptosis were found scattered throughout the hearts in both OFT‐banded (*n* = 5) and sham (*n* = 5) groups. Significant decreases were seen in the RA, LA, RV, LV and interventricular septum (IVS) crest. In the RA, a decrease in apoptosis of 66.7% was seen (3.04 ± 0.35% sham, 1.06 ± 0.07% OFT‐banded; *P* < 0.01; Fig. 5Aa’,b’,B), and a 62.3% decrease in the LA (2.26 ± 0.39% sham, 0.85 ± 0.09% OFT‐banded; *P* < 0.05; Fig. 5Aa’’,b’’,B). Apoptosis in the RV was decreased by 56.4% (1.95 ± 0.24% sham, 0.85 ± 0.11% OFT‐banded hearts; *P* < 0.01; Fig. 5Ca’,b’,D), and in the LV by 43.8% (0.89 ± 0.12% sham, 0.5% ± 0.04% OFT‐banded hearts; *P* < 0.05; Fig. 5Ca’’,b’’,D). The crest of the IVS was decreased 42.7% (1.41 ± 0.07% sham, 0.81 ± 0.12% OFT‐banded; *P* < 0.05; Fig. 5Ca’’’’,b’’’’,D). However, no differences in apoptosis were seen at the base of the IVS (Fig. 5Ca’’’,b’’’,D).

### OFT‐banded hearts show no changes in glycogen deposition

**Figure 6:**
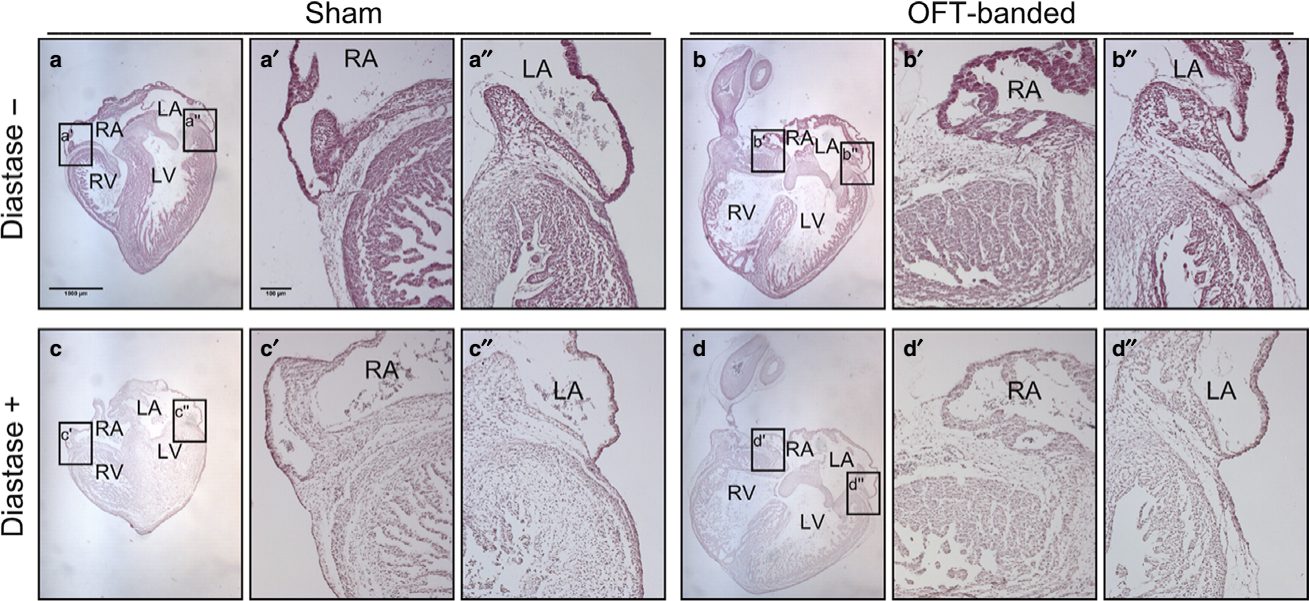
Glycogen storage is not affected in HH35‐banded hearts. Sham and OFT‐banded heart sections were treated with periodic acid‐Schiff (PAS). PAS without the addition of diastase (−) (a,b) or with a diastase reaction taking place (+) (c,d). There was no apparent difference in the treatment groups or between the right (a’,b’) and left side (a’’,b’’) of the hearts in non‐diastase‐treated and diastase‐treated right (c’,d’) and left sides (c’’,d’’). LA, left atrium; LV, left ventricle; RA, right atrium; RV, right ventricle. Scale bars: 1000 μm (a‐d), 100 μm (a’,a’’,b’,b’’,c’,c’’,d’,d’’). *n* = 3 for each group.

Expression data showing downregulation of AMPK regulator PRKAG3 could be indicative of altered glycogen storage, with glycogen storage disease and *PRKAG2* (isoform of *PRKAG3*) mutation seen in some cases of cardiomyopathy (Porto et al. 2016; Banankhah et al. 2018). To see whether any phenotype of glycogen storage was present, glycogen deposition was analysed by periodic acid‐Schiff stain with diastase (PAS‐D) at HH35 in OFT‐banded and sham hearts (*n* = 3 per group). HH35 was chosen because this stage represented the highest level of differential regulation of *PRAKG3*. Glycogen storage disease was not present; no differences were seen in glycogen deposition between OFT‐banded and control hearts (Fig. 6).

## Discussion

Environmental and genetic factors affect embryonic heart formation, with blood flow playing a critical role in cardiac remodelling (Midgett & Rugonyi, 2014; Courchaine et al. 2019). Though many haemodynamically altered heart models exist in the literature (Sedmera et al. 1999; Tomanek et al. 1999; Miller et al. 2003; Buffinton et al. 2013; Chivukula et al. 2016; Midgett et al. 2017), a global molecular characterisation of whole hearts during cardiogenesis has not been performed. Additionally, RNA sequencing was carried out on library preparations with globin transcripts removed. The globin depletion technique has been shown to increase the detection of non‐globin transcripts in expression studies, allowing greater detection of genes with low read counts (Wu, 2007; Choi et al. 2014). However, it has not been performed previously in the chick embryo. Not all species have high levels of globin mRNA, with a recent study showing that equine and bovine blood have low levels (Correia et al. 2018). However, the data presented here show six globin genes with very high expression relative to reference genes such as GAPDH, suggesting the need for globin depletion in the chick.

The OFT‐banded heart is a typical pressure overloaded embryonic heart model that gives rise to a range of structural malformations such as pharyngeal arch anomalies, persistent truncus arteriosus, ventricular septal defects and valve defects (Clark & Rosenquist, 1978; Sedmera et al. 1999; Tobita et al. 2002; Hall et al. 2004; Midgett et al. 2017; Pang et al. 2017), while also showing a left‐sided dilation. In addition, previous data from our group show changes in the ECM of the epicardium at a molecular and morphological level with a progressive aberrant phenotype from HH29 to HH35 (Perdios et al. 2019). However, overall embryonic growth was not affected upon banding, as the cardiac output and overall embryo size are the similar in control and banded embryos (Clark et al. 1989; Keller et al. 1997; Pang et al. 2017). In this study, evidence is provided of molecular mechanisms involved in OFT‐banded hearts, highlighting potential cardioprotective responses, with further analyses characterising the levels of apoptosis and glycogen accumulation.

Though some dilation of the ventricles has been reported previously (Buffinton et al. 2013), this study found dilation of both the left ventricle and atria, with an increased lumen and a decrease in myocardium and trabeculae. Though OFT‐banded models have been shown generally to give rise to a thicker myocardium (Clark et al. 1989; Sedmera et al. 1999; Tobita et al. 2002), analysis at later stages (HH36) has shown a reversal of this, with a significant reduction seen (Tomanek et al. 1999). Left atrial dilation has been postulated as a consequence of more extensive left ventricular damage, and dilated cardiomyopathy patients with dilation of the ventricle accompanied by atrial dilation are associated with a higher risk of morbidity (Modena et al. 1997). Therefore, the left‐sided dilation and reduced myocardium seen herein could be indicative of a more advanced OFT‐banded phenotype (Sedmera et al. 1999; Tobita et al. 2002). Heart dilation has been seen in humans subsequent to a previous diagnosis of a hypertrophic heart, suggesting the heart is failing (Hamada et al. 2010). Conversely, the developing or postnatal heart with a complex congenital defect (having two or more heart defects) is prone to abnormal haemodynamics, with a dilated heart seen as a secondary effect in some cases (Sanchez‐Gomez et al. 2017; Courchaine et al. 2018). In addition, a recent study has indicated that as heart development progresses (HH22 to HH36), the proximal part of the OFT (conus) becomes part of the right myocardial wall (Lazzarini et al. 2018); this process could rescue the right ventricular wall from a dilating phenotype. Therefore, to assess the molecular mechanisms involved with this phenotype during cardiogenesis, we performed global RNA sequencing on OFT‐banded and control hearts.

Sequencing found differential regulation of genes predicted to be involved in heart metabolism, apoptosis, neural development and contractility (see pathway analysis Table S6). qPCR confirmed that the most differentially expressed genes identified can be used as a guide for further functional studies. This differential gene expression as a consequence of the OFT‐banding procedure could be expected as a response to stress in order to maintain cardiac output.

One of the more unexpected sequencing results was that two of the three top differentially regulated genes were the neural developmental genes *ENS‐1* and *ERNI*. These genes are expressed during proliferation and are downregulated upon differentiation (Jean et al. 2013). They are known to be expressed in pluripotent cells of the epiblast and later in the prospective neural plate (Mey et al. 2012). Another gene, *Gbx2*, known to be involved in cardiac neural crest activation (Calmont et al. 2009; Simoes‐Costa & Bronner, 2015) showed an upregulation in OFT‐banded hearts. Multipotent cells derived from the neural crest have been detected in the conduction system of the heart into late development (Keyte & Hutson, 2012), and ablation of neural crest cells has led to a retardation of the apex to base conduction pattern (Gurjarpadhye et al. 2007). This would suggest that these cells have a role in cell differentiation in the heart. The downregulation of *ENS‐1*/*ERNI*, along with the upregulation of Gbx2, suggests that OFT‐banded hearts could suffer from abnormalities caused by aberrant neural crest differentiation. Further investigations to analyse neural crest differentiation would be of interest in future studies.

Expression of *PRKAG3*, a gamma regulatory unit of key metabolic regulator AMPK, was confirmed in the embryonic chick heart at HH29 and HH35. *PRKAG3* and *PRKAG1* are the dominant isoforms, with *PRKAG2* showing near‐zero levels of expression at HH29. In the normal human adult heart, *PRKAG2* is highly expressed, whereas *PRKAG3* shows very low expression (Pinter et al. 2012). Gamma subunits are co‐ordinately regulated in response to metabolic requirements (Pinter et al. 2013), and it is likely that in the embryonic chick heart, *PRKAG3* is expressed to compensate for the low levels of *PRKAG2*. Further, PRKAG2 has been associated with heart disorders such as cardiac hypertrophy, left ventricular dilation, supraventricular tachyarrhythmias, ventricular pre‐excitation and glycogen storage cardiomyopathies (Porto et al. 2016). The gamma subunit in response to AMP/ADP alters AMPK conformation, promoting phosphorylation and subsequently stopping dephosphorylation and AMPK inactivation (Zaha & Young, 2012). Thus, the reduction in *PRKAG3* upon OFT‐banding would be expected to lead to reduced sensitivity of AMPK to AMP/ADP/ATP levels. AMPK is a cell master metabolic regulator with many functions involved in protein kinase and transcriptional regulatory activity (Zaha & Young, 2012; Bairwa et al. 2016). Therefore, affecting AMPK activity could have many knock‐on effects in OFT‐banded hearts and is an area for further study.

Differential expression was also found for another metabolic gene that plays a key role in glucose metabolism, lactate dehydrogenase B (*LDHB*). *LDHB* is the predominant isoform in the adult heart (Valvona et al. 2016), and our sequencing confirms this is also the case in the chick embryo. It favours the reaction of lactate to pyruvate, thus promoting the TCA cycle and oxidative metabolism (Schisler et al. 2015). Under conditions of pressure overload and anaerobic stress, the heart can alter metabolism dependence in order to maintain output in response to substrate and/or oxygen availability and load (Das et al. 1987; An & Rodrigues, 2006; Lai et al. 2014). The data described here show significant upregulation at HH29; however, differential regulation is eliminated at HH35. This would suggest that at HH29 the OFT‐banded heart is trying to promote oxidative metabolism in an effort to maintain cardiac output. However, by HH35, metabolism may be becoming increasingly anaerobic, perhaps as oxygen levels are not high enough to maintain output, and the heart switches again to try to preserve cardiac output. This study analysed hearts during a period of active remodelling during cardiogenesis; metabolism is known to be involved in heart remodelling (Doenst et al. 2013). Therefore, these results would suggest a disruption to energy metabolism in OFT‐banded hearts that could have a bearing on heart morphology.

The PAS‐D assay was performed to examine any differences in glycogen storage between the OFT‐banded and control hearts. Glycogen stains a purple‐magenta colour using PAS and the amount of glycogen can be measured semi‐quantitatively by the intensity of the stain (McManus, 1948). The addition of diastase acts as a control for the diastase negative sections, as glycogen is not the only PAS‐positive element. PAS‐D can also identify other PAS‐positive elements that can be a sign of a disorder (e.g. increase in mucins) because the glycogen is digested and washed out (McManus, 1948). Although the data described here found differential regulation in genes associated with glycogen storage, the PAS‐D assay showed that the glycogen storage of the experimental model was not significantly affected in comparison with shams, suggesting glycogen storage disease was not present.

This study has also shown significant upregulation in multiple Ca^2+^ sequestering genes, which play roles in the heart’s ability to contract. Such increases could be expected as part of the heart’s response to stress in an attempt to increase contractility. A recent study has shown that treatment with a drug expected to increase contractility (phosphatase‐inhibitor‐1) in a pressure overload model actually led to heart deformities, but non‐pressure overload hearts showed the expected improvement with treatment (Schwab et al. 2018). The differential expression of *PVALB* and *S100A11* are of particular interest*.* PVALB expression was increased upon OFT‐banding in HH29 hearts, with differential expression seen to increase at later stages. *PVALB,* expressed in fast‐conducting skeletal muscle, has been used in preclinical rat trials where its expression accelerated relaxation by the action of sequestering calcium from TNNC2 to the sarcoplasmic reticulum (Szatkowski et al. 2001; Wolfram & Donahue, 2013). Its action is independent of ATP (Wolfram & Donahue, 2013), and with our results showing disruption to genes involved in energy metabolism, a protein that is not energy dependent could be an ideal therapeutic target. S100A11 has been found to be co‐expressed with S100A1 on internal cell membranes and at Z‐discs in skeletal muscle (Arcuri et al. 2002). S100A11 has not been linked directly with pressure overload (Thuny et al. 2012) but has been found to be upregulated following β‐adrenergic stimulation in the rat heart (Inamoto et al. 2000). *S100A1*‐deficient mice have been shown to have impaired cardiac contractility in response to pressure overload (Inamoto et al. 2000). Our sequencing at HH29 reveals *S100A11* to be dominantly expressed over *S100A1,* and hence a potential cardioprotective effect seen to be increasing from HH29 to HH35. Overexpression of *S100A11* is associated with reduced apoptosis, whereas suppression has been seen to lead to apoptosis (Kanamori et al. 2004). The decrease in apoptosis seen in OFT‐banded hearts suggests the increase in *S100A11* expression may be involved.

Upregulation of PDCD4 is associated with apoptosis and is a target of microRNA‐21 and microRNA‐499‐5p, both of which have been linked to cardioprotection (Cheng et al. 2010; Liu et al. 2012; Jia et al. 2016; Li et al. 2016). Therefore, downregulation of PDCD4 expression with a concomitant decrease of apoptosis in this study suggests the embryonic hearts were undergoing an intrinsic cardioprotective mechanism in response to pressure overloading. Further, AMPK has been found to be a key regulator of cell death in response to stress, with AMPK activation coinciding with cell cycle arrest and induction of apoptosis (Hwang et al. 2010; Law et al. 2017). Apoptosis is not a characteristic of the normal human adult heart; however, increased apoptosis has been linked to a heart that is failing (Narula et al. 2000; Zhao et al. 2016). A decrease in apoptosis has been shown to provide a protective/defensive mechanism in the fetal heart (Schaffer et al. 2000; Tao et al. 2015). Therefore, these studies support the notion that the heart is undergoing a protective response, and are further evidence that the heart is not yet failing.

With a key period of chamber development being haemodynamically altered, from banding at HH21 to RNA sequencing and structural analysis at HH29, it could be that the differential gene regulation seen in metabolic genes, though attempting to respond to changes in load, had a direct bearing on the anatomical phenotype seen in OFT‐banded hearts. The pressure overloaded developing chick heart was found to have left‐sided dilation and a decrease in apoptosis. Upon analysis of the global gene expression profile using RNA sequencing on globin ‐depleted RNA, changes in gene expression related to metabolism, apoptosis, neural development and contractility, with further targeted expression studies at a later stage of development performed. These data suggest the heart is responding in a cardioprotective manner. Together, this study provides insights into the effect that altered haemodynamics has on heart structure and function, which will help increase the understanding of heart development while subjected to pressure overload. This may focus future functional and therapeutic studies to elucidate the mechanisms involved.

## Author contributions

S.L. M.P. and K.P. conceived the initial project idea. M.P. K.P. and C.P. designed experiments. M.P. C.P. K.P. and S.R. performed the experiments. M.P. analysed and interpreted data.; S.L. supervised the project. M.P. took the lead in writing the manuscript, with all authors contributing.

## Conflict of interest

The authors declare no conflict of interest.

## Supporting information


**Fig. S1.** Relative mRNA quantity comparison in RNase‐treated (+RNase, globin‐depleted) and non‐RNase treated (–RNase, non‐globin‐treated) samples. An approximately one‐fold decrease in relative quantity was seen in globin‐depleted samples. Three untreated control HH29 hearts where used, with the same hearts used in both groups.
**Fig. S2.** Normalised expression of *GJA5* in non‐experimental control hearts for RNase‐treated (globin‐depleted) and non‐RNase‐treated (non‐globin‐depleted).
**Fig. S3.** Analysis of reference gene stability by qPCR in HH35 OFT‐banded hearts by Genorm, BestKeeper and normFinder (*n *= 4 OFT‐banded, 4 sham, 4 untreated hearts).
**Fig. S4.** qPCR reference gene primer optimisation**.**
**Fig. S5.** RNA sequencing correlation in OFT‐banded and sham controls, using non‐corrected fold change (FC) values compared with qPCR, demonstrate a close relationship: r(16) = 0.98, *P* ≤ 0.0001 (*n* = 17).
**Fig. S6.** OFT‐banded hearts show unbiased mapping to distinct clusters based on a profile of 45 genes linked to cardiac development and stress (all genes are shown in Tables 1 and S7)
**Table S1.** Oligonucleotide design for globin gene hybridisation.
**Table S2.** qPCR primer sequences
**Table S3.** qPCR on globin‐depleted and non‐globin‐depleted mRNA show consistency of mean Cq
**Table S4.** RNA sequencing of housekeeping and qPCR reference genes at HH29 shows consistent expression.
**Table S5.** Comparison of targeted genes by RNA sequencing and qPCR at HH29 and HH35.
**Table S6.** Molecular pathway analysis of significant differentially regulated genes in OFT‐banded hearts.
**Table S7.** Fragment per kilobase per million (FPKM) of genes of biological interest from RNA sequenced OFT‐banded and sham control hearts
**Table S8.** RNA sequencing gene regulation (OFT‐banded vs sham) by DEseq

## Data Availability

The data discussed in this publication have been deposited in the NCBI Gene Expression Omnibus (Barrett et al. 2013) and are accessible through GEO Series accession number http://www.ncbi.nlm.nih.gov/geo/query/acc.cgi?acc=GSE120498.

## References

[joa13112-bib-0001] An D , Rodrigues B (2006) Role of changes in cardiac metabolism in development of diabetic cardiomyopathy. Am J Physiol Heart Circ Physiol 291, H1489–506. 16751293 10.1152/ajpheart.00278.2006

[joa13112-bib-0002] Anders S , Huber W (2010) Differential expression analysis for sequence count data. Genome Biol 11, R106. 20979621 10.1186/gb-2010-11-10-r106 PMC3218662

[joa13112-bib-0003] Arcuri C , Giambanco I , Bianchi R , et al (2002) Subcellular localization of S100A11 (S100C, calgizzarin) in developing and adult avian skeletal muscles. Biochim Biophys Acta 1600, 84–94. 12445463 10.1016/s1570-9639(02)00448-x

[joa13112-bib-0004] Bairwa SC , Parajuli N , Dyck JR (2016) The role of AMPK in cardiomyocyte health and survival. Biochim Biophys Acta 1862, 2199–2210. 27412473 10.1016/j.bbadis.2016.07.001

[joa13112-bib-0005] Banankhah P , Fishbein GA , Dota A , et al (2018) Cardiac manifestations of PRKAG2 mutation. BMC Med Genet 19, 1. 29298659 10.1186/s12881-017-0512-6 PMC5751825

[joa13112-bib-0006] Barrett T , Wilhite SE , Ledoux P , et al (2013) NCBI GEO: archive for functional genomics data sets–update. Nucleic Acids Res 41, D991–995. 23193258 10.1093/nar/gks1193 PMC3531084

[joa13112-bib-0007] Buffinton CM , Faas D , Sedmera D (2013) Stress and strain adaptation in load‐dependent remodeling of the embryonic left ventricle. Biomech Model Mechanobiol 12, 1037–1051. 23254562 10.1007/s10237-012-0461-0 PMC3646082

[joa13112-bib-0008] Calmont A , Ivins S , van Bueren KL , et al (2009) Tbx1 controls cardiac neural crest cell migration during arch artery development by regulating Gbx2 expression in the pharyngeal ectoderm. Development 136, 3173–3183. 19700621 10.1242/dev.028902 PMC2730371

[joa13112-bib-0009] Cheng Y , Zhu P , Yang J , et al (2010) Ischaemic preconditioning‐regulated miR‐21 protects heart against ischaemia/reperfusion injury via anti‐apoptosis through its target PDCD4. Cardiovasc Res 87, 431–439. 20219857 10.1093/cvr/cvq082 PMC2904662

[joa13112-bib-0010] Chivukula VK , Goenezen S , Liu A , et al (2016) Effect of outflow tract banding on embryonic cardiac hemodynamics. J Cardiovasc Dev Dis 3, pii: 1. 10.3390/jcdd3010001 PMC4827265 27088080

[joa13112-bib-0011] Choi I , Bao H , Kommadath A , et al(2014) Increasing gene discovery and coverage using RNA‐seq of globin RNA reduced porcine blood samples. BMC Genom 15, 954. 10.1186/1471-2164-15-954 PMC4230834 25374277

[joa13112-bib-0012] Clark EB , Rosenquist GC (1978) Spectrum of cardiovascular anomalies following cardiac loop constriction in the chick embryo. Birth Defects Orig Artic Ser 14, 431–442. 737312

[joa13112-bib-0013] Clark EB , Hu N , Frommelt P , et al (1989) Effect of increased pressure on ventricular growth in stage 21 chick embryos. Am J Physiol 257, H55–61. 2750949 10.1152/ajpheart.1989.257.1.H55

[joa13112-bib-0014] Correia CN , McLoughlin KE , Nalpas NC , et al (2018) RNA sequencing (RNA‐Seq) reveals extremely low levels of reticulocyte‐derived globin gene transcripts in peripheral blood from horses (*Equus caballus*) and cattle (*Bos taurus*). Front Genet 9, 278. 30154823 10.3389/fgene.2018.00278 PMC6102425

[joa13112-bib-0015] Courchaine K , Rykiel G , Rugonyi S (2018) Influence of blood flow on cardiac development. Prog Biophys Mol Biol 137, 95–110. 29772208 10.1016/j.pbiomolbio.2018.05.005 PMC6109420

[joa13112-bib-0016] Courchaine K , Gray MJ , Beel K , et al. (2019) 4‐D computational modeling of cardiac outflow tract hemodynamics over looping developmental stages in chicken embryos. J Cardiovasc Dev Dis 6, pii: E11. 10.3390/jcdd6010011 PMC6463052 30818869

[joa13112-bib-0017] Das DK , Engelman RM , Rousou JA , et al. (1987) Aerobic vs anaerobic metabolism during ischemia in heart muscle. Ann Chir Gynaecol 76, 68–76. 3592561

[joa13112-bib-0018] Doenst T , Nguyen TD , Abel ED (2013) Cardiac metabolism in heart failure: implications beyond ATP production. Circ Res 113, 709–724. 23989714 10.1161/CIRCRESAHA.113.300376 PMC3896379

[joa13112-bib-0112] Fabregat A , Sidiropoulos K , Viteri G , et al. (2017) Reactome pathway analysis: a high‐performance in‐memory approach. BMC Bioinformatics 18, 142. 28249561 10.1186/s12859-017-1559-2 PMC5333408

[joa13112-bib-0019] Gurjarpadhye A , Hewett KW , Justus C , et al. (2007) Cardiac neural crest ablation inhibits compaction and electrical function of conduction system bundles. Am J Physiol Heart Circ Physiol 292, H1291–300. 17172273 10.1152/ajpheart.01017.2006

[joa13112-bib-0020] Hall CE , Hurtado R , Hewett KW , et al. (2004) Hemodynamic‐dependent patterning of endothelin converting enzyme 1 expression and differentiation of impulse‐conducting Purkinje fibers in the embryonic heart. Development 131, 581–592. 14711873 10.1242/dev.00947

[joa13112-bib-0021] Hamada T , Kubo T , Kitaoka H , et al. (2010) Clinical features of the dilated phase of hypertrophic cardiomyopathy in comparison with those of dilated cardiomyopathy. Clin Cardiol 33, E24–28. 10.1002/clc.20533 PMC6652988 20641106

[joa13112-bib-0022] Hamburger V , Hamilton HL (1992) A series of normal stages in the development of the chick embryo. Developmental Dynamics 195, 231–272. 1304821 10.1002/aja.1001950404

[joa13112-bib-0023] Hwang SK , Piao L , Lim HT , et al. (2010) Suppression of lung tumorigenesis by leucine zipper/EF hand‐containing transmembrane‐1. PLoS ONE 5(9), e12535. 20824095 10.1371/journal.pone.0012535 PMC2932724

[joa13112-bib-0024] Inamoto S , Murao S , Yokoyama M , et al. (2000) Isoproterenol‐induced myocardial injury resulting in altered S100A4 and S100A11 protein expression in the rat. Pathol Int 50, 480–485. 10886724 10.1046/j.1440-1827.2000.01069.x

[joa13112-bib-0025] Jean C , Aubel P , Soleihavoup C , et al. (2013) Pluripotent genes in avian stem cells. Dev Growth Differ 55, 41–51. 23278808 10.1111/dgd.12021

[joa13112-bib-0026] Jia Z , Wang J , Shi Q , et al. (2016) SOX6 and PDCD4 enhance cardiomyocyte apoptosis through LPS‐induced miR‐499 inhibition. Apoptosis 21, 174–183. 26659076 10.1007/s10495-015-1201-6 PMC4712245

[joa13112-bib-0027] Kanamori T , Takakura K , Mandai M , et al. (2004) Increased expression of calcium‐binding protein S100 in human uterine smooth muscle tumours. Mol Hum Reprod 10, 735–742. 15322223 10.1093/molehr/gah100

[joa13112-bib-0028] Keller BB , Yoshigi M , Tinney JP (1997) Ventricular‐vascular uncoupling by acute conotruncal occlusion in the stage 21 chick embryo. Am J Physiol 273, H2861–2866. 9435625 10.1152/ajpheart.1997.273.6.H2861

[joa13112-bib-0029] Keyte A , Hutson MR (2012) The neural crest in cardiac congenital anomalies. Differentiation 84, 25–40. 22595346 10.1016/j.diff.2012.04.005 PMC3389200

[joa13112-bib-0110] Krishnan A , Samtani R , Dhanantwari P , et al. (2014) A detailed comparison of mouse and human cardiac development. Pediatric Research 76(6), 500–507. 25167202 10.1038/pr.2014.128 PMC4233008

[joa13112-bib-0030] Lai L , Leone TC , Keller MP , et al. (2014) Energy metabolic reprogramming in the hypertrophied and early stage failing heart: a multisystems approach. Circ Heart Fail 7, 1022–1031. 25236884 10.1161/CIRCHEARTFAILURE.114.001469 PMC4241130

[joa13112-bib-0031] Law BYK , Gordillo‐Martinez F , Qu YQ , et al. (2017) Thalidezine, a novel AMPK activator, eliminates apoptosis‐resistant cancer cells through energy‐mediated autophagic cell death. Oncotarget 8, 30077–30091. 28404910 10.18632/oncotarget.15616 PMC5444727

[joa13112-bib-0032] Lazzarini R , Gomez‐Quiroz LE , Gonzalez‐Marquez H , et al. (2018) The proximal segment of the embryonic outflow (conus) does not participate in aortic vestibule development. PLoS ONE 13, e0209930. 30596770 10.1371/journal.pone.0209930 PMC6312233

[joa13112-bib-0033] Li Y , Lu J , Bao X , et al. (2016). MiR‐499‐5p protects cardiomyocytes against ischaemic injury via anti‐apoptosis by targeting PDCD4. Oncotarget 7, 35607‐35617. 27231854 10.18632/oncotarget.9597 PMC5094948

[joa13112-bib-0034] Liu C , Peng Z , Zhang N , et al. (2012) Identification of differentially expressed microRNAs and their PKC‐isoform specific gene network prediction during hypoxic pre‐conditioning and focal cerebral ischemia of mice. J Neurochem 120, 830–841. 22171942 10.1111/j.1471-4159.2011.07624.x

[joa13112-bib-0035] Martinsen BJ (2005) Reference guide to the stages of chick heart embryology. Dev Dyn 233, 1217–1237. 15986452 10.1002/dvdy.20468

[joa13112-bib-0036] Mayhew TM (1991) The new stereological methods for interpreting functional morphology from slices of cells and organs. Exp Physiol 76, 639–665. 1742008 10.1113/expphysiol.1991.sp003533

[joa13112-bib-0037] McManus JF (1948) Histological and histochemical uses of periodic acid. Stain Technol 23, 99–108. 18867618 10.3109/10520294809106232

[joa13112-bib-0038] Mey A , Acloque H , Lerat E , et al. (2012) The endogenous retrovirus ENS‐1 provides active binding sites for transcription factors in embryonic stem cells that specify extra embryonic tissue. Retrovirology 9, 21. 22420414 10.1186/1742-4690-9-21 PMC3362752

[joa13112-bib-0039] Midgett M , Rugonyi S (2014) Congenital heart malformations induced by hemodynamic altering surgical interventions. Front Physiol 5, 287. 25136319 10.3389/fphys.2014.00287 PMC4117980

[joa13112-bib-0040] Midgett M , Thornburg K , Rugonyi S (2017) Blood flow patterns underlie developmental heart defects. Am J Physiol Heart Circ Physiol 312, H632–H642. 28062416 10.1152/ajpheart.00641.2016 PMC5402020

[joa13112-bib-0041] Miller CE , Wong CL , Sedmera D (2003) Pressure overload alters stress‐strain properties of the developing chick heart. Am J Physiol Heart Circ Physiol 285, H1849–1856. 12855423 10.1152/ajpheart.00384.2002

[joa13112-bib-0042] Modena MG , Muia N , Sgura FA , et al. (1997) Left atrial size is the major predictor of cardiac death and overall clinical outcome in patients with dilated cardiomyopathy: A long‐term follow‐up study. Clin Cardiol 20, 553–560. 9181267 10.1002/clc.4960200609 PMC6655314

[joa13112-bib-0043] Narula J , Kolodgie FD , Virmani R (2000) Apoptosis and cardiomyopathy. Curr Opin Cardiol 15, 183–188. 10952426 10.1097/00001573-200005000-00011

[joa13112-bib-0044] Pang KL , Parnall M , Loughna S (2017) Effect of altered haemodynamics on the developing mitral valve in chick embryonic heart. J Mol Cell Cardiol 108, 114–126. 28576718 10.1016/j.yjmcc.2017.05.012 PMC5529288

[joa13112-bib-0045] Paz MV , Cotan D , Maraver JG , et al. (2016) Erratum to: AMPK regulation of cell growth, apoptosis, autophagy, and bioenergetics. Exp Suppl 107, E1. 28197974 10.1007/978-3-319-43589-3_21

[joa13112-bib-0046] Pedra SR , Smallhorn JF , Ryan G , et al. (2002) Fetal cardiomyopathies: pathogenic mechanisms, hemodynamic findings, and clinical outcome. Circulation 106, 585–591. 12147541 10.1161/01.cir.0000023900.58293.fe

[joa13112-bib-0047] Perdios C , Parnall M , Pang KL , et al. (2019) Altered haemodynamics causes aberrations in the epicardium. J Anat 234, 800–814. 30882904 10.1111/joa.12977 PMC6539700

[joa13112-bib-0048] Pfaffl MW (2001) A new mathematical model for relative quantification in real‐time RT–PCR. Nucleic Acids Res 29, e45–e45. 11328886 10.1093/nar/29.9.e45 PMC55695

[joa13112-bib-0049] Pinter K , Grignani RT , Czibik G , et al. (2012) Embryonic expression of AMPK gamma subunits and the identification of a novel gamma2 transcript variant in adult heart. J Mol Cell Cardiol 53, 342–349. 22683324 10.1016/j.yjmcc.2012.05.017 PMC3477313

[joa13112-bib-0050] Pinter K , Grignani RT , Watkins H , et al. (2013) Localisation of AMPK gamma subunits in cardiac and skeletal muscles. J Muscle Res Cell Motil 34, 369–378. 24037260 10.1007/s10974-013-9359-4 PMC3853370

[joa13112-bib-0051] Porto AG , Brun F , Severini GM , et al. (2016) Clinical Spectrum of PRKAG2 Syndrome. Circ Arrhythm Electrophysiol 9, e003121. 26729852 10.1161/CIRCEP.115.003121 PMC4704128

[joa13112-bib-0111] Ruifrok AC , Johnston DA (2001) Quantification of histochemical staining by color deconvolution. Analytical and quantitative cytology and histology 23(4), 291–299. 11531144

[joa13112-bib-0052] Sanchez‐Gomez MC , Garcia‐Mejia KA , Perez‐Diaz Conti M , et al. (2017) MicroRNAs association in the cardiac hypertrophy secondary to complex congenital heart disease in children. Pediatr Cardiol 38, 991–1003. 28382463 10.1007/s00246-017-1607-8

[joa13112-bib-0053] Schaffer SW , Croft CB , Solodushko V (2000) Cardioprotective effect of chronic hyperglycemia: effect on hypoxia‐induced apoptosis and necrosis. Am J Physiol Heart Circ Physiol 278, H1948–1954. 10843893 10.1152/ajpheart.2000.278.6.H1948

[joa13112-bib-0054] Schisler JC , Grevengoed TJ , Pascual F , et al. (2015) Cardiac energy dependence on glucose increases metabolites related to glutathione and activates metabolic genes controlled by mechanistic target of rapamycin. J Am Heart Assoc 4, pii: e001136. 10.1161/JAHA.114.001136 PMC4345858 25713290

[joa13112-bib-0055] Schwab DM , Tilemann L , Bauer R , et al. (2018) AAV‐9 mediated phosphatase‐1 inhibitor‐1 overexpression improves cardiac contractility in unchallenged mice but is deleterious in pressure‐overload. Gene Ther 25, 13–19. 29350681 10.1038/gt.2017.97

[joa13112-bib-0056] Sedmera D , Pexieder T , Rychterova V , et al. (1999) Remodeling of chick embryonic ventricular myoarchitecture under experimentally changed loading conditions. Anat Rec 254, 238–252. 9972809 10.1002/(SICI)1097-0185(19990201)254:2<238::AID-AR10>3.0.CO;2-V

[joa13112-bib-0057] Shi L , Goenezen S , Haller S , et al. (2013) Alterations in pulse wave propagation reflect the degree of outflow tract banding in HH18 chicken embryos. Am J Physiol Heart Circ Physiol 305, H386–396. 23709601 10.1152/ajpheart.00100.2013 PMC3742871

[joa13112-bib-0058] Simoes‐Costa M , Bronner ME (2015) Establishing neural crest identity: a gene regulatory recipe. Development 142, 242–257. 25564621 10.1242/dev.105445 PMC4302844

[joa13112-bib-0059] Soneson C , Delorenzi M (2013) A comparison of methods for differential expression analysis of RNA‐seq data. BMC Bioinformatics 14, 91. 23497356 10.1186/1471-2105-14-91 PMC3608160

[joa13112-bib-0060] Szatkowski ML , Westfall MV , Gomez CA , et al. (2001) In vivo acceleration of heart relaxation performance by parvalbumin gene delivery. J Clin Invest 107, 191–198. 11160135 10.1172/JCI9862 PMC198873

[joa13112-bib-0061] Tao L , Bei Y , Zhang H , et al. (2015) Exercise for the heart: signaling pathways. Oncotarget 6, 20773–20784. 26318584 10.18632/oncotarget.4770 PMC4673228

[joa13112-bib-0062] Teringova E , Tousek P (2017) Apoptosis in ischemic heart disease. J Transl Med 15, 87. 28460644 10.1186/s12967-017-1191-y PMC5412049

[joa13112-bib-0063] Thuny F , Textoris J , Amara AB , et al. (2012) The gene expression analysis of blood reveals S100A11 and AQP9 as potential biomarkers of infective endocarditis. PLoS ONE 7, e31490. 22319637 10.1371/journal.pone.0031490 PMC3272041

[joa13112-bib-0064] Tobita K , Schroder EA , Tinney JP , et al. (2002) Regional passive ventricular stress‐strain relations during development of altered loads in chick embryo. Am J Physiol Heart Circ Physiol 282, H2386–2396. 12003850 10.1152/ajpheart.00879.2001

[joa13112-bib-0065] Tobita K , Garrison JB , Liu LJ , et al. (2005) Three‐dimensional myofiber architecture of the embryonic left ventricle during normal development and altered mechanical loads. Anat Rec A Discov Mol Cell Evol Biol 283, 193–201. 15678488 10.1002/ar.a.20133

[joa13112-bib-0066] Tomanek RJ , Hu N , Phan B , et al. (1999) Rate of coronary vascularization during embryonic chicken development is influenced by the rate of myocardial growth. Cardiovasc Res 41, 663–671. 10435038 10.1016/s0008-6363(98)00330-7

[joa13112-bib-0067] Valvona CJ , Fillmore HL , Nunn PB , et al. (2016) The regulation and function of lactate dehydrogenase A: therapeutic potential in brain tumor. Brain Pathol 26, 3–17. 26269128 10.1111/bpa.12299 PMC8029296

[joa13112-bib-0068] Wolfram JA , Donahue JK (2013) Gene therapy to treat cardiovascular disease. J Am Heart Assoc 2, e000119. 23963752 10.1161/JAHA.113.000119 PMC3828796

[joa13112-bib-0069] Wu KMG , Martin J , Finkelstein D . (2007) Globin reduction protocol: a method for processing whole blood RNA samples for improved array results, Santa Clara, CA: Affymetrix Technical Note.

[joa13112-bib-0070] Zaha VG , Young LH (2012) AMP‐activated protein kinase regulation and biological actions in the heart. Circ Res 111, 800–814. 22935535 10.1161/CIRCRESAHA.111.255505 PMC4397099

[joa13112-bib-0071] Zhao J , Yin M , Deng H , et al. (2016) Cardiac Gab1 deletion leads to dilated cardiomyopathy associated with mitochondrial damage and cardiomyocyte apoptosis. Cell Death Differ 23, 695–706. 26517531 10.1038/cdd.2015.143 PMC4986641

