# Supplementary material for: Characterisation of the developing heart in a pressure overloaded model utilising RNA sequencing to direct functional analysis
Source: J Anat. 2019 Nov 14;236(3):549–63. doi: 10.1111/joa.13112 (PMC7018637; doi:10.1111/joa.13112)
Supplement: Supplementary file 1 — Fig. S1. Relative mRNA quantity comparison in RNase‐treated (+RNase, globin‐depleted) and non‐RNase treated (–RNase, non‐globin‐treated) samples. An approximately one‐fold decrease in relative quantity was seen in globin‐depleted samples. Three untreated control HH29 hearts where used, with the same hearts used in both groups. Fig. S2. Normalised expression of GJA5 in non‐experimental control hearts for RNase‐treated (globin‐depleted) and non‐RNase‐treated (non‐globin‐depleted). Fig. S3. Analysis of reference gene stability by qPCR in HH35 OFT‐banded hearts by Genorm, BestKeeper and normFinder (n = 4 OFT‐banded, 4 sham, 4 untreated hearts). Fig. S4. qPCR reference gene primer optimisation. Fig. S5. RNA sequencing correlation in OFT‐banded and sham controls, using non‐corrected fold change (FC) values compared with qPCR, demonstrate a close relationship: r(16) = 0.98, P ≤ 0.0001 (n = 17). Fig. S6. OFT‐banded hearts show unbiased mapping to distinct clusters based on a profile of 45 genes linked to cardiac development and stress (all genes are shown in Tables 1 and S7) Table S1. Oligonucleotide design for globin gene hybridisation. Table S2. qPCR primer sequences Table S3. qPCR on globin‐depleted and non‐globin‐depleted mRNA show consistency of mean Cq Table S4. RNA sequencing of housekeeping and qPCR reference genes at HH29 shows consistent expression. Table S5. Comparison of targeted genes by RNA sequencing and qPCR at HH29 and HH35. Table S6. Molecular pathway analysis of significant differentially regulated genes in OFT‐banded hearts. Table S7. Fragment per kilobase per million (FPKM) of genes of biological interest from RNA sequenced OFT‐banded and sham control hearts Table S8. RNA sequencing gene regulation (OFT‐banded vs sham) by DEseq [file JOA-236-549-s001.docx]

**Supplementary material**


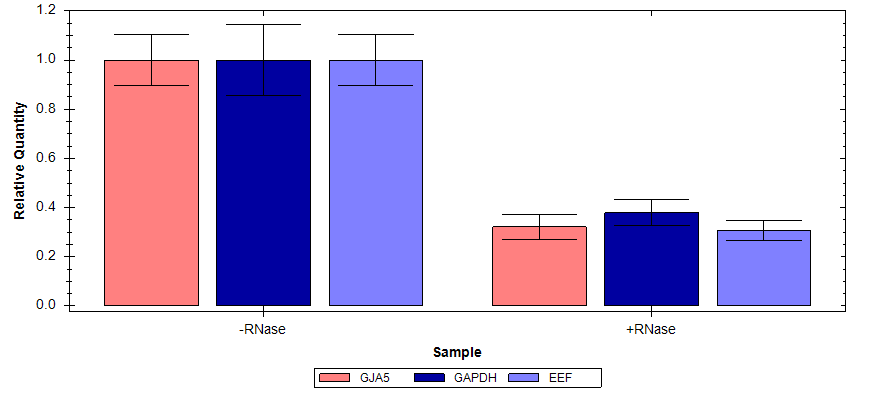


**Supplementary Figure 1.** Relative mRNA quantity comparison in RNase treated (+Rnase, globin depleted) and non-Rnase treated (-Rnase, non-globin treated) samples. A decrease in relative quantity was seen of approximately one fold in globin depleted samples. Three untreated control HH29 hearts where used, with the same hearts used in both groups.


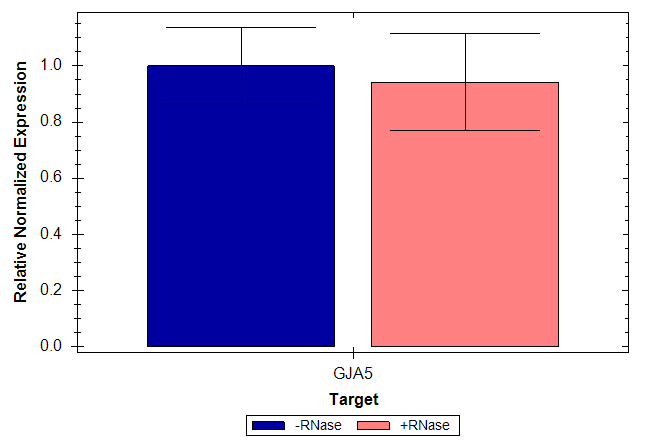


**Supplementary Figure 2.** Normalised expression of *GJA5* in non-experimental control hearts for Rnase treated (globin depleted) and non-Rnase treated (non-globin depleted). There was no significant difference in expression *(t*(4)=2.77,*p*=0.78), demonstrating globin depletion has no effect on relative expression.

**Supplementary Figure 3.** Analysis of reference gene stability by qPCR in HH35 OFT-banded hearts by Genorm, BestKeeper and normFinder (n= 4 OFT-banded, 4 sham, 4 untreated hearts).

A

B

c

GAPDH

RT: + + +

L 64 62 60


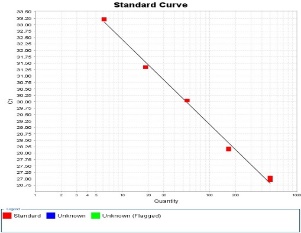

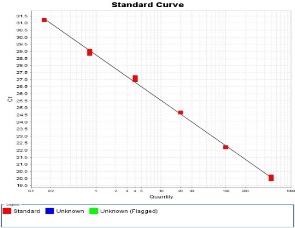

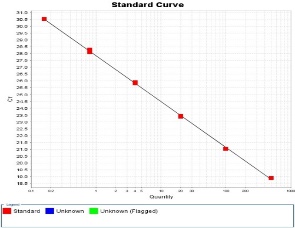

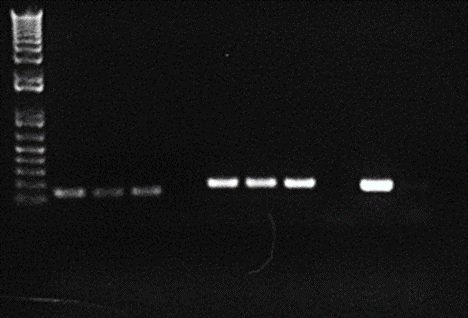

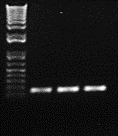

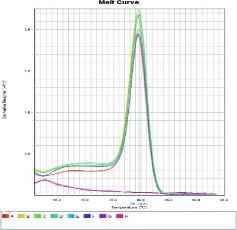

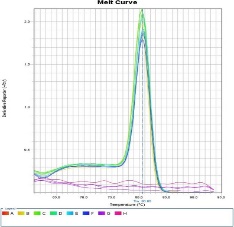

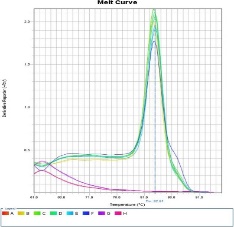


TBP EEF1A1 Con RT : + + + H_2_O + + + H_2_0 + -

L 64 62 60 64 62 60 62 62

**Supplementary Figure 4.** qPCR reference gene primer optimization**.** Data for electrophoresis gels (top), standard (middle) and melt (bottom) curves for gene-specific primers to reference genes (A) 179 bp *TBP* (105% Eff) (B) 218 bp *EEF1A1* (104% Eff*)* (C) 180 bp *GAPDH* (100% Eff) with all r^2^ values >0.98. L, 1 Kb plus ladder; +, reverse transcriptase positive; -, reverse transcriptase negative; Con, control (220 bp *GAPDH*); H_2_O, water control; Eff, efficiency.

**Supplementary Figure 5.** RNA sequencing correlation in OFT-banded and sham controls, using non-corrected fold change (FC) values compared to qPCR, demonstrate a close relationship (r(16)=0.98, p= <0.0001) (n = 17).


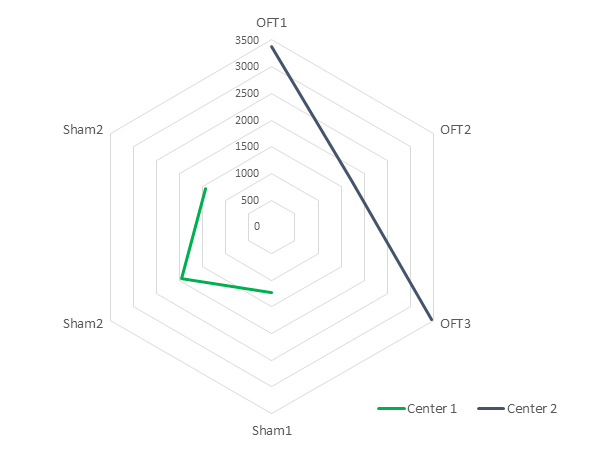


**Supplementary Figure 6.** OFT-banded hearts show unbiased mapping to distinct clusters based on a profile of 45 genes linked to cardiac development and stress (all genes are shown in Table 1 and Supplementary Table 7). The two group centres were identified by k-means clustering using FPKM values from sequenced OFT-banded (n=3) and sham hearts (n=3).

**Supplementary Table 1.** Oligonucleotide design for globin gene hybridisation.

| **Globin gene** | **Oligonucleotide sequence** | **Tm (°C)** |
| --- | --- | --- |
| ***HBAA*** | 5’- CAT CTC ATT TGG CTG CTC GCT GTC GGA GGG -3’ | 67.4 |
| ***HBAD*** | 5’ – TGT ACT TCT CAG CCA GCA CAG CAG ACA CGG – 3’ | 66.2 |
| ***HBB*** | 5’- TTACAGCCCAGCAGACCCCGAGGATGCCAG-3’ | 69.3 |
| ***HBG1*** | 5’ - CTG TGT CCT GCT CTG GGA GCT CAG TGG TAC – 3’ | 65.9 |
| ***HBG2*** | 5’ – TGT CAC AAG CTG TCA GCT CCG GAG CAT – 3’ | 65.4 |
| ***HBZ*** | 5’- CTC AGT CAG AAC AGA GGA AAT GCT GGA CAG -3’ | 61.7 |

**Supplementary Table 2.** qPCR primer sequences.

| **Gene** | **Forward sequence (5’-3’)** | **Reverse sequence (5’-3’)** | **Conc (µM)** | **T_m_ (°C)** | **Eff. (%)** | **R^2^** |
| --- | --- | --- | --- | --- | --- | --- |
| ***PVALB*** | GGCGATGACTGACGTGCTCA | TCAACCCCACCATCTCAAAGAACT | 0.375 | 62 | 107 | 0.989 |
| ***S100A11*** | GAGACAACCTCAAGCTCTCCAAG | GCGGGGTCCTTCTGATTCTTTG | 0.375 | 62 | 105 | 0.991 |
| ***ERNI*** | TTCACCGCAACAGTGGAGACT | GTAGAGGAGGATCGGAAGGC | 0.375 | 60 | 100 | 0.999 |
| ***ENS-1*** | CACCAGTCAGGACCCAAAGTG | CAATAAGTCTTGGGCTAGGAGGC | 0.375 | 62 | 103 | 0.998 |
| ***ITPK1*** | TCATCCTCGAAGCAGACCAGAA | GGTGAACAGATCCTCTCATCTTGC | 0.375 | 62 | 107 | 0.981 |
| ***TRPV4*** | GTGCTTCAGGGTGGATGAGGT | CGTTGGGCAGCTCTTGTTGAG | 0.375 | 62 | 103 | 0.994 |
| ***DNAJC6*** | CACCAAACCTGAACTGGATGCC | AGTGGGACCTTTACCTGCCAA | 0.375 | 62 | 103 | 0.999 |
| ***TBX3*** | AGTGCACCTGGAAGCCAAAGA | GTCGTCAGCCGCCACAATATC | 0.375 | 62 | 98 | 0.999 |
| ***TBP*** | CACATCACAGCTGCCCACCA | GAGAACCACGTACTACTGCGCTT | 0.375 | 62 | 105 | 0.998 |
| ***EEF1A1*** | TGGCTTCAGGACACCAGTTTC | GCTCTAACATGCCCTGGTTCAAG | 0.375 | 62 | 103 | 0.998 |
| ***GAPDH*** | AGACGGTGGATGGCCCCTCT | AGACGGTGGATGGCCCCTCT | 0.375 | 62 | 101 | 0.999 |
| ***MYL1*** | TGCCAAGATTACCCTGAGCCA | TCCTCTTTGCTGGGGTTGCC | 0.375 | 62 | 99 | 0.999 |
| ***PRKM*** | GACGCTTCGATGACGCTGTG | TGTGACCGCTCTTGGTGGAG | 0.375 | 62 | 104 | 0.984 |
| ***LDHB*** | TCCTCTTTGCTGGGGTTGCC | TCCTCTTTGCTGGGGTTGCC | 0.375 | 62 | 103 | 0.987 |
| ***PRKAG3*** | TCAACATCCTGCACCGCTACT | TCAACATCCTGCACCGCTACT | 0.375 | 62 | 101 | 0.998 |
| ***HDAC7*** | CGCCTGAAACTGGACAATGGG | AGTGCAGCTCATTCCAGATGGT | 0.375 | 62 | 103 | 0,994 |
| ***TNNC2*** | AGTGCAGCTCATTCCAGATGGT | AGTGCAGCTCATTCCAGATGGT | 0.375 | 62 | 105 | 0.997 |
| ***HBAA*** | GTATGGCGCCGAGACCCTGG | TGTGGGCATGGAGGTCGCTG | 0.375 | 62 | 108 | 0.987 |
| ***HBAD*** | GCAAGAAGGTGTTGGGTGCCC | AGCCAGCACAGCAGACACGG | 0.375 | 62 | 106 | 0.994 |
| ***HBB*** | AAGCCGTGAAGAACCTGGACAA | CAAATTGGCAGGCAGGAGTGAAA | 0.375 | 62 | 108 | 0.996 |
| ***HBG1*** | GGCCAGGCTGCTGATCGTCTA | GTTCTCGGGGTCCACGTGCA | 0.375 | 62 | 102 | 0.992 |
| ***HBG2*** | TGTGGACCCCGAGAACTTCA | GGTGCTCCGTGATCTTTGGT | 0.375 | 62 | 104 | 0.986 |
| ***HBZ*** | TGAGAAGGCTGCCGTGACCAC | TCTTCACAGCTTCCCCAATGGCA | 0.375 | 62 | 105 | 0.993 |
| ***CX42*** | GTGGGGTGCAGTGAGACAGA | CAGACCTTCCCCACCACTGT | 0.375 | 62 | 104 | 0.998 |

**Supplementary Table 3.** qPCR on globin depleted and non-globin depleted mRNA show consistency of mean Cq.

| **Target** | **Sample** | **Mean Cq** | **Mean efficiency corrected Cq** | **Normalized expression** | **Relative normalized expression** | **Regulation** | **Compared to regulation threshold** | **P-value** |
| --- | --- | --- | --- | --- | --- | --- | --- | --- |
| ***HBAA*** | GD | 33.20 | 32.96 | 0.00208 | 0.00008 | -13073.80327 | Down regulated | 0.000000 |
| ***HBAD*** | GD | 33.12 | 33.12 | 0.00185 | 0.00005 | -21787.81025 | Down regulated | 0.000000 |
| ***HBB*** | GD | 33.48 | 34.90 | 0.00054 | 0.00045 | -2202.78117 | Down regulated | 0.000000 |
| ***HBG1*** | GD | 31.78 | 32.01 | 0.00399 | 0.00013 | -7549.23437 | Down regulated | 0.000000 |
| ***HBG2*** | GD | 31.41 | 32.53 | 0.00279 | 0.00005 | -21442.95845 | Down regulated | 0.000000 |
| ***HBZ*** | GD | 33.91 | 34.88 | 0.00055 | 0.00001 | -184727.82727 | Down regulated | 0.000000 |
| ***HBAA*** | Non-GD | 15.50 | 15.39 | 27.12853 | 1.00000 | 1.00000 |  |  |
| ***HBAD*** | Non-GD | 14.82 | 14.82 | 40.30894 | 1.00000 | 1.00000 |  |  |
| ***HBB*** | Non-GD | 19.10 | 19.91 | 1.18363 | 1.00000 | 1.00000 |  |  |
| ***HBG1*** | Non-GD | 15.13 | 15.24 | 30.15254 | 1.00000 | 1.00000 |  |  |
| ***HBG2*** | Non-GD | 13.76 | 14.25 | 59.92080 | 1.00000 | 1.00000 |  |  |
| ***HBZ*** | Non-GD | 13.12 | 13.49 | 101.31535 | 1.00000 | 1.00000 |  |  |

GD, globin depleted; Non-GD, non-globin depleted.

**Supplementary Table 4.** RNA sequencing of house keeping and

qPCR reference genes at HH29 shows consistent expression.

| **Gene** | **Heart** | **FPKM** | **Log_2_FC** | ***P*-value** |
| --- | --- | --- | --- | --- |
| ***EEF1A1*** | **OFT 1**  **OFT 2**  **OFT 3**  **Sham 1**  **Sham 2**  **Sham 3** | **6046**  **5733**  **6009**  **6191**  **6471**  **6543** | **-0.04** | **0.9** |
| ***GAPDH*** | **OFT 1**  **OFT 2**  **OFT 3**  **Sham 1**  **Sham 2**  **Sham 3** | **14188**  **13138**  **12395**  **13347**  **12907**  **12701** | **0.09** | **0.8** |
| ***TBP*** | **OFT 1**  **OFT 2**  **OFT 3**  **Sham 1**  **Sham 2**  **Sham 3** | **46**  **54**  **51**  **51**  **49**  **49** | **0.07** | **0.7** |

**Supplementary Table 5.** Comparison of targeted genes by RNA sequencing and qPCR at HH29 and HH35.

| **Gene** | **RNA sequencing HH29** | | | | **qPCR HH29** | | | | **qPCR HH35** | | | |
| --- | --- | --- | --- | --- | --- | --- | --- | --- | --- | --- | --- | --- |
|  | **Mean fold change** | **Mean log_2_ fold change (cor)** | **SEM** | ***P* value (cor)** | **Mean fold change** | **Mean log_2_ fold change** | **SEM** | ***P* value** | **Mean fold change** | **Mean log_2_ fold change** | **SEM** | ***P* value** |
| ***PRKAG3***  ***LDHB***  ***HDAC7***  ***MYL1***  ***TNNC2***  ***ENS-1***  ***ERNI***  ***PVALB***  ***S100A11***  ***DNAJC6***  ***TBX3*** | 0.652  1.6  0.75  1.52  1.8  0.4  0.48  1.85  1.49  0.64  1.35 | **-0.46**  **0.64**  **-0.37**  **0.54**  **0.75**  **-0.91**  **-0.76**  **0.7**  **0.52**  **-0.48**  **0.4** | 0.13  0.13  0.09  0.15  0.16  0.16  0.15  0.17  0.14  0.13  0.15 | 0.06  <0.05  0.06  <0.05  <0.05  <0.05  <0.05  <0.05  <0.05  <0.05  0.2 | 0.68  1.54  0.7  1.57  1.6  0.39  0.47  1.76  1.6  0.59  1.3 | **-0.55**  **0.62**  **-0.51**  **0.65**  **0.65**  **-1.35**  **-1.08**  **0.81**  **0.67**  **-0.75**  **0.38** | 0.05  0.24  0.04  0.12  0.19  0.02  0.04  0.17  0.07  0.03  0.05 | <0.05  <0.05  0.12  0.13  <0.05  <0.05  <0.05  <0.05  <0.05  <0.05  0.2 | 0.44  1.10  0.75  2.66  1.54  0.72  0.45  2.249  2.11  0.76  1.07 | **-1.18**  **0.13**  **-0.41**  **1.41**  **0.62**  **-0.47**  **-1.15**  **1.16**  **1.07**  **-0.41**  **0.14** | 0.04  0.06  0.04  0.2  0.16  0.16  0.10  0.37  0.18  0.07  0.08 | <0.05  0.61  0.22  <0.05  0.32  0.63  0.22  <0.05  <0.05  0.15  0.84 |

Cor, corrected.

**Supplementary Table 6.** Molecular pathway analysis of significant differentially regulated genes in OFT-banded hearts.

| **Implicated pathway name** | **Molecules found** | **Molecule pathway total** | **Molecule ratio** | **Molecule pValue** | **Molecule FDR*** | **Reactions found** | **Reactions total** | **Reactions ratio** |
| --- | --- | --- | --- | --- | --- | --- | --- | --- |
| Activation of PPARGC1A (PGC-1alpha) by phosphorylation | 2 | 13 | 9E-04 | 0.004 | 0.420 | 1 | 2 | 1.7E-04 |
| Striated Muscle Contraction | 2 | 40 | 3E-03 | 0.008 | 0.420 | 4 | 4 | 3.3E-04 |
| Activation of AMPK downstream of NMDARs | 2 | 34 | 2E-03 | 0.009 | 0.420 | 3 | 3 | 2.5E-04 |
| Adenosine P1 receptors | 1 | 5 | 4E-04 | 0.009 | 0.420 | 1 | 2 | 1.7E-04 |
| Energy dependent regulation of mTOR by LKB1-AMPK | 2 | 36 | 3E-03 | 0.011 | 0.420 | 5 | 7 | 5.8E-04 |
| Apoptosis induced DNA fragmentation | 1 | 13 | 9E-04 | 0.030 | 0.420 | 2 | 12 | 9.9E-04 |
| Muscle contraction | 3 | 256 | 2E-02 | 0.039 | 0.420 | 10 | 41 | 3.4E-03 |
| Neurophilin interactions with VEGF and VEGFR | 0 | 4 | 3E-04 | 0.050 | 0.420 | 1 | 2 | 1.7E-04 |
| VEGF binds to VEGFR leading to receptor dimerization | 0 | 8 | 6E-04 | 0.050 | 0.420 | 1 | 3 | 2.5E-04 |
| VEGF ligand-receptor interactions | 0 | 8 | 6E-04 | 0.050 | 0.420 | 1 | 4 | 3.3E-04 |
| TP53 Regulates Metabolic Genes | 2 | 125 | 9E-03 | 0.059 | 0.420 | 2 | 34 | 2.8E-03 |
| Translocation of SLC2A4 (GLUT4) to the plasma membrane | 2 | 79 | 6E-03 | 0.063 | 0.420 | 3 | 15 | 1.2E-03 |
| COPI-dependent Golgi-to-ER retrograde traffic | 2 | 107 | 8E-03 | 0.066 | 0.420 | 2 | 11 | 9.1E-04 |
| Regulation of TP53 Activity through Phosphorylation | 2 | 95 | 7E-03 | 0.070 | 0.420 | 1 | 26 | 2.1E-03 |
| COPI-mediated anterograde transport | 2 | 107 | 8E-03 | 0.073 | 0.420 | 2 | 12 | 9.9E-04 |
| TRAF3 deficiency - HSE | 0 | 3 | 2E-04 | 0.073 | 0.420 | 1 | 1 | 8.3E-05 |
| Other semaphorin interactions | 0 | 19 | 1E-03 | 0.075 | 0.420 | 1 | 9 | 7.4E-04 |
| Chylomicron remodeling | 0 | 17 | 1E-03 | 0.077 | 0.420 | 1 | 3 | 2.5E-04 |
| Fibronectin matrix formation | 0 | 7 | 5E-04 | 0.078 | 0.420 | 2 | 3 | 2.5E-04 |
| RUNX1 and FOXP3 control the development of regulatory T lymphocytes (Tregs) | 0 | 17 | 1E-03 | 0.091 | 0.420 | 1 | 20 | 1.7E-03 |
| Macroautophagy | 2 | 103 | 7E-03 | 0.093 | 0.420 | 8 | 54 | 4.5E-03 |
| AMPK inhibits chREBP transcriptional activation activity | 1 | 11 | 8E-04 | 0.096 | 0.420 | 3 | 4 | 3.3E-04 |
| MECP2 regulates transcription factors | 0 | 10 | 7E-04 | 0.096 | 0.420 | 1 | 8 | 6.6E-04 |
| Synthesis of pyrophosphates in the cytosol | 1 | 29 | 2E-03 | 0.099 | 0.420 | 2 | 15 | 1.2E-03 |
| Clathrin-mediated endocytosis | 2 | 161 | 1E-02 | 0.102 | 0.420 | 7 | 34 | 2.8E-03 |

PRKAG2 added instead of PRKAG3 due to embryonic replacement in chick, with subsequently comparison analysis to pathways in human mature heart tissue. Analysis by Reactome (Fabregat et al., 2017).

*False discovery rate.

**Supplementary Table 7**. Fragment per kilobase per million (FPKM) of genes of biological interest from RNA sequenced OFT-banded and sham control hearts.

| **Gene symbol (name)** | **Biological function** | **OFT-Banded FPKM** | | | | | | **SHAM FPKM** | | | | |
| --- | --- | --- | --- | --- | --- | --- | --- | --- | --- | --- | --- | --- |
|  |  | **1** | | **2** | **3** | | **Mean** | **1** | **2** | **3** | | **Mean** |
| ***ACTA1***  *(*Actin, alpha 1, skeletal muscle) | Contractile apparatus | 2463 | | 4681 | 5433 | | **4192** | 3808 | 3216 | 3912 | | **3645** |
| ***PLN***  *(*Phospholamban) | Modulates the contractility of the heart | 613 | | 617 | 740 | | **656** | 472 | 618 | 451 | | **513** |
| ***MYH7***  *(*B*eta*  *myosin, heavy chain)* | 'Slow' ATPase ventricular myosin in mature heart | 2410 | | 2781 | 3227 | | **2806** | 2183 | 3363 | 2158 | | **2568** |
| ***MYH6***  *(Alpha myosin heavy chain)* | 'Fast' ATPase atrial myosin in the mature heart; however, during development higher expression of beta myosin heavy chain is seen throughout the heart | 263 | | 229 | 283 | | **258** | 365 | 316 | 218 | | **300** |
| ***ANKRD1***  *(*Ankyrin repeat domain 1) | Transcription factor | 29 | | 27 | 197 | | **84** | 23 | 20 | 11 | | **18** |
| ***DMD***  *(*Dystrophin) | Anchor protein | 10.9 | | 10 | 12.5 | | **11.1** | 14.2 | 14.1 | 12.8 | | **13.7** |
| ***BIN1***  *(*Bridging integrator 1) | MYC-interacting protein | 3.1 | | 6.8 | 6.7 | | **5.5** | 5.0 | 8.5 | 9.5 | | **7.7** |
| ***NOS2***  *(*Nitric oxide synthase 2) | Produces nitric oxide | 6.2 | 8.1 | | | 7.3 | **7.2** | 6.8 | 8.4 | 9 | **8** | |
| ***MYL4***  (Myosin, light chain 4) | Regulatory light chain of myosin | 730 | 781 | | | 740 | **750** | 849 | 720 | 836 | **801** | |
| ***PRKAG3***  (Protein kinase AMP-activated non-catalytic subunit gamma 3) | AMP/ATP-binding regulatory γ subunit of AMP-activated protein kinase (AMPK) | 8.81 | 8.58 | | | 8.47 | **8.62** | 11.36 | 12.97 | 15.33 | **13.22** | |
| ***PRKAG1***  *(*Protein kinase AMP-activated non-catalytic subunit gamma 1) | AMP/ATP-binding regulatory γ subunit of AMP-activated protein kinase (AMPK) | 47.46 | 46.12 | | | 40.56 | **42.38** | 42.51 | 52.25 | 49.90 | **48.22** | |
| ***PRKAG2***  *(*Protein kinase AMP-activated non-catalytic subunit gamma 2) | AMP/ATP-binding regulatory γ subunit of AMP-activated protein kinase (AMPK) | 0.13 | 0.11 | | | 0.28 | **0.17** | 0.00 | 0.10 | 0.20 | **0.15** | |
| ***S100A1***  *(*S100 calcium binding protein A1*)* | Calcium ion binding/ contractility | 123.94 | 102.60 | | | 100 | **108.84** | 98.73 | 85.63 | 86.82 | **90.39** | |
| ***S100A11***  *(*S100 calcium binding protein A11) | Calcium ion binding/ contractility | 263 | 249 | | | 214 | **242** | 185 | 156 | 146 | **162** | |
| ***LDHB***  *(*Lactate dehydrogenase B) | Metabolic catalytic activity | 3391 | 3203 | | | 2527 | **3040** | 1883 | 1873 | 1930 | **1895** | |
| ***LDHA***  *(Lactate dehydrogenase A)* | Metabolic catalytic activity | 413 | 691 | | | 436 | **513** | 469 | 421 | 547 | **479** | |
| ***HDAC7***  *(Histone deacetylase 7)* | Histones deacetylation/regulator of expression | 16.82 | 18.92 | | | 20.10 | **18.61** | 23.15 | 24.35 | 26.36 | **24.62** | |
| ***STK11***  (Serine/threonine kinase 11) | AMPK/ metabolic regulator | 20.56 | 24.66 | | | 25.42 | **23.54** | 24.83 | 30.19 | 35.99 | **30.33** | |
| ***TRPV4***  *(*Transient receptor potential cation channel subfamily V member 4*)* | Ion channel protein | 2.07 | 234 | | | 2.12 | **2.17** | 3.38 | 3.19 | 3.88 | **3.48** | |
| ***PFKM***  (Phosphofructokinase) | Metabolic enzyme in glycolysis | 33.06 | 45.17 | | | 38.69 | **38.97** | 47.97 | 62.12 | 54.64 | **54.91** | |
| ***TBX3***  (T-box 3) | Transcription factor | 10.31 | 9.06 | | | 8.25 | **9.20** | 5.64 | 7.27 | 7.56 | **6.82** | |

**Supplementary Table 8.** RNA sequencing gene regulation (OFT-banded vs sham) by DEseq.

| **Gene ID** | **Gene name** | **Log^2^ fold change** | **lfcSE** | **Adjusted *p-*value** |
| --- | --- | --- | --- | --- |
| NM_001080873 | ENS-1 | -0.91 | 0.16 | 2.98E-05 |
| NM_001302201 | TIMM10 | 0.72 | 0.13 | 2.98E-05 |
| NM_001080874 | ERNI | -0.76 | 0.15 | 4.79E-04 |
| NM_205068 | GBX2 | 0.83 | 0.17 | 8.27E-04 |
| NM_204177 | LDHB | 0.64 | 0.13 | 1.23E-03 |
| NM_205450 | TNNC2 | 0.75 | 0.16 | 1.34E-03 |
| NM_001079490 | PNO1 | 0.59 | 0.13 | 3.62E-03 |
| NM_001159315 | PVALB | 0.70 | 0.17 | 1.91E-02 |
| NM_205087 | ADORA2B | -0.74 | 0.18 | 2.08E-02 |
| NM_001012588 | ITPK1 | -0.68 | 0.17 | 2.34E-02 |
| NM_205160 | GLRX | -0.71 | 0.18 | 3.33E-02 |
| NM_204304 | PDCD4 | -0.41 | 0.11 | 3.52E-02 |
| NM_001030626 | PIAS2 | -0.36 | 0.10 | 4.37E-02 |
| NM_001097526 | MYL10 | 0.67 | 0.18 | 4.37E-02 |
| NM_205448 | TGM2 | -0.38 | 0.10 | 4.37E-02 |
| NM_001177415 | DNAJC6 | -0.48 | 0.13 | 4.40E-02 |
| NM_001031478 | RAP1GAP2 | -0.57 | 0.15 | 4.46E-02 |
| NM_001044632 | MYL1 | 0.54 | 0.15 | 4.46E-02 |
| NM_001044673 | H1F0 | -0.64 | 0.18 | 4.46E-02 |
| NM_001130387 | IFNGR1 | 0.55 | 0.15 | 4.46E-02 |
| NM_001030857 | ARFGAP1 | -0.47 | 0.13 | 4.46E-02 |
| NM_205166 | S100A11 | 0.52 | 0.14 | 4.46E-02 |
| NM_001031038 | LBH | 0.60 | 0.17 | 4.86E-02 |
| NM_001100289 | CDH8 | -0.60 | 0.16 | 4.86E-02 |
| NM_204818 | GSTA4 | -0.65 | 0.18 | 4.88E-02 |
| NM_205495 | LGALS1 | 0.64 | 0.18 | 4.88E-02 |
| NM_001079501 | LLPH | 0.58 | 0.16 | 4.97E-02 |
| NM_001006388 | MRPL15 | 0.44 | 0.12 | 5.66E-02 |
| NM_001012534 | TOB2 | -0.36 | 0.10 | 5.66E-02 |
| NM_001031608 | MBLAC2 | -0.62 | 0.18 | 5.66E-02 |
| NM_001024579 | WISP1 | 0.40 | 0.12 | 6.22E-02 |
| NM_001031258 | PRKAG3 | -0.46 | 0.13 | 6.22E-02 |
| NM_001097540 | DAG1 | 0.42 | 0.12 | 6.39E-02 |
| NM_001278025 | TINAG | 0.52 | 0.15 | 6.96E-02 |
| NM_001031402 | HDAC7 | -0.31 | 0.09 | 6.98E-02 |
| NM_001006423 | LOC422090 | -0.55 | 0.16 | 7.18E-02 |
| NM_001030637 | HAGHL | -0.42 | 0.12 | 7.48E-02 |
| NM_001097637 | NDUFA5 | 0.46 | 0.14 | 7.48E-02 |
| NM_204915 | EYA2 | 0.47 | 0.14 | 7.59E-02 |
| NM_001305209 | STK10 | -0.56 | 0.17 | 8.04E-02 |
| NM_001012532 | EHMT1 | -0.33 | 0.10 | 8.46E-02 |
| NM_205402 | GNAI2 | 0.39 | 0.12 | 8.46E-02 |
| NM_001006527 | MCM6 | 0.38 | 0.11 | 8.54E-02 |
| NM_001030628 | WDR24 | -0.34 | 0.10 | 8.67E-02 |
| NM_205459 | MYLK | 0.60 | 0.18 | 8.67E-02 |
| NM_213579 | GEM | 0.42 | 0.13 | 8.67E-02 |
| NM_204316 | ADORA1 | -0.41 | 0.13 | 8.74E-02 |
| NM_001010842 | HSP25 | 0.60 | 0.18 | 8.87E-02 |
| NM_001277912 | PPIH | 0.34 | 0.10 | 8.87E-02 |
| NM_001006460 | MXD4 | -0.57 | 0.17 | 9.02E-02 |
| NM_001277910 | DHRS3 | 0.35 | 0.11 | 9.02E-02 |
| NM_001199549 | SLC38A4 | 0.58 | 0.18 | 9.15E-02 |
| NM_001030789 | RCSD1 | 0.47 | 0.15 | 9.42E-02 |
| NM_001031279 | SLC6A9 | -0.53 | 0.16 | 9.42E-02 |
| NM_001097539 | LRRN1 | -0.51 | 0.16 | 9.88E-02 |
| NM_001114503 | LOC420716 | 0.55 | 0.17 | 9.88E-02 |
| NM_001115017 | SELO | -0.56 | 0.18 | 9.95E-02 |
| NM_205021 | COL12A1 | 0.49 | 0.15 | 1.00E-01 |
| NM_001282329 | C10H15orf61 | 0.46 | 0.14 | 1.04E-01 |
| NM_001008453 | CCND3 | -0.52 | 0.16 | 1.06E-01 |
| NM_001030752 | WDR91 | -0.26 | 0.08 | 1.06E-01 |
| NM_001195561 | PRORSD1P | 0.38 | 0.12 | 1.06E-01 |
| NM_001199447 | ANGPT1 | -0.34 | 0.11 | 1.06E-01 |
| NM_001277918 | HDDC2 | 0.49 | 0.15 | 1.06E-01 |
| NM_204287 | THYN1 | 0.34 | 0.11 | 1.06E-01 |
| NM_001039299 | CENPU | 0.43 | 0.14 | 1.11E-01 |
| NM_204692 | TRPV4 | -0.47 | 0.15 | 1.14E-01 |
| NM_001001462 | SDC2 | 0.45 | 0.15 | 1.17E-01 |
| NM_001006290 | ADA | -0.56 | 0.18 | 1.17E-01 |
| NM_001030682 | SURF6 | 0.55 | 0.18 | 1.17E-01 |
| NM_001031527 | B3GAT2 | 0.48 | 0.16 | 1.17E-01 |
| NM_001099354 | TRIM7.2 | -0.27 | 0.09 | 1.17E-01 |
| NM_001111259 | NPPC | 0.54 | 0.17 | 1.17E-01 |
| NM_204467 | RHCE | -0.45 | 0.15 | 1.17E-01 |
| NM_204624 | MOXD1 | 0.36 | 0.12 | 1.17E-01 |
| NM_205086 | FTH1 | -0.47 | 0.15 | 1.17E-01 |
| NM_001277878 | IMMP1L | 0.42 | 0.14 | 1.22E-01 |
| NM_204849 | HBEGF | 0.56 | 0.18 | 1.22E-01 |
| NM_001030599 | TPRA1 | -0.30 | 0.10 | 1.28E-01 |
| NM_204228 | MYH1B | 0.33 | 0.11 | 1.28E-01 |
| NM_204158 | CALD1 | 0.43 | 0.14 | 1.33E-01 |
| NM_001031233 | TRIM8 | -0.28 | 0.09 | 1.33E-01 |
| NM_205491 | HSPA5 | 0.34 | 0.11 | 1.36E-01 |
| NM_001195785 | LOC100502566 | 0.46 | 0.15 | 1.37E-01 |
| NM_204141 | RPL22 | 0.49 | 0.16 | 1.37E-01 |
| NM_001007917 | MPP1 | -0.49 | 0.17 | 1.45E-01 |
| NM_001030580 | CBFA2T3 | -0.53 | 0.18 | 1.45E-01 |
| NM_204223 | PFKM | -0.37 | 0.13 | 1.45E-01 |
| NM_001030688 | ASB6 | -0.49 | 0.17 | 1.45E-01 |
| NM_001044672 | CAPN1 | -0.53 | 0.18 | 1.45E-01 |
| NM_001159425 | MED6 | 0.26 | 0.09 | 1.45E-01 |
| NM_204383 | FTL | -0.52 | 0.18 | 1.45E-01 |
| NM_001199486 | CSRP3 | 0.39 | 0.13 | 1.52E-01 |
| NM_205317 | CA2 | 0.45 | 0.16 | 1.54E-01 |
| NM_001030674 | BRD2 | -0.36 | 0.12 | 1.55E-01 |
| NM_001012896 | AP2A2 | -0.22 | 0.08 | 1.58E-01 |
| NM_001030559 | KATNB1 | -0.37 | 0.13 | 1.58E-01 |
| NM_001030946 | TP53INP1 | -0.39 | 0.14 | 1.58E-01 |
| NM_001030998 | GMPR | -0.50 | 0.17 | 1.58E-01 |
| NM_001033645 | EPHX2 | -0.48 | 0.16 | 1.58E-01 |
| NM_001167732 | ACE | -0.34 | 0.12 | 1.58E-01 |
| NM_001197326 | ATP6V1H | 0.41 | 0.14 | 1.58E-01 |
| NM_001199441 | CSRP2BP | -0.30 | 0.10 | 1.58E-01 |
| NM_001271988 | COL9A2 | 0.36 | 0.13 | 1.58E-01 |
| NM_001277355 | LSM8 | 0.44 | 0.15 | 1.58E-01 |
| NM_001305113 | KCNH6 | -0.34 | 0.12 | 1.58E-01 |
| NM_204216 | DDB1 | -0.28 | 0.10 | 1.58E-01 |
| NM_205005 | CALM | 0.26 | 0.09 | 1.58E-01 |
| NM_001292085 | C10H15ORF15 | 0.47 | 0.17 | 1.62E-01 |
| NM_001031034 | PYGB | -0.42 | 0.15 | 1.66E-01 |
| NM_001277654 | HYPK | 0.45 | 0.16 | 1.66E-01 |
| NM_001031186 | ORAOV1 | 0.34 | 0.12 | 1.71E-01 |
| NM_001030671 | TRIM27.1 | -0.50 | 0.18 | 1.71E-01 |
| NM_001030754 | HEBP1 | -0.49 | 0.17 | 1.71E-01 |
| NM_001044652 | PACSIN3 | 0.40 | 0.14 | 1.71E-01 |
| NM_204556 | HOPX | 0.49 | 0.17 | 1.71E-01 |
| NM_001030633 | C14H17ORF103 | -0.41 | 0.14 | 1.73E-01 |
| NM_001038497 | SLC24A5 | -0.28 | 0.10 | 1.74E-01 |
| NM_001044643 | SLC9A1 | -0.36 | 0.13 | 1.74E-01 |
| NM_001001468 | IFRD1 | 0.31 | 0.11 | 1.74E-01 |
| NM_001199630 | KCNMB4 | 0.51 | 0.18 | 1.74E-01 |
| NM_205347 | PLS1 | -0.46 | 0.16 | 1.74E-01 |
| NM_001025441 | SELK | 0.44 | 0.16 | 1.76E-01 |
| NM_204196 | FECH | -0.50 | 0.18 | 1.77E-01 |
| NM_001034821 | ATG9A | -0.32 | 0.12 | 1.78E-01 |
| NM_204561 | SMAD2 | -0.41 | 0.15 | 1.82E-01 |
| NM_204909 | RHOB | 0.33 | 0.12 | 1.82E-01 |
| NM_205494 | TAGLN | 0.46 | 0.17 | 1.82E-01 |
| NM_001030854 | TTPAL | -0.31 | 0.11 | 1.83E-01 |
| NM_204955 | ACAN | 0.50 | 0.18 | 1.86E-01 |
| NM_001113289 | MDK | 0.39 | 0.14 | 1.87E-01 |
| NM_001285926 | CMSS1 | 0.41 | 0.15 | 1.87E-01 |
| NM_001291758 | SCG5 | 0.50 | 0.18 | 1.87E-01 |
| NM_206844 | CITED2 | -0.47 | 0.17 | 1.87E-01 |
| NM_001079718 | UBN1 | -0.39 | 0.14 | 1.88E-01 |
| NM_001199639 | RAB3IL1 | -0.49 | 0.18 | 1.88E-01 |
| NM_001001299 | BVES | 0.24 | 0.09 | 1.88E-01 |
| NM_001031192 | DGKZ | -0.23 | 0.08 | 1.88E-01 |
| NM_001159698 | HSPH1 | 0.39 | 0.14 | 1.88E-01 |
| NM_205486 | HMGB2 | 0.45 | 0.16 | 1.88E-01 |
| NM_001278047 | GNG11 | 0.46 | 0.17 | 1.88E-01 |
| NM_001030547 | ADAL | -0.48 | 0.18 | 1.95E-01 |
| NM_204482 | HS6ST1 | 0.28 | 0.10 | 1.95E-01 |
| NM_001031597 | PABPC1 | -0.26 | 0.10 | 1.98E-01 |
| NM_001006346 | FABP5 | 0.48 | 0.18 | 2.05E-01 |
| NM_001270878 | TBX3 | 0.40 | 0.15 | 2.05E-01 |
| NM_204630 | ADCY9 | -0.35 | 0.13 | 2.05E-01 |
| NM_205352 | TAL1 | -0.49 | 0.18 | 2.05E-01 |
| NM_001012575 | TMLHE | -0.37 | 0.14 | 2.10E-01 |
| NM_001012942 | XPO7 | -0.38 | 0.14 | 2.16E-01 |
| NM_001277858 | LOC425783 | -0.47 | 0.18 | 2.22E-01 |
| NM_001080860 | TUBB4B | 0.25 | 0.10 | 2.25E-01 |
| NM_001167752 | MB | 0.30 | 0.11 | 2.25E-01 |
| NM_001305064 | CREG1 | -0.40 | 0.15 | 2.25E-01 |
| NM_001001744 | IRX4 | -0.26 | 0.10 | 2.25E-01 |
| NM_001030699 | FAM18B1 | 0.22 | 0.08 | 2.25E-01 |
| NM_001302139 | LYRM9 | 0.40 | 0.15 | 2.26E-01 |
| NM_001199686 | ZFYVE1 | -0.34 | 0.13 | 2.32E-01 |
| NM_204097 | TENM2 | -0.39 | 0.15 | 2.33E-01 |
| NM_001199325 | DNAJC2 | 0.43 | 0.17 | 2.36E-01 |
| NM_001285979 | CLCN6 | -0.29 | 0.11 | 2.36E-01 |
| NM_204779 | STAT5B | -0.42 | 0.16 | 2.45E-01 |
| NM_205034 | CACNA1D | -0.33 | 0.13 | 2.45E-01 |
| NM_001278079 | RCAN1 | 0.40 | 0.15 | 2.45E-01 |
| NM_205219 | E2F1 | -0.38 | 0.15 | 2.45E-01 |
| NM_001159345 | NR3C2 | -0.27 | 0.11 | 2.46E-01 |
| NM_001190896 | DNAJC24 | 0.44 | 0.17 | 2.46E-01 |
| NM_001030922 | TFEB | -0.21 | 0.08 | 2.47E-01 |
| NM_001198680 | EMC2 | 0.35 | 0.14 | 2.47E-01 |
| NM_001277626 | MTHFS | 0.27 | 0.10 | 2.48E-01 |
| NM_204186 | SAT1 | -0.38 | 0.15 | 2.52E-01 |
| NM_001030872 | AURKAIP1 | 0.39 | 0.15 | 2.54E-01 |
| NM_001033869 | VDAC1 | 0.45 | 0.17 | 2.55E-01 |
| NM_001079478 | RP11-292K15.2 | 0.32 | 0.12 | 2.55E-01 |
| NM_001005805 | WDR61 | 0.39 | 0.15 | 2.64E-01 |
| NM_205410 | PLN | 0.35 | 0.14 | 2.64E-01 |
| NM_001139477 | MEIS1 | -0.40 | 0.16 | 2.65E-01 |
| NM_204532 | CENPI | 0.28 | 0.11 | 2.72E-01 |
| NM_001005800 | CTD-2116N17.1 | 0.38 | 0.15 | 2.77E-01 |
| NM_001030719 | FLOT2 | -0.27 | 0.11 | 2.77E-01 |
| NM_001278156 | TMEM183A | -0.31 | 0.12 | 2.77E-01 |
| NM_204332 | CBX1 | 0.35 | 0.14 | 2.77E-01 |
| NM_001003797 | GATA2 | -0.45 | 0.18 | 2.84E-01 |
| NM_001006367 | ACAD11 | 0.36 | 0.15 | 2.84E-01 |
| NM_001006495 | NCOA4 | -0.33 | 0.13 | 2.84E-01 |
| NM_001006514 | LSS | -0.37 | 0.15 | 2.84E-01 |
| NM_001012933 | DAPP1 | -0.31 | 0.12 | 2.84E-01 |
| NM_001030635 | FLII | -0.30 | 0.12 | 2.84E-01 |
| NM_001031031 | BCL11A | -0.29 | 0.11 | 2.84E-01 |
| NM_001031260 | UMPS | 0.24 | 0.10 | 2.84E-01 |
| NM_001293097 | SLC8A3 | -0.34 | 0.14 | 2.84E-01 |
| NM_204633 | SPINZ | -0.25 | 0.10 | 2.84E-01 |
| NM_001030643 | DAGLB | -0.34 | 0.14 | 2.84E-01 |
| NM_001199193 | MAPT | -0.45 | 0.18 | 2.84E-01 |
| NM_001277766 | SZT2 | -0.37 | 0.15 | 2.84E-01 |
| NM_001277839 | B3GAT1 | -0.44 | 0.18 | 2.84E-01 |
| NM_204130 | ZNF622 | 0.26 | 0.11 | 2.84E-01 |
| NM_001252027 | CYP46A1 | 0.39 | 0.16 | 2.93E-01 |
| NM_001257316 | LGI2 | -0.35 | 0.14 | 2.93E-01 |
| NM_001277368 | HIST1H110 | 0.40 | 0.16 | 2.93E-01 |
| NM_001030656 | GLT1D1 | 0.24 | 0.10 | 2.94E-01 |
| NM_001252063 | MCF2L | -0.32 | 0.13 | 2.94E-01 |
| NM_001177517 | MOSPD2 | -0.24 | 0.10 | 2.99E-01 |
| NM_001277905 | MINOS1 | 0.38 | 0.16 | 2.99E-01 |
| NM_204845 | PHOSPHO1 | -0.42 | 0.17 | 2.99E-01 |
| NM_001048078 | IL13RA2 | 0.30 | 0.13 | 3.02E-01 |
| NM_001079481 | ACTC1 | 0.31 | 0.13 | 3.02E-01 |
| NM_001199657 | INO80 | -0.26 | 0.11 | 3.02E-01 |
| NM_001277672 | RAB32 | -0.33 | 0.13 | 3.02E-01 |
| NM_001278042 | MRPL20 | 0.42 | 0.17 | 3.02E-01 |
| NM_001302118 | CKS2 | 0.41 | 0.17 | 3.02E-01 |
| NM_204858 | IFNAR2 | -0.34 | 0.14 | 3.02E-01 |
| NM_205067 | HSPE1 | 0.35 | 0.15 | 3.02E-01 |
| NM_001277857 | SPC25 | 0.37 | 0.15 | 3.05E-01 |
| NM_001030711 | RFFL | -0.29 | 0.12 | 3.07E-01 |
| NM_001030913 | AHCYL1 | -0.32 | 0.13 | 3.07E-01 |
| NM_204861 | ANPEP | -0.19 | 0.08 | 3.07E-01 |
| NM_001030940 | SGK3 | 0.38 | 0.16 | 3.09E-01 |
| NM_001277665 | ANGPTL1 | 0.37 | 0.15 | 3.10E-01 |
| NM_204114 | DIO2 | -0.42 | 0.18 | 3.10E-01 |
| NM_204925 | NPPB | 0.40 | 0.17 | 3.10E-01 |
| NM_205304 | TF | 0.37 | 0.16 | 3.10E-01 |
| NM_001007822 | BIRC2 | -0.36 | 0.15 | 3.11E-01 |
| NM_001030641 | TMC7 | -0.39 | 0.16 | 3.13E-01 |
| NM_001271150 | VPS16 | -0.22 | 0.09 | 3.13E-01 |
| NM_001305107 | AGTPBP1 | -0.31 | 0.13 | 3.13E-01 |
| NM_001031251 | PLEKHA3 | -0.37 | 0.15 | 3.14E-01 |
| NM_001031610 | PSIP1 | 0.41 | 0.17 | 3.14E-01 |
| NM_001163245 | GPX7 | 0.29 | 0.12 | 3.14E-01 |
| NM_001004768 | MCAM | 0.34 | 0.15 | 3.17E-01 |
| NM_001012789 | HAGH | -0.33 | 0.14 | 3.17E-01 |
| NM_001012916 | HSPD1 | 0.25 | 0.11 | 3.17E-01 |
| NM_204126 | SREBF1 | -0.38 | 0.16 | 3.19E-01 |
| NM_001030852 | MAFB | -0.27 | 0.11 | 3.19E-01 |
| NM_001030558 | TLR21 | -0.30 | 0.13 | 3.20E-01 |
| NM_001030885 | BNIP3L | -0.38 | 0.16 | 3.20E-01 |
| NM_001030985 | HNRNPA2B1 | 0.38 | 0.16 | 3.20E-01 |
| NM_001080866 | RHOT2 | -0.32 | 0.13 | 3.20E-01 |
| NM_001114638 | FN3KRP | -0.24 | 0.10 | 3.20E-01 |
| NM_001198712 | FN1 | -0.27 | 0.11 | 3.20E-01 |
| NM_001277361 | PCGF5 | 0.37 | 0.16 | 3.20E-01 |
| NM_001278048 | FBP1 | -0.42 | 0.18 | 3.20E-01 |
| NM_001281807 | GATSL2 | 0.29 | 0.12 | 3.20E-01 |
| NM_204417 | PTPRC | -0.36 | 0.15 | 3.20E-01 |
| NM_205456 | TNC | 0.40 | 0.17 | 3.20E-01 |
| NM_001012843 | MOV10 | -0.32 | 0.14 | 3.20E-01 |
| NM_204946 | TCF15 | 0.32 | 0.14 | 3.24E-01 |
| NM_001030956 | FBXO32 | -0.36 | 0.16 | 3.25E-01 |
| NM_001277877 | NINJ1 | -0.25 | 0.11 | 3.25E-01 |
| NM_001302137 | NDUFA2 | 0.36 | 0.15 | 3.25E-01 |
| NM_204717 | ECE1 | -0.25 | 0.11 | 3.25E-01 |
| NM_205520 | ATP1B1 | 0.28 | 0.12 | 3.25E-01 |
| NM_213580 | RP5-966M1.6 | 0.42 | 0.18 | 3.25E-01 |
| NM_001012913 | SLC40A1 | -0.32 | 0.14 | 3.28E-01 |
| NM_001190925 | NUMA1 | -0.25 | 0.11 | 3.28E-01 |
| NM_001199502 | DEPDC6 | 0.28 | 0.12 | 3.28E-01 |
| NM_204757 | MAFF | 0.30 | 0.13 | 3.28E-01 |
| NM_001252311 | MRPS18C | 0.38 | 0.16 | 3.28E-01 |
| NM_001007888 | TCEB1 | 0.23 | 0.10 | 3.30E-01 |
| NM_001286163 | TIMM44 | 0.28 | 0.12 | 3.31E-01 |
| NM_001031105 | CYTH1 | -0.20 | 0.09 | 3.32E-01 |
| NM_204504 | BHLHE23 | -0.42 | 0.18 | 3.34E-01 |
| NM_001004389 | CCT8 | 0.29 | 0.13 | 3.34E-01 |
| NM_001031437 | KIAA0020 | 0.27 | 0.12 | 3.34E-01 |
| NM_001080210 | FBXO18 | -0.41 | 0.18 | 3.34E-01 |
| NM_204739 | TWIST1 | 0.32 | 0.14 | 3.35E-01 |
| NM_001012928 | DEPDC1 | 0.25 | 0.11 | 3.36E-01 |
| NM_205125 | DKK3 | 0.29 | 0.13 | 3.39E-01 |
| NM_001277873 | HNMT | 0.36 | 0.16 | 3.42E-01 |
| NM_001001607 | GAL12 | 0.39 | 0.17 | 3.45E-01 |
| NM_001012701 | SNRNP200 | -0.21 | 0.09 | 3.45E-01 |
| NM_204734 | POLR2F | 0.34 | 0.15 | 3.45E-01 |
| NM_001001291 | ADCYAP1 | 0.33 | 0.15 | 3.46E-01 |
| NM_001278007 | SSBP1 | 0.37 | 0.16 | 3.47E-01 |
| NM_001031187 | PDHX | 0.26 | 0.11 | 3.50E-01 |
| NM_001007977 | ALDOB | -0.36 | 0.16 | 3.52E-01 |
| NM_001085439 | SHFM1 | 0.40 | 0.18 | 3.52E-01 |
| NM_001001530 | LEPREL1 | 0.39 | 0.17 | 3.52E-01 |
| NM_001006203 | EDF1 | 0.31 | 0.14 | 3.52E-01 |
| NM_001012954 | PAN2 | -0.31 | 0.14 | 3.52E-01 |
| NM_001030834 | PPME1 | -0.19 | 0.08 | 3.52E-01 |
| NM_001031491 | CTSA | -0.21 | 0.09 | 3.52E-01 |
| NM_001038496 | EIF5B | 0.35 | 0.16 | 3.52E-01 |
| NM_001083362 | CXCR7 | -0.27 | 0.12 | 3.52E-01 |
| NM_001113454 | TMEM167A | 0.26 | 0.11 | 3.52E-01 |
| NM_001145200 | C12orf75 | 0.29 | 0.13 | 3.52E-01 |
| NM_001277503 | CINP | 0.38 | 0.17 | 3.52E-01 |
| NM_001277583 | GPBP1L1 | -0.22 | 0.10 | 3.52E-01 |
| NM_001302181 | LYRM5 | 0.39 | 0.17 | 3.52E-01 |
| NM_001305041 | HSF3 | -0.25 | 0.11 | 3.52E-01 |
| NM_001305234 | ENTPD6 | 0.26 | 0.11 | 3.52E-01 |
| NM_204289 | HSP90B1 | 0.26 | 0.12 | 3.52E-01 |
| NM_204597 | EIF2S2 | 0.24 | 0.11 | 3.52E-01 |
| NM_204800 | SPRY2 | 0.26 | 0.12 | 3.52E-01 |
| NM_205196 | IRF2 | -0.37 | 0.17 | 3.52E-01 |
| NM_001109785 | HSP90AA1 | 0.31 | 0.14 | 3.53E-01 |
| NM_001141926 | BEAN1 | -0.36 | 0.16 | 3.53E-01 |
| NM_001277379 | HABP2 | 0.41 | 0.18 | 3.53E-01 |
| NM_205298 | ETS1 | 0.29 | 0.13 | 3.53E-01 |
| NM_205351 | ANXA2 | 0.28 | 0.13 | 3.53E-01 |
| NM_001031414 | LYSMD3 | -0.29 | 0.13 | 3.56E-01 |
| NM_001199390 | MRPS5 | 0.35 | 0.15 | 3.56E-01 |
| NM_001277724 | PRRX1 | 0.40 | 0.18 | 3.57E-01 |
| NM_001012771 | EIF3J | 0.30 | 0.14 | 3.58E-01 |
| NM_001277560 | C8H1ORF52 | 0.34 | 0.15 | 3.62E-01 |
| NM_001177739 | ATOH8 | 0.31 | 0.14 | 3.62E-01 |
| NM_204357 | ATXN3 | -0.39 | 0.17 | 3.62E-01 |
| NM_001006284 | POLD3 | -0.30 | 0.14 | 3.63E-01 |
| NM_001039264 | CDK2AP1 | 0.27 | 0.12 | 3.63E-01 |
| NM_001198657 | COX6C | 0.32 | 0.15 | 3.63E-01 |
| NM_001198752 | CCR7 | -0.40 | 0.18 | 3.63E-01 |
| NM_001277482 | ANAPC13 | 0.35 | 0.16 | 3.63E-01 |
| NM_001302188 | SEC61G | 0.35 | 0.16 | 3.63E-01 |
| NM_001276300 | ZBTB17 | -0.29 | 0.13 | 3.66E-01 |
| NM_001030919 | C26H6ORF106 | -0.23 | 0.10 | 3.68E-01 |
| NM_001031552 | ALKBH1 | -0.31 | 0.14 | 3.68E-01 |
| NM_001079762 | LOC772071 | -0.25 | 0.11 | 3.68E-01 |
| NM_001195401 | BAMBI | 0.25 | 0.11 | 3.68E-01 |
| NM_001199595 | PHLDA2 | 0.35 | 0.16 | 3.68E-01 |
| NM_204811 | PSAP | -0.29 | 0.13 | 3.68E-01 |
| NM_001004371 | CDH11 | 0.27 | 0.12 | 3.70E-01 |
| NM_001271612 | C26H6ORF125 | 0.33 | 0.15 | 3.70E-01 |
| NM_001012800 | GTF2H4 | -0.25 | 0.11 | 3.75E-01 |
| NM_001030794 | SETD4 | -0.35 | 0.16 | 3.75E-01 |
| NM_001257289 | SERPINF1 | 0.34 | 0.16 | 3.75E-01 |
| NM_001290554 | SLC4A1 | -0.29 | 0.13 | 3.75E-01 |
| NM_001006459 | GRK4 | 0.28 | 0.13 | 3.79E-01 |
| NM_001008452 | RASSF5 | -0.27 | 0.13 | 3.79E-01 |
| NM_001198600 | LRRC17 | 0.27 | 0.12 | 3.79E-01 |
| NM_204859 | IFNAR1 | -0.38 | 0.17 | 3.80E-01 |
| NM_001271936 | ISCA1 | -0.20 | 0.09 | 3.82E-01 |
| NM_204148 | S100A6 | 0.39 | 0.18 | 3.82E-01 |
| NM_204807 | EPAS1 | -0.39 | 0.18 | 3.82E-01 |
| NM_205368 | EEF2 | -0.18 | 0.08 | 3.82E-01 |
| NM_001277421 | CPQ | 0.35 | 0.16 | 3.83E-01 |
| NM_205525 | APOA1 | 0.38 | 0.18 | 3.83E-01 |
| NM_001006413 | LMBRD1 | 0.22 | 0.10 | 3.91E-01 |
| NM_204274 | CTGF | 0.36 | 0.17 | 3.91E-01 |
| NM_001277726 | PSMB5 | -0.28 | 0.13 | 3.96E-01 |
| NM_001287196 | NPRL2 | -0.32 | 0.15 | 3.96E-01 |
| NM_001024590 | IL1RL1 | 0.33 | 0.15 | 3.98E-01 |
| NM_001044664 | VCP | -0.19 | 0.09 | 3.98E-01 |
| NM_001079733 | ATE1 | -0.34 | 0.16 | 3.98E-01 |
| NM_001201447 | GTPBP1 | -0.17 | 0.08 | 3.98E-01 |
| NM_001252088 | C15H12ORF65 | 0.36 | 0.17 | 3.98E-01 |
| NM_001293074 | APTX | 0.31 | 0.14 | 3.98E-01 |
| NM_001302190 | SMPX | 0.31 | 0.15 | 3.98E-01 |
| NM_204971 | CTSK | 0.27 | 0.13 | 3.98E-01 |
| NM_001030889 | FABP3 | 0.32 | 0.15 | 3.99E-01 |
| NM_001277461 | TM6SF1 | 0.31 | 0.14 | 3.99E-01 |
| NM_204948 | LFNG | 0.24 | 0.11 | 3.99E-01 |
| NM_205445 | TUBB1 | -0.34 | 0.16 | 3.99E-01 |
| NM_001031504 | RAB8A | 0.31 | 0.14 | 4.01E-01 |
| NM_001162400 | ATM | -0.23 | 0.11 | 4.01E-01 |
| NM_001006300 | RER1 | 0.24 | 0.11 | 4.01E-01 |
| NM_001277853 | GPX1 | -0.21 | 0.10 | 4.02E-01 |
| NM_001006510 | ZC3H15 | 0.30 | 0.14 | 4.02E-01 |
| NM_205449 | TNNT2 | 0.36 | 0.17 | 4.03E-01 |
| NM_001012592 | HNRNPH3 | 0.28 | 0.13 | 4.04E-01 |
| NM_001171644 | SPCS1 | 0.35 | 0.17 | 4.04E-01 |
| NM_204119 | EDNRA | -0.22 | 0.10 | 4.04E-01 |
| NM_001097534 | ATP5I | 0.34 | 0.16 | 4.04E-01 |
| NM_001277862 | LSM3 | 0.35 | 0.16 | 4.04E-01 |
| NM_001305459 | ENTPD1 | -0.35 | 0.17 | 4.04E-01 |
| NM_204234 | GNA11 | 0.27 | 0.13 | 4.04E-01 |
| NM_001030749 | ELK3 | 0.21 | 0.10 | 4.05E-01 |
| NM_001030646 | NRF1 | -0.38 | 0.18 | 4.06E-01 |
| NM_204238 | DAB1 | -0.38 | 0.18 | 4.06E-01 |
| NM_001006554 | PDCD10 | 0.24 | 0.12 | 4.09E-01 |
| NM_001030624 | LOC416354 | -0.20 | 0.09 | 4.09E-01 |
| NM_001031061 | SPAST | -0.20 | 0.09 | 4.09E-01 |
| NM_001199592 | GTF2H1 | -0.22 | 0.10 | 4.09E-01 |
| NM_001257265 | PLXNB1 | -0.37 | 0.17 | 4.09E-01 |
| NM_001302092 | FAM136A | 0.36 | 0.17 | 4.09E-01 |
| NM_204511 | ST6GALNAC4 | 0.34 | 0.16 | 4.09E-01 |
| NM_205509 | FGFR3 | -0.25 | 0.12 | 4.09E-01 |
| NM_001159754 | TBL1X | -0.18 | 0.09 | 4.09E-01 |
| NM_001031107 | TMEM104 | -0.30 | 0.14 | 4.10E-01 |
| NM_205049 | RREB1 | -0.37 | 0.18 | 4.11E-01 |
| NM_001293174 | UBB | 0.31 | 0.15 | 4.11E-01 |
| NM_001007830 | SLC25A46 | -0.27 | 0.13 | 4.12E-01 |
| NM_001007847 | YPEL2 | -0.30 | 0.14 | 4.12E-01 |
| NM_001012779 | ADAT1 | -0.24 | 0.11 | 4.12E-01 |
| NM_001012871 | ABHD12 | -0.26 | 0.13 | 4.12E-01 |
| NM_001031029 | KDM5B | -0.21 | 0.10 | 4.12E-01 |
| NM_001031356 | PLEKHO1 | -0.26 | 0.12 | 4.12E-01 |
| NM_001031367 | DCTN1 | -0.21 | 0.10 | 4.12E-01 |
| NM_001037836 | LY75 | -0.38 | 0.18 | 4.12E-01 |
| NM_001039266 | PITPNB | 0.23 | 0.11 | 4.12E-01 |
| NM_001045829 | SURF2 | 0.28 | 0.14 | 4.12E-01 |
| NM_001199449 | PUS10 | -0.24 | 0.11 | 4.12E-01 |
| NM_001257313 | MND1 | 0.32 | 0.15 | 4.12E-01 |
| NM_001277699 | ANGPTL2 | 0.29 | 0.14 | 4.12E-01 |
| NM_001277755 | RPL31 | 0.32 | 0.15 | 4.12E-01 |
| NM_204239 | ERAL1 | -0.32 | 0.15 | 4.12E-01 |
| NM_204782 | NRP1 | -0.37 | 0.18 | 4.12E-01 |
| NM_205239 | CCNB3 | 0.25 | 0.12 | 4.12E-01 |
| NM_205377 | NCS1 | 0.30 | 0.14 | 4.12E-01 |
| NM_205426 | LHX9 | -0.37 | 0.18 | 4.12E-01 |
| NM_001006348 | MTERF3 | 0.25 | 0.12 | 4.14E-01 |
| NM_001030721 | RPS6KB1 | -0.32 | 0.16 | 4.14E-01 |
| NM_001160071 | UCK2 | 0.22 | 0.11 | 4.14E-01 |
| NM_001171677 | RPL29 | 0.29 | 0.14 | 4.14E-01 |
| NM_001199702 | SNRK | -0.33 | 0.16 | 4.14E-01 |
| NM_001006359 | IGF2BP3 | -0.20 | 0.10 | 4.16E-01 |
| NM_001006586 | AP3S1 | 0.24 | 0.12 | 4.16E-01 |
| NM_001012606 | UTP15 | 0.19 | 0.09 | 4.16E-01 |
| NM_001017413 | PTX3 | 0.27 | 0.13 | 4.16E-01 |
| NM_001024694 | RLBP1 | 0.37 | 0.18 | 4.16E-01 |
| NM_001024828 | DNMT3B | -0.21 | 0.10 | 4.16E-01 |
| NM_001031075 | RWDD1 | 0.31 | 0.15 | 4.16E-01 |
| NM_001031213 | FBXO34 | -0.18 | 0.09 | 4.16E-01 |
| NM_001277711 | TCF21 | 0.24 | 0.12 | 4.16E-01 |
| NM_001305093 | RBM38 | -0.17 | 0.08 | 4.16E-01 |
| NM_204105 | SDK1 | -0.26 | 0.13 | 4.16E-01 |
| NM_204576 | DBI | 0.32 | 0.16 | 4.16E-01 |
| NM_206978 | ZCCHC17 | 0.25 | 0.12 | 4.16E-01 |
| NM_001044693 | XKR8 | 0.27 | 0.13 | 4.20E-01 |
| NM_001190477 | LPHN2 | -0.23 | 0.11 | 4.21E-01 |
| NM_001031026 | TIMM17A | 0.25 | 0.12 | 4.22E-01 |
| NM_204837 | HSD17B1 | -0.37 | 0.18 | 4.22E-01 |
| NM_205314 | CDK1 | 0.28 | 0.14 | 4.22E-01 |
| NM_001006286 | CHMP4B | 0.24 | 0.12 | 4.23E-01 |
| NM_001007884 | CCDC43 | 0.20 | 0.10 | 4.23E-01 |
| NM_001030718 | FAM222B | -0.19 | 0.09 | 4.23E-01 |
| NM_001030896 | RRAGC | -0.18 | 0.09 | 4.23E-01 |
| NM_001030964 | WDR48 | -0.21 | 0.10 | 4.23E-01 |
| NM_001031167 | NELFA | -0.19 | 0.09 | 4.23E-01 |
| NM_001031211 | BAG5 | -0.20 | 0.10 | 4.23E-01 |
| NM_001031242 | NEMP2 | -0.21 | 0.10 | 4.23E-01 |
| NM_001031540 | UBE2K | 0.31 | 0.15 | 4.23E-01 |
| NM_001037837 | TNFAIP6 | 0.32 | 0.16 | 4.23E-01 |
| NM_001080869 | VAPA | -0.29 | 0.14 | 4.23E-01 |
| NM_001097536 | SRP9 | 0.29 | 0.14 | 4.23E-01 |
| NM_001190802 | AC005943.2 | 0.30 | 0.15 | 4.23E-01 |
| NM_001193669 | SSB | 0.27 | 0.13 | 4.23E-01 |
| NM_001198782 | COX7A2 | 0.31 | 0.15 | 4.23E-01 |
| NM_001199230 | NDUFAF2 | 0.31 | 0.15 | 4.23E-01 |
| NM_001199458 | SYPL1 | 0.37 | 0.18 | 4.23E-01 |
| NM_001199525 | DNAJC9 | 0.31 | 0.15 | 4.23E-01 |
| NM_001277667 | PRPF38A | 0.22 | 0.11 | 4.23E-01 |
| NM_001277733 | CENPW | 0.29 | 0.14 | 4.23E-01 |
| NM_001278020 | SMIM8 | 0.34 | 0.17 | 4.23E-01 |
| NM_001282303 | CRIP1 | 0.27 | 0.13 | 4.23E-01 |
| NM_204110 | PDIA3 | 0.21 | 0.10 | 4.23E-01 |
| NM_205050 | CNP | -0.29 | 0.15 | 4.23E-01 |
| NM_001257371 | NME5 | 0.37 | 0.18 | 4.23E-01 |
| NM_001013394 | PPM1M | 0.25 | 0.13 | 4.27E-01 |
| NM_001039291 | ACP1 | 0.27 | 0.14 | 4.27E-01 |
| NM_204297 | HIF1A | -0.17 | 0.09 | 4.27E-01 |
| NM_001004402 | WDR1 | 0.25 | 0.13 | 4.27E-01 |
| NM_001006437 | NKAP | 0.26 | 0.13 | 4.27E-01 |
| NM_001012559 | Sep-07 | 0.20 | 0.10 | 4.27E-01 |
| NM_001142848 | DRAXIN | 0.32 | 0.16 | 4.27E-01 |
| NM_001199170 | NDUFV3 | 0.30 | 0.15 | 4.27E-01 |
| NM_001199572 | CDC25A | 0.23 | 0.12 | 4.27E-01 |
| NM_204166 | LAMB2 | -0.21 | 0.11 | 4.27E-01 |
| NM_001001189 | CFDP1 | 0.30 | 0.15 | 4.28E-01 |
| NM_001030871 | CDC2L1 | 0.28 | 0.14 | 4.28E-01 |
| NM_001291781 | RASA4 | 0.31 | 0.16 | 4.28E-01 |
| NM_001302169 | COX17 | 0.33 | 0.17 | 4.28E-01 |
| NM_001006145 | IK | 0.27 | 0.14 | 4.30E-01 |
| NM_205422 | LCN8 | 0.35 | 0.18 | 4.30E-01 |
| NM_001006301 | MIB2 | -0.29 | 0.15 | 4.33E-01 |
| NM_001037826 | NR3C1 | -0.36 | 0.18 | 4.33E-01 |
| NM_205341 | MYL12A | 0.25 | 0.13 | 4.33E-01 |
| NM_205510 | FGFR1 | -0.16 | 0.08 | 4.34E-01 |
| NM_001006262 | ASB9 | -0.30 | 0.15 | 4.34E-01 |
| NM_001040228 | INTS8 | -0.21 | 0.10 | 4.34E-01 |
| NM_001001897 | TCP11L2 | -0.36 | 0.18 | 4.34E-01 |
| NM_001112809 | HSBP1 | 0.28 | 0.14 | 4.34E-01 |
| NM_001199634 | CHID1 | 0.30 | 0.15 | 4.34E-01 |
| NM_001012841 | KPNA6 | -0.19 | 0.09 | 4.35E-01 |
| NM_001305216 | FZD5 | -0.33 | 0.17 | 4.40E-01 |
| NM_204960 | PTCH1 | -0.33 | 0.17 | 4.41E-01 |
| NM_001012576 | HSPA4L | 0.22 | 0.11 | 4.42E-01 |
| NM_001111346 | LCT | 0.25 | 0.13 | 4.42E-01 |
| NM_204236 | OPTN | 0.17 | 0.08 | 4.42E-01 |
| NM_001008479 | SUB1 | 0.18 | 0.09 | 4.42E-01 |
| NM_001031011 | SLMO1 | 0.31 | 0.16 | 4.44E-01 |
| NM_001044663 | CENPK | 0.24 | 0.12 | 4.44E-01 |
| NM_001080717 | SUMO2 | 0.27 | 0.14 | 4.44E-01 |
| NM_204330 | FKBP1A | 0.25 | 0.13 | 4.44E-01 |
| NM_001006154 | C13H5ORF15 | 0.22 | 0.11 | 4.47E-01 |
| NM_001031585 | ZC3H3 | -0.25 | 0.13 | 4.47E-01 |
| NM_206905 | LGALS1 | 0.31 | 0.16 | 4.47E-01 |
| NM_001006296 | VAPB | 0.23 | 0.12 | 4.49E-01 |
| NM_001079489 | MAFG | 0.32 | 0.16 | 4.49E-01 |
| NM_001257348 | LOC427025 | -0.20 | 0.10 | 4.49E-01 |
| NM_204232 | HIRA | -0.31 | 0.16 | 4.49E-01 |
| NM_205250 | SNRPE | 0.29 | 0.15 | 4.49E-01 |
| NM_001031057 | FAM98A | 0.19 | 0.10 | 4.51E-01 |
| NM_001077586 | CHD7 | -0.23 | 0.12 | 4.51E-01 |
| NM_001277169 | TSR3 | 0.24 | 0.12 | 4.51E-01 |
| NM_001277549 | MRPS14 | 0.28 | 0.14 | 4.51E-01 |
| NM_001198556 | NFRKB | -0.18 | 0.09 | 4.52E-01 |
| NM_001006557 | SELT | 0.20 | 0.11 | 4.53E-01 |
| NM_001031006 | CCDC127 | -0.34 | 0.17 | 4.53E-01 |
| NM_001031195 | RP5-1021I20.4 | -0.27 | 0.14 | 4.53E-01 |
| NM_001031582 | PSMF1 | -0.23 | 0.12 | 4.53E-01 |
| NM_001039263 | RSL1D1 | 0.21 | 0.11 | 4.53E-01 |
| NM_001184713 | TFDP2 | -0.16 | 0.08 | 4.53E-01 |
| NM_001199617 | TOMM7 | 0.31 | 0.16 | 4.53E-01 |
| NM_001252144 | NHP2 | 0.31 | 0.16 | 4.53E-01 |
| NM_001302173 | S100A1 | 0.27 | 0.14 | 4.53E-01 |
| NM_001007905 | PSMB1 | 0.24 | 0.13 | 4.56E-01 |
| NM_001030610 | KIAA1191 | -0.27 | 0.14 | 4.56E-01 |
| NM_001198884 | OST4 | 0.29 | 0.15 | 4.56E-01 |
| NM_001277727 | MRPL33 | 0.31 | 0.16 | 4.56E-01 |
| NM_204116 | BASP1 | 0.25 | 0.13 | 4.56E-01 |
| NM_204502 | FSTL4 | -0.35 | 0.18 | 4.56E-01 |
| NM_205527 | AGRN | -0.24 | 0.13 | 4.56E-01 |
| NM_001005427 | MEOX2 | 0.32 | 0.17 | 4.56E-01 |
| NM_001006470 | TSSC4 | 0.18 | 0.09 | 4.56E-01 |
| NM_001013397 | MYH1E | 0.33 | 0.17 | 4.56E-01 |
| NM_001025609 | DNAJC18 | 0.19 | 0.10 | 4.56E-01 |
| NM_001030538 | JAK2 | -0.34 | 0.18 | 4.56E-01 |
| NM_001031013 | LDLRAD4 | 0.20 | 0.11 | 4.56E-01 |
| NM_001031094 | UBXN2A | 0.28 | 0.15 | 4.56E-01 |
| NM_001031361 | GSDMA | -0.26 | 0.14 | 4.56E-01 |
| NM_001031523 | NTPCR | 0.27 | 0.14 | 4.56E-01 |
| NM_001038693 | PGM1 | 0.20 | 0.11 | 4.56E-01 |
| NM_001039310 | MXD1 | -0.21 | 0.11 | 4.56E-01 |
| NM_001045833 | STK11 | -0.25 | 0.13 | 4.56E-01 |
| NM_001085359 | DUSP1 | 0.26 | 0.14 | 4.56E-01 |
| NM_001184758 | NDUFA12 | 0.26 | 0.14 | 4.56E-01 |
| NM_001198641 | NUBP1 | 0.20 | 0.10 | 4.56E-01 |
| NM_001199404 | TRIM55 | 0.26 | 0.14 | 4.56E-01 |
| NM_001199647 | PHF21A | -0.15 | 0.08 | 4.56E-01 |
| NM_001277548 | RHOJ | 0.34 | 0.18 | 4.56E-01 |
| NM_001277717 | RPL22L1 | 0.24 | 0.13 | 4.56E-01 |
| NM_001277882 | PTPMT1 | 0.27 | 0.14 | 4.56E-01 |
| NM_001278124 | MPC2 | 0.26 | 0.14 | 4.56E-01 |
| NM_001293107 | ELN | 0.19 | 0.10 | 4.56E-01 |
| NM_001293318 | RAD54L2 | -0.26 | 0.14 | 4.56E-01 |
| NM_204271 | LMO2 | -0.26 | 0.13 | 4.56E-01 |
| NM_204350 | PIK3AP1 | 0.30 | 0.16 | 4.56E-01 |
| NM_204395 | RGS2 | 0.34 | 0.18 | 4.56E-01 |
| NM_204501 | SHISA2 | 0.19 | 0.10 | 4.56E-01 |
| NM_204589 | ID3 | 0.30 | 0.16 | 4.56E-01 |
| NM_204721 | PDGFC | -0.18 | 0.10 | 4.56E-01 |
| NM_204887 | WNT5A | -0.34 | 0.18 | 4.56E-01 |
| NM_205011 | HPGDS | 0.34 | 0.18 | 4.56E-01 |
| NM_205237 | BMP4 | 0.26 | 0.14 | 4.56E-01 |
| NM_001006490 | NUDT14 | -0.17 | 0.09 | 4.57E-01 |
| NM_001008437 | DNAJC3 | 0.19 | 0.10 | 4.57E-01 |
| NM_001252376 | HERC2 | -0.23 | 0.12 | 4.60E-01 |
| NM_001277705 | MYEOV2 | 0.31 | 0.16 | 4.60E-01 |
| NM_001030927 | MAP3K14 | -0.31 | 0.17 | 4.64E-01 |
| NM_001006383 | FAM206A | 0.27 | 0.14 | 4.65E-01 |
| NM_001277567 | LYPLAL1 | 0.27 | 0.15 | 4.65E-01 |
| NM_205145 | SCNN1A | -0.29 | 0.16 | 4.65E-01 |
| NM_205500 | CST3 | -0.31 | 0.16 | 4.65E-01 |
| NM_001199700 | EXD2 | -0.18 | 0.09 | 4.65E-01 |
| NM_001030724 | AATF | 0.23 | 0.12 | 4.66E-01 |
| NM_001079754 | RP11-26J3.4 | -0.17 | 0.09 | 4.66E-01 |
| NM_001277897 | HADH | 0.23 | 0.12 | 4.66E-01 |
| NM_204835 | NUMB | -0.24 | 0.13 | 4.66E-01 |
| NM_204881 | ITPKA | -0.30 | 0.16 | 4.66E-01 |
| NM_001079576 | NVL | -0.18 | 0.10 | 4.67E-01 |
| NM_001114851 | FANCI | -0.28 | 0.15 | 4.67E-01 |
| NM_205100 | CRTAP | 0.22 | 0.12 | 4.67E-01 |
| NM_001010843 | LGALS8 | -0.29 | 0.16 | 4.67E-01 |
| NM_001012567 | TTC27 | 0.29 | 0.16 | 4.67E-01 |
| NM_001006313 | TMEM39B | -0.22 | 0.12 | 4.69E-01 |
| NM_001007477 | LOC396531 | 0.33 | 0.18 | 4.69E-01 |
| NM_001030778 | NAA50 | -0.26 | 0.14 | 4.69E-01 |
| NM_001282210 | GAPVD1 | -0.21 | 0.11 | 4.69E-01 |
| NM_001012851 | DHX30 | -0.19 | 0.10 | 4.70E-01 |
| NM_001013396 | MYH1A | 0.23 | 0.12 | 4.70E-01 |
| NM_204152 | RPL6 | 0.24 | 0.13 | 4.70E-01 |
| NM_001031173 | BET1L | -0.18 | 0.09 | 4.70E-01 |
| NM_001256543 | RTTN | 0.20 | 0.11 | 4.70E-01 |
| NM_001012552 | GATAD2A | -0.30 | 0.16 | 4.71E-01 |
| NM_204129 | MID1 | -0.34 | 0.18 | 4.71E-01 |
| NM_001030799 | MAOA | -0.20 | 0.11 | 4.71E-01 |
| NM_001113453 | PNPLA6 | -0.24 | 0.13 | 4.71E-01 |
| NM_001030677 | MVB12B | -0.19 | 0.11 | 4.74E-01 |
| NM_001006176 | CORO7 | -0.19 | 0.10 | 4.74E-01 |
| NM_001006525 | EAF2 | -0.30 | 0.16 | 4.74E-01 |
| NM_001031530 | LUC7L3 | 0.28 | 0.15 | 4.74E-01 |
| NM_001277133 | CNOT1 | -0.20 | 0.11 | 4.74E-01 |
| NM_001277916 | MRTO4 | 0.28 | 0.15 | 4.74E-01 |
| NM_001007839 | YWHAH | 0.27 | 0.15 | 4.75E-01 |
| NM_001008678 | RAB9A | -0.18 | 0.10 | 4.75E-01 |
| NM_001012878 | NCOA7 | -0.33 | 0.18 | 4.75E-01 |
| NM_001030553 | FEM1B | -0.19 | 0.10 | 4.75E-01 |
| NM_001030876 | NOC2L | 0.19 | 0.10 | 4.75E-01 |
| NM_001030892 | RPA2 | 0.23 | 0.12 | 4.75E-01 |
| NM_001031091 | MYCN | -0.22 | 0.12 | 4.75E-01 |
| NM_001031493 | FAM210B | -0.28 | 0.15 | 4.75E-01 |
| NM_001038652 | CNR1 | -0.21 | 0.11 | 4.75E-01 |
| NM_001199490 | DHCR7 | -0.19 | 0.10 | 4.75E-01 |
| NM_001199703 | CMTM8 | 0.25 | 0.14 | 4.75E-01 |
| NM_001204815 | FPGT | -0.21 | 0.11 | 4.75E-01 |
| NM_001277413 | NECAB1 | 0.33 | 0.18 | 4.75E-01 |
| NM_001277635 | TYW3 | 0.33 | 0.18 | 4.75E-01 |
| NM_001277664 | COX20 | 0.27 | 0.15 | 4.75E-01 |
| NM_001302149 | UQCR10 | 0.29 | 0.16 | 4.75E-01 |
| NM_204221 | FZD7 | -0.28 | 0.15 | 4.75E-01 |
| NM_204486 | TCF3 | -0.27 | 0.15 | 4.75E-01 |
| NM_204498 | LOC395159 | 0.30 | 0.17 | 4.76E-01 |
| NM_001030582 | DHX38 | -0.16 | 0.09 | 4.76E-01 |
| NM_001030585 | APEH | -0.17 | 0.09 | 4.76E-01 |
| NM_001277494 | ST6GALNAC5 | 0.29 | 0.16 | 4.78E-01 |
| NM_001252104 | ALDH4A1 | -0.30 | 0.17 | 4.78E-01 |
| NM_204379 | TNFSF10 | -0.31 | 0.17 | 4.78E-01 |
| NM_001030853 | STK4 | -0.16 | 0.09 | 4.79E-01 |
| NM_001198907 | DPY30 | 0.28 | 0.15 | 4.81E-01 |
| NM_205305 | COL9A3 | 0.31 | 0.17 | 4.81E-01 |
| NM_001006133 | PRKCD | 0.25 | 0.14 | 4.81E-01 |
| NM_001012856 | PHF14 | 0.24 | 0.13 | 4.81E-01 |
| NM_001030999 | PAK1IP1 | 0.24 | 0.13 | 4.81E-01 |
| NM_001031019 | PCMTD1 | -0.28 | 0.15 | 4.81E-01 |
| NM_001031238 | CACUL1 | -0.27 | 0.15 | 4.81E-01 |
| NM_001031280 | EIF2B3 | 0.22 | 0.12 | 4.81E-01 |
| NM_001039301 | DDB2 | 0.26 | 0.14 | 4.81E-01 |
| NM_001199597 | RTKN2 | -0.33 | 0.18 | 4.81E-01 |
| NM_001291811 | A4GALT | -0.24 | 0.13 | 4.81E-01 |
| NM_001308441 | PIGBOS1 | 0.31 | 0.17 | 4.81E-01 |
| NM_204122 | PTPRO | -0.29 | 0.16 | 4.81E-01 |
| NM_204631 | ADCY5 | -0.33 | 0.18 | 4.81E-01 |
| NM_204747 | HDAC3 | -0.28 | 0.16 | 4.81E-01 |
| NM_001044635 | CENPP | 0.21 | 0.12 | 4.82E-01 |
| NM_001137651 | TMEM101 | 0.27 | 0.15 | 4.83E-01 |
| NM_204931 | GJB6 | -0.33 | 0.18 | 4.83E-01 |
| NM_001198655 | EDN2 | 0.33 | 0.18 | 4.85E-01 |
| NM_204136 | EGR1 | 0.33 | 0.18 | 4.86E-01 |
| NM_205159 | MYL3 | 0.26 | 0.14 | 4.86E-01 |
| NM_205295 | HMGB3 | 0.20 | 0.11 | 4.86E-01 |
| NM_001160428 | FADS2 | -0.32 | 0.18 | 4.87E-01 |
| NM_001199497 | CRELD2 | 0.22 | 0.12 | 4.88E-01 |
| NM_001277828 | ZNF330 | 0.19 | 0.11 | 4.90E-01 |
| NM_001079496 | ZNF384 | -0.22 | 0.12 | 4.91E-01 |
| NM_001172781 | HTR1B | -0.32 | 0.18 | 4.91E-01 |
| NM_001006270 | GPALPP1 | 0.24 | 0.14 | 4.92E-01 |
| NM_001030989 | SH3BP5 | -0.14 | 0.08 | 4.92E-01 |
| NM_001031386 | CIR1 | 0.30 | 0.17 | 4.92E-01 |
| NM_001199470 | GAS2 | 0.30 | 0.17 | 4.92E-01 |
| NM_001277821 | COMMD8 | 0.29 | 0.16 | 4.92E-01 |
| NM_001305144 | TMEM237 | -0.25 | 0.14 | 4.92E-01 |
| NM_001305681 | EGFL7 | 0.18 | 0.10 | 4.92E-01 |
| NM_204328 | FOXO1 | -0.21 | 0.11 | 4.92E-01 |
| NM_204354 | DUSP6 | 0.23 | 0.13 | 4.92E-01 |
| NM_204528 | SH3GL3 | -0.24 | 0.14 | 4.92E-01 |
| NM_205003 | HSPA8 | 0.26 | 0.14 | 4.92E-01 |
| NR_105510 | MIR6642 | -0.25 | 0.14 | 4.92E-01 |
| NM_001031295 | LRRC40 | -0.18 | 0.10 | 4.93E-01 |
| NM_001198612 | EMC3 | 0.22 | 0.12 | 4.93E-01 |
| NM_001291406 | ALAD | -0.18 | 0.10 | 4.93E-01 |
| NM_204523 | TLN1 | -0.27 | 0.15 | 4.93E-01 |
| NM_001079505 | DENR | 0.29 | 0.16 | 4.96E-01 |
| NM_001277859 | SELM | 0.20 | 0.11 | 4.96E-01 |
| NM_205267 | NPM1 | 0.25 | 0.14 | 4.96E-01 |
| NM_001006579 | NSA2 | 0.26 | 0.15 | 4.96E-01 |
| NM_001006251 | ING4 | 0.19 | 0.11 | 4.96E-01 |
| NM_001007913 | KCTD2 | -0.14 | 0.08 | 4.96E-01 |
| NM_001031444 | DCAF12 | -0.19 | 0.10 | 4.96E-01 |
| NM_001305091 | MIF | 0.24 | 0.13 | 4.96E-01 |
| NM_204788 | AMT | -0.20 | 0.11 | 4.96E-01 |
| NM_001002856 | IFI27L2 | 0.30 | 0.17 | 4.99E-01 |
| NM_001006461 | LETM1 | 0.25 | 0.14 | 4.99E-01 |
| NM_001009928 | NOCT | -0.24 | 0.13 | 4.99E-01 |
| NM_001031116 | KLHL13 | -0.16 | 0.09 | 4.99E-01 |
| NM_001032362 | DRG1 | 0.18 | 0.10 | 4.99E-01 |
| NM_001252160 | TRAPPC6B | 0.25 | 0.14 | 4.99E-01 |
| NM_204358 | BMP2 | 0.25 | 0.14 | 4.99E-01 |
| NM_204658 | PRKDC | -0.26 | 0.15 | 4.99E-01 |
| NM_001199504 | PHYHIPL | -0.27 | 0.15 | 5.03E-01 |
| NM_001270967 | TOM1L2 | -0.17 | 0.10 | 5.03E-01 |
| NM_001079743 | USP48 | -0.24 | 0.13 | 5.04E-01 |
| NM_001277558 | MIA3 | -0.17 | 0.10 | 5.05E-01 |
| NM_001005346 | TSKU | -0.27 | 0.15 | 5.08E-01 |
| NM_001006174 | RMI2 | -0.29 | 0.17 | 5.08E-01 |
| NM_001006382 | FH | 0.19 | 0.11 | 5.08E-01 |
| NM_001007844 | WDR45B | -0.16 | 0.09 | 5.08E-01 |
| NM_001012544 | PDHA1 | 0.18 | 0.10 | 5.08E-01 |
| NM_001012563 | CCT5 | 0.19 | 0.11 | 5.08E-01 |
| NM_001012827 | PDS5B | 0.18 | 0.11 | 5.08E-01 |
| NM_001012842 | BSDC1 | -0.19 | 0.11 | 5.08E-01 |
| NM_001012927 | MYSM1 | -0.24 | 0.14 | 5.08E-01 |
| NM_001012941 | APCDD1 | 0.21 | 0.12 | 5.08E-01 |
| NM_001030690 | RP11-101E3.5 | -0.18 | 0.10 | 5.08E-01 |
| NM_001030902 | SMAP2 | -0.22 | 0.13 | 5.08E-01 |
| NM_001030914 | RBBP5 | -0.18 | 0.10 | 5.08E-01 |
| NM_001031369 | TBL1XR1 | -0.26 | 0.15 | 5.08E-01 |
| NM_001031411 | UBE2R2 | -0.26 | 0.15 | 5.08E-01 |
| NM_001044661 | ERBB2 | -0.22 | 0.13 | 5.08E-01 |
| NM_001099357 | TAP2 | 0.28 | 0.16 | 5.08E-01 |
| NM_001164350 | MZT1 | 0.24 | 0.14 | 5.08E-01 |
| NM_001199407 | TCP11L1 | -0.22 | 0.12 | 5.08E-01 |
| NM_001199648 | SGOL1 | 0.17 | 0.10 | 5.08E-01 |
| NM_001204217 | AKAP2 | 0.21 | 0.12 | 5.08E-01 |
| NM_001277112 | MRPL46 | 0.27 | 0.16 | 5.08E-01 |
| NM_001277362 | SNRPF | 0.29 | 0.17 | 5.08E-01 |
| NM_001277614 | DEXI | 0.22 | 0.13 | 5.08E-01 |
| NM_001277677 | ATP6V1G1 | 0.24 | 0.14 | 5.08E-01 |
| NM_001277829 | DCTN6 | 0.24 | 0.14 | 5.08E-01 |
| NM_001277896 | NDUFB2 | 0.27 | 0.16 | 5.08E-01 |
| NM_001278012 | RPL38 | 0.27 | 0.16 | 5.08E-01 |
| NM_001278015 | PFDN4 | 0.24 | 0.14 | 5.08E-01 |
| NM_001278093 | ZNF341 | -0.25 | 0.14 | 5.08E-01 |
| NM_204115 | TNFRSF10B | -0.30 | 0.17 | 5.08E-01 |
| NM_204227 | CDH5 | -0.23 | 0.13 | 5.08E-01 |
| NM_204590 | ID1 | 0.27 | 0.16 | 5.08E-01 |
| NM_204972 | PRELID1 | 0.21 | 0.12 | 5.08E-01 |
| NM_205163 | MYO1A | -0.27 | 0.16 | 5.08E-01 |
| NM_205230 | SMC2 | 0.21 | 0.12 | 5.08E-01 |
| NM_001277566 | GNPTG | 0.20 | 0.11 | 5.08E-01 |
| NM_001006353 | CCDC12 | 0.25 | 0.15 | 5.08E-01 |
| NM_001006249 | PHC1 | -0.17 | 0.10 | 5.09E-01 |
| NM_001006250 | FKBP4 | 0.18 | 0.10 | 5.10E-01 |
| NM_001277673 | UQCRH | 0.25 | 0.15 | 5.10E-01 |
| NM_001302176 | ABRACL | 0.24 | 0.14 | 5.10E-01 |
| NM_001031469 | SBNO1 | -0.21 | 0.12 | 5.11E-01 |
| NM_001282294 | INPP5K | -0.20 | 0.12 | 5.11E-01 |
| NM_001031152 | ADH5 | 0.18 | 0.11 | 5.11E-01 |
| NM_204564 | SSBP3 | -0.20 | 0.12 | 5.11E-01 |
| NM_205023 | SPI1 | 0.27 | 0.16 | 5.11E-01 |
| NM_001012935 | RNF166 | -0.18 | 0.11 | 5.12E-01 |
| NM_001005802 | SEC11A | 0.24 | 0.14 | 5.12E-01 |
| NM_001006136 | ACAD9 | 0.17 | 0.10 | 5.12E-01 |
| NM_001199575 | BTBD10 | 0.15 | 0.09 | 5.12E-01 |
| NM_001252154 | EXOSC7 | 0.25 | 0.14 | 5.14E-01 |
| NM_001030812 | PDCL3 | 0.22 | 0.13 | 5.14E-01 |
| NM_001030625 | HAUS1 | 0.26 | 0.15 | 5.15E-01 |
| NM_001079715 | MAN2C1 | -0.30 | 0.18 | 5.16E-01 |
| NM_001199250 | RBX1 | 0.24 | 0.14 | 5.17E-01 |
| NM_001256123 | MPDZ | -0.21 | 0.13 | 5.17E-01 |
| NM_001204761 | NME1 | 0.29 | 0.17 | 5.17E-01 |
| NM_001278078 | N6AMT1 | 0.21 | 0.12 | 5.17E-01 |
| NM_001199503 | PTDSS2 | -0.31 | 0.18 | 5.17E-01 |
| NM_001031501 | EFTUD2 | -0.13 | 0.08 | 5.18E-01 |
| NM_001302136 | C14orf2 | 0.29 | 0.17 | 5.18E-01 |
| NM_001007838 | SNRPD3 | 0.23 | 0.14 | 5.19E-01 |
| NM_001031206 | TMED8 | -0.28 | 0.17 | 5.19E-01 |
| NM_001012826 | FNDC3A | -0.24 | 0.14 | 5.19E-01 |
| NM_001012953 | MTA1 | -0.15 | 0.09 | 5.19E-01 |
| NM_001201452 | RAC2 | -0.30 | 0.18 | 5.19E-01 |
| NM_205121 | CHST3 | -0.23 | 0.14 | 5.19E-01 |
| NM_001006192 | XBP1 | 0.21 | 0.12 | 5.20E-01 |
| NM_001199530 | DNAJC12 | -0.29 | 0.17 | 5.20E-01 |
| NM_001097538 | MT3 | 0.30 | 0.18 | 5.21E-01 |
| NM_001278070 | ATG3 | 0.18 | 0.11 | 5.21E-01 |
| NM_001001764 | PDLIM3 | 0.29 | 0.17 | 5.23E-01 |
| NM_001006451 | DCK | 0.20 | 0.12 | 5.23E-01 |
| NM_001008729 | AZIN1 | 0.16 | 0.10 | 5.23E-01 |
| NM_001256157 | ARID3B | -0.30 | 0.18 | 5.23E-01 |
| NM_001199522 | GCHFR | -0.30 | 0.18 | 5.25E-01 |
| NM_001277460 | RPS27L | 0.27 | 0.16 | 5.25E-01 |
| NM_204484 | SOX4 | -0.23 | 0.14 | 5.25E-01 |
| NM_205148 | BMP5 | -0.30 | 0.18 | 5.25E-01 |
| NM_001006272 | CAB39L | -0.26 | 0.16 | 5.26E-01 |
| NM_001006575 | PHAX | 0.18 | 0.11 | 5.26E-01 |
| NM_001012580 | PDS5A | -0.27 | 0.16 | 5.26E-01 |
| NM_001030636 | TOP3A | -0.20 | 0.12 | 5.26E-01 |
| NM_001031108 | GGA3 | -0.18 | 0.11 | 5.26E-01 |
| NM_001136182 | HSBP1L1 | 0.30 | 0.18 | 5.26E-01 |
| NM_001199825 | ALG11 | 0.20 | 0.12 | 5.26E-01 |
| NM_001257346 | RRBP1 | 0.22 | 0.13 | 5.26E-01 |
| NM_204431 | CCDC80 | 0.18 | 0.11 | 5.26E-01 |
| NM_204611 | CENPH | 0.25 | 0.15 | 5.26E-01 |
| NM_204970 | IGF2R | -0.19 | 0.11 | 5.26E-01 |
| NM_204998 | RP13-512J5.1 | 0.19 | 0.11 | 5.26E-01 |
| NM_205012 | HESX1 | 0.28 | 0.17 | 5.26E-01 |
| NM_205406 | YBX3 | -0.22 | 0.13 | 5.26E-01 |
| NM_001198601 | PAPOLA | -0.21 | 0.13 | 5.26E-01 |
| NM_001277749 | MAD2L1 | 0.23 | 0.14 | 5.28E-01 |
| NM_001006464 | TMEM258 | 0.25 | 0.15 | 5.30E-01 |
| NM_001031050 | NEK2 | 0.20 | 0.12 | 5.31E-01 |
| NM_001199591 | RSU1 | 0.16 | 0.10 | 5.31E-01 |
| NM_001007087 | BLM | -0.16 | 0.10 | 5.32E-01 |
| NM_204848 | HPRT1 | 0.24 | 0.14 | 5.32E-01 |
| NM_001012918 | STK11IP | -0.23 | 0.14 | 5.33E-01 |
| NM_001277761 | TNS3 | -0.30 | 0.18 | 5.33E-01 |
| NM_001278153 | HN1L | 0.30 | 0.18 | 5.33E-01 |
| NM_001007473 | DAD1 | 0.21 | 0.13 | 5.33E-01 |
| NM_001012833 | YTHDF1 | -0.15 | 0.09 | 5.33E-01 |
| NM_001012883 | SLC25A14 | 0.20 | 0.12 | 5.33E-01 |
| NM_001012948 | VCPIP1 | -0.30 | 0.18 | 5.33E-01 |
| NM_001030539 | CRABP1 | 0.30 | 0.18 | 5.33E-01 |
| NM_001030707 | COG1 | -0.24 | 0.14 | 5.33E-01 |
| NM_001030790 | ADPRH | -0.26 | 0.16 | 5.33E-01 |
| NM_001031142 | HNRNPDL | 0.18 | 0.11 | 5.33E-01 |
| NM_001031382 | OGDH | -0.21 | 0.13 | 5.33E-01 |
| NM_001079738 | KIAA1671 | -0.26 | 0.16 | 5.33E-01 |
| NM_001281515 | PCGF2 | 0.27 | 0.16 | 5.33E-01 |
| NM_001293243 | LYSMD2 | 0.18 | 0.11 | 5.33E-01 |
| NM_204465 | ARVCF | -0.29 | 0.17 | 5.33E-01 |
| NM_205493 | GLUL | -0.24 | 0.14 | 5.33E-01 |
| NM_205523 | GOT2 | -0.16 | 0.10 | 5.33E-01 |
| NM_001006576 | DEPDC1B | 0.17 | 0.10 | 5.33E-01 |
| NM_001012768 | MYEF2 | 0.22 | 0.13 | 5.34E-01 |
| NM_205073 | TSN | -0.20 | 0.12 | 5.35E-01 |
| NM_001031207 | GTF2A1 | -0.16 | 0.10 | 5.35E-01 |
| NM_204470 | CCNL2 | -0.15 | 0.09 | 5.36E-01 |
| NM_001004380 | CAPZA2 | 0.13 | 0.08 | 5.36E-01 |
| NM_001031076 | GTF3C6 | 0.22 | 0.14 | 5.36E-01 |
| NM_001034828 | EXOSC9 | 0.19 | 0.11 | 5.36E-01 |
| NM_001199638 | OXNAD1 | 0.24 | 0.15 | 5.36E-01 |
| NM_001199715 | LMOD2 | 0.29 | 0.18 | 5.36E-01 |
| NM_001277750 | UBN2 | -0.27 | 0.16 | 5.36E-01 |
| NM_001031183 | PPP6R3 | -0.19 | 0.12 | 5.37E-01 |
| NM_001302159 | SEC61B | 0.24 | 0.15 | 5.37E-01 |
| NM_001012556 | DNAJB6 | 0.16 | 0.10 | 5.37E-01 |
| NM_001039600 | ZDHHC5 | -0.17 | 0.11 | 5.37E-01 |
| NM_001302156 | DYNLL2 | 0.22 | 0.14 | 5.37E-01 |
| NM_204740 | NCAN | -0.26 | 0.16 | 5.37E-01 |
| NM_001007967 | RPL30 | 0.24 | 0.15 | 5.37E-01 |
| NM_204943 | HSD17B4 | 0.17 | 0.10 | 5.38E-01 |
| NM_001030932 | HNRNPM | 0.21 | 0.13 | 5.39E-01 |
| NM_001031351 | TRMU | 0.23 | 0.14 | 5.39E-01 |
| NM_001277840 | MRPS25 | 0.24 | 0.15 | 5.41E-01 |
| NM_204206 | GIT2 | -0.29 | 0.18 | 5.41E-01 |
| NM_001001315 | TMSB4X | 0.22 | 0.13 | 5.41E-01 |
| NM_001006246 | ATP6V1E1 | 0.18 | 0.11 | 5.41E-01 |
| NM_001006477 | EIF2S1 | 0.18 | 0.11 | 5.41E-01 |
| NM_001012294 | POMGNT2 | -0.21 | 0.13 | 5.41E-01 |
| NM_001012813 | ATF7IP | -0.19 | 0.12 | 5.41E-01 |
| NM_001012932 | RPRD2 | -0.27 | 0.17 | 5.41E-01 |
| NM_001030830 | CRYL1 | -0.21 | 0.13 | 5.41E-01 |
| NM_001031363 | MED24 | -0.23 | 0.14 | 5.41E-01 |
| NM_001031370 | SUV39H2 | -0.17 | 0.10 | 5.41E-01 |
| NM_001031496 | USP28 | -0.14 | 0.09 | 5.41E-01 |
| NM_001039260 | WWP2 | -0.20 | 0.12 | 5.41E-01 |
| NM_001039330 | DNAJC16 | -0.27 | 0.17 | 5.41E-01 |
| NM_001044638 | CENPM | -0.23 | 0.14 | 5.41E-01 |
| NM_001135550 | MGST1 | 0.24 | 0.15 | 5.41E-01 |
| NM_001159511 | PACSIN2 | 0.24 | 0.15 | 5.41E-01 |
| NM_001199537 | AKAP8L | 0.24 | 0.15 | 5.41E-01 |
| NM_001199581 | GJC2 | -0.29 | 0.18 | 5.41E-01 |
| NM_204636 | MXRA8 | 0.18 | 0.11 | 5.41E-01 |
| NM_205117 | NFE2L2 | -0.29 | 0.18 | 5.41E-01 |
| NM_205299 | DMD | -0.20 | 0.12 | 5.41E-01 |
| NM_205367 | ACVR2A | -0.23 | 0.14 | 5.41E-01 |
| NM_001031023 | UBXN2B | 0.22 | 0.14 | 5.42E-01 |
| NM_001030869 | ZFP64 | -0.17 | 0.11 | 5.42E-01 |
| NM_001008456 | LGALSL | -0.20 | 0.12 | 5.43E-01 |
| NM_001030814 | NCK2 | -0.18 | 0.11 | 5.43E-01 |
| NM_001277670 | ASB12 | -0.22 | 0.14 | 5.43E-01 |
| NM_001006558 | GYG1 | 0.13 | 0.08 | 5.44E-01 |
| NM_001007975 | NANS | -0.15 | 0.10 | 5.44E-01 |
| NM_001031250 | CWC22 | 0.20 | 0.13 | 5.44E-01 |
| NM_001305566 | MPHOSPH8 | 0.25 | 0.16 | 5.44E-01 |
| NM_001031435 | PTGR1 | 0.18 | 0.11 | 5.45E-01 |
| NM_001008457 | B3GNT2 | 0.18 | 0.11 | 5.45E-01 |
| NM_205256 | TFRC | -0.21 | 0.13 | 5.45E-01 |
| NM_001030536 | ACO1 | 0.21 | 0.13 | 5.46E-01 |
| NM_001001296 | IFI6 | -0.29 | 0.18 | 5.48E-01 |
| NM_001001858 | STMN1 | 0.24 | 0.15 | 5.48E-01 |
| NM_001012862 | JARID2 | -0.21 | 0.13 | 5.48E-01 |
| NM_001030394 | HMG20A | -0.15 | 0.09 | 5.48E-01 |
| NM_001199615 | TWISTNB | 0.22 | 0.14 | 5.48E-01 |
| NM_001277571 | SMYD2 | 0.18 | 0.12 | 5.48E-01 |
| NM_204453 | TBX18 | -0.29 | 0.18 | 5.48E-01 |
| NM_204851 | SPON1 | -0.29 | 0.18 | 5.48E-01 |
| NM_205155 | FASN | -0.20 | 0.13 | 5.48E-01 |
| NM_205211 | GDF3 | -0.21 | 0.13 | 5.48E-01 |
| NM_205234 | HPCAL1 | 0.18 | 0.11 | 5.48E-01 |
| NM_205453 | TXN | 0.22 | 0.14 | 5.48E-01 |
| NM_001164348 | C11H16orf61 | 0.27 | 0.17 | 5.48E-01 |
| NM_001305666 | MTMR3 | -0.18 | 0.11 | 5.48E-01 |
| NM_205474 | MYH10 | 0.20 | 0.13 | 5.49E-01 |
| NM_001030692 | NAIF1 | -0.15 | 0.10 | 5.51E-01 |
| NM_001079756 | LRRC45 | 0.21 | 0.13 | 5.51E-01 |
| NM_001302166 | IER3IP1 | 0.23 | 0.15 | 5.51E-01 |
| NM_205337 | RPL27 | 0.22 | 0.14 | 5.53E-01 |
| NM_001012808 | CNOT2 | -0.16 | 0.10 | 5.53E-01 |
| NM_001030577 | COX4I1 | 0.24 | 0.15 | 5.53E-01 |
| NM_001030598 | ABTB1 | -0.22 | 0.14 | 5.53E-01 |
| NM_001031387 | IDH3B | 0.16 | 0.10 | 5.53E-01 |
| NM_001039317 | TRPC4AP | -0.14 | 0.09 | 5.53E-01 |
| NM_001277819 | FGL1 | 0.27 | 0.17 | 5.53E-01 |
| NM_204288 | MKRN2 | -0.19 | 0.12 | 5.53E-01 |
| NM_204426 | COL2A1 | -0.22 | 0.14 | 5.53E-01 |
| NM_204907 | CNBP | 0.17 | 0.11 | 5.53E-01 |
| NM_205096 | HDLBP | -0.23 | 0.14 | 5.53E-01 |
| NM_205209 | SLC2A1 | -0.18 | 0.11 | 5.53E-01 |
| NM_205535 | ATP1B3 | 0.13 | 0.08 | 5.53E-01 |
| NM_001030823 | SUGT1 | 0.21 | 0.14 | 5.54E-01 |
| NM_001277511 | BBIP1 | 0.20 | 0.12 | 5.55E-01 |
| NM_001305092 | WDR83OS | 0.25 | 0.16 | 5.55E-01 |
| NM_001277488 | LXN | -0.21 | 0.14 | 5.56E-01 |
| NM_205333 | P2RY1 | -0.28 | 0.18 | 5.57E-01 |
| NM_001012583 | CARS | 0.14 | 0.09 | 5.57E-01 |
| NM_001001466 | RTN1 | -0.25 | 0.16 | 5.58E-01 |
| NM_001006564 | ANP32E | -0.19 | 0.12 | 5.58E-01 |
| NM_001012602 | SEC62 | 0.19 | 0.12 | 5.58E-01 |
| NM_001012949 | SLC25A13 | -0.18 | 0.11 | 5.58E-01 |
| NM_001031448 | AMACR | 0.18 | 0.11 | 5.58E-01 |
| NM_001113709 | MYH1C | 0.24 | 0.16 | 5.58E-01 |
| NM_001277906 | PSMD10 | 0.21 | 0.13 | 5.58E-01 |
| NM_205176 | CRYAB | 0.28 | 0.18 | 5.58E-01 |
| NM_001006312 | ADPRHL2 | -0.17 | 0.11 | 5.58E-01 |
| NM_001039289 | SNX14 | -0.14 | 0.09 | 5.58E-01 |
| NM_001198646 | RAD51D | 0.18 | 0.11 | 5.58E-01 |
| NM_001277172 | COQ7 | 0.24 | 0.15 | 5.58E-01 |
| NM_001001615 | CDH2 | -0.24 | 0.15 | 5.58E-01 |
| NM_001006305 | NFU1 | 0.20 | 0.13 | 5.58E-01 |
| NM_001006311 | NUDC | 0.16 | 0.10 | 5.58E-01 |
| NM_001006320 | TRAPPC4 | 0.21 | 0.13 | 5.58E-01 |
| NM_001006370 | PDIA4 | 0.18 | 0.11 | 5.58E-01 |
| NM_001012781 | DENND6A | -0.15 | 0.10 | 5.58E-01 |
| NM_001030742 | MDM1 | -0.17 | 0.11 | 5.58E-01 |
| NM_001031371 | PPP2R4 | -0.13 | 0.08 | 5.58E-01 |
| NM_001039280 | PARD3 | -0.24 | 0.16 | 5.58E-01 |
| NM_001044667 | ISLR2 | -0.28 | 0.18 | 5.58E-01 |
| NM_001198637 | YIPF5 | 0.20 | 0.13 | 5.58E-01 |
| NM_001199000 | NFAT5 | -0.27 | 0.17 | 5.58E-01 |
| NM_001199848 | RAB5B | -0.19 | 0.12 | 5.58E-01 |
| NM_001267575 | DNAJC17 | 0.23 | 0.15 | 5.58E-01 |
| NM_001277376 | PHF5A | 0.21 | 0.13 | 5.58E-01 |
| NM_001277915 | ELMOD2 | -0.28 | 0.18 | 5.58E-01 |
| NM_001291971 | SIRT7 | -0.20 | 0.13 | 5.58E-01 |
| NM_204415 | CHRNA5 | -0.28 | 0.18 | 5.58E-01 |
| NM_204497 | NF2 | -0.19 | 0.12 | 5.58E-01 |
| NM_001277779 | ACAT1 | 0.24 | 0.16 | 5.58E-01 |
| NM_001005618 | PLOD1 | 0.17 | 0.11 | 5.60E-01 |
| NM_001012403 | MBD2 | -0.22 | 0.15 | 5.60E-01 |
| NM_001012554 | WWP1 | -0.24 | 0.16 | 5.60E-01 |
| NM_001012597 | ABCD3 | -0.23 | 0.15 | 5.60E-01 |
| NM_001012782 | QARS | -0.17 | 0.11 | 5.60E-01 |
| NM_001030701 | Sep-09 | -0.17 | 0.11 | 5.60E-01 |
| NM_001031030 | ZC3H11A | -0.21 | 0.13 | 5.60E-01 |
| NM_001031172 | VPS51 | -0.18 | 0.12 | 5.60E-01 |
| NM_001079730 | ADD1 | -0.18 | 0.12 | 5.60E-01 |
| NM_001109692 | BAG1 | 0.22 | 0.14 | 5.60E-01 |
| NM_001252034 | CCDC18 | 0.24 | 0.16 | 5.60E-01 |
| NM_001282235 | BEND7 | -0.22 | 0.15 | 5.60E-01 |
| NM_204575 | LIFR | -0.20 | 0.13 | 5.60E-01 |
| NM_204641 | FKBP3 | 0.23 | 0.15 | 5.60E-01 |
| NM_204805 | TH | 0.27 | 0.18 | 5.60E-01 |
| NM_205187 | SOX11 | -0.24 | 0.16 | 5.60E-01 |
| NM_001006334 | NT5C3B | -0.19 | 0.12 | 5.60E-01 |
| NM_001030653 | PPIL2 | -0.14 | 0.09 | 5.60E-01 |
| NM_001031502 | DNAJC7 | 0.16 | 0.11 | 5.60E-01 |
| NM_001040707 | KIAA0586 | -0.20 | 0.13 | 5.60E-01 |
| NM_001199543 | BAHD1 | -0.28 | 0.18 | 5.60E-01 |
| NM_204261 | ADAM10 | -0.14 | 0.09 | 5.60E-01 |
| NM_204825 | UCHL3 | 0.22 | 0.14 | 5.60E-01 |
| NM_001012891 | KDM3A | -0.17 | 0.11 | 5.60E-01 |
| NM_001277471 | MRPL13 | 0.22 | 0.14 | 5.60E-01 |
| NM_001257314 | TRIM2 | -0.26 | 0.17 | 5.60E-01 |
| NM_205407 | PTPRS | -0.28 | 0.18 | 5.60E-01 |
| NM_001307950 | URAHP | 0.22 | 0.15 | 5.61E-01 |
| NM_204798 | KCNN2 | -0.26 | 0.17 | 5.61E-01 |
| NM_001012945 | DNAJA1 | 0.17 | 0.11 | 5.61E-01 |
| NM_001030756 | NFE2L1 | -0.11 | 0.07 | 5.61E-01 |
| NM_001031028 | MYBPH | -0.22 | 0.15 | 5.61E-01 |
| NM_001293184 | SIN3A | -0.28 | 0.18 | 5.61E-01 |
| NM_001302134 | TOMM6 | 0.25 | 0.16 | 5.61E-01 |
| NM_205243 | YAP1 | -0.28 | 0.18 | 5.61E-01 |
| NM_001195423 | MEMO1 | -0.20 | 0.13 | 5.62E-01 |
| NM_001199392 | TTL | -0.12 | 0.08 | 5.64E-01 |
| NM_001305558 | RBL2 | -0.28 | 0.18 | 5.64E-01 |
| NM_204306 | PDGFA | 0.25 | 0.17 | 5.64E-01 |
| NM_001030540 | ACLY | -0.13 | 0.08 | 5.64E-01 |
| NM_001277370 | EMP1 | 0.23 | 0.15 | 5.65E-01 |
| NM_001277175 | SNRNP35 | 0.24 | 0.16 | 5.66E-01 |
| NM_001105675 | MCTS1 | 0.21 | 0.14 | 5.66E-01 |
| NM_204189 | UNG | -0.18 | 0.12 | 5.66E-01 |
| NM_001039276 | AGO4 | -0.25 | 0.16 | 5.67E-01 |
| NM_001079493 | RPL37A | 0.22 | 0.14 | 5.67E-01 |
| NM_001081709 | TLR1LB | 0.24 | 0.16 | 5.67E-01 |
| NM_001130488 | PPAP2B | -0.18 | 0.12 | 5.67E-01 |
| NM_204682 | SIK1 | 0.25 | 0.16 | 5.67E-01 |
| NM_204918 | TJP2 | 0.16 | 0.11 | 5.67E-01 |
| NM_204490 | HS6ST2 | -0.27 | 0.18 | 5.68E-01 |
| NM_001006248 | DERA | 0.20 | 0.13 | 5.68E-01 |
| NM_001040090 | AR | -0.24 | 0.16 | 5.68E-01 |
| NM_001080887 | ENG | 0.22 | 0.15 | 5.68E-01 |
| NM_001030917 | AP4B1 | -0.21 | 0.14 | 5.70E-01 |
| NM_001031060 | CEBPZ | 0.16 | 0.10 | 5.70E-01 |
| NM_001277827 | MYOZ2 | 0.23 | 0.15 | 5.70E-01 |
| NM_205322 | RPLP1 | 0.24 | 0.16 | 5.71E-01 |
| NM_001001304 | POLH | -0.18 | 0.12 | 5.71E-01 |
| NM_001030826 | RNASEH2B | 0.19 | 0.12 | 5.72E-01 |
| NM_205307 | RAF1 | -0.23 | 0.16 | 5.72E-01 |
| NM_001080861 | EIF4H | -0.14 | 0.09 | 5.73E-01 |
| NM_001277752 | FAHD1 | 0.15 | 0.10 | 5.73E-01 |
| NM_001006205 | ACOX1 | -0.15 | 0.10 | 5.74E-01 |
| NM_001007849 | TSPAN12 | 0.15 | 0.10 | 5.74E-01 |
| NM_001007948 | ACTR1A | -0.22 | 0.15 | 5.74E-01 |
| NM_001012890 | CTBP1 | -0.17 | 0.11 | 5.74E-01 |
| NM_001024691 | PYGO1 | -0.18 | 0.12 | 5.74E-01 |
| NM_001030722 | INTS2 | -0.21 | 0.14 | 5.74E-01 |
| NM_001030966 | INSIG1 | -0.21 | 0.14 | 5.74E-01 |
| NM_001030995 | STK17A | 0.18 | 0.12 | 5.74E-01 |
| NM_001195469 | ZNF277 | 0.17 | 0.12 | 5.74E-01 |
| NM_001199454 | HBP1 | -0.27 | 0.18 | 5.74E-01 |
| NM_001199526 | UBE2D1 | 0.15 | 0.10 | 5.74E-01 |
| NM_001201487 | BCL10 | 0.16 | 0.11 | 5.74E-01 |
| NM_001257200 | ATP5J2 | 0.25 | 0.16 | 5.74E-01 |
| NM_001277392 | NUP37 | 0.18 | 0.12 | 5.74E-01 |
| NM_001277586 | PDCD5 | 0.15 | 0.10 | 5.74E-01 |
| NM_001277738 | EGFL6 | 0.23 | 0.16 | 5.74E-01 |
| NM_204233 | BTK | -0.27 | 0.18 | 5.74E-01 |
| NM_204397 | PSMB7 | 0.24 | 0.16 | 5.74E-01 |
| NM_205062 | LRPAP1 | 0.20 | 0.13 | 5.74E-01 |
| NM_205133 | TNNC1 | 0.26 | 0.18 | 5.74E-01 |
| NM_205207 | MFI2 | -0.23 | 0.15 | 5.74E-01 |
| NM_205365 | GSTT1 | 0.22 | 0.15 | 5.74E-01 |
| NM_001031203 | NPC2 | -0.26 | 0.17 | 5.74E-01 |
| NM_001004379 | RPL7A | 0.21 | 0.14 | 5.75E-01 |
| NM_001012922 | STAM2 | -0.17 | 0.11 | 5.75E-01 |
| NM_001030948 | DCAF13 | 0.15 | 0.10 | 5.75E-01 |
| NM_001048076 | VIM | 0.15 | 0.10 | 5.75E-01 |
| NM_001278155 | HACE1 | -0.18 | 0.12 | 5.75E-01 |
| NM_204772 | FRZB | 0.16 | 0.11 | 5.75E-01 |
| NM_001006132 | AP1G1 | -0.13 | 0.08 | 5.75E-01 |
| NM_001167764 | HSF2 | 0.15 | 0.10 | 5.75E-01 |
| NM_001199618 | JAZF1 | -0.14 | 0.10 | 5.75E-01 |
| NM_001271931 | FZD6 | -0.14 | 0.09 | 5.75E-01 |
| NM_001305060 | GPC1 | -0.18 | 0.12 | 5.75E-01 |
| NM_204210 | DIAPH1 | -0.17 | 0.12 | 5.75E-01 |
| NM_204707 | SALL1 | -0.22 | 0.15 | 5.75E-01 |
| NM_204758 | C5H11ORF58 | 0.21 | 0.14 | 5.75E-01 |
| NM_001030802 | KLHL15 | -0.25 | 0.17 | 5.77E-01 |
| NM_001195551 | SUGP1 | -0.18 | 0.12 | 5.77E-01 |
| NM_001277685 | SLC31A2 | 0.24 | 0.17 | 5.77E-01 |
| NM_001039486 | VAMP1 | -0.24 | 0.16 | 5.77E-01 |
| NM_001044648 | KATNA1 | -0.18 | 0.12 | 5.77E-01 |
| NM_001276357 | LUM | 0.27 | 0.18 | 5.77E-01 |
| NM_205388 | MAP2K2 | -0.13 | 0.09 | 5.77E-01 |
| NM_001005835 | E2F4 | -0.18 | 0.13 | 5.78E-01 |
| NM_205342 | LBR | -0.22 | 0.15 | 5.78E-01 |
| NM_001031284 | NRD1 | -0.18 | 0.12 | 5.79E-01 |
| NM_205240 | ST6GALNAC1 | -0.27 | 0.18 | 5.79E-01 |
| NM_001199451 | CD93 | -0.27 | 0.18 | 5.79E-01 |
| NM_001030825 | NUFIP1 | 0.16 | 0.11 | 5.79E-01 |
| NM_001199406 | DEPDC7 | 0.20 | 0.14 | 5.79E-01 |
| NM_001199584 | GALNT18 | 0.18 | 0.12 | 5.81E-01 |
| NM_001302107 | NDUFA4 | 0.22 | 0.15 | 5.82E-01 |
| NM_001006487 | EVL | 0.14 | 0.09 | 5.82E-01 |
| NM_001030910 | UBE4A | -0.14 | 0.09 | 5.82E-01 |
| NM_001162382 | LYPLA2 | -0.17 | 0.12 | 5.82E-01 |
| NM_001204816 | TNNI3K | -0.19 | 0.13 | 5.82E-01 |
| NM_001277254 | CPN1 | 0.23 | 0.16 | 5.82E-01 |
| NM_001277495 | NUB1 | 0.17 | 0.12 | 5.82E-01 |
| NM_001030591 | SHISA5 | -0.16 | 0.11 | 5.83E-01 |
| NM_001031376 | EED | 0.20 | 0.14 | 5.83E-01 |
| NM_001305089 | MCM9 | -0.26 | 0.18 | 5.83E-01 |
| NM_001006177 | APMAP | 0.17 | 0.12 | 5.83E-01 |
| NM_001109787 | YRK | -0.22 | 0.15 | 5.83E-01 |
| NM_001190924 | G0S2 | 0.23 | 0.16 | 5.83E-01 |
| NM_001199385 | MRPL23 | 0.23 | 0.16 | 5.83E-01 |
| NM_204098 | FZD10 | -0.26 | 0.18 | 5.83E-01 |
| NM_001031378 | LOC426385 | 0.22 | 0.15 | 5.84E-01 |
| NM_204495 | CRMP1 | -0.25 | 0.17 | 5.84E-01 |
| NM_001004369 | CCNB2 | 0.15 | 0.10 | 5.85E-01 |
| NM_001031052 | PPM1B | -0.17 | 0.12 | 5.85E-01 |
| NM_001278108 | NDUFA6 | 0.22 | 0.15 | 5.85E-01 |
| NM_001305470 | ETFA | 0.21 | 0.15 | 5.85E-01 |
| NM_205504 | GJA5 | -0.17 | 0.12 | 5.85E-01 |
| NM_001001879 | CYP4V2 | -0.25 | 0.18 | 5.85E-01 |
| NM_001006583 | MRPL50 | 0.20 | 0.14 | 5.85E-01 |
| NM_001031234 | NT5C2 | -0.16 | 0.11 | 5.85E-01 |
| NM_001198648 | CSTB | 0.20 | 0.14 | 5.85E-01 |
| NM_001252329 | NEMF | 0.21 | 0.14 | 5.85E-01 |
| NM_001278031 | DAPK1 | -0.26 | 0.18 | 5.85E-01 |
| NM_001302146 | LOC771947 | 0.23 | 0.16 | 5.85E-01 |
| NM_204127 | ACTN1 | 0.21 | 0.14 | 5.85E-01 |
| NM_204688 | HINTW | 0.24 | 0.16 | 5.85E-01 |
| NM_204963 | SLC4A2 | -0.19 | 0.13 | 5.85E-01 |
| NM_001278018 | WBP2 | 0.16 | 0.11 | 5.86E-01 |
| NM_001282277 | RPL17 | 0.19 | 0.13 | 5.86E-01 |
| NM_204664 | C4BPA | -0.17 | 0.12 | 5.86E-01 |
| NM_204947 | RFNG | -0.16 | 0.11 | 5.86E-01 |
| NM_001006170 | FOPNL | 0.20 | 0.14 | 5.87E-01 |
| NM_001305140 | TUBA3E | 0.19 | 0.13 | 5.87E-01 |
| NM_001001782 | MPL | -0.24 | 0.16 | 5.88E-01 |
| NM_001006271 | SUCLA2 | 0.17 | 0.12 | 5.88E-01 |
| NM_001006536 | CDC20 | 0.20 | 0.14 | 5.88E-01 |
| NM_001012840 | PUM1 | -0.22 | 0.16 | 5.88E-01 |
| NM_001012944 | SKIV2L2 | 0.14 | 0.10 | 5.88E-01 |
| NM_001030694 | FBXW5 | -0.13 | 0.09 | 5.88E-01 |
| NM_001030924 | PLEKHM1 | -0.18 | 0.12 | 5.88E-01 |
| NM_001031121 | VAMP7 | -0.13 | 0.09 | 5.88E-01 |
| NM_001031263 | WDSUB1 | 0.16 | 0.11 | 5.88E-01 |
| NM_001031427 | GNAQ | -0.22 | 0.15 | 5.88E-01 |
| NM_001199423 | CFAP36 | 0.18 | 0.13 | 5.88E-01 |
| NM_204191 | SPINW | -0.18 | 0.12 | 5.88E-01 |
| NM_204229 | RGS19 | -0.20 | 0.14 | 5.88E-01 |
| NM_001006143 | EDEM1 | -0.26 | 0.18 | 5.89E-01 |
| NM_001031117 | MST4 | -0.17 | 0.12 | 5.89E-01 |
| NM_001277806 | Sep-08 | 0.18 | 0.13 | 5.89E-01 |
| NM_001277866 | MRPL41 | 0.19 | 0.13 | 5.89E-01 |
| NM_204211 | SOD2 | 0.20 | 0.14 | 5.89E-01 |
| NM_204273 | RPL35 | 0.22 | 0.15 | 5.89E-01 |
| NM_205070 | LDB1 | -0.24 | 0.17 | 5.89E-01 |
| NM_206984 | GJA4 | -0.22 | 0.15 | 5.90E-01 |
| NM_001001743 | FGF13 | -0.26 | 0.18 | 5.90E-01 |
| NM_001012903 | ANXA11 | 0.14 | 0.10 | 5.90E-01 |
| NM_001029980 | PRR5 | -0.19 | 0.13 | 5.90E-01 |
| NM_001031053 | PREPL | -0.26 | 0.18 | 5.90E-01 |
| NM_001082417 | RPS3A | 0.21 | 0.14 | 5.90E-01 |
| NM_001199550 | AMIGO2 | -0.20 | 0.14 | 5.90E-01 |
| NM_001277576 | MRPS10 | 0.23 | 0.16 | 5.90E-01 |
| NM_001031182 | PDE3B | -0.20 | 0.14 | 5.90E-01 |
| NM_001044680 | GNLY | 0.24 | 0.17 | 5.92E-01 |
| NM_001252077 | REEP5 | 0.20 | 0.14 | 5.92E-01 |
| NM_204689 | HINT1 | 0.21 | 0.14 | 5.92E-01 |
| NM_001199520 | BMS1 | 0.17 | 0.12 | 5.93E-01 |
| NM_204581 | RPL5 | 0.19 | 0.13 | 5.93E-01 |
| NM_204643 | CBX3 | 0.15 | 0.10 | 5.93E-01 |
| NM_001006438 | MSMO1 | 0.16 | 0.11 | 5.94E-01 |
| NM_001302167 | NDUFB3 | 0.25 | 0.18 | 5.94E-01 |
| NM_001130738 | PLA2G6 | -0.24 | 0.17 | 5.94E-01 |
| NM_001039313 | CLTA | 0.21 | 0.15 | 5.94E-01 |
| NM_001199418 | AHSA2 | 0.15 | 0.11 | 5.94E-01 |
| NM_001005819 | BTBD1 | 0.13 | 0.09 | 5.95E-01 |
| NM_001031062 | GPR137B | 0.22 | 0.15 | 5.95E-01 |
| NM_001277967 | MRPS21 | 0.22 | 0.15 | 5.95E-01 |
| NM_001305680 | CNTRL | 0.17 | 0.12 | 5.95E-01 |
| NM_204765 | MEOX1 | 0.24 | 0.17 | 5.95E-01 |
| NM_205269 | NFYB | 0.16 | 0.12 | 5.95E-01 |
| NM_001012811 | CNOT4 | -0.25 | 0.18 | 5.96E-01 |
| NM_001199416 | USP34 | -0.18 | 0.13 | 5.96E-01 |
| NM_001276362 | PTN | -0.17 | 0.12 | 5.96E-01 |
| NM_001277652 | XPO5 | -0.19 | 0.13 | 5.96E-01 |
| NM_204353 | IGFBP4 | -0.19 | 0.13 | 5.96E-01 |
| NM_204549 | EIF4A2 | 0.10 | 0.07 | 5.96E-01 |
| NM_001080888 | RCHY1 | 0.19 | 0.14 | 5.97E-01 |
| NM_001199603 | SLC38A1 | -0.20 | 0.14 | 5.98E-01 |
| NM_001018012 | ALAS1 | 0.15 | 0.11 | 5.98E-01 |
| NM_001006563 | RHNO1 | 0.23 | 0.16 | 5.99E-01 |
| NM_001032398 | EEF1A2 | -0.16 | 0.11 | 5.99E-01 |
| NM_001277486 | ARPC3 | 0.19 | 0.14 | 5.99E-01 |
| NM_001277995 | DHRS7C | 0.21 | 0.15 | 5.99E-01 |
| NM_001302115 | NDUFA1 | 0.23 | 0.16 | 5.99E-01 |
| NM_205202 | B4GALT1 | -0.18 | 0.13 | 5.99E-01 |
| NM_205401 | TPM1 | 0.19 | 0.14 | 5.99E-01 |
| NM_205508 | FOS | 0.25 | 0.17 | 5.99E-01 |
| NM_001006473 | RNH1 | 0.16 | 0.11 | 5.99E-01 |
| NM_001030824 | WBP4 | -0.17 | 0.12 | 5.99E-01 |
| NM_001031018 | UBE2V2 | 0.17 | 0.12 | 5.99E-01 |
| NM_001039326 | SOX17 | 0.24 | 0.17 | 5.99E-01 |
| NM_001080865 | SRI | 0.14 | 0.10 | 5.99E-01 |
| NM_001137650 | CAST | -0.24 | 0.17 | 5.99E-01 |
| NM_001168002 | ATP2B1 | -0.16 | 0.11 | 5.99E-01 |
| NM_001178132 | FSCN1 | -0.23 | 0.17 | 5.99E-01 |
| NM_001199655 | VPS18 | -0.25 | 0.18 | 5.99E-01 |
| NM_001257318 | USP40 | -0.25 | 0.18 | 5.99E-01 |
| NM_001277596 | CCDC167 | 0.19 | 0.13 | 5.99E-01 |
| NM_001277709 | PEX7 | 0.20 | 0.15 | 5.99E-01 |
| NM_204163 | PSEN1 | -0.25 | 0.18 | 5.99E-01 |
| NM_204567 | FKBP1B | 0.20 | 0.14 | 5.99E-01 |
| NM_204588 | XIAP | -0.24 | 0.17 | 5.99E-01 |
| NM_205395 | SLMAP | 0.18 | 0.13 | 5.99E-01 |
| NM_001006199 | FUBP3 | -0.19 | 0.14 | 6.00E-01 |
| NM_001006231 | RPAP3 | 0.12 | 0.09 | 6.00E-01 |
| NM_001006394 | PLK1S1 | 0.20 | 0.14 | 6.00E-01 |
| NM_001012804 | PRKCA | -0.20 | 0.14 | 6.00E-01 |
| NM_001030587 | MON1A | -0.16 | 0.11 | 6.00E-01 |
| NM_001031080 | PNISR | 0.22 | 0.16 | 6.00E-01 |
| NM_001031202 | FAM177A1 | -0.14 | 0.10 | 6.00E-01 |
| NM_001031353 | ADRBK1 | -0.14 | 0.10 | 6.00E-01 |
| NM_001177385 | CLASP2 | -0.24 | 0.17 | 6.00E-01 |
| NM_001278146 | C12ORF57 | 0.24 | 0.17 | 6.00E-01 |
| NM_001287202 | PHF13 | -0.20 | 0.14 | 6.00E-01 |
| NM_001302186 | MRPL34 | 0.21 | 0.15 | 6.00E-01 |
| NM_001006224 | TRIM37 | -0.15 | 0.11 | 6.00E-01 |
| NM_204919 | MAP6 | -0.25 | 0.18 | 6.00E-01 |
| NM_001006504 | NHLRC2 | 0.21 | 0.15 | 6.02E-01 |
| NM_204464 | RHAG | -0.16 | 0.12 | 6.03E-01 |
| NM_205359 | IGFBP2 | 0.13 | 0.09 | 6.03E-01 |
| NM_001012593 | FAM21A | 0.12 | 0.09 | 6.04E-01 |
| NM_001012693 | MGAT3 | -0.19 | 0.14 | 6.04E-01 |
| NM_001030828 | SLC46A3 | -0.25 | 0.18 | 6.04E-01 |
| NM_001252606 | AUH | 0.14 | 0.10 | 6.04E-01 |
| NM_204679 | TWIST2 | 0.21 | 0.15 | 6.04E-01 |
| NM_205135 | MYOM2 | 0.25 | 0.18 | 6.04E-01 |
| NM_205446 | TPM2 | 0.16 | 0.11 | 6.04E-01 |
| NM_001006376 | EXOC2 | -0.22 | 0.16 | 6.04E-01 |
| NM_001030744 | KRR1 | 0.23 | 0.17 | 6.04E-01 |
| NM_001030983 | FAM126A | -0.20 | 0.15 | 6.04E-01 |
| NM_001081708 | GTF2A2 | 0.22 | 0.16 | 6.04E-01 |
| NM_001303453 | ZW10 | -0.20 | 0.15 | 6.04E-01 |
| NM_205521 | ATP1A1 | 0.11 | 0.08 | 6.04E-01 |
| NM_001012404 | EPGN | 0.24 | 0.17 | 6.06E-01 |
| NM_001256162 | KRAS | 0.21 | 0.15 | 6.06E-01 |
| NM_001257372 | MARS | -0.18 | 0.13 | 6.06E-01 |
| NM_001293314 | RNF123 | -0.14 | 0.10 | 6.06E-01 |
| NM_204392 | PYGL | -0.16 | 0.11 | 6.06E-01 |
| NM_204563 | DDX1 | 0.14 | 0.10 | 6.06E-01 |
| NM_204762 | CD9 | 0.25 | 0.18 | 6.06E-01 |
| NM_001278049 | LPAR4 | -0.16 | 0.11 | 6.06E-01 |
| NM_001006584 | DHFR | 0.18 | 0.13 | 6.07E-01 |
| NM_001079491 | FEN1 | 0.15 | 0.11 | 6.07E-01 |
| NM_001166325 | COTL1 | -0.24 | 0.17 | 6.07E-01 |
| NM_206951 | EPHB2 | -0.19 | 0.14 | 6.07E-01 |
| NM_001098610 | FAAP100 | -0.16 | 0.11 | 6.09E-01 |
| NM_001006338 | RNF126 | -0.17 | 0.12 | 6.09E-01 |
| NM_205165 | PBRM1 | -0.18 | 0.13 | 6.09E-01 |
| NM_001197245 | EAPP | 0.14 | 0.10 | 6.11E-01 |
| NM_001031300 | LOC424740 | -0.13 | 0.09 | 6.11E-01 |
| NM_001039314 | FNIP1 | -0.24 | 0.18 | 6.11E-01 |
| NM_001197042 | SLIT3 | -0.20 | 0.14 | 6.11E-01 |
| NM_001256807 | COX19 | 0.20 | 0.15 | 6.11E-01 |
| NM_204705 | HABP4 | 0.20 | 0.15 | 6.11E-01 |
| NM_001012860 | PSMA2 | 0.18 | 0.14 | 6.12E-01 |
| NM_204207 | HAT1 | 0.18 | 0.13 | 6.12E-01 |
| NM_204958 | PSMC1 | 0.17 | 0.13 | 6.12E-01 |
| NM_001007979 | SMU1 | -0.12 | 0.09 | 6.15E-01 |
| NM_001012904 | CHUK | -0.19 | 0.14 | 6.15E-01 |
| NM_001122777 | TXNRD3 | 0.11 | 0.08 | 6.15E-01 |
| NM_204637 | ACTR6 | 0.19 | 0.14 | 6.15E-01 |
| NM_001039292 | PUM2 | -0.23 | 0.17 | 6.15E-01 |
| NM_001012855 | GLCCI1 | -0.13 | 0.09 | 6.15E-01 |
| NM_001031466 | TMEM11 | 0.16 | 0.12 | 6.15E-01 |
| NM_001031577 | PLAG1 | -0.24 | 0.18 | 6.15E-01 |
| NM_204226 | PKNOX2 | -0.24 | 0.18 | 6.15E-01 |
| NM_001012605 | ARL6IP5 | 0.13 | 0.10 | 6.16E-01 |
| NM_205420 | GATA6 | -0.22 | 0.16 | 6.16E-01 |
| NM_001006167 | LCMT1 | 0.17 | 0.13 | 6.16E-01 |
| NM_001006239 | RPS19BP1 | 0.18 | 0.13 | 6.16E-01 |
| NM_001012769 | SPPL2A | -0.11 | 0.08 | 6.16E-01 |
| NM_001012868 | ARL8A | 0.15 | 0.11 | 6.16E-01 |
| NM_001024831 | TRDMT1 | 0.22 | 0.16 | 6.16E-01 |
| NM_001030926 | DDX42 | -0.12 | 0.09 | 6.16E-01 |
| NM_001031412 | C1H11ORF73 | 0.20 | 0.15 | 6.16E-01 |
| NM_001079752 | CHAF1A | 0.13 | 0.10 | 6.16E-01 |
| NM_001080884 | TESC | 0.21 | 0.15 | 6.16E-01 |
| NM_001110177 | SOBP | -0.23 | 0.17 | 6.16E-01 |
| NM_001199414 | QSER1 | -0.22 | 0.17 | 6.16E-01 |
| NM_001199429 | NPHP1 | 0.17 | 0.13 | 6.16E-01 |
| NM_001254739 | FKBP8 | -0.17 | 0.12 | 6.16E-01 |
| NM_001277358 | ZCRB1 | 0.16 | 0.12 | 6.16E-01 |
| NM_001277617 | GAS8 | 0.20 | 0.14 | 6.16E-01 |
| NM_001277886 | TAF7 | 0.18 | 0.14 | 6.16E-01 |
| NM_001305200 | SCP2 | 0.18 | 0.13 | 6.16E-01 |
| NM_204100 | TFAM | 0.18 | 0.13 | 6.16E-01 |
| NM_204487 | EIF2AK2 | -0.18 | 0.14 | 6.16E-01 |
| NM_204493 | DPYSL3 | 0.15 | 0.11 | 6.16E-01 |
| NM_205528 | DSTN | 0.17 | 0.12 | 6.16E-01 |
| NM_001006368 | TGM4 | 0.23 | 0.17 | 6.17E-01 |
| NM_001006485 | VRK1 | 0.14 | 0.10 | 6.17E-01 |
| NM_001024734 | VIMP | 0.17 | 0.13 | 6.17E-01 |
| NM_001031348 | VPS33B | -0.14 | 0.10 | 6.17E-01 |
| NM_001039605 | PRKAA2 | -0.24 | 0.18 | 6.17E-01 |
| NM_001282194 | CLN5 | -0.20 | 0.15 | 6.17E-01 |
| NM_204219 | TRAF5 | -0.22 | 0.16 | 6.17E-01 |
| NM_001004368 | KDR | -0.18 | 0.13 | 6.18E-01 |
| NM_001005837 | HSDL1 | 0.17 | 0.13 | 6.18E-01 |
| NM_001006685 | HSPA2 | 0.17 | 0.12 | 6.18E-01 |
| NM_001012875 | SCCPDH | -0.12 | 0.09 | 6.18E-01 |
| NM_001031027 | ADIPOR1 | -0.12 | 0.09 | 6.18E-01 |
| NM_001031102 | SUPT3H | -0.18 | 0.13 | 6.18E-01 |
| NM_001031566 | DBR1 | -0.18 | 0.13 | 6.18E-01 |
| NM_001079483 | MTX3 | 0.14 | 0.11 | 6.18E-01 |
| NM_001205097 | UBE2V1 | 0.11 | 0.08 | 6.18E-01 |
| NM_001252126 | RPS8 | 0.20 | 0.15 | 6.18E-01 |
| NM_001277508 | TRIAP1 | 0.16 | 0.12 | 6.18E-01 |
| NM_001302172 | POLR2L | 0.21 | 0.16 | 6.18E-01 |
| NM_204213 | CCND2 | -0.24 | 0.18 | 6.18E-01 |
| NM_204647 | SALL3 | -0.24 | 0.18 | 6.18E-01 |
| NM_001006138 | RUVBL1 | 0.16 | 0.12 | 6.19E-01 |
| NM_001031174 | RIC8A | -0.14 | 0.10 | 6.19E-01 |
| NM_001163003 | SMIM5 | -0.21 | 0.16 | 6.19E-01 |
| NM_001278148 | PCP4 | 0.23 | 0.17 | 6.19E-01 |
| NM_205265 | NCL | 0.21 | 0.16 | 6.19E-01 |
| NM_205403 | GNAI1 | 0.18 | 0.14 | 6.19E-01 |
| NM_001079727 | RCOR3 | -0.24 | 0.18 | 6.21E-01 |
| NM_204827 | DDX5 | 0.12 | 0.09 | 6.21E-01 |
| NM_001024586 | TLR5 | -0.23 | 0.17 | 6.22E-01 |
| NM_001031557 | MOB4 | 0.14 | 0.10 | 6.24E-01 |
| NM_001030804 | EIF1AY | 0.20 | 0.15 | 6.25E-01 |
| NM_204865 | UBE2A | -0.15 | 0.11 | 6.25E-01 |
| NM_001198888 | LYRM4 | 0.18 | 0.14 | 6.27E-01 |
| NM_001039321 | LOC428335 | -0.15 | 0.11 | 6.27E-01 |
| NM_001098609 | PCDH8 | -0.22 | 0.17 | 6.28E-01 |
| NM_001012577 | TBCK | 0.17 | 0.13 | 6.28E-01 |
| NM_001031200 | C5H15ORF41 | -0.17 | 0.13 | 6.28E-01 |
| NM_001039489 | VAMP3 | 0.20 | 0.15 | 6.28E-01 |
| NM_204545 | SULT1B1 | 0.14 | 0.10 | 6.28E-01 |
| NM_001031461 | MAP1LC3B | -0.14 | 0.11 | 6.28E-01 |
| NM_001031605 | RFK | 0.15 | 0.11 | 6.28E-01 |
| NM_001302096 | SH3BGRL3 | 0.12 | 0.09 | 6.28E-01 |
| NM_001305162 | TAF2 | -0.13 | 0.10 | 6.28E-01 |
| NM_205210 | ZNF335 | -0.15 | 0.11 | 6.28E-01 |
| NM_001012574 | SH3BGRL | -0.10 | 0.07 | 6.28E-01 |
| NM_001031046 | GPATCH2 | -0.18 | 0.14 | 6.30E-01 |
| NM_001199710 | DPH6 | 0.17 | 0.13 | 6.31E-01 |
| NM_001302192 | SVIP | 0.20 | 0.15 | 6.31E-01 |
| NM_001004383 | LARGE | -0.24 | 0.18 | 6.32E-01 |
| NM_001006218 | CHCHD2 | 0.18 | 0.14 | 6.32E-01 |
| NM_001006294 | NELFCD | -0.15 | 0.12 | 6.32E-01 |
| NM_001006303 | PGD | 0.15 | 0.11 | 6.32E-01 |
| NM_001006331 | CDC27 | -0.11 | 0.08 | 6.32E-01 |
| NM_001012847 | ELL | 0.20 | 0.15 | 6.32E-01 |
| NM_001030676 | GARNL3 | -0.17 | 0.13 | 6.32E-01 |
| NM_001030905 | RP11-49K24.9 | -0.11 | 0.08 | 6.32E-01 |
| NM_001031035 | PTK7 | -0.12 | 0.10 | 6.32E-01 |
| NM_001031307 | RNF7 | 0.15 | 0.12 | 6.32E-01 |
| NM_001031321 | MFSD1 | -0.13 | 0.10 | 6.32E-01 |
| NM_001031352 | PDK1 | -0.22 | 0.17 | 6.32E-01 |
| NM_001031396 | ADAM9 | -0.11 | 0.08 | 6.32E-01 |
| NM_001079726 | LOC420419 | 0.17 | 0.13 | 6.32E-01 |
| NM_001112806 | CKS1B | 0.18 | 0.14 | 6.32E-01 |
| NM_001145229 | PROKR2 | -0.23 | 0.18 | 6.32E-01 |
| NM_001252062 | ANKS1B | 0.22 | 0.17 | 6.32E-01 |
| NM_001257373 | RP11-108K14.8 | -0.23 | 0.18 | 6.32E-01 |
| NM_001271929 | MYL2 | 0.19 | 0.15 | 6.32E-01 |
| NM_001277529 | BAIAP2L1 | 0.19 | 0.15 | 6.32E-01 |
| NM_001277722 | GEMIN6 | 0.20 | 0.15 | 6.32E-01 |
| NM_001277879 | LOC427708 | 0.21 | 0.16 | 6.32E-01 |
| NM_001278350 | DTYMK | 0.17 | 0.13 | 6.32E-01 |
| NM_001285501 | ERN1 | -0.23 | 0.18 | 6.32E-01 |
| NM_204669 | ALDH1A3 | -0.22 | 0.17 | 6.32E-01 |
| NM_205301 | YES1 | -0.23 | 0.18 | 6.32E-01 |
| NM_206952 | DNMT1 | -0.23 | 0.18 | 6.32E-01 |
| NM_001024827 | FOXP1 | -0.24 | 0.18 | 6.32E-01 |
| NM_001277809 | RSRC2 | 0.18 | 0.14 | 6.33E-01 |
| NM_001031506 | SLC25A32 | -0.23 | 0.18 | 6.33E-01 |
| NM_001277409 | POLR2D | 0.18 | 0.14 | 6.34E-01 |
| NM_001004403 | SLBP | 0.19 | 0.15 | 6.35E-01 |
| NM_001030589 | TWF2 | 0.10 | 0.08 | 6.36E-01 |
| NM_204781 | EPHA4 | -0.23 | 0.18 | 6.36E-01 |
| NM_001006266 | PCID2 | -0.20 | 0.16 | 6.37E-01 |
| NM_204985 | PGK1 | 0.14 | 0.11 | 6.39E-01 |
| NM_205061 | VTN | -0.18 | 0.14 | 6.39E-01 |
| NM_001006585 | INIP | 0.17 | 0.13 | 6.39E-01 |
| NM_204101 | HK1 | -0.19 | 0.15 | 6.40E-01 |
| NM_001012788 | EPN2 | -0.20 | 0.15 | 6.42E-01 |
| NM_001079497 | WRB | -0.14 | 0.11 | 6.42E-01 |
| NM_001195600 | ZSWIM8 | -0.17 | 0.13 | 6.42E-01 |
| NM_001277458 | BBS4 | -0.19 | 0.15 | 6.42E-01 |
| NM_001008465 | COMMD5 | -0.12 | 0.09 | 6.43E-01 |
| NM_204916 | OAZ1 | 0.19 | 0.15 | 6.43E-01 |
| NM_001030925 | FTSJ3 | 0.18 | 0.14 | 6.43E-01 |
| NM_001031441 | TNFAIP8 | 0.15 | 0.12 | 6.43E-01 |
| NM_001199511 | WAPAL | -0.21 | 0.16 | 6.43E-01 |
| NM_001277674 | NUDT21 | 0.16 | 0.13 | 6.43E-01 |
| NM_204451 | UNC5C | -0.19 | 0.15 | 6.43E-01 |
| NM_001197050 | CLSTN1 | -0.15 | 0.12 | 6.44E-01 |
| NM_001005821 | MRPS11 | 0.18 | 0.14 | 6.44E-01 |
| NM_001006524 | BIN1 | -0.23 | 0.18 | 6.44E-01 |
| NM_001293202 | STRA6 | -0.22 | 0.18 | 6.44E-01 |
| NM_204459 | RGS3 | -0.20 | 0.16 | 6.44E-01 |
| NR_105429 | MIR3064 | -0.17 | 0.14 | 6.44E-01 |
| NM_001006191 | TRAFD1 | 0.17 | 0.13 | 6.44E-01 |
| NM_001006475 | ERH | 0.18 | 0.14 | 6.44E-01 |
| NM_001277720 | ORC6 | 0.21 | 0.17 | 6.45E-01 |
| NM_205455 | TNS1 | 0.12 | 0.09 | 6.46E-01 |
| NM_001031150 | RAP1GDS1 | 0.13 | 0.10 | 6.46E-01 |
| NM_001006395 | MDH1 | 0.13 | 0.10 | 6.46E-01 |
| NM_001012697 | MAGI3 | -0.16 | 0.13 | 6.46E-01 |
| NM_001012790 | TASP1 | 0.15 | 0.12 | 6.46E-01 |
| NM_001199645 | KBTBD4 | -0.12 | 0.09 | 6.46E-01 |
| NM_001302133 | C7orf73 | 0.19 | 0.15 | 6.46E-01 |
| NM_204248 | SMAD6 | 0.14 | 0.11 | 6.46E-01 |
| NM_001008454 | PEX2 | -0.12 | 0.10 | 6.47E-01 |
| NM_001012536 | RHOG | -0.22 | 0.18 | 6.47E-01 |
| NM_001031227 | AP3M1 | -0.11 | 0.09 | 6.47E-01 |
| NM_001079729 | ZBTB33 | -0.22 | 0.18 | 6.47E-01 |
| NM_001278040 | RBP5 | 0.18 | 0.14 | 6.47E-01 |
| NM_204660 | CACNG3 | 0.23 | 0.18 | 6.47E-01 |
| NM_001271947 | ZCCHC6 | -0.13 | 0.10 | 6.47E-01 |
| NM_001040120 | BRI3BP | -0.23 | 0.18 | 6.48E-01 |
| NM_001257294 | SYF2 | 0.14 | 0.11 | 6.48E-01 |
| NM_001014968 | SMAD5 | -0.23 | 0.18 | 6.48E-01 |
| NM_204272 | RPL39 | 0.18 | 0.14 | 6.48E-01 |
| NM_001006533 | ZNF326 | 0.14 | 0.11 | 6.49E-01 |
| NM_205095 | MAPK9 | 0.21 | 0.17 | 6.49E-01 |
| NM_001030954 | MTSS1 | -0.23 | 0.18 | 6.50E-01 |
| NM_001006492 | ACTR10 | 0.17 | 0.14 | 6.50E-01 |
| NM_001031137 | PRIMPOL | 0.19 | 0.15 | 6.50E-01 |
| NM_205122 | PPP1CB | -0.22 | 0.17 | 6.50E-01 |
| NM_001030583 | KARS | 0.11 | 0.09 | 6.50E-01 |
| NM_001012522 | ABCC1 | -0.13 | 0.11 | 6.51E-01 |
| NM_001252255 | RPL32 | 0.18 | 0.14 | 6.51E-01 |
| NM_001277899 | AKR1B1 | 0.19 | 0.15 | 6.52E-01 |
| NM_001031341 | MYST2 | -0.12 | 0.10 | 6.52E-01 |
| NM_204585 | PARK7 | 0.18 | 0.15 | 6.52E-01 |
| NM_204164 | COL18A1 | -0.16 | 0.13 | 6.52E-01 |
| NM_001001528 | AMY1A | 0.12 | 0.10 | 6.52E-01 |
| NM_001080716 | ROR2 | -0.22 | 0.18 | 6.53E-01 |
| NM_001004377 | PAM16 | 0.17 | 0.14 | 6.53E-01 |
| NM_001199616 | SLC35E3 | 0.14 | 0.11 | 6.53E-01 |
| NM_204355 | SURF4 | -0.12 | 0.10 | 6.53E-01 |
| NM_001199711 | STXBP6 | -0.23 | 0.18 | 6.54E-01 |
| NM_001004372 | GCSH | 0.20 | 0.16 | 6.54E-01 |
| NM_001006350 | DERL1 | -0.10 | 0.08 | 6.54E-01 |
| NM_001012542 | POGLUT1 | -0.14 | 0.11 | 6.54E-01 |
| NM_001012572 | RAP2C | -0.22 | 0.17 | 6.54E-01 |
| NM_001012812 | KIAA1467 | -0.16 | 0.13 | 6.54E-01 |
| NM_001012911 | MXI1 | -0.16 | 0.13 | 6.54E-01 |
| NM_001030890 | SERINC2 | -0.17 | 0.14 | 6.54E-01 |
| NM_001278057 | TSPO | 0.16 | 0.13 | 6.54E-01 |
| NM_001031298 | CCDC101 | -0.12 | 0.09 | 6.55E-01 |
| NM_001081707 | ARPP19 | 0.12 | 0.10 | 6.55E-01 |
| NM_001031472 | SF3A1 | -0.10 | 0.08 | 6.56E-01 |
| NM_204613 | PSMA7 | 0.20 | 0.16 | 6.56E-01 |
| NM_001030579 | SLC7A5 | -0.21 | 0.17 | 6.56E-01 |
| NM_001205066 | SLC46A1 | -0.15 | 0.12 | 6.56E-01 |
| NM_204112 | LMO4 | 0.16 | 0.13 | 6.56E-01 |
| NM_204722 | TCIRG1 | -0.16 | 0.13 | 6.56E-01 |
| NM_001031292 | MIER1 | -0.19 | 0.16 | 6.56E-01 |
| NM_001039270 | ALG12 | 0.13 | 0.11 | 6.56E-01 |
| NM_205334 | COL14A1 | 0.22 | 0.18 | 6.56E-01 |
| NM_001006402 | SLC30A6 | 0.15 | 0.12 | 6.56E-01 |
| NM_001030588 | RBM6 | 0.14 | 0.11 | 6.57E-01 |
| NM_001031445 | DCTN3 | 0.17 | 0.14 | 6.58E-01 |
| NM_001305211 | ASCC3 | -0.14 | 0.11 | 6.58E-01 |
| NM_204813 | CHRNB2 | -0.21 | 0.17 | 6.58E-01 |
| NM_001006255 | HJURP | -0.17 | 0.14 | 6.59E-01 |
| NM_001030771 | PPFIBP1 | -0.12 | 0.10 | 6.59E-01 |
| NM_001277836 | VPS13A | -0.20 | 0.17 | 6.59E-01 |
| NM_001006404 | EXOC8 | -0.14 | 0.12 | 6.59E-01 |
| NM_001007871 | GID8 | -0.12 | 0.10 | 6.59E-01 |
| NM_001012836 | CPSF3L | 0.15 | 0.12 | 6.59E-01 |
| NM_001030949 | EBAG9 | 0.12 | 0.10 | 6.59E-01 |
| NM_001031326 | CLK2 | -0.15 | 0.12 | 6.59E-01 |
| NM_001302177 | RPS28 | 0.20 | 0.16 | 6.59E-01 |
| NM_001198649 | GSTK1 | 0.13 | 0.11 | 6.59E-01 |
| NM_001012595 | SCYL3 | -0.12 | 0.10 | 6.60E-01 |
| NM_204111 | UGP2 | -0.10 | 0.08 | 6.60E-01 |
| NM_001012889 | RNF4 | -0.10 | 0.08 | 6.60E-01 |
| NM_001038692 | AOX1 | -0.22 | 0.18 | 6.60E-01 |
| NM_204680 | ZBTB7A | -0.20 | 0.17 | 6.61E-01 |
| NM_001012601 | FXR1 | 0.11 | 0.09 | 6.61E-01 |
| NM_001031511 | PFKP | 0.15 | 0.13 | 6.61E-01 |
| NM_001245927 | TPM3 | 0.13 | 0.11 | 6.61E-01 |
| NM_205228 | RAB2A | 0.10 | 0.08 | 6.61E-01 |
| NM_001031140 | PKD2 | -0.21 | 0.17 | 6.61E-01 |
| NM_001012940 | NAT10 | 0.14 | 0.12 | 6.63E-01 |
| NM_001004767 | SIRT1 | -0.20 | 0.16 | 6.63E-01 |
| NM_001006491 | PSMA3 | 0.17 | 0.14 | 6.63E-01 |
| NM_001012930 | BCL6 | 0.17 | 0.14 | 6.63E-01 |
| NM_001030554 | KIF23 | -0.11 | 0.09 | 6.63E-01 |
| NM_001031567 | LPP | 0.22 | 0.18 | 6.63E-01 |
| NM_205109 | AK1 | 0.20 | 0.16 | 6.63E-01 |
| NM_001177736 | CDC14A | -0.22 | 0.18 | 6.64E-01 |
| NM_001199531 | C5H15ORF57 | 0.17 | 0.14 | 6.64E-01 |
| NM_001007829 | SNAPC5 | 0.15 | 0.13 | 6.65E-01 |
| NM_001011689 | CBFA2T2 | -0.22 | 0.18 | 6.65E-01 |
| NM_001031398 | DYM | -0.13 | 0.10 | 6.65E-01 |
| NM_001277998 | SNRPD1 | 0.20 | 0.17 | 6.65E-01 |
| NM_001293209 | NPTN | -0.10 | 0.08 | 6.65E-01 |
| NM_001305438 | EXT2 | -0.22 | 0.18 | 6.65E-01 |
| NM_001012700 | LARP4 | -0.14 | 0.12 | 6.66E-01 |
| NM_001030629 | PARN | 0.13 | 0.11 | 6.66E-01 |
| NM_001031400 | NARS | -0.16 | 0.13 | 6.66E-01 |
| NM_204808 | MTCH2 | 0.14 | 0.12 | 6.66E-01 |
| NM_204380 | TERF1 | 0.11 | 0.09 | 6.69E-01 |
| NM_204655 | U2AF1 | 0.13 | 0.10 | 6.69E-01 |
| NM_001006258 | C1H21ORF59 | 0.18 | 0.15 | 6.69E-01 |
| NM_001030879 | UBIAD1 | -0.14 | 0.11 | 6.69E-01 |
| NM_001031171 | ZDHHC13 | -0.13 | 0.11 | 6.70E-01 |
| NM_001079719 | CRLF3 | -0.14 | 0.12 | 6.70E-01 |
| NM_001006553 | NDUFB5 | 0.19 | 0.16 | 6.70E-01 |
| NM_001199396 | DZANK1 | -0.22 | 0.18 | 6.70E-01 |
| NM_205296 | H3F3B | 0.11 | 0.09 | 6.70E-01 |
| NM_001006316 | EI24 | -0.12 | 0.10 | 6.71E-01 |
| NM_001277105 | LOC407092 | -0.11 | 0.09 | 6.71E-01 |
| NM_001145914 | POP4 | 0.19 | 0.16 | 6.71E-01 |
| NM_001199453 | THBS1 | 0.21 | 0.18 | 6.71E-01 |
| NM_001113759 | CHCHD4 | 0.19 | 0.16 | 6.72E-01 |
| NM_001007933 | SLC39A9 | -0.13 | 0.11 | 6.74E-01 |
| NM_001081494 | ARL1 | 0.18 | 0.15 | 6.74E-01 |
| NR_105643 | MIR7476 | -0.19 | 0.16 | 6.74E-01 |
| NM_001004396 | RNF113A | 0.15 | 0.13 | 6.75E-01 |
| NM_001006216 | CCT6A | 0.11 | 0.09 | 6.75E-01 |
| NM_001006374 | TXNDC5 | -0.10 | 0.09 | 6.75E-01 |
| NM_001006432 | ARHGEF6 | -0.18 | 0.15 | 6.75E-01 |
| NM_001012834 | KIF3B | -0.18 | 0.16 | 6.75E-01 |
| NM_001030951 | EIF3H | -0.10 | 0.08 | 6.75E-01 |
| NM_001199538 | PIK3CG | -0.22 | 0.18 | 6.75E-01 |
| NM_001277999 | PBK | 0.17 | 0.14 | 6.75E-01 |
| NM_204593 | NR0B1 | 0.21 | 0.18 | 6.75E-01 |
| NM_204603 | SP3 | -0.20 | 0.17 | 6.75E-01 |
| NM_205376 | KTN1 | 0.14 | 0.12 | 6.75E-01 |
| NM_001004387 | EPHB6 | -0.16 | 0.13 | 6.77E-01 |
| NM_001008460 | DESI2 | -0.12 | 0.10 | 6.77E-01 |
| NM_001008458 | BRE | -0.12 | 0.10 | 6.77E-01 |
| NM_001277737 | SYAP1 | 0.11 | 0.09 | 6.77E-01 |
| NM_204265 | UBE2I | 0.14 | 0.12 | 6.77E-01 |
| NM_204450 | CREB1 | -0.20 | 0.17 | 6.77E-01 |
| NM_205111 | TSC22D1 | -0.19 | 0.16 | 6.77E-01 |
| NM_001097535 | GTF2H5 | 0.16 | 0.14 | 6.78E-01 |
| NM_001006179 | SEC23B | 0.12 | 0.10 | 6.78E-01 |
| NM_001006573 | LOC427001 | 0.14 | 0.12 | 6.78E-01 |
| NM_001030552 | CLPX | -0.10 | 0.08 | 6.78E-01 |
| NM_001030809 | NIPA2 | 0.10 | 0.09 | 6.78E-01 |
| NM_001031089 | ADI1 | -0.14 | 0.12 | 6.78E-01 |
| NM_001031257 | INHA | 0.17 | 0.14 | 6.78E-01 |
| NM_001293106 | GATA4 | -0.12 | 0.10 | 6.78E-01 |
| NM_001030915 | PM20D1 | -0.14 | 0.12 | 6.79E-01 |
| NM_001252128 | GIGYF2 | -0.12 | 0.10 | 6.79E-01 |
| NM_001006574 | FAM172A | -0.13 | 0.11 | 6.80E-01 |
| NM_001030973 | KRIT1 | -0.12 | 0.10 | 6.80E-01 |
| NM_001031246 | BARD1 | 0.18 | 0.15 | 6.80E-01 |
| NM_001031395 | ASH2L | -0.12 | 0.10 | 6.80E-01 |
| NM_001305272 | TUBA1C | 0.16 | 0.14 | 6.80E-01 |
| NM_204270 | PDGFB | 0.21 | 0.18 | 6.80E-01 |
| NM_204600 | SOCS3 | 0.21 | 0.18 | 6.80E-01 |
| NM_214674 | PCDHGC3 | -0.21 | 0.18 | 6.80E-01 |
| NR_105520 | MIR6652 | -0.18 | 0.15 | 6.80E-01 |
| NM_001006299 | WRAP73 | 0.17 | 0.14 | 6.80E-01 |
| NM_001252064 | FARP1 | -0.10 | 0.08 | 6.81E-01 |
| NM_001006361 | DFNA5 | -0.15 | 0.13 | 6.81E-01 |
| NM_001030672 | TRIM41 | -0.21 | 0.18 | 6.81E-01 |
| NM_001199411 | HIPK3 | -0.11 | 0.09 | 6.81E-01 |
| NM_205197 | MMP16 | -0.18 | 0.15 | 6.81E-01 |
| NM_001030981 | HDAC9 | -0.21 | 0.18 | 6.82E-01 |
| NM_001199445 | COG5 | -0.12 | 0.10 | 6.82E-01 |
| NM_001277663 | RPL27A | 0.17 | 0.15 | 6.82E-01 |
| NM_204507 | DHRS11 | -0.14 | 0.12 | 6.82E-01 |
| NM_204906 | KCNAB1 | -0.17 | 0.14 | 6.82E-01 |
| NM_205020 | PSMA1 | 0.16 | 0.14 | 6.82E-01 |
| NM_001030901 | NCDN | -0.11 | 0.10 | 6.83E-01 |
| NM_001204932 | APITD1 | 0.17 | 0.15 | 6.83E-01 |
| NM_001199450 | ZC3H6 | -0.10 | 0.09 | 6.85E-01 |
| NM_204891 | ATP2A3 | -0.21 | 0.18 | 6.85E-01 |
| NM_001004429 | TACC3 | 0.11 | 0.09 | 6.86E-01 |
| NM_001006276 | RFC3 | 0.15 | 0.13 | 6.87E-01 |
| NM_001007875 | SSU72 | 0.14 | 0.12 | 6.87E-01 |
| NM_001030740 | NELL2 | -0.18 | 0.16 | 6.87E-01 |
| NM_001039297 | INTS10 | 0.16 | 0.14 | 6.87E-01 |
| NM_001098606 | ADCYAP1R1 | 0.20 | 0.17 | 6.87E-01 |
| NM_001302135 | GNG5 | 0.18 | 0.16 | 6.87E-01 |
| NM_001302182 | C17orf89 | 0.16 | 0.14 | 6.87E-01 |
| NM_001006506 | BUB3 | 0.15 | 0.13 | 6.87E-01 |
| NM_001012603 | GINS1 | 0.17 | 0.14 | 6.87E-01 |
| NM_001030616 | THG1L | 0.17 | 0.14 | 6.87E-01 |
| NM_001031403 | SLC48A1 | 0.12 | 0.11 | 6.87E-01 |
| NM_001031475 | ARRDC1 | -0.15 | 0.13 | 6.87E-01 |
| NM_001044690 | ASF1A | 0.15 | 0.13 | 6.87E-01 |
| NM_001198650 | MRPS33 | 0.16 | 0.14 | 6.87E-01 |
| NM_001199628 | HACL1 | -0.20 | 0.18 | 6.87E-01 |
| NM_001277535 | SIVA1 | 0.18 | 0.16 | 6.87E-01 |
| NM_001293166 | SNRPC | 0.16 | 0.14 | 6.87E-01 |
| NM_001293311 | ATP6V0D1 | -0.10 | 0.09 | 6.87E-01 |
| NM_204715 | FURIN | -0.17 | 0.15 | 6.87E-01 |
| NM_204968 | PTPN11 | -0.16 | 0.14 | 6.87E-01 |
| NM_205016 | RAC3 | -0.12 | 0.10 | 6.87E-01 |
| NM_205064 | SOD1 | 0.15 | 0.13 | 6.87E-01 |
| NM_205274 | MYH11 | -0.15 | 0.13 | 6.87E-01 |
| NM_001004400 | TUBB2B | 0.13 | 0.12 | 6.87E-01 |
| NM_001006253 | PCNP | 0.11 | 0.10 | 6.87E-01 |
| NM_001006269 | ELF1 | -0.15 | 0.13 | 6.87E-01 |
| NM_001030862 | RPRD1B | -0.12 | 0.10 | 6.87E-01 |
| NM_001030933 | SPPL2B | -0.20 | 0.17 | 6.87E-01 |
| NM_001031056 | AARS2 | -0.13 | 0.11 | 6.87E-01 |
| NM_001031059 | NANP | -0.11 | 0.10 | 6.87E-01 |
| NM_001097529 | FCRL2 | -0.16 | 0.14 | 6.87E-01 |
| NM_001199516 | EXOSC3 | 0.19 | 0.16 | 6.87E-01 |
| NM_001257341 | SCRN3 | 0.10 | 0.09 | 6.87E-01 |
| NM_001277553 | GRAP | 0.18 | 0.16 | 6.87E-01 |
| NM_001012810 | UBE2N | 0.14 | 0.12 | 6.88E-01 |
| NM_001012914 | STAT1 | -0.14 | 0.12 | 6.88E-01 |
| NM_001031215 | CAT | -0.10 | 0.09 | 6.88E-01 |
| NM_001040413 | PTRH2 | 0.18 | 0.15 | 6.88E-01 |
| NM_001167735 | EIF1 | 0.15 | 0.13 | 6.88E-01 |
| NM_204144 | TBX20 | -0.21 | 0.18 | 6.88E-01 |
| NM_204282 | ID4 | 0.20 | 0.17 | 6.88E-01 |
| NM_001006566 | QDPR | 0.11 | 0.09 | 6.90E-01 |
| NM_001030957 | RP4-613B23.5 | 0.13 | 0.12 | 6.91E-01 |
| NM_001199500 | WASL | -0.12 | 0.11 | 6.92E-01 |
| NM_001006302 | SDF4 | 0.10 | 0.09 | 6.93E-01 |
| NM_001030607 | RARS | 0.11 | 0.10 | 6.93E-01 |
| NM_001031139 | USO1 | -0.09 | 0.08 | 6.93E-01 |
| NM_001044688 | LOC693265 | 0.12 | 0.10 | 6.93E-01 |
| NM_001277514 | ZMAT5 | 0.18 | 0.16 | 6.93E-01 |
| NM_204170 | PCNA | 0.12 | 0.11 | 6.93E-01 |
| NM_001101037 | CENPA | -0.19 | 0.17 | 6.93E-01 |
| NM_001030703 | JMJD6 | -0.15 | 0.13 | 6.93E-01 |
| NM_001031045 | TGFB2 | 0.16 | 0.14 | 6.93E-01 |
| NM_001198668 | FDX1 | 0.12 | 0.11 | 6.93E-01 |
| NM_001278117 | RINT1 | 0.15 | 0.13 | 6.93E-01 |
| NM_001001305 | SRSF2 | -0.12 | 0.10 | 6.94E-01 |
| NM_001001613 | CD74 | -0.19 | 0.17 | 6.94E-01 |
| NM_001006153 | CTD-2410N18.5 | -0.13 | 0.11 | 6.94E-01 |
| NM_001006164 | PEMT | -0.14 | 0.13 | 6.94E-01 |
| NM_001006392 | CEPT1 | -0.11 | 0.10 | 6.94E-01 |
| NM_001006436 | NXT2 | -0.16 | 0.14 | 6.94E-01 |
| NM_001006503 | CUTC | 0.14 | 0.12 | 6.94E-01 |
| NM_001006581 | RIOK2 | 0.11 | 0.10 | 6.94E-01 |
| NM_001007855 | FGFR1OP2 | -0.10 | 0.09 | 6.94E-01 |
| NM_001007858 | GABPA | -0.09 | 0.08 | 6.94E-01 |
| NM_001008448 | OSBPL2 | -0.18 | 0.16 | 6.94E-01 |
| NM_001012521 | CELF1 | -0.14 | 0.12 | 6.94E-01 |
| NM_001012879 | TMEM30A | 0.09 | 0.08 | 6.94E-01 |
| NM_001012938 | ZC3HAV1 | -0.15 | 0.13 | 6.94E-01 |
| NM_001030605 | IRAK2 | -0.13 | 0.12 | 6.94E-01 |
| NM_001030665 | TFIP11 | -0.11 | 0.10 | 6.94E-01 |
| NM_001030704 | MMD | 0.14 | 0.12 | 6.94E-01 |
| NM_001030986 | SNX10 | 0.16 | 0.14 | 6.94E-01 |
| NM_001031134 | C4H4ORF29 | 0.18 | 0.16 | 6.94E-01 |
| NM_001031143 | HNRNPD | 0.12 | 0.11 | 6.94E-01 |
| NM_001031365 | CAPRIN1 | -0.20 | 0.18 | 6.94E-01 |
| NM_001031373 | UBQLN4 | -0.10 | 0.09 | 6.94E-01 |
| NM_001031490 | TBC1D22A | -0.10 | 0.09 | 6.94E-01 |
| NM_001031524 | MPC1 | 0.13 | 0.12 | 6.94E-01 |
| NM_001031549 | GPHN | 0.15 | 0.14 | 6.94E-01 |
| NM_001031562 | HIAT1 | -0.13 | 0.11 | 6.94E-01 |
| NM_001031589 | GCC1 | -0.15 | 0.14 | 6.94E-01 |
| NM_001031773 | EFHD1 | -0.19 | 0.17 | 6.94E-01 |
| NM_001039269 | KCTD7 | -0.16 | 0.14 | 6.94E-01 |
| NM_001039293 | ELP3 | 0.14 | 0.12 | 6.94E-01 |
| NM_001044670 | CENPN | 0.17 | 0.15 | 6.94E-01 |
| NM_001079744 | WDR92 | -0.14 | 0.12 | 6.94E-01 |
| NM_001080103 | TNFRSF19 | -0.20 | 0.18 | 6.94E-01 |
| NM_001081506 | TICAM1 | -0.13 | 0.12 | 6.94E-01 |
| NM_001105319 | MCPH1 | 0.17 | 0.15 | 6.94E-01 |
| NM_001114852 | SCOC | 0.11 | 0.10 | 6.94E-01 |
| NM_001167729 | TBCA | 0.16 | 0.15 | 6.94E-01 |
| NM_001167737 | DYNC1LI1 | 0.13 | 0.12 | 6.94E-01 |
| NM_001199532 | REEP3 | -0.18 | 0.16 | 6.94E-01 |
| NM_001201468 | PON2 | -0.16 | 0.14 | 6.94E-01 |
| NM_001252080 | ABCC6 | -0.18 | 0.16 | 6.94E-01 |
| NM_001256801 | ELMO3 | -0.16 | 0.14 | 6.94E-01 |
| NM_001276324 | ERP29 | 0.12 | 0.11 | 6.94E-01 |
| NM_001277395 | ECHS1 | 0.15 | 0.13 | 6.94E-01 |
| NM_001277762 | TEX30 | 0.19 | 0.17 | 6.94E-01 |
| NM_001277911 | RPS12 | 0.17 | 0.15 | 6.94E-01 |
| NM_001292069 | PSMC5 | 0.14 | 0.13 | 6.94E-01 |
| NM_001302157 | COX14 | 0.19 | 0.17 | 6.94E-01 |
| NM_001305114 | ARL6 | 0.17 | 0.15 | 6.94E-01 |
| NM_204361 | KIT | 0.19 | 0.17 | 6.94E-01 |
| NM_204436 | NT5C3A | 0.12 | 0.11 | 6.94E-01 |
| NM_204515 | IL17RD | 0.20 | 0.17 | 6.94E-01 |
| NM_204529 | SH3GL1 | -0.20 | 0.18 | 6.94E-01 |
| NM_204534 | RARRES1 | -0.17 | 0.15 | 6.94E-01 |
| NM_204539 | TMEM121 | -0.14 | 0.12 | 6.94E-01 |
| NM_204550 | DYRK1A | -0.20 | 0.18 | 6.94E-01 |
| NM_204559 | MSX2 | 0.19 | 0.17 | 6.94E-01 |
| NM_204586 | GJA1 | -0.20 | 0.18 | 6.94E-01 |
| NM_204801 | TSPAN18 | 0.18 | 0.16 | 6.94E-01 |
| NM_204803 | MEIS2 | -0.18 | 0.16 | 6.94E-01 |
| NM_205055 | AKT1 | -0.19 | 0.17 | 6.94E-01 |
| NM_205057 | RABEP1 | 0.12 | 0.11 | 6.94E-01 |
| NM_205108 | RPS4X | 0.16 | 0.14 | 6.94E-01 |
| NM_205332 | CTCF | -0.10 | 0.09 | 6.94E-01 |
| NM_205405 | C3 | 0.20 | 0.18 | 6.94E-01 |
| NM_205505 | ACACA | -0.21 | 0.18 | 6.94E-01 |
| NM_206865 | POPDC2 | 0.11 | 0.10 | 6.94E-01 |
| NM_001005839 | BRD7 | 0.13 | 0.12 | 6.94E-01 |
| NM_001007988 | BMI1 | -0.18 | 0.16 | 6.94E-01 |
| NM_001030768 | BPGM | -0.18 | 0.16 | 6.94E-01 |
| NM_001031058 | YIPF4 | 0.10 | 0.09 | 6.94E-01 |
| NM_001031149 | PDLIM5 | -0.13 | 0.12 | 6.94E-01 |
| NM_001079731 | ADCK1 | 0.14 | 0.13 | 6.94E-01 |
| NM_001114623 | FIG4 | -0.11 | 0.10 | 6.94E-01 |
| NM_001277540 | AIDA | -0.12 | 0.11 | 6.94E-01 |
| NM_204742 | MKRN1 | -0.10 | 0.09 | 6.94E-01 |
| NM_001030841 | TAF3 | -0.11 | 0.10 | 6.94E-01 |
| NM_001031259 | RNF25 | -0.19 | 0.17 | 6.94E-01 |
| NM_001006162 | WIPI2 | -0.11 | 0.10 | 6.95E-01 |
| NM_001012906 | Mar-05 | -0.14 | 0.13 | 6.95E-01 |
| NM_001287187 | ANKRD26 | 0.12 | 0.11 | 6.95E-01 |
| NM_204767 | OLFM1 | 0.17 | 0.16 | 6.95E-01 |
| NM_205035 | EFNB1 | -0.13 | 0.12 | 6.95E-01 |
| NM_001006206 | GPS1 | 0.09 | 0.08 | 6.95E-01 |
| NM_001030708 | FAM104A | -0.11 | 0.10 | 6.97E-01 |
| NM_204905 | PCBD1 | 0.15 | 0.13 | 6.97E-01 |
| NM_001031229 | ACTA2 | 0.16 | 0.14 | 6.98E-01 |
| NM_001037827 | VEZF1 | -0.20 | 0.18 | 6.98E-01 |
| NM_001006484 | TTC7B | 0.15 | 0.14 | 6.99E-01 |
| NM_001007845 | PRKAR1A | -0.11 | 0.10 | 6.99E-01 |
| NM_001010841 | NGEF | -0.20 | 0.18 | 6.99E-01 |
| NM_001012523 | TAOK3 | -0.14 | 0.12 | 6.99E-01 |
| NM_001012526 | NUP93 | 0.09 | 0.09 | 6.99E-01 |
| NM_001012785 | TCOF1 | 0.16 | 0.14 | 6.99E-01 |
| NM_001030911 | PAFAH1B2 | -0.11 | 0.10 | 6.99E-01 |
| NM_001031151 | METAP1 | -0.15 | 0.14 | 6.99E-01 |
| NM_001031430 | SYK | -0.16 | 0.15 | 6.99E-01 |
| NM_001031447 | TARS | 0.10 | 0.09 | 6.99E-01 |
| NM_001034823 | ERCC5 | 0.15 | 0.14 | 6.99E-01 |
| NM_001079761 | BRAP | -0.13 | 0.12 | 6.99E-01 |
| NM_001195595 | C22H2ORF42 | -0.18 | 0.17 | 6.99E-01 |
| NM_001199420 | MPPED2 | -0.15 | 0.14 | 6.99E-01 |
| NM_001199499 | ZNF800 | -0.16 | 0.15 | 6.99E-01 |
| NM_001201448 | MICALL1 | 0.13 | 0.12 | 6.99E-01 |
| NM_001252029 | USMG5 | 0.17 | 0.16 | 6.99E-01 |
| NM_001277422 | RFTN2 | 0.15 | 0.13 | 6.99E-01 |
| NM_001277552 | PALMD | -0.19 | 0.18 | 6.99E-01 |
| NM_001277591 | CLUAP1 | 0.14 | 0.13 | 6.99E-01 |
| NM_001277603 | TSPAN6 | 0.12 | 0.11 | 6.99E-01 |
| NM_001278005 | SYS1 | 0.18 | 0.16 | 6.99E-01 |
| NM_001293280 | SHC1 | -0.13 | 0.12 | 6.99E-01 |
| NM_204237 | TLE4 | -0.12 | 0.11 | 6.99E-01 |
| NM_204260 | CELF2 | -0.18 | 0.17 | 6.99E-01 |
| NM_204315 | RP11-290H9.2 | -0.16 | 0.15 | 6.99E-01 |
| NM_204340 | TNKS | -0.20 | 0.18 | 6.99E-01 |
| NM_204390 | SGMS1 | -0.14 | 0.13 | 6.99E-01 |
| NM_204489 | MRAS | 0.11 | 0.10 | 6.99E-01 |
| NM_204783 | WNT4 | 0.18 | 0.16 | 6.99E-01 |
| NM_205051 | CENPC | 0.12 | 0.11 | 6.99E-01 |
| NM_205297 | SPCS3 | 0.14 | 0.13 | 6.99E-01 |
| NM_001031283 | EPS15 | -0.15 | 0.13 | 6.99E-01 |
| NM_001001777 | GSTA3 | 0.16 | 0.15 | 7.01E-01 |
| NM_001006237 | RANGAP1 | -0.12 | 0.11 | 7.01E-01 |
| NM_001031043 | ACBD3 | 0.11 | 0.10 | 7.01E-01 |
| NM_001039255 | SMARCB1 | 0.11 | 0.10 | 7.01E-01 |
| NM_001079482 | PPP2R5C | 0.11 | 0.11 | 7.01E-01 |
| NM_001197057 | COX16 | 0.18 | 0.17 | 7.01E-01 |
| NM_001199408 | TMX4 | -0.11 | 0.11 | 7.01E-01 |
| NM_001252140 | NBEA | -0.18 | 0.17 | 7.01E-01 |
| NM_001277338 | H2AFY2 | 0.18 | 0.17 | 7.01E-01 |
| NM_204244 | CRY2 | -0.20 | 0.18 | 7.01E-01 |
| NM_204784 | WNT11 | 0.15 | 0.14 | 7.01E-01 |
| NM_001012776 | WDR59 | -0.13 | 0.12 | 7.02E-01 |
| NM_001012955 | FOXM1 | -0.11 | 0.10 | 7.02E-01 |
| NM_001030565 | C11H16ORF70 | -0.11 | 0.11 | 7.02E-01 |
| NM_001031086 | SLC17A5 | -0.15 | 0.14 | 7.02E-01 |
| NM_001039911 | B4GALT7 | 0.17 | 0.16 | 7.02E-01 |
| NM_001079485 | HMGN2 | 0.12 | 0.12 | 7.02E-01 |
| NM_001079575 | NTHL1 | -0.19 | 0.18 | 7.02E-01 |
| NM_001195603 | FOCAD | -0.11 | 0.10 | 7.02E-01 |
| NM_204980 | CHRD | -0.13 | 0.13 | 7.02E-01 |
| NM_205462 | RPS15 | 0.14 | 0.13 | 7.02E-01 |
| NM_001006372 | DTNBP1 | 0.13 | 0.12 | 7.02E-01 |
| NM_001109771 | RPS6KA1 | -0.10 | 0.09 | 7.03E-01 |
| NM_001006425 | HN1 | 0.13 | 0.12 | 7.04E-01 |
| NM_001257283 | MCL1 | -0.11 | 0.10 | 7.04E-01 |
| NM_001006163 | COPS3 | 0.10 | 0.09 | 7.04E-01 |
| NM_001030720 | SLC47A1 | -0.14 | 0.13 | 7.04E-01 |
| NM_001031181 | WEE1 | 0.11 | 0.10 | 7.04E-01 |
| NM_001257333 | RPL9 | 0.12 | 0.11 | 7.04E-01 |
| NM_204255 | RIMBP2 | -0.19 | 0.18 | 7.04E-01 |
| NM_001271991 | SMIM12 | -0.16 | 0.15 | 7.04E-01 |
| NM_001031509 | MELK | 0.13 | 0.12 | 7.05E-01 |
| NM_001006386 | LPIN2 | -0.14 | 0.13 | 7.05E-01 |
| NM_001031339 | NCKAP1L | -0.18 | 0.17 | 7.05E-01 |
| NM_001031436 | BX255923.2 | 0.13 | 0.12 | 7.05E-01 |
| NM_001006336 | MVP | 0.15 | 0.14 | 7.06E-01 |
| NM_001006355 | RAB18 | 0.10 | 0.09 | 7.06E-01 |
| NM_001006358 | BZW2 | 0.11 | 0.11 | 7.06E-01 |
| NM_001006375 | SSR1 | 0.12 | 0.11 | 7.06E-01 |
| NM_001006381 | GALNT1 | -0.11 | 0.10 | 7.06E-01 |
| NM_001007937 | LIN52 | -0.16 | 0.15 | 7.06E-01 |
| NM_001012548 | C26H6ORF89 | -0.09 | 0.08 | 7.06E-01 |
| NM_001012865 | FAM65B | 0.19 | 0.18 | 7.06E-01 |
| NM_001030929 | RPL19 | 0.14 | 0.13 | 7.06E-01 |
| NM_001031177 | TALDO1 | 0.14 | 0.13 | 7.06E-01 |
| NM_001031465 | ZMAT2 | 0.13 | 0.12 | 7.06E-01 |
| NM_001031595 | IKZF5 | -0.16 | 0.15 | 7.06E-01 |
| NM_001171886 | LAMTOR5 | 0.13 | 0.12 | 7.06E-01 |
| NM_001199194 | CLSPN | 0.14 | 0.13 | 7.06E-01 |
| NM_001199424 | MTIF2 | 0.13 | 0.12 | 7.06E-01 |
| NM_001199518 | MRPL21 | 0.17 | 0.17 | 7.06E-01 |
| NM_001257269 | RUFY1 | -0.10 | 0.09 | 7.06E-01 |
| NM_001257295 | BCAS2 | 0.14 | 0.13 | 7.06E-01 |
| NM_001287193 | LRRK2 | -0.17 | 0.16 | 7.06E-01 |
| NM_204200 | ARNT | -0.14 | 0.13 | 7.06E-01 |
| NM_204587 | MYH7B | 0.15 | 0.14 | 7.06E-01 |
| NM_204645 | CENPF | 0.13 | 0.13 | 7.06E-01 |
| NM_205225 | RPS6 | 0.14 | 0.13 | 7.06E-01 |
| NM_205451 | TPI1 | 0.14 | 0.14 | 7.06E-01 |
| NM_001199582 | USP47 | -0.12 | 0.12 | 7.06E-01 |
| NM_001199507 | BICC1 | -0.18 | 0.17 | 7.07E-01 |
| NM_001006160 | NUDT1 | 0.15 | 0.14 | 7.08E-01 |
| NM_001033941 | FANCL | 0.11 | 0.11 | 7.08E-01 |
| NM_204194 | ILK | 0.08 | 0.08 | 7.08E-01 |
| NM_001030735 | DNAJB9 | 0.13 | 0.13 | 7.09E-01 |
| NM_204374 | STK25 | -0.18 | 0.17 | 7.10E-01 |
| NM_213573 | ATG4B | -0.19 | 0.18 | 7.10E-01 |
| NM_205465 | PRNP | -0.13 | 0.12 | 7.11E-01 |
| NM_204966 | HAND2 | 0.15 | 0.15 | 7.11E-01 |
| NM_204388 | FAM53A | -0.13 | 0.12 | 7.12E-01 |
| NM_001079725 | DDX10 | 0.14 | 0.14 | 7.12E-01 |
| NM_001143931 | TPCN3 | 0.15 | 0.15 | 7.12E-01 |
| NM_001277640 | CETN2 | 0.14 | 0.13 | 7.12E-01 |
| NM_204579 | FMO6P | 0.19 | 0.18 | 7.12E-01 |
| NM_205461 | PPIB | 0.15 | 0.15 | 7.12E-01 |
| NM_001030295 | NOTCH1 | -0.12 | 0.12 | 7.13E-01 |
| NM_001293232 | KANK1 | -0.13 | 0.13 | 7.13E-01 |
| NM_204372 | ARSH | 0.14 | 0.13 | 7.13E-01 |
| NM_205217 | ST3GAL1 | 0.17 | 0.17 | 7.13E-01 |
| NM_001031492 | PMEPA1 | 0.13 | 0.12 | 7.13E-01 |
| NM_001243590 | AKR1B1L | 0.12 | 0.11 | 7.13E-01 |
| NM_001195569 | SUGP2 | -0.17 | 0.16 | 7.14E-01 |
| NM_001030663 | HVCN1 | -0.10 | 0.10 | 7.15E-01 |
| NM_001031118 | FAM122A | -0.09 | 0.09 | 7.15E-01 |
| NM_001199751 | RNF111 | -0.10 | 0.09 | 7.15E-01 |
| NM_001276283 | FZD9 | 0.12 | 0.12 | 7.15E-01 |
| NM_204396 | CDKN1A | -0.19 | 0.18 | 7.15E-01 |
| NM_204802 | DCTN2 | -0.13 | 0.13 | 7.15E-01 |
| NM_205106 | HMGN1 | 0.14 | 0.14 | 7.15E-01 |
| NM_205126 | ACTN4 | -0.19 | 0.18 | 7.15E-01 |
| NM_001012537 | FBXO7 | -0.10 | 0.10 | 7.15E-01 |
| NM_001006235 | TMPO | 0.08 | 0.07 | 7.15E-01 |
| NM_001031144 | FAM175A | -0.13 | 0.13 | 7.15E-01 |
| NM_001001469 | GART | -0.11 | 0.11 | 7.16E-01 |
| NM_001012792 | IFT140 | 0.18 | 0.18 | 7.16E-01 |
| NM_001030870 | NADK | -0.11 | 0.11 | 7.16E-01 |
| NM_001031083 | SYNCRIP | -0.11 | 0.11 | 7.16E-01 |
| NM_001006197 | PMPCA | -0.12 | 0.11 | 7.17E-01 |
| NM_001007837 | VPS29 | 0.11 | 0.10 | 7.17E-01 |
| NM_001012760 | TSC22D1 | -0.10 | 0.10 | 7.17E-01 |
| NM_001030548 | PRTG | -0.17 | 0.17 | 7.17E-01 |
| NM_001030644 | CLCN7 | -0.09 | 0.09 | 7.17E-01 |
| NM_001031100 | INTS9 | -0.10 | 0.10 | 7.17E-01 |
| NM_001031405 | COPA | -0.11 | 0.11 | 7.17E-01 |
| NM_001172662 | RAB40C | 0.17 | 0.16 | 7.17E-01 |
| NM_001199541 | ZBED4 | -0.16 | 0.16 | 7.17E-01 |
| NM_001278022 | IRAK1BP1 | 0.18 | 0.17 | 7.17E-01 |
| NM_001305188 | RPL11 | 0.15 | 0.15 | 7.17E-01 |
| NM_204377 | CSNK1E | -0.17 | 0.17 | 7.17E-01 |
| NM_204605 | F2 | -0.15 | 0.15 | 7.17E-01 |
| NM_204727 | PPP1R12B | -0.19 | 0.18 | 7.17E-01 |
| NM_204809 | SYNM | -0.12 | 0.12 | 7.17E-01 |
| NM_204852 | RBBP4 | -0.10 | 0.09 | 7.17E-01 |
| NM_204874 | LSAMP | 0.18 | 0.18 | 7.17E-01 |
| NM_205369 | C5H14ORF166 | 0.14 | 0.14 | 7.17E-01 |
| NM_001030600 | SLC6A6 | -0.18 | 0.17 | 7.18E-01 |
| NM_001031581 | CANT1 | -0.13 | 0.13 | 7.19E-01 |
| NM_001031593 | VPS45 | -0.11 | 0.11 | 7.19E-01 |
| NM_001044640 | RAB6A | 0.13 | 0.12 | 7.19E-01 |
| NM_204285 | DFFB | -0.13 | 0.13 | 7.19E-01 |
| NM_001008676 | IFNGR2 | -0.10 | 0.10 | 7.20E-01 |
| NM_001031063 | ACTA1 | 0.17 | 0.17 | 7.20E-01 |
| NM_001006208 | RHOT1 | -0.10 | 0.10 | 7.20E-01 |
| NM_001006260 | EIF2S3L | -0.08 | 0.08 | 7.20E-01 |
| NM_001006323 | SLC16A1 | -0.13 | 0.13 | 7.20E-01 |
| NM_001012809 | TBC1D15 | -0.09 | 0.09 | 7.20E-01 |
| NM_001030372 | TEC | -0.15 | 0.15 | 7.20E-01 |
| NM_001031097 | RAB10 | -0.10 | 0.10 | 7.20E-01 |
| NM_001031384 | SUPT5H | -0.10 | 0.09 | 7.20E-01 |
| NM_001114972 | SEPN1 | -0.18 | 0.18 | 7.20E-01 |
| NM_001271894 | PODXL | -0.11 | 0.10 | 7.20E-01 |
| NM_001278010 | C3H6ORF203 | 0.14 | 0.14 | 7.20E-01 |
| NM_001291966 | PCYT2 | -0.11 | 0.11 | 7.20E-01 |
| NM_204582 | GJD2 | 0.18 | 0.18 | 7.20E-01 |
| NM_204651 | ADRM1 | 0.11 | 0.11 | 7.20E-01 |
| NM_204751 | RDX | -0.12 | 0.12 | 7.20E-01 |
| NM_205128 | OCLN | -0.15 | 0.14 | 7.20E-01 |
| NM_205275 | MT4 | 0.17 | 0.17 | 7.20E-01 |
| NM_205380 | COL3A1 | 0.15 | 0.15 | 7.20E-01 |
| NM_001030728 | NAMPT | -0.17 | 0.17 | 7.21E-01 |
| NM_204492 | PTPRU | -0.18 | 0.18 | 7.21E-01 |
| NM_001006293 | DDX27 | 0.13 | 0.13 | 7.21E-01 |
| NM_001044642 | ZBTB48 | -0.13 | 0.13 | 7.21E-01 |
| NM_001080862 | NCAPD2 | -0.08 | 0.08 | 7.21E-01 |
| NM_001080872 | SALL4 | -0.11 | 0.11 | 7.21E-01 |
| NM_001277519 | GTF2H3 | 0.12 | 0.12 | 7.21E-01 |
| NM_001277594 | FIBIN | 0.12 | 0.12 | 7.21E-01 |
| NM_204230 | KHDRBS1 | -0.10 | 0.10 | 7.21E-01 |
| NM_001001293 | GHR | -0.18 | 0.18 | 7.22E-01 |
| NM_001006147 | HSPA9 | 0.11 | 0.11 | 7.22E-01 |
| NM_001006349 | EIF3E | -0.10 | 0.10 | 7.22E-01 |
| NM_001006458 | GRPEL1 | 0.11 | 0.11 | 7.22E-01 |
| NM_001030803 | SMS | 0.08 | 0.08 | 7.22E-01 |
| NM_001030839 | UVRAG | -0.18 | 0.18 | 7.22E-01 |
| NM_001031122 | CHIC1 | 0.14 | 0.14 | 7.22E-01 |
| NM_001031289 | JUN | 0.17 | 0.17 | 7.22E-01 |
| NM_001031303 | KLHL6 | -0.12 | 0.12 | 7.22E-01 |
| NM_001031379 | SGTA | 0.09 | 0.09 | 7.22E-01 |
| NM_001079764 | MTO1 | -0.14 | 0.14 | 7.22E-01 |
| NM_001098238 | THOC7 | 0.15 | 0.15 | 7.22E-01 |
| NM_001199585 | PRTFDC1 | -0.18 | 0.18 | 7.22E-01 |
| NM_001277439 | ATP6V1D | 0.13 | 0.13 | 7.22E-01 |
| NM_204169 | BRCA1 | -0.14 | 0.14 | 7.22E-01 |
| NM_001099860 | EIF4G2 | -0.18 | 0.18 | 7.22E-01 |
| NM_204923 | PPP1R9B | 0.11 | 0.11 | 7.22E-01 |
| NM_001177322 | CACNA2D1 | -0.13 | 0.13 | 7.23E-01 |
| NM_001305150 | GDPD5 | -0.14 | 0.14 | 7.23E-01 |
| NM_204140 | RPL36 | 0.16 | 0.16 | 7.23E-01 |
| NM_205229 | VLDLR | -0.14 | 0.14 | 7.24E-01 |
| NM_001006496 | TUBGCP2 | -0.09 | 0.09 | 7.24E-01 |
| NM_001012691 | CSNK2A2 | -0.11 | 0.11 | 7.24E-01 |
| NM_001030784 | TBC1D23 | 0.10 | 0.10 | 7.24E-01 |
| NM_001030952 | MYC | 0.10 | 0.11 | 7.24E-01 |
| NM_001031002 | KDSR | -0.17 | 0.17 | 7.24E-01 |
| NM_001031044 | DUSP10 | 0.13 | 0.13 | 7.24E-01 |
| NM_001031170 | RPIA | -0.12 | 0.12 | 7.24E-01 |
| NM_001031194 | VPS39 | -0.12 | 0.12 | 7.24E-01 |
| NM_001031235 | TAF5 | -0.13 | 0.13 | 7.24E-01 |
| NM_001031256 | PSMD14 | 0.12 | 0.12 | 7.24E-01 |
| NM_001031375 | DNAJB14 | 0.09 | 0.09 | 7.24E-01 |
| NM_001031443 | COMMD10 | 0.16 | 0.16 | 7.24E-01 |
| NM_001031519 | ESF1 | 0.15 | 0.15 | 7.24E-01 |
| NM_001079503 | YEATS4 | 0.15 | 0.15 | 7.24E-01 |
| NM_001146332 | NDUFS3 | 0.15 | 0.15 | 7.24E-01 |
| NM_001162907 | ULK3 | -0.10 | 0.10 | 7.24E-01 |
| NM_001199422 | METTL15 | 0.14 | 0.14 | 7.24E-01 |
| NM_001199605 | MON2 | -0.13 | 0.13 | 7.24E-01 |
| NM_001252314 | TBC1D14 | -0.17 | 0.17 | 7.24E-01 |
| NM_001277660 | IDS | 0.11 | 0.12 | 7.24E-01 |
| NM_001282330 | CCDC28B | 0.14 | 0.14 | 7.24E-01 |
| NM_001302195 | TIMM13 | 0.15 | 0.16 | 7.24E-01 |
| NM_204748 | P2RX5 | -0.14 | 0.14 | 7.24E-01 |
| NM_204777 | FGF14 | -0.16 | 0.16 | 7.24E-01 |
| NM_204843 | RAP1GAP | 0.10 | 0.10 | 7.24E-01 |
| NM_207179 | AKAP9 | 0.13 | 0.13 | 7.24E-01 |
| NM_001006456 | RFC1 | 0.12 | 0.12 | 7.24E-01 |
| NM_001198647 | MRPS17 | 0.14 | 0.14 | 7.24E-01 |
| NM_204627 | TYRO3 | -0.11 | 0.11 | 7.24E-01 |
| NM_001030681 | NACC2 | -0.15 | 0.15 | 7.26E-01 |
| NM_001012838 | EMC1 | 0.10 | 0.10 | 7.26E-01 |
| NM_001007826 | ABHD17C | -0.12 | 0.12 | 7.27E-01 |
| NM_001031157 | PI4K2B | -0.16 | 0.16 | 7.27E-01 |
| NM_001278726 | JMJD8 | 0.11 | 0.11 | 7.27E-01 |
| NM_204249 | GNAI3 | -0.11 | 0.11 | 7.27E-01 |
| NM_001030638 | PGP | -0.11 | 0.12 | 7.27E-01 |
| NM_001122763 | RPL37 | 0.13 | 0.14 | 7.27E-01 |
| NM_001302151 | PKIG | -0.11 | 0.11 | 7.27E-01 |
| NM_001031088 | TSSC1 | -0.11 | 0.12 | 7.28E-01 |
| NM_001031415 | ARSK | -0.15 | 0.15 | 7.28E-01 |
| NM_001277572 | ATP6V0B | 0.14 | 0.14 | 7.28E-01 |
| NM_205138 | FOXC2 | -0.16 | 0.16 | 7.28E-01 |
| NM_001012696 | PIK3CD | -0.17 | 0.17 | 7.28E-01 |
| NM_001030931 | STAT3 | -0.18 | 0.18 | 7.28E-01 |
| NM_001195554 | SRSF3 | 0.13 | 0.13 | 7.28E-01 |
| NM_001004378 | GNB2L1 | -0.11 | 0.12 | 7.29E-01 |
| NM_001006352 | ARF1 | 0.08 | 0.08 | 7.29E-01 |
| NM_001006501 | ADK | -0.12 | 0.12 | 7.29E-01 |
| NM_001006529 | PKP4 | -0.11 | 0.12 | 7.29E-01 |
| NM_001008682 | ADAM17 | -0.10 | 0.11 | 7.29E-01 |
| NM_001030008 | AP1S3 | 0.17 | 0.18 | 7.29E-01 |
| NM_001030820 | NDFIP2 | -0.12 | 0.12 | 7.29E-01 |
| NM_001030894 | TAF12 | 0.13 | 0.13 | 7.29E-01 |
| NM_001030958 | IBA57 | -0.11 | 0.12 | 7.29E-01 |
| NM_001031009 | STX17 | -0.16 | 0.17 | 7.29E-01 |
| NM_001031399 | LMAN1 | 0.12 | 0.12 | 7.29E-01 |
| NM_001031424 | ZFAND5 | -0.09 | 0.09 | 7.29E-01 |
| NM_001031432 | GNE | -0.11 | 0.11 | 7.29E-01 |
| NM_001031456 | PIAS1 | -0.17 | 0.18 | 7.29E-01 |
| NM_001031561 | ARPC5 | 0.09 | 0.10 | 7.29E-01 |
| NM_001038691 | ZDHHC8 | -0.18 | 0.18 | 7.29E-01 |
| NM_001110364 | CALM1 | 0.08 | 0.08 | 7.29E-01 |
| NM_001168004 | EPB41 | -0.16 | 0.17 | 7.29E-01 |
| NM_001197144 | CCM2 | -0.18 | 0.18 | 7.29E-01 |
| NM_001199428 | MALL | -0.15 | 0.15 | 7.29E-01 |
| NM_001199482 | BRD1 | -0.10 | 0.10 | 7.29E-01 |
| NM_001257284 | ARPC4 | 0.14 | 0.14 | 7.29E-01 |
| NM_001277374 | POLR3H | 0.14 | 0.14 | 7.29E-01 |
| NM_001277778 | IQGAP2 | 0.13 | 0.14 | 7.29E-01 |
| NM_001305398 | SLC20A2 | -0.17 | 0.17 | 7.29E-01 |
| NM_205029 | MITF | -0.17 | 0.18 | 7.29E-01 |
| NM_001007946 | SGPL1 | -0.09 | 0.10 | 7.31E-01 |
| NM_001031337 | STK3 | -0.18 | 0.18 | 7.32E-01 |
| NM_001001312 | KRT15 | -0.17 | 0.18 | 7.32E-01 |
| NM_206983 | PLA2R1 | -0.12 | 0.13 | 7.32E-01 |
| NM_001001467 | TOB1 | -0.10 | 0.10 | 7.33E-01 |
| NM_001006195 | THOC5 | -0.09 | 0.09 | 7.33E-01 |
| NM_001012772 | CTDSPL2 | -0.11 | 0.11 | 7.33E-01 |
| NM_001030661 | SETD1B | -0.16 | 0.17 | 7.33E-01 |
| NM_204648 | EIF2AK1 | 0.09 | 0.09 | 7.33E-01 |
| NM_205315 | TUBB | -0.11 | 0.12 | 7.33E-01 |
| NM_001030801 | ATP6AP2 | 0.14 | 0.15 | 7.33E-01 |
| NM_001031368 | SRSF7 | 0.13 | 0.13 | 7.33E-01 |
| NM_001031440 | NCBP1 | -0.08 | 0.08 | 7.33E-01 |
| NM_001162372 | PAK1 | -0.14 | 0.15 | 7.33E-01 |
| NM_001195689 | ARHGEF39 | -0.13 | 0.13 | 7.33E-01 |
| NM_001277399 | CHMP5 | 0.14 | 0.15 | 7.33E-01 |
| NM_001277613 | HSPB11 | 0.17 | 0.18 | 7.33E-01 |
| NM_001277887 | JDP2 | -0.13 | 0.13 | 7.33E-01 |
| NM_204926 | USP2 | -0.13 | 0.13 | 7.33E-01 |
| NM_205313 | THRA | -0.12 | 0.12 | 7.33E-01 |
| NM_001030753 | H2AFJ | 0.17 | 0.17 | 7.33E-01 |
| NM_001277384 | GLRX3 | 0.12 | 0.12 | 7.33E-01 |
| NM_001030341 | FLNB | -0.12 | 0.13 | 7.33E-01 |
| NM_001006588 | HYOU1 | 0.12 | 0.13 | 7.34E-01 |
| NM_001031010 | GMNN | 0.12 | 0.12 | 7.35E-01 |
| NM_001030884 | RASSF2 | 0.17 | 0.18 | 7.35E-01 |
| NM_001012823 | ACOT9 | -0.13 | 0.14 | 7.35E-01 |
| NM_001006405 | TCP1 | 0.11 | 0.12 | 7.36E-01 |
| NM_001031548 | ACP2 | -0.11 | 0.11 | 7.36E-01 |
| NM_204657 | BCKDHB | 0.11 | 0.11 | 7.36E-01 |
| NM_001006207 | UTP6 | 0.08 | 0.09 | 7.37E-01 |
| NM_001006396 | WDR43 | -0.13 | 0.14 | 7.37E-01 |
| NM_001277907 | ALYREF | 0.14 | 0.14 | 7.37E-01 |
| NM_204341 | TNKS2 | -0.17 | 0.18 | 7.37E-01 |
| NM_205412 | LOC396380 | 0.15 | 0.16 | 7.37E-01 |
| NM_205271 | NFIC | 0.13 | 0.13 | 7.37E-01 |
| NM_001006542 | HOOK1 | -0.17 | 0.18 | 7.37E-01 |
| NM_001012533 | CCT2 | 0.10 | 0.11 | 7.37E-01 |
| NM_001012730 | EXOSC10 | -0.11 | 0.12 | 7.37E-01 |
| NM_001039300 | CAMK2D | 0.14 | 0.14 | 7.37E-01 |
| NM_001082418 | ADAM33 | -0.17 | 0.18 | 7.37E-01 |
| NM_001277550 | GNAO1 | -0.17 | 0.18 | 7.37E-01 |
| NM_001305644 | UBE3B | -0.17 | 0.18 | 7.37E-01 |
| NM_204384 | TFCP2 | 0.13 | 0.13 | 7.37E-01 |
| NM_204870 | JAK1 | -0.17 | 0.18 | 7.37E-01 |
| NM_205172 | ACTG2 | -0.17 | 0.18 | 7.37E-01 |
| NM_001006340 | PEX11G | -0.15 | 0.16 | 7.37E-01 |
| NM_001277475 | SSR3 | 0.10 | 0.11 | 7.37E-01 |
| NM_001001471 | PTRF | -0.09 | 0.10 | 7.38E-01 |
| NM_001030918 | CSDE1 | -0.10 | 0.11 | 7.38E-01 |
| NM_001031468 | PMM2 | 0.10 | 0.11 | 7.38E-01 |
| NM_205428 | TGFBR2 | -0.17 | 0.18 | 7.38E-01 |
| NM_001001192 | ST3GAL5 | -0.16 | 0.17 | 7.38E-01 |
| NM_001293302 | POLR2C | 0.15 | 0.15 | 7.38E-01 |
| NM_001006488 | EIF5 | 0.09 | 0.09 | 7.38E-01 |
| NM_001012607 | SNX2 | 0.08 | 0.08 | 7.38E-01 |
| NM_001030700 | COX10 | -0.10 | 0.10 | 7.38E-01 |
| NM_001030811 | REV1 | -0.12 | 0.12 | 7.38E-01 |
| NM_001030840 | USP6NL | -0.13 | 0.14 | 7.38E-01 |
| NM_001031347 | CIRBP | 0.10 | 0.11 | 7.38E-01 |
| NM_001079747 | AAMP | 0.11 | 0.12 | 7.38E-01 |
| NM_001199667 | HAUS2 | 0.12 | 0.13 | 7.38E-01 |
| NM_001277551 | CPSF4 | -0.09 | 0.09 | 7.38E-01 |
| NM_204713 | RDH5 | 0.16 | 0.17 | 7.38E-01 |
| NM_204766 | MYH15 | 0.15 | 0.16 | 7.38E-01 |
| NM_205001 | HMGA2 | -0.17 | 0.18 | 7.38E-01 |
| NM_001006556 | GMPS | 0.09 | 0.10 | 7.39E-01 |
| NM_001007832 | PAIP2 | 0.09 | 0.09 | 7.39E-01 |
| NM_001199909 | PDK4 | -0.16 | 0.17 | 7.39E-01 |
| NM_001277437 | PLEK2 | -0.17 | 0.18 | 7.39E-01 |
| NM_001277562 | ATP5S | 0.15 | 0.16 | 7.39E-01 |
| NM_001282341 | GLOD4 | 0.14 | 0.15 | 7.39E-01 |
| NM_204370 | CAV3 | 0.12 | 0.13 | 7.39E-01 |
| NM_204525 | RAB5C | 0.08 | 0.08 | 7.39E-01 |
| NM_001006281 | RP11-234B24.6 | 0.11 | 0.11 | 7.39E-01 |
| NM_001199425 | SPTBN1 | 0.11 | 0.12 | 7.39E-01 |
| NM_204778 | MRE11A | -0.15 | 0.16 | 7.40E-01 |
| NM_001277276 | PYROXD1 | -0.13 | 0.14 | 7.40E-01 |
| NM_001199608 | RHOBTB1 | -0.17 | 0.18 | 7.41E-01 |
| NM_001281500 | RPL34 | 0.14 | 0.15 | 7.41E-01 |
| NM_001199477 | TMEM60 | 0.14 | 0.15 | 7.41E-01 |
| NM_001005830 | MAP2K1 | 0.12 | 0.12 | 7.42E-01 |
| NM_001006337 | LSM7 | 0.09 | 0.10 | 7.42E-01 |
| NM_001006422 | ENPP4 | 0.10 | 0.11 | 7.42E-01 |
| NM_001007846 | CRK | -0.16 | 0.17 | 7.45E-01 |
| NM_001079714 | COL1A2 | 0.13 | 0.14 | 7.45E-01 |
| NM_001012774 | FHOD1 | -0.12 | 0.12 | 7.45E-01 |
| NM_205425 | CSK | -0.12 | 0.12 | 7.45E-01 |
| NM_001039284 | FOXN2 | -0.16 | 0.17 | 7.46E-01 |
| NM_001042538 | SPTAN1 | -0.13 | 0.14 | 7.46E-01 |
| NM_204510 | CXCL12 | -0.16 | 0.17 | 7.47E-01 |
| NM_001031159 | DHX15 | 0.08 | 0.08 | 7.47E-01 |
| NM_001006421 | MCM3 | -0.09 | 0.10 | 7.48E-01 |
| NM_001001749 | LAMP2 | -0.16 | 0.17 | 7.48E-01 |
| NM_001012699 | MTMR8 | -0.12 | 0.13 | 7.48E-01 |
| NM_001031163 | TMEM128 | 0.14 | 0.15 | 7.48E-01 |
| NM_001031266 | SEC22B | -0.07 | 0.08 | 7.48E-01 |
| NM_001031345 | CTSS | -0.12 | 0.13 | 7.48E-01 |
| NM_001031522 | PIGM | 0.13 | 0.14 | 7.48E-01 |
| NM_001277489 | NIP7 | 0.13 | 0.14 | 7.48E-01 |
| NM_204526 | CASQ2 | 0.13 | 0.14 | 7.48E-01 |
| NM_204746 | PBX1 | 0.11 | 0.12 | 7.48E-01 |
| NM_001030945 | CCNE2 | 0.13 | 0.14 | 7.48E-01 |
| NM_001004413 | NCSTN | -0.10 | 0.11 | 7.49E-01 |
| NM_205258 | RAN | 0.13 | 0.15 | 7.49E-01 |
| NM_001030612 | SPINK5 | 0.16 | 0.18 | 7.49E-01 |
| NM_001031433 | IDUA | -0.12 | 0.13 | 7.49E-01 |
| NM_001079477 | SLC26A5 | -0.17 | 0.18 | 7.49E-01 |
| NM_213581 | NUCKS1 | 0.16 | 0.17 | 7.49E-01 |
| NM_001167765 | CCNC | -0.11 | 0.12 | 7.49E-01 |
| NM_001006180 | AL158801.1 | 0.09 | 0.10 | 7.50E-01 |
| NM_001006227 | MAPK11 | 0.14 | 0.15 | 7.50E-01 |
| NM_001006471 | TOLLIP | -0.15 | 0.16 | 7.50E-01 |
| NM_001007853 | DDX47 | 0.10 | 0.11 | 7.50E-01 |
| NM_001012546 | PLEKHB1 | 0.15 | 0.17 | 7.50E-01 |
| NM_001012549 | NRAS | 0.08 | 0.09 | 7.50E-01 |
| NM_001012806 | VPS53 | -0.12 | 0.14 | 7.50E-01 |
| NM_001012897 | LRP5 | -0.14 | 0.16 | 7.50E-01 |
| NM_001012909 | ARHGAP19 | -0.11 | 0.12 | 7.50E-01 |
| NM_001030546 | LACTB | -0.11 | 0.12 | 7.50E-01 |
| NM_001030687 | TOR1A | 0.09 | 0.10 | 7.50E-01 |
| NM_001031132 | SGK196 | 0.09 | 0.10 | 7.50E-01 |
| NM_001031533 | SNX12 | -0.11 | 0.12 | 7.50E-01 |
| NM_001031579 | LMBRD2 | -0.16 | 0.18 | 7.50E-01 |
| NM_001079753 | PRC1 | 0.08 | 0.09 | 7.50E-01 |
| NM_001080864 | RP11-156P1.2 | -0.11 | 0.13 | 7.50E-01 |
| NM_001081698 | KIFC1 | -0.16 | 0.17 | 7.50E-01 |
| NM_001114126 | COX15 | -0.12 | 0.13 | 7.50E-01 |
| NM_001135166 | DMB2 | -0.16 | 0.18 | 7.50E-01 |
| NM_001199435 | PLCB4 | -0.11 | 0.12 | 7.50E-01 |
| NM_001199536 | CTNNA3 | -0.13 | 0.15 | 7.50E-01 |
| NM_001199586 | SPAG6 | 0.16 | 0.18 | 7.50E-01 |
| NM_001199684 | RBM25 | 0.11 | 0.12 | 7.50E-01 |
| NM_001277351 | TUBAL3 | 0.16 | 0.18 | 7.50E-01 |
| NM_001277612 | CDIP1 | -0.15 | 0.16 | 7.50E-01 |
| NM_001277713 | RAB24 | 0.14 | 0.16 | 7.50E-01 |
| NM_001277729 | IAH1 | 0.12 | 0.14 | 7.50E-01 |
| NM_001293102 | SEMA4D | -0.13 | 0.14 | 7.50E-01 |
| NM_204369 | HMGA1 | -0.15 | 0.17 | 7.50E-01 |
| NM_204381 | THY1 | 0.09 | 0.10 | 7.50E-01 |
| NM_204599 | SNRPB | 0.14 | 0.15 | 7.50E-01 |
| NM_205199 | LPAR6 | -0.16 | 0.17 | 7.50E-01 |
| NM_205308 | FABP7 | 0.14 | 0.16 | 7.50E-01 |
| NM_205350 | BTG1 | -0.11 | 0.12 | 7.50E-01 |
| NM_206878 | CCT4 | 0.10 | 0.11 | 7.50E-01 |
| NM_213574 | NDUFC2 | 0.15 | 0.17 | 7.50E-01 |
| NM_001006182 | SEPT2L | 0.09 | 0.10 | 7.50E-01 |
| NM_001257297 | RPS10 | 0.13 | 0.14 | 7.50E-01 |
| NM_001006457 | PPARGC1A | -0.15 | 0.17 | 7.51E-01 |
| NM_204477 | NDC80 | -0.11 | 0.12 | 7.52E-01 |
| NM_001277743 | SFXN1 | -0.12 | 0.14 | 7.52E-01 |
| NM_001267573 | TBC1D1 | -0.11 | 0.12 | 7.52E-01 |
| NM_204466 | STK40 | -0.08 | 0.09 | 7.53E-01 |
| NM_001006509 | ASNSD1 | -0.10 | 0.11 | 7.53E-01 |
| NM_204730 | ANXA6 | 0.10 | 0.11 | 7.53E-01 |
| NM_001012820 | CLDND1 | 0.10 | 0.11 | 7.55E-01 |
| NM_205056 | HADHA | -0.10 | 0.11 | 7.55E-01 |
| NM_001005843 | UQCRFS1 | 0.13 | 0.14 | 7.56E-01 |
| NM_001006244 | C1H12ORF23 | 0.08 | 0.10 | 7.56E-01 |
| NM_001006277 | N6AMT2 | -0.09 | 0.10 | 7.56E-01 |
| NM_001006315 | PNRC2 | -0.08 | 0.09 | 7.56E-01 |
| NM_001006325 | NFYA | -0.10 | 0.12 | 7.56E-01 |
| NM_001006412 | HMGN3 | 0.11 | 0.13 | 7.56E-01 |
| NM_001012538 | SLC37A3 | -0.12 | 0.14 | 7.56E-01 |
| NM_001012571 | ITM2A | -0.07 | 0.08 | 7.56E-01 |
| NM_001030339 | CD81 | -0.07 | 0.08 | 7.56E-01 |
| NM_001030619 | RPS14 | 0.13 | 0.15 | 7.56E-01 |
| NM_001030620 | CANX | 0.11 | 0.12 | 7.56E-01 |
| NM_001030642 | CDR2 | -0.13 | 0.15 | 7.56E-01 |
| NM_001030833 | RPS11 | 0.11 | 0.13 | 7.56E-01 |
| NM_001031047 | ANGEL2 | 0.12 | 0.14 | 7.56E-01 |
| NM_001031096 | ASXL2 | -0.16 | 0.18 | 7.56E-01 |
| NM_001031112 | MSN | -0.08 | 0.09 | 7.56E-01 |
| NM_001031141 | SEC31A | -0.10 | 0.11 | 7.56E-01 |
| NM_001031297 | ZRANB2 | 0.09 | 0.11 | 7.56E-01 |
| NM_001031428 | DAZAP1 | 0.10 | 0.11 | 7.56E-01 |
| NM_001039335 | SMC5 | 0.12 | 0.14 | 7.56E-01 |
| NM_001142850 | ADAM12 | -0.14 | 0.16 | 7.56E-01 |
| NM_001166326 | PPIA | 0.12 | 0.13 | 7.56E-01 |
| NM_001194927 | SQLE | -0.15 | 0.16 | 7.56E-01 |
| NM_001199644 | SATB1 | -0.11 | 0.12 | 7.56E-01 |
| NM_001271939 | PGRMC1 | 0.10 | 0.11 | 7.56E-01 |
| NM_001277498 | GATAD1 | 0.13 | 0.15 | 7.56E-01 |
| NM_001277525 | NDUFB6 | 0.13 | 0.15 | 7.56E-01 |
| NM_001282608 | LTBP1 | 0.12 | 0.13 | 7.56E-01 |
| NM_001302128 | SMIM3 | 0.14 | 0.16 | 7.56E-01 |
| NM_204471 | USP7 | -0.10 | 0.11 | 7.56E-01 |
| NM_204485 | HMGCR | -0.14 | 0.16 | 7.56E-01 |
| NM_204542 | NR1H3 | -0.09 | 0.10 | 7.56E-01 |
| NM_001001316 | CTDSPL | -0.14 | 0.16 | 7.56E-01 |
| NM_001031037 | SERTAD2 | -0.14 | 0.15 | 7.56E-01 |
| NM_001204865 | STON1 | -0.12 | 0.13 | 7.56E-01 |
| NM_001008473 | GSKIP | 0.11 | 0.12 | 7.57E-01 |
| NM_001079741 | BORA | 0.09 | 0.10 | 7.57E-01 |
| NM_001004405 | CAPN3 | -0.16 | 0.18 | 7.57E-01 |
| NM_001030781 | QTRTD1 | -0.10 | 0.11 | 7.57E-01 |
| NM_001244905 | TAF11 | 0.10 | 0.11 | 7.57E-01 |
| NM_204860 | CD44 | 0.16 | 0.18 | 7.57E-01 |
| NM_001080212 | UCHL1 | 0.14 | 0.15 | 7.59E-01 |
| NM_001198607 | GLT8D1 | 0.09 | 0.11 | 7.59E-01 |
| NM_001006151 | RBM22 | -0.11 | 0.12 | 7.59E-01 |
| NM_204518 | SMC4 | 0.09 | 0.10 | 7.59E-01 |
| NM_001006379 | DROSHA | 0.11 | 0.13 | 7.60E-01 |
| NM_001012777 | BBS2 | 0.12 | 0.14 | 7.60E-01 |
| NM_205357 | BMPR1A | -0.15 | 0.17 | 7.60E-01 |
| NM_204697 | POPDC3 | -0.10 | 0.12 | 7.61E-01 |
| NM_001080863 | DMTF1 | -0.11 | 0.13 | 7.61E-01 |
| NM_001007841 | C15H12ORF49 | -0.08 | 0.09 | 7.62E-01 |
| NM_001195424 | UBE2E3 | -0.10 | 0.12 | 7.62E-01 |
| NM_204536 | RARA | -0.12 | 0.13 | 7.62E-01 |
| NM_204604 | SP1 | -0.13 | 0.15 | 7.62E-01 |
| NM_204904 | ATF2 | -0.16 | 0.18 | 7.62E-01 |
| NM_001006518 | NDUFS1 | -0.09 | 0.11 | 7.62E-01 |
| NM_001012539 | ARFGAP3 | 0.13 | 0.15 | 7.62E-01 |
| NM_001039603 | PRKAA1 | -0.14 | 0.16 | 7.62E-01 |
| NM_001079486 | FGL2 | 0.16 | 0.18 | 7.62E-01 |
| NM_001305245 | GLI1 | -0.15 | 0.17 | 7.62E-01 |
| NM_205457 | SRC | -0.13 | 0.15 | 7.62E-01 |
| NM_001031306 | GK5 | 0.13 | 0.15 | 7.62E-01 |
| NM_204738 | TAX1BP1 | 0.09 | 0.11 | 7.62E-01 |
| NM_001030877 | SAMD11 | -0.15 | 0.17 | 7.63E-01 |
| NM_204339 | TGFBR3 | -0.15 | 0.17 | 7.63E-01 |
| NM_204461 | RHOQ | -0.15 | 0.17 | 7.63E-01 |
| NM_001012587 | RPS6KA5 | -0.15 | 0.17 | 7.64E-01 |
| NM_001030916 | WDR77 | 0.11 | 0.13 | 7.64E-01 |
| NM_001031077 | OSTM1 | -0.14 | 0.16 | 7.64E-01 |
| NM_001245037 | PCDHGA2 | 0.15 | 0.18 | 7.64E-01 |
| NM_205469 | PKM | -0.08 | 0.09 | 7.64E-01 |
| NM_001006339 | COPE | 0.09 | 0.11 | 7.64E-01 |
| NM_001252123 | RND2 | -0.12 | 0.14 | 7.64E-01 |
| NM_204977 | SEMA3A | -0.15 | 0.18 | 7.64E-01 |
| NM_001031214 | GMFB | -0.15 | 0.18 | 7.64E-01 |
| NM_205047 | NME2 | 0.13 | 0.15 | 7.65E-01 |
| NM_001031074 | SERINC1 | 0.07 | 0.08 | 7.67E-01 |
| NM_205381 | CCND1 | 0.10 | 0.12 | 7.67E-01 |
| NM_001031230 | KIF11 | 0.08 | 0.10 | 7.67E-01 |
| NM_001006521 | RQCD1 | -0.09 | 0.10 | 7.67E-01 |
| NM_001080718 | MARCKSL1 | 0.11 | 0.13 | 7.67E-01 |
| NM_001277789 | C1GALT1C1 | 0.12 | 0.14 | 7.67E-01 |
| NM_001305166 | EYA3 | -0.07 | 0.09 | 7.67E-01 |
| NM_204506 | DSTYK | -0.15 | 0.18 | 7.67E-01 |
| NM_204880 | ATF4 | 0.09 | 0.10 | 7.67E-01 |
| NM_001001127 | EDNRB | -0.12 | 0.14 | 7.67E-01 |
| NM_001001527 | HSPB2 | 0.08 | 0.09 | 7.67E-01 |
| NM_001012920 | PTPLB | -0.09 | 0.10 | 7.67E-01 |
| NM_213584 | RAD17 | -0.11 | 0.12 | 7.67E-01 |
| NM_001004399 | LY86 | 0.14 | 0.16 | 7.68E-01 |
| NM_001006333 | NKIRAS2 | 0.09 | 0.10 | 7.68E-01 |
| NM_001006539 | AKR1A1 | -0.11 | 0.13 | 7.68E-01 |
| NM_001007956 | TMED5 | -0.07 | 0.08 | 7.68E-01 |
| NM_001030900 | AGO3 | 0.14 | 0.16 | 7.68E-01 |
| NM_001031066 | ARL14EP | 0.09 | 0.10 | 7.68E-01 |
| NM_001031323 | EIF2A | 0.09 | 0.11 | 7.68E-01 |
| NM_001130737 | PNPLA8 | -0.14 | 0.17 | 7.68E-01 |
| NM_001293287 | KCNJ8 | 0.12 | 0.14 | 7.68E-01 |
| NM_204617 | CXCR4 | 0.14 | 0.16 | 7.68E-01 |
| NM_204678 | TRA2B | 0.11 | 0.13 | 7.68E-01 |
| NM_205319 | FGFR2 | -0.10 | 0.12 | 7.68E-01 |
| NM_205372 | IRF7 | -0.14 | 0.17 | 7.68E-01 |
| NM_001001783 | RPS13 | 0.13 | 0.15 | 7.68E-01 |
| NM_001044646 | NSL1 | -0.15 | 0.17 | 7.68E-01 |
| NM_001199176 | PTCHD2 | -0.15 | 0.17 | 7.68E-01 |
| NM_205184 | EFNA5 | 0.13 | 0.16 | 7.68E-01 |
| NM_205303 | CAPN11 | -0.12 | 0.14 | 7.68E-01 |
| NM_001031343 | YWHAZ | 0.11 | 0.12 | 7.69E-01 |
| NM_001004769 | BTC | 0.14 | 0.17 | 7.71E-01 |
| NM_001005811 | FAM214A | -0.10 | 0.12 | 7.71E-01 |
| NM_001030774 | KPNA1 | -0.09 | 0.11 | 7.71E-01 |
| NM_001293211 | SENP8 | -0.11 | 0.13 | 7.71E-01 |
| NM_001006443 | SLC25A4 | 0.09 | 0.10 | 7.71E-01 |
| NM_001030935 | PTBP1 | -0.11 | 0.13 | 7.71E-01 |
| NM_001012950 | PAPD7 | -0.09 | 0.11 | 7.72E-01 |
| NM_001305123 | RELN | 0.14 | 0.17 | 7.72E-01 |
| NM_001007485 | USF1 | -0.09 | 0.11 | 7.72E-01 |
| NM_001012996 | KDELR3 | 0.08 | 0.10 | 7.72E-01 |
| NM_001030648 | NET1 | 0.12 | 0.14 | 7.72E-01 |
| NM_001030747 | DCN | -0.14 | 0.16 | 7.72E-01 |
| NM_001031197 | SRSF5 | 0.09 | 0.11 | 7.72E-01 |
| NM_001031245 | LANCL1 | 0.10 | 0.12 | 7.72E-01 |
| NM_001039275 | SLC9A8 | -0.14 | 0.17 | 7.72E-01 |
| NM_001199709 | SPRED1 | 0.12 | 0.14 | 7.72E-01 |
| NM_001278016 | LYRM2 | -0.15 | 0.18 | 7.72E-01 |
| NM_204231 | SLC25A6 | -0.08 | 0.10 | 7.72E-01 |
| NM_204667 | MMP9 | 0.14 | 0.17 | 7.72E-01 |
| NM_205083 | EPHA7 | -0.15 | 0.18 | 7.72E-01 |
| NM_205110 | TOP1 | 0.11 | 0.13 | 7.72E-01 |
| NM_205178 | ATIC | -0.09 | 0.11 | 7.72E-01 |
| NM_001282339 | GEMIN4 | -0.08 | 0.10 | 7.72E-01 |
| NM_001012801 | TBCD | -0.09 | 0.10 | 7.72E-01 |
| NM_001030816 | LIG4 | 0.14 | 0.17 | 7.72E-01 |
| NM_001162393 | USPL1 | -0.11 | 0.13 | 7.72E-01 |
| NM_001167726 | REL | -0.11 | 0.14 | 7.73E-01 |
| NM_001303036 | LSP1 | 0.09 | 0.11 | 7.73E-01 |
| NM_001004409 | TRPC1 | -0.15 | 0.18 | 7.73E-01 |
| NM_001030734 | C1H7ORF60 | -0.09 | 0.10 | 7.73E-01 |
| NM_001031232 | TMEM180 | -0.10 | 0.12 | 7.73E-01 |
| NM_001199666 | SNAP23 | -0.15 | 0.18 | 7.73E-01 |
| NM_001277383 | GLT8D2 | 0.15 | 0.18 | 7.73E-01 |
| NM_205291 | SERPINH1 | 0.13 | 0.16 | 7.73E-01 |
| NM_205344 | HMOX1 | -0.12 | 0.14 | 7.73E-01 |
| NM_001044660 | DSN1 | 0.11 | 0.14 | 7.73E-01 |
| NM_001012799 | ODF2 | -0.11 | 0.13 | 7.74E-01 |
| NM_001277745 | FAM13B | -0.15 | 0.18 | 7.75E-01 |
| NM_001012908 | RRP12 | 0.10 | 0.12 | 7.76E-01 |
| NM_001030573 | DYNC1LI2 | -0.15 | 0.18 | 7.76E-01 |
| NM_001031205 | ANGEL1 | -0.09 | 0.11 | 7.76E-01 |
| NM_001006317 | APLP2 | -0.07 | 0.09 | 7.77E-01 |
| NM_001007843 | URM1 | 0.11 | 0.13 | 7.77E-01 |
| NM_001030947 | PLEKHF2 | -0.11 | 0.13 | 7.77E-01 |
| NM_001006341 | CHERP | -0.07 | 0.09 | 7.77E-01 |
| NM_001031494 | SRRM1 | -0.08 | 0.09 | 7.77E-01 |
| NM_001045839 | FYCO1 | -0.14 | 0.17 | 7.77E-01 |
| NM_001199473 | MRPL53 | 0.10 | 0.12 | 7.77E-01 |
| NM_001290480 | C2CD2L | -0.15 | 0.18 | 7.77E-01 |
| NM_001004397 | SF3A2 | 0.10 | 0.12 | 7.78E-01 |
| NM_001167731 | TST | -0.11 | 0.13 | 7.78E-01 |
| NM_001277573 | C11H16ORF87 | 0.10 | 0.13 | 7.78E-01 |
| NM_001278011 | CPNE2 | -0.11 | 0.13 | 7.78E-01 |
| NM_001252033 | NOTCH2 | -0.11 | 0.13 | 7.78E-01 |
| NM_001006511 | ACADL | 0.10 | 0.12 | 7.78E-01 |
| NM_205330 | INCENP | 0.10 | 0.12 | 7.78E-01 |
| NM_206992 | POT1 | -0.10 | 0.12 | 7.79E-01 |
| NM_001005616 | PROX1 | -0.13 | 0.17 | 7.80E-01 |
| NM_001005823 | SNRPA1 | 0.12 | 0.15 | 7.80E-01 |
| NM_001006420 | NRBP1 | -0.08 | 0.10 | 7.80E-01 |
| NM_001079500 | DGUOK | 0.13 | 0.16 | 7.80E-01 |
| NM_001199493 | SIRT3 | -0.12 | 0.15 | 7.80E-01 |
| NM_001012527 | SPG7 | -0.07 | 0.09 | 7.81E-01 |
| NM_001030053 | MRPL48 | 0.13 | 0.15 | 7.81E-01 |
| NM_001310087 | TCTN3 | 0.09 | 0.11 | 7.81E-01 |
| NM_001030963 | GORASP1 | -0.09 | 0.11 | 7.81E-01 |
| NM_204939 | MRPS30 | 0.12 | 0.15 | 7.81E-01 |
| NM_001012786 | FBXL18 | -0.13 | 0.16 | 7.82E-01 |
| NM_001031180 | SWAP70 | 0.08 | 0.10 | 7.82E-01 |
| NM_001206496 | ALDOC | 0.08 | 0.10 | 7.82E-01 |
| NM_001277754 | LIPT1 | 0.10 | 0.13 | 7.82E-01 |
| NM_205507 | CKM | -0.14 | 0.17 | 7.82E-01 |
| NM_001001311 | KRT14 | -0.14 | 0.18 | 7.82E-01 |
| NM_001006166 | TBL3 | -0.09 | 0.11 | 7.82E-01 |
| NM_001039319 | BUD13 | -0.10 | 0.13 | 7.82E-01 |
| NM_001080117 | CLTC | -0.07 | 0.09 | 7.82E-01 |
| NM_001277547 | ACBD6 | 0.11 | 0.14 | 7.82E-01 |
| NM_205236 | HIC1 | 0.10 | 0.12 | 7.82E-01 |
| NM_001008788 | SLC30A7 | -0.12 | 0.14 | 7.84E-01 |
| NM_001012570 | LRRC59 | 0.09 | 0.11 | 7.84E-01 |
| NM_001012895 | SBF2 | -0.15 | 0.18 | 7.84E-01 |
| NM_001030895 | TMEM57 | 0.09 | 0.11 | 7.84E-01 |
| NM_001030938 | DDX49 | -0.07 | 0.09 | 7.84E-01 |
| NM_001031388 | NOP56 | 0.13 | 0.16 | 7.84E-01 |
| NM_001044684 | SP5 | -0.15 | 0.18 | 7.84E-01 |
| NM_001079476 | NMB | 0.14 | 0.18 | 7.84E-01 |
| NM_001277920 | CETN1 | 0.08 | 0.10 | 7.84E-01 |
| NM_001293315 | GMPPB | 0.11 | 0.14 | 7.84E-01 |
| NM_204443 | ZMYND19 | 0.11 | 0.14 | 7.84E-01 |
| NM_204481 | HS2ST1 | 0.08 | 0.09 | 7.84E-01 |
| NM_205103 | TBP | 0.07 | 0.09 | 7.84E-01 |
| NR_102328 | MKRN3 | 0.13 | 0.16 | 7.84E-01 |
| NM_001113744 | YKT6 | 0.09 | 0.11 | 7.84E-01 |
| NM_204403 | BUB1B | 0.07 | 0.09 | 7.84E-01 |
| NM_001012600 | PSMD1 | 0.09 | 0.11 | 7.84E-01 |
| NM_001006409 | ATG5 | -0.08 | 0.10 | 7.85E-01 |
| NM_001031135 | SEC24B | 0.07 | 0.09 | 7.85E-01 |
| NM_001031273 | SASS6 | 0.08 | 0.10 | 7.85E-01 |
| NM_001031390 | PRPF3 | 0.08 | 0.10 | 7.85E-01 |
| NM_001031541 | CCT7 | 0.08 | 0.10 | 7.85E-01 |
| NM_001031556 | PGAM1 | 0.09 | 0.12 | 7.85E-01 |
| NM_001101730 | NEFM | 0.14 | 0.17 | 7.85E-01 |
| NM_001109786 | MSANTD2 | -0.10 | 0.13 | 7.85E-01 |
| NM_001168009 | CTSL2 | 0.09 | 0.11 | 7.85E-01 |
| NM_001197034 | C17H9ORF16 | 0.12 | 0.15 | 7.85E-01 |
| NM_001199569 | VPS37C | -0.08 | 0.09 | 7.85E-01 |
| NM_001305054 | GAP43 | -0.14 | 0.18 | 7.85E-01 |
| NM_204302 | PSEN2 | -0.14 | 0.17 | 7.85E-01 |
| NM_205284 | LDHA | 0.12 | 0.15 | 7.85E-01 |
| NM_001007479 | RPL4 | -0.07 | 0.09 | 7.87E-01 |
| NM_001030385 | EDC3 | -0.13 | 0.17 | 7.87E-01 |
| NM_001030542 | NPAS2 | 0.14 | 0.17 | 7.87E-01 |
| NM_001030831 | PSPC1 | 0.07 | 0.09 | 7.87E-01 |
| NM_001030838 | STIM1 | -0.11 | 0.14 | 7.87E-01 |
| NM_001030891 | PPP1R8 | -0.09 | 0.12 | 7.87E-01 |
| NM_001031136 | UFSP2 | 0.07 | 0.08 | 7.87E-01 |
| NM_001031208 | CPSF2 | -0.07 | 0.09 | 7.87E-01 |
| NM_001031278 | PIGK | -0.10 | 0.13 | 7.87E-01 |
| NM_001100911 | COL9A1 | 0.14 | 0.18 | 7.87E-01 |
| NM_001105672 | ST7 | 0.10 | 0.13 | 7.87E-01 |
| NM_001199704 | ARG2 | 0.12 | 0.16 | 7.87E-01 |
| NM_001277443 | PSMA6 | 0.11 | 0.14 | 7.87E-01 |
| NM_001278024 | ATP6V0C | 0.10 | 0.12 | 7.87E-01 |
| NM_204264 | SUFU | -0.10 | 0.13 | 7.87E-01 |
| NR_105466 | MIR6597 | -0.14 | 0.18 | 7.87E-01 |
| NM_001030695 | ZNF302 | -0.14 | 0.17 | 7.87E-01 |
| NM_001097528 | GNG2 | -0.10 | 0.13 | 7.87E-01 |
| NM_001244912 | PANK4 | -0.09 | 0.11 | 7.87E-01 |
| NM_001305651 | HNRNPLL | 0.07 | 0.09 | 7.87E-01 |
| NM_204133 | ARNTL2 | -0.09 | 0.11 | 7.87E-01 |
| NM_204540 | SOCS2 | 0.12 | 0.15 | 7.87E-01 |
| NM_205419 | NTN1 | 0.14 | 0.18 | 7.87E-01 |
| NM_001302187 | SLN | -0.14 | 0.18 | 7.87E-01 |
| NM_001004407 | GYPC | -0.13 | 0.17 | 7.88E-01 |
| NM_204208 | CBL | -0.14 | 0.17 | 7.88E-01 |
| NM_001305665 | THRAP3 | -0.09 | 0.12 | 7.88E-01 |
| NM_205053 | CSNK1A1 | -0.12 | 0.15 | 7.88E-01 |
| NM_001006499 | TSPAN15 | 0.14 | 0.18 | 7.88E-01 |
| NM_001031315 | CCDC50 | -0.11 | 0.14 | 7.88E-01 |
| NM_001031464 | BRPF1 | -0.08 | 0.11 | 7.88E-01 |
| NM_001044634 | WDR36 | 0.08 | 0.11 | 7.88E-01 |
| NM_204305 | GAPDH | 0.09 | 0.11 | 7.88E-01 |
| NM_001006134 | USP4 | -0.09 | 0.11 | 7.88E-01 |
| NM_205468 | CTTN | -0.14 | 0.18 | 7.88E-01 |
| NM_001008450 | LZIC | -0.09 | 0.12 | 7.89E-01 |
| NM_001031422 | PIP5K1B | -0.07 | 0.09 | 7.89E-01 |
| NM_001031480 | DYRK2 | 0.09 | 0.11 | 7.89E-01 |
| NM_001031564 | CMPK1 | 0.08 | 0.10 | 7.89E-01 |
| NM_001080105 | GPR39 | -0.11 | 0.15 | 7.89E-01 |
| NM_001198602 | PSMA4 | 0.11 | 0.15 | 7.89E-01 |
| NM_001293319 | TEX264 | -0.12 | 0.15 | 7.89E-01 |
| NM_001006242 | DNAL4 | 0.08 | 0.10 | 7.89E-01 |
| NM_001031115 | DKC1 | 0.10 | 0.12 | 7.89E-01 |
| NM_001039306 | C7H2ORF69 | -0.09 | 0.12 | 7.89E-01 |
| NM_001109755 | SNX13 | -0.13 | 0.17 | 7.89E-01 |
| NM_001199413 | ADCK3 | 0.07 | 0.09 | 7.89E-01 |
| NM_205033 | NES | 0.10 | 0.13 | 7.89E-01 |
| NM_001006140 | CCDC174 | -0.10 | 0.13 | 7.89E-01 |
| NM_001201445 | SRRD | 0.09 | 0.11 | 7.89E-01 |
| NM_204829 | LDB2 | -0.14 | 0.18 | 7.89E-01 |
| NM_001030723 | APPBP2 | -0.07 | 0.10 | 7.90E-01 |
| NM_205328 | HNRNPAB | 0.09 | 0.11 | 7.90E-01 |
| NR_003250 | LOC768251 | 0.12 | 0.15 | 7.90E-01 |
| NM_205497 | EGFR | -0.13 | 0.17 | 7.91E-01 |
| NM_001005431 | FKBP5 | -0.10 | 0.13 | 7.92E-01 |
| NM_001006146 | SLU7 | 0.08 | 0.11 | 7.92E-01 |
| NM_001006274 | CKAP2 | 0.10 | 0.13 | 7.92E-01 |
| NM_001006515 | GTF3C3 | -0.09 | 0.12 | 7.92E-01 |
| NM_001006531 | STX6 | -0.08 | 0.10 | 7.92E-01 |
| NM_001030357 | PCSK7 | -0.13 | 0.17 | 7.92E-01 |
| NM_001030837 | RRM1 | 0.06 | 0.08 | 7.92E-01 |
| NM_001031374 | H2AFZ | 0.12 | 0.16 | 7.92E-01 |
| NM_001199651 | SLC35C1 | 0.09 | 0.11 | 7.92E-01 |
| NM_001252272 | PSPH | 0.14 | 0.18 | 7.92E-01 |
| NM_001277880 | RPS29 | 0.11 | 0.15 | 7.92E-01 |
| NM_001302185 | COA5 | 0.12 | 0.15 | 7.92E-01 |
| NM_204190 | MAB21L2 | 0.12 | 0.16 | 7.92E-01 |
| NM_204833 | ELAVL1 | -0.11 | 0.14 | 7.92E-01 |
| NM_205154 | PTPRA | -0.08 | 0.10 | 7.92E-01 |
| NM_001039305 | MGEA5 | -0.10 | 0.13 | 7.92E-01 |
| NM_001006321 | AP002884.2 | 0.11 | 0.15 | 7.93E-01 |
| NM_001006356 | ACBD5 | 0.07 | 0.09 | 7.93E-01 |
| NM_001006541 | TMEM59 | 0.08 | 0.11 | 7.93E-01 |
| NM_001006578 | OXCT1 | 0.09 | 0.12 | 7.93E-01 |
| NM_001012568 | CSTF3 | -0.06 | 0.08 | 7.93E-01 |
| NM_001031294 | WLS | 0.09 | 0.11 | 7.93E-01 |
| NM_001031536 | TRAPPC11 | 0.08 | 0.11 | 7.93E-01 |
| NM_001044692 | DACT1 | -0.09 | 0.11 | 7.93E-01 |
| NM_001135678 | ANKRD27 | -0.14 | 0.18 | 7.93E-01 |
| NM_001164084 | SEPHS1 | -0.07 | 0.09 | 7.93E-01 |
| NM_001267553 | JUP | -0.09 | 0.11 | 7.93E-01 |
| NM_001277449 | NCBP2 | 0.12 | 0.16 | 7.93E-01 |
| NM_001277456 | LRRC6 | -0.12 | 0.16 | 7.93E-01 |
| NM_204149 | NBL1 | 0.10 | 0.14 | 7.93E-01 |
| NM_204356 | TIA1 | 0.08 | 0.10 | 7.93E-01 |
| NM_204428 | FUT11 | 0.10 | 0.13 | 7.93E-01 |
| NM_204522 | NR13 | 0.12 | 0.16 | 7.93E-01 |
| NM_204665 | CD40 | 0.10 | 0.13 | 7.93E-01 |
| NM_204719 | CSPG5 | -0.11 | 0.14 | 7.93E-01 |
| NM_205177 | RP11-295K3.1 | -0.12 | 0.16 | 7.93E-01 |
| NM_001006129 | NAE1 | 0.09 | 0.12 | 7.93E-01 |
| NM_001006223 | ALDH3A2 | -0.08 | 0.10 | 7.93E-01 |
| NM_001006528 | DARS | 0.09 | 0.12 | 7.93E-01 |
| NM_001006550 | RFC4 | 0.11 | 0.15 | 7.93E-01 |
| NM_001012787 | MAP2K3 | -0.09 | 0.12 | 7.93E-01 |
| NM_001031155 | SGCB | 0.10 | 0.13 | 7.93E-01 |
| NM_001031272 | C8H1ORF27 | 0.09 | 0.12 | 7.93E-01 |
| NM_001031560 | NPL | -0.11 | 0.15 | 7.93E-01 |
| NM_001113571 | GTF3A | 0.09 | 0.12 | 7.93E-01 |
| NM_001198662 | ENPP2 | 0.12 | 0.16 | 7.93E-01 |
| NM_001277371 | RRP7A | 0.12 | 0.16 | 7.93E-01 |
| NM_001277838 | OGG1 | 0.11 | 0.15 | 7.93E-01 |
| NM_001282239 | MUM1 | -0.06 | 0.09 | 7.93E-01 |
| NM_204454 | CRHR2 | 0.14 | 0.18 | 7.93E-01 |
| NM_204749 | RP11-231C18.3 | -0.09 | 0.12 | 7.93E-01 |
| NM_205472 | POU2F1 | 0.13 | 0.18 | 7.93E-01 |
| NM_001031598 | TUBB3 | 0.14 | 0.18 | 7.93E-01 |
| NM_001039298 | HERC3 | 0.12 | 0.15 | 7.93E-01 |
| NM_001277491 | HPD | 0.13 | 0.18 | 7.94E-01 |
| NM_204670 | RBBP7 | 0.10 | 0.13 | 7.94E-01 |
| NM_001031085 | FAM46A | -0.10 | 0.14 | 7.94E-01 |
| NM_001031248 | GLS | -0.13 | 0.17 | 7.94E-01 |
| NM_001252611 | ATP6AP1 | -0.07 | 0.09 | 7.94E-01 |
| NM_204886 | MTPN | -0.07 | 0.10 | 7.94E-01 |
| NM_001024456 | NSF | -0.06 | 0.08 | 7.94E-01 |
| NM_001006442 | OSTC | 0.11 | 0.14 | 7.95E-01 |
| NM_001006472 | CD151 | 0.07 | 0.09 | 7.95E-01 |
| NM_001006474 | API5 | -0.07 | 0.10 | 7.95E-01 |
| NM_001030943 | RIPK2 | 0.09 | 0.11 | 7.95E-01 |
| NM_001031316 | FYTTD1 | -0.08 | 0.11 | 7.95E-01 |
| NM_001031611 | RGP1 | -0.09 | 0.12 | 7.95E-01 |
| NM_001163232 | GPX3 | 0.09 | 0.12 | 7.95E-01 |
| NM_001278058 | BIK | -0.13 | 0.18 | 7.95E-01 |
| NM_204635 | BCMO1 | -0.13 | 0.18 | 7.95E-01 |
| NM_205312 | ETS2 | -0.13 | 0.17 | 7.95E-01 |
| NM_205534 | COL6A3 | 0.10 | 0.13 | 7.95E-01 |
| NM_001005808 | IDH3A | 0.07 | 0.09 | 7.95E-01 |
| NM_001006188 | RNF34 | -0.10 | 0.13 | 7.95E-01 |
| NM_001006535 | FUBP1 | 0.10 | 0.14 | 7.95E-01 |
| NM_001008677 | CHAF1B | -0.12 | 0.17 | 7.95E-01 |
| NM_001012531 | NEK6 | -0.07 | 0.09 | 7.95E-01 |
| NM_001012775 | COG4 | -0.07 | 0.10 | 7.95E-01 |
| NM_001012900 | G2E3 | -0.11 | 0.15 | 7.95E-01 |
| NM_001030767 | CECR1 | -0.12 | 0.16 | 7.95E-01 |
| NM_001030832 | CHORDC1 | 0.13 | 0.17 | 7.95E-01 |
| NM_001030937 | HMHA1 | -0.12 | 0.16 | 7.95E-01 |
| NM_001031360 | MTHFD2 | -0.08 | 0.11 | 7.95E-01 |
| NM_001031482 | H3F3C | 0.08 | 0.11 | 7.95E-01 |
| NM_001083920 | SERPINE2 | 0.12 | 0.17 | 7.95E-01 |
| NM_001135677 | GSPT1 | -0.06 | 0.09 | 7.95E-01 |
| NM_001143969 | ARL5B | 0.07 | 0.10 | 7.95E-01 |
| NM_001168705 | RAB27A | -0.13 | 0.17 | 7.95E-01 |
| NM_001177735 | PHB | 0.11 | 0.14 | 7.95E-01 |
| NM_001199857 | CDK2 | 0.11 | 0.14 | 7.95E-01 |
| NM_001277364 | ARHGDIB | 0.08 | 0.10 | 7.95E-01 |
| NM_001278019 | RARS2 | 0.07 | 0.09 | 7.95E-01 |
| NM_001301686 | SRA1 | 0.10 | 0.13 | 7.95E-01 |
| NM_204103 | ADD3 | -0.12 | 0.17 | 7.95E-01 |
| NM_204508 | MTF2 | -0.07 | 0.09 | 7.95E-01 |
| NM_204681 | MGAT4C | -0.10 | 0.14 | 7.95E-01 |
| NM_204724 | ATP6V0A1 | -0.10 | 0.14 | 7.95E-01 |
| NM_205142 | SNAI1 | -0.10 | 0.14 | 7.95E-01 |
| NM_205208 | CSRP2 | 0.11 | 0.14 | 7.95E-01 |
| NM_205242 | LRP1 | -0.13 | 0.18 | 7.95E-01 |
| NM_206859 | CEBPG | 0.09 | 0.13 | 7.95E-01 |
| NM_213558 | DNAJC27 | -0.13 | 0.18 | 7.95E-01 |
| NR_131063 | RBM24 | -0.09 | 0.12 | 7.95E-01 |
| NM_001005345 | PDLIM7 | -0.08 | 0.11 | 7.95E-01 |
| NM_001006280 | MED17 | -0.07 | 0.09 | 7.95E-01 |
| NM_001031204 | TTLL5 | 0.09 | 0.12 | 7.95E-01 |
| NM_001031285 | BTF3L4 | 0.07 | 0.09 | 7.95E-01 |
| NM_001031332 | GTSE1 | 0.07 | 0.10 | 7.95E-01 |
| NM_001114659 | JMJD7 | 0.12 | 0.16 | 7.95E-01 |
| NM_001256948 | MFAP1 | 0.09 | 0.13 | 7.95E-01 |
| NM_001305161 | LTN1 | -0.11 | 0.14 | 7.95E-01 |
| NM_204145 | ABCA1 | -0.08 | 0.12 | 7.95E-01 |
| NM_204150 | MAPK1 | -0.13 | 0.18 | 7.95E-01 |
| NM_204401 | TRIB2 | 0.06 | 0.08 | 7.95E-01 |
| NM_204934 | GSN | -0.07 | 0.09 | 7.95E-01 |
| NM_205022 | F10 | 0.13 | 0.18 | 7.95E-01 |
| NM_001308001 | TMED11P | -0.12 | 0.17 | 7.96E-01 |
| NM_001199714 | SCFD1 | 0.10 | 0.13 | 7.96E-01 |
| NM_001006131 | DHODH | 0.11 | 0.16 | 7.97E-01 |
| NM_001006204 | SRP68 | -0.06 | 0.08 | 7.97E-01 |
| NM_001006502 | RP11-411B6.6 | 0.11 | 0.15 | 7.97E-01 |
| NM_001012586 | ZC3H14 | -0.08 | 0.11 | 7.97E-01 |
| NM_001030541 | POSTN | -0.13 | 0.18 | 7.97E-01 |
| NM_001031290 | USP1 | 0.08 | 0.10 | 7.97E-01 |
| NM_001031454 | IREB2 | -0.07 | 0.09 | 7.97E-01 |
| NM_001134359 | FANCA | -0.10 | 0.13 | 7.97E-01 |
| NM_001277723 | FHL5 | -0.12 | 0.17 | 7.97E-01 |
| NM_001282295 | TXNDC12 | 0.08 | 0.12 | 7.97E-01 |
| NM_001305076 | FAM195B | 0.12 | 0.17 | 7.97E-01 |
| NM_205421 | GATA5 | 0.07 | 0.09 | 7.97E-01 |
| NM_204790 | COL5A1 | -0.09 | 0.12 | 7.97E-01 |
| NM_001008463 | PGS1 | -0.07 | 0.09 | 7.97E-01 |
| NM_001045827 | PCDH1 | -0.13 | 0.18 | 7.97E-01 |
| NM_001252141 | SRF | -0.11 | 0.15 | 7.97E-01 |
| NM_001006414 | FBXO9 | -0.08 | 0.11 | 7.97E-01 |
| NM_204552 | BID | -0.10 | 0.14 | 7.98E-01 |
| NM_001302180 | SNN | -0.13 | 0.17 | 7.99E-01 |
| NM_001030669 | RAB35 | -0.07 | 0.10 | 7.99E-01 |
| NM_001007824 | ACTG1 | 0.09 | 0.13 | 7.99E-01 |
| NM_001006500 | POLR3A | -0.13 | 0.18 | 8.00E-01 |
| NM_001030886 | PPP2R2A | -0.06 | 0.09 | 8.00E-01 |
| NM_001031583 | NDUFV1 | -0.07 | 0.10 | 8.00E-01 |
| NM_001159663 | SMAD7B | 0.09 | 0.13 | 8.00E-01 |
| NM_001031420 | PLIN2 | -0.13 | 0.18 | 8.00E-01 |
| NM_001079473 | SLC8A1 | -0.13 | 0.18 | 8.00E-01 |
| NM_001270577 | UCKL1 | -0.09 | 0.13 | 8.00E-01 |
| NM_001130742 | PNPLA7 | -0.11 | 0.15 | 8.00E-01 |
| NM_001012874 | BPNT1 | 0.08 | 0.12 | 8.01E-01 |
| NM_001006158 | CCZ1 | 0.06 | 0.09 | 8.02E-01 |
| NM_001031539 | ELOVL6 | 0.10 | 0.14 | 8.02E-01 |
| NM_001115082 | LPAR1 | -0.08 | 0.12 | 8.02E-01 |
| NM_001006254 | NFKBIZ | -0.12 | 0.17 | 8.02E-01 |
| NM_001004391 | CDH4 | -0.13 | 0.18 | 8.02E-01 |
| NM_001012822 | SCAF4 | -0.11 | 0.15 | 8.02E-01 |
| NM_001031532 | NONO | -0.07 | 0.09 | 8.02E-01 |
| NM_001199646 | CKAP5 | -0.11 | 0.16 | 8.03E-01 |
| NM_001292086 | LEO1 | 0.08 | 0.11 | 8.03E-01 |
| NM_001291805 | CYB5R3 | -0.06 | 0.09 | 8.03E-01 |
| NM_205443 | PLAU | 0.13 | 0.18 | 8.03E-01 |
| NM_204795 | DACH2 | 0.13 | 0.18 | 8.04E-01 |
| NM_001030732 | TRABD | -0.08 | 0.12 | 8.04E-01 |
| NM_001031082 | ORC3 | 0.08 | 0.11 | 8.04E-01 |
| NM_001031389 | RFXANK | 0.09 | 0.13 | 8.04E-01 |
| NM_001031528 | EPT1 | -0.13 | 0.18 | 8.04E-01 |
| NM_001034815 | DNASE1L3 | 0.13 | 0.18 | 8.04E-01 |
| NM_204737 | C1H21ORF91 | -0.10 | 0.14 | 8.04E-01 |
| NM_205375 | TCF12 | -0.08 | 0.12 | 8.04E-01 |
| NM_001006481 | EIF2B2 | 0.07 | 0.10 | 8.05E-01 |
| NM_001030867 | RAB22A | -0.12 | 0.18 | 8.05E-01 |
| NM_001277872 | C7H2ORF76 | -0.12 | 0.17 | 8.05E-01 |
| NM_001001529 | LEPRE1 | 0.06 | 0.09 | 8.05E-01 |
| NM_001005347 | EVC | -0.12 | 0.18 | 8.05E-01 |
| NM_001006171 | RP11-1035H13.3 | 0.09 | 0.13 | 8.05E-01 |
| NM_001006183 | RANBP1 | 0.08 | 0.12 | 8.05E-01 |
| NM_001006345 | RPL7 | 0.09 | 0.13 | 8.05E-01 |
| NM_001006407 | CDC40 | -0.08 | 0.11 | 8.05E-01 |
| NM_001006435 | MAGT1 | -0.06 | 0.09 | 8.05E-01 |
| NM_001006543 | FARSB | 0.07 | 0.10 | 8.05E-01 |
| NM_001012543 | PSMG1 | 0.11 | 0.16 | 8.05E-01 |
| NM_001012784 | MFAP3 | -0.10 | 0.14 | 8.05E-01 |
| NM_001012886 | TMEM164 | -0.12 | 0.17 | 8.05E-01 |
| NM_001030593 | VGLL4 | -0.12 | 0.17 | 8.05E-01 |
| NM_001031147 | YTHDC1 | 0.11 | 0.16 | 8.05E-01 |
| NM_001031198 | KATNBL1 | 0.07 | 0.11 | 8.05E-01 |
| NM_001031222 | SLC16A9 | -0.08 | 0.12 | 8.05E-01 |
| NM_001031362 | PSMD3 | -0.08 | 0.12 | 8.05E-01 |
| NM_001039304 | ZFYVE27 | -0.09 | 0.13 | 8.05E-01 |
| NM_001039912 | PRKAB1 | -0.08 | 0.11 | 8.05E-01 |
| NM_001159715 | KDELR2 | 0.09 | 0.13 | 8.05E-01 |
| NM_001163650 | CALCRL | -0.12 | 0.17 | 8.05E-01 |
| NM_001168008 | DDOST | 0.08 | 0.11 | 8.05E-01 |
| NM_001199524 | ECD | -0.08 | 0.11 | 8.05E-01 |
| NM_001199625 | CPM | -0.10 | 0.14 | 8.05E-01 |
| NM_001252068 | SLC25A22 | 0.10 | 0.14 | 8.05E-01 |
| NM_001252163 | ARMC8 | -0.07 | 0.10 | 8.05E-01 |
| NM_001252398 | ARFIP1 | 0.07 | 0.10 | 8.05E-01 |
| NM_001277597 | MRPS34 | 0.07 | 0.10 | 8.05E-01 |
| NM_001282377 | ALKBH3 | -0.10 | 0.14 | 8.05E-01 |
| NM_001285499 | LOC417414 | -0.09 | 0.13 | 8.05E-01 |
| NM_001293210 | ADPGK | -0.09 | 0.13 | 8.05E-01 |
| NM_204368 | TRMT11 | -0.09 | 0.13 | 8.05E-01 |
| NM_204437 | TPX2 | -0.09 | 0.13 | 8.05E-01 |
| NM_204693 | MBD4 | 0.10 | 0.14 | 8.05E-01 |
| NM_204780 | NUDT16L1 | 0.11 | 0.16 | 8.05E-01 |
| NM_206981 | NAPRT | -0.08 | 0.11 | 8.05E-01 |
| NM_204386 | LOC378902 | -0.13 | 0.18 | 8.06E-01 |
| NM_001031221 | CCDC6 | -0.07 | 0.10 | 8.06E-01 |
| NM_001006483 | SPTLC2 | 0.10 | 0.15 | 8.07E-01 |
| NM_001007080 | BMPER | 0.11 | 0.15 | 8.07E-01 |
| NM_001030729 | ORC5 | -0.09 | 0.13 | 8.07E-01 |
| NM_001030772 | CDPF1 | -0.09 | 0.14 | 8.07E-01 |
| NM_001030994 | ELMO1 | 0.11 | 0.16 | 8.07E-01 |
| NM_001031218 | TSPAN14 | -0.12 | 0.18 | 8.07E-01 |
| NM_001031304 | KLHL24 | 0.09 | 0.13 | 8.07E-01 |
| NM_001031310 | WDFY1 | 0.07 | 0.10 | 8.07E-01 |
| NM_001079758 | TMEM38A | 0.08 | 0.12 | 8.07E-01 |
| NM_001099356 | ZNF692 | -0.12 | 0.18 | 8.07E-01 |
| NM_001271982 | CYB5R2 | 0.08 | 0.11 | 8.07E-01 |
| NM_001039601 | AOX2P | -0.12 | 0.18 | 8.08E-01 |
| NM_001004404 | GYLTL1B | -0.12 | 0.17 | 8.09E-01 |
| NM_001162390 | COL4A2 | -0.12 | 0.18 | 8.09E-01 |
| NM_205288 | ITGA8 | -0.12 | 0.18 | 8.09E-01 |
| NM_001257298 | NUDT3 | -0.10 | 0.14 | 8.09E-01 |
| NM_204509 | ROR1 | -0.10 | 0.14 | 8.09E-01 |
| NM_205416 | IRF8 | -0.10 | 0.15 | 8.09E-01 |
| NM_001001302 | MYH7 | -0.11 | 0.16 | 8.09E-01 |
| NM_001031103 | TNFRSF21 | -0.07 | 0.10 | 8.09E-01 |
| NM_001080715 | MYOCD | -0.12 | 0.18 | 8.09E-01 |
| NR_131971 | OIP5-AS1 | -0.05 | 0.08 | 8.11E-01 |
| NM_001199578 | MSRB3 | -0.08 | 0.12 | 8.12E-01 |
| NM_204337 | NBN | 0.10 | 0.15 | 8.12E-01 |
| NM_001006240 | TAB1 | -0.07 | 0.11 | 8.13E-01 |
| NM_001008471 | SLC39A13 | 0.09 | 0.13 | 8.13E-01 |
| NM_001256963 | TMEM208 | 0.11 | 0.16 | 8.13E-01 |
| NM_001282230 | NME3 | -0.08 | 0.12 | 8.13E-01 |
| NM_001289779 | PER3 | -0.10 | 0.15 | 8.13E-01 |
| NM_213583 | RAD9A | 0.11 | 0.16 | 8.13E-01 |
| NM_001012698 | TMEM18 | 0.10 | 0.15 | 8.13E-01 |
| NM_001195002 | WDR70 | -0.08 | 0.12 | 8.16E-01 |
| NM_001007963 | KPNA4 | -0.08 | 0.11 | 8.16E-01 |
| NM_204212 | HK2 | -0.11 | 0.16 | 8.16E-01 |
| NM_001031576 | MICAL1 | -0.12 | 0.18 | 8.17E-01 |
| NM_001080876 | NEIL1 | -0.11 | 0.16 | 8.18E-01 |
| NM_001199807 | MAPKAPK5 | -0.07 | 0.10 | 8.19E-01 |
| NM_001001899 | GGNBP2 | 0.07 | 0.11 | 8.20E-01 |
| NM_001257224 | MORC3 | -0.07 | 0.11 | 8.20E-01 |
| NM_001030763 | PARPBP | -0.10 | 0.16 | 8.21E-01 |
| NM_001031572 | LMF2 | -0.06 | 0.10 | 8.21E-01 |
| NM_001277347 | ANXA7 | 0.08 | 0.13 | 8.21E-01 |
| NM_204578 | CPZ | 0.07 | 0.10 | 8.21E-01 |
| NM_205487 | TIMP3 | 0.11 | 0.17 | 8.21E-01 |
| NM_204203 | PCBD2 | 0.11 | 0.16 | 8.21E-01 |
| NM_001199430 | NAPB | -0.09 | 0.13 | 8.21E-01 |
| NM_001030962 | MYD88 | 0.07 | 0.11 | 8.22E-01 |
| NM_001012814 | POLDIP3 | -0.06 | 0.09 | 8.23E-01 |
| NM_001012877 | REPS1 | -0.12 | 0.18 | 8.23E-01 |
| NM_001030783 | DCBLD2 | -0.08 | 0.12 | 8.23E-01 |
| NM_001080878 | SNUPN | -0.08 | 0.12 | 8.23E-01 |
| NM_001122691 | TXNRD2 | -0.07 | 0.10 | 8.23E-01 |
| NM_204099 | FZD4 | -0.12 | 0.18 | 8.23E-01 |
| NM_204483 | ITGB5 | -0.09 | 0.14 | 8.23E-01 |
| NM_001101649 | SNX24 | 0.08 | 0.12 | 8.23E-01 |
| NM_001031093 | C3H2ORF43 | -0.07 | 0.10 | 8.23E-01 |
| NM_001167701 | CASP2 | 0.06 | 0.09 | 8.23E-01 |
| NR_105445 | MIR6578 | -0.09 | 0.13 | 8.23E-01 |
| NM_001031223 | ERLIN1 | -0.07 | 0.11 | 8.24E-01 |
| NM_001031510 | GARS | 0.07 | 0.11 | 8.24E-01 |
| NM_001111323 | FAT3 | -0.11 | 0.17 | 8.24E-01 |
| NM_001277518 | MGME1 | 0.09 | 0.14 | 8.24E-01 |
| NM_001282159 | NUP107 | 0.07 | 0.10 | 8.24E-01 |
| NM_001305103 | USP13 | -0.09 | 0.13 | 8.24E-01 |
| NM_204710 | RAD54B | 0.09 | 0.13 | 8.24E-01 |
| NR_035086 | MIR1600 | 0.11 | 0.17 | 8.24E-01 |
| NM_001031243 | HIBCH | -0.06 | 0.10 | 8.24E-01 |
| NM_001005838 | AKTIP | -0.08 | 0.12 | 8.24E-01 |
| NM_204217 | RPS17 | 0.10 | 0.15 | 8.24E-01 |
| NM_204959 | MYOM1 | -0.11 | 0.17 | 8.25E-01 |
| NM_204251 | BIVM | -0.05 | 0.08 | 8.26E-01 |
| NM_001006335 | SMARCE1 | 0.06 | 0.09 | 8.28E-01 |
| NM_001193595 | AKIRIN2 | 0.08 | 0.12 | 8.29E-01 |
| NM_001007490 | AIFM1 | 0.07 | 0.11 | 8.29E-01 |
| NM_001007828 | HACD3 | -0.07 | 0.11 | 8.29E-01 |
| NM_001006308 | SNRNP40 | 0.08 | 0.12 | 8.29E-01 |
| NM_001006479 | ARHGAP11A | 0.07 | 0.11 | 8.29E-01 |
| NM_001006552 | TTC14 | -0.06 | 0.10 | 8.29E-01 |
| NM_001008478 | DR1 | 0.07 | 0.10 | 8.29E-01 |
| NM_001012770 | ASB7 | -0.10 | 0.16 | 8.29E-01 |
| NM_001012952 | CHN1 | 0.06 | 0.10 | 8.29E-01 |
| NM_001030581 | CYB5B | 0.06 | 0.09 | 8.29E-01 |
| NM_001030667 | DDT | 0.08 | 0.12 | 8.29E-01 |
| NM_001030759 | TMEM184B | -0.07 | 0.11 | 8.29E-01 |
| NM_001031101 | MTMR9 | -0.07 | 0.11 | 8.29E-01 |
| NM_001031604 | RP11-145E5.5 | 0.07 | 0.12 | 8.29E-01 |
| NM_001105610 | RAMP2 | 0.11 | 0.16 | 8.29E-01 |
| NM_001193639 | FAP | -0.11 | 0.18 | 8.29E-01 |
| NM_001198605 | COQ9 | 0.09 | 0.13 | 8.29E-01 |
| NM_001277574 | C16ORF45 | -0.10 | 0.15 | 8.29E-01 |
| NM_204743 | HOXB3 | -0.12 | 0.18 | 8.29E-01 |
| NM_204961 | NOS2 | -0.08 | 0.13 | 8.29E-01 |
| NM_205006 | FOXC1 | -0.10 | 0.15 | 8.29E-01 |
| NM_205339 | BCL2 | -0.10 | 0.16 | 8.29E-01 |
| NM_205382 | TEF | -0.11 | 0.16 | 8.29E-01 |
| NM_001193585 | MRPL45 | 0.10 | 0.15 | 8.29E-01 |
| NM_001005827 | RAB11A | -0.07 | 0.11 | 8.29E-01 |
| NM_001006252 | TAF13 | 0.09 | 0.14 | 8.29E-01 |
| NM_001006447 | COPS4 | 0.08 | 0.12 | 8.29E-01 |
| NM_001012535 | EIF3L | -0.06 | 0.09 | 8.29E-01 |
| NM_001012835 | GNB1 | -0.05 | 0.08 | 8.29E-01 |
| NM_001030622 | KIF3A | -0.07 | 0.11 | 8.29E-01 |
| NM_001030942 | E2F5 | -0.07 | 0.11 | 8.29E-01 |
| NM_001031407 | PSMA5 | 0.10 | 0.15 | 8.29E-01 |
| NM_001031486 | MRPS6 | 0.10 | 0.16 | 8.29E-01 |
| NM_001039599 | COLEC12 | -0.11 | 0.17 | 8.29E-01 |
| NM_001040642 | HIST1H101 | -0.08 | 0.13 | 8.29E-01 |
| NM_001044674 | RP11-195F19.29 | 0.11 | 0.17 | 8.29E-01 |
| NM_001044677 | MIS12 | -0.09 | 0.14 | 8.29E-01 |
| NM_001079495 | ARPC1A | 0.08 | 0.12 | 8.29E-01 |
| NM_001167762 | ACYP2 | 0.11 | 0.17 | 8.29E-01 |
| NM_204544 | FET1 | 0.09 | 0.14 | 8.29E-01 |
| NM_204623 | TES | -0.09 | 0.14 | 8.29E-01 |
| NM_205144 | TOM1 | -0.09 | 0.13 | 8.29E-01 |
| NM_205179 | ALCAM | -0.07 | 0.10 | 8.29E-01 |
| NM_001006428 | CHMP1B | 0.06 | 0.10 | 8.30E-01 |
| NM_001006236 | SLC25A3 | 0.08 | 0.12 | 8.30E-01 |
| NM_001012898 | CPT1A | -0.09 | 0.14 | 8.30E-01 |
| NM_001079499 | ARCN1 | -0.06 | 0.09 | 8.30E-01 |
| NM_001252015 | LOC420849 | -0.06 | 0.09 | 8.30E-01 |
| NM_001270662 | RAB1A | 0.06 | 0.10 | 8.30E-01 |
| NM_001282320 | C9orf69 | 0.07 | 0.11 | 8.30E-01 |
| NM_205371 | CTSB | 0.07 | 0.10 | 8.31E-01 |
| NM_001006446 | FBXO8 | 0.09 | 0.14 | 8.31E-01 |
| NM_001277375 | GSTO1 | 0.10 | 0.16 | 8.31E-01 |
| NM_204124 | NR2C1 | 0.09 | 0.14 | 8.31E-01 |
| NM_205203 | B4GALT2 | -0.07 | 0.12 | 8.32E-01 |
| NM_001030907 | RCAN3 | -0.07 | 0.10 | 8.33E-01 |
| NM_001030765 | CHRM2 | -0.10 | 0.15 | 8.33E-01 |
| NM_001031087 | CLN8 | -0.08 | 0.12 | 8.33E-01 |
| NM_205349 | FYN | -0.11 | 0.18 | 8.33E-01 |
| NM_001007900 | YPEL5 | -0.06 | 0.09 | 8.34E-01 |
| NM_001277753 | UNC50 | 0.09 | 0.15 | 8.34E-01 |
| NM_001146044 | CDA | -0.08 | 0.12 | 8.34E-01 |
| NM_001199606 | XPOT | -0.07 | 0.12 | 8.34E-01 |
| NM_001039294 | FDFT1 | -0.10 | 0.15 | 8.34E-01 |
| NM_001006373 | BLOC1S5 | -0.05 | 0.08 | 8.34E-01 |
| NM_001006526 | SEC22A | 0.06 | 0.10 | 8.34E-01 |
| NM_001007925 | RNF141 | -0.05 | 0.08 | 8.34E-01 |
| NM_001031126 | NRK | -0.11 | 0.18 | 8.34E-01 |
| NM_204999 | RPL13 | 0.08 | 0.13 | 8.34E-01 |
| NM_001008789 | CYGB | 0.11 | 0.18 | 8.34E-01 |
| NM_001039287 | ACAT2 | 0.09 | 0.14 | 8.34E-01 |
| NM_204517 | SMC3 | 0.07 | 0.12 | 8.34E-01 |
| NM_001287205 | RPS27A | 0.10 | 0.16 | 8.34E-01 |
| NM_001030340 | PPP3CB | -0.09 | 0.15 | 8.35E-01 |
| NM_001007958 | LEPROT | -0.05 | 0.08 | 8.35E-01 |
| NM_001199395 | ABCD2 | 0.07 | 0.12 | 8.35E-01 |
| NM_205173 | RAD51 | 0.07 | 0.12 | 8.35E-01 |
| NM_205194 | ARHGAP26 | -0.06 | 0.10 | 8.35E-01 |
| NM_001142254 | DLK1 | -0.11 | 0.17 | 8.35E-01 |
| NM_001277871 | TANK | -0.07 | 0.11 | 8.35E-01 |
| NM_001031587 | IRF5 | 0.11 | 0.18 | 8.35E-01 |
| NM_001006513 | SLC19A1 | -0.08 | 0.12 | 8.35E-01 |
| NM_001007850 | YAF2 | 0.07 | 0.11 | 8.35E-01 |
| NM_001006385 | CNDP2 | -0.05 | 0.08 | 8.36E-01 |
| NM_001007965 | ENSA | -0.07 | 0.11 | 8.36E-01 |
| NM_001012561 | MYLIP | 0.09 | 0.14 | 8.36E-01 |
| NM_001030691 | SET | 0.06 | 0.10 | 8.36E-01 |
| NM_001030755 | LUC7L2 | 0.06 | 0.10 | 8.36E-01 |
| NM_001031160 | LCORL | 0.11 | 0.18 | 8.36E-01 |
| NM_001257258 | TSPAN3 | -0.08 | 0.13 | 8.36E-01 |
| NM_001277561 | TAF1A | 0.10 | 0.16 | 8.36E-01 |
| NM_204831 | HDAC2 | 0.06 | 0.10 | 8.36E-01 |
| NM_001039267 | EWSR1 | 0.06 | 0.10 | 8.37E-01 |
| NM_205044 | MGP | 0.11 | 0.18 | 8.37E-01 |
| NM_001278151 | SLCO4A1 | -0.11 | 0.18 | 8.37E-01 |
| NM_001012598 | ELOVL1 | 0.06 | 0.09 | 8.37E-01 |
| NM_001257369 | TP53I11 | -0.11 | 0.17 | 8.37E-01 |
| NM_001031271 | RGL1 | -0.08 | 0.13 | 8.38E-01 |
| NM_001199164 | DPM2 | 0.09 | 0.15 | 8.38E-01 |
| NM_204932 | KRT7 | -0.11 | 0.18 | 8.38E-01 |
| NM_001277888 | LOC100857579 | -0.06 | 0.09 | 8.38E-01 |
| NM_001031168 | TMEM129 | -0.07 | 0.12 | 8.39E-01 |
| NM_001031287 | CPT2 | 0.07 | 0.12 | 8.39E-01 |
| NM_001006393 | PSME4 | -0.11 | 0.18 | 8.39E-01 |
| NM_001302194 | PLAC9 | 0.11 | 0.18 | 8.39E-01 |
| NM_205300 | MYO5A | -0.11 | 0.17 | 8.39E-01 |
| NM_001080101 | SRGAP1 | -0.08 | 0.13 | 8.39E-01 |
| NM_001012951 | SNAP91 | -0.10 | 0.17 | 8.39E-01 |
| NM_001030903 | PPT1 | 0.06 | 0.10 | 8.39E-01 |
| NM_001113741 | SRSF1 | -0.06 | 0.10 | 8.39E-01 |
| NM_204259 | PTGDS | 0.11 | 0.18 | 8.39E-01 |
| NM_001006169 | NDE1 | 0.06 | 0.10 | 8.40E-01 |
| NM_001030846 | RALGAPB | -0.10 | 0.17 | 8.40E-01 |
| NM_001033058 | BRIP1 | -0.11 | 0.18 | 8.40E-01 |
| NM_001193624 | DAAM1 | 0.06 | 0.10 | 8.40E-01 |
| NM_001199421 | PEX13 | -0.06 | 0.09 | 8.40E-01 |
| NM_001277923 | RAB28 | -0.08 | 0.13 | 8.40E-01 |
| NM_001293242 | GNB5 | 0.07 | 0.12 | 8.40E-01 |
| NM_204789 | SOUL | -0.09 | 0.15 | 8.40E-01 |
| NM_001006190 | PPP1CC | 0.06 | 0.10 | 8.40E-01 |
| NM_001006259 | PDK3 | -0.05 | 0.09 | 8.40E-01 |
| NM_001006307 | ZNF593 | 0.08 | 0.13 | 8.40E-01 |
| NM_001007935 | BRMS1L | 0.06 | 0.10 | 8.40E-01 |
| NM_001030714 | ASL2 | 0.10 | 0.16 | 8.40E-01 |
| NM_001030921 | OARD1 | -0.08 | 0.13 | 8.40E-01 |
| NM_001030923 | TAF8 | -0.08 | 0.13 | 8.40E-01 |
| NM_001030941 | STAU2 | -0.11 | 0.18 | 8.40E-01 |
| NM_001031065 | C3H6ORF120 | 0.07 | 0.12 | 8.40E-01 |
| NM_001031606 | LOC431499 | -0.10 | 0.16 | 8.40E-01 |
| NM_001079504 | DCAF7 | -0.08 | 0.14 | 8.40E-01 |
| NM_001079737 | C20H20ORF24 | 0.06 | 0.10 | 8.40E-01 |
| NM_001171770 | NLGN4X | 0.11 | 0.18 | 8.40E-01 |
| NM_001177329 | LASP1 | -0.10 | 0.17 | 8.40E-01 |
| NM_001199555 | LRIG3 | -0.10 | 0.17 | 8.40E-01 |
| NM_001277125 | DHRS4 | 0.06 | 0.09 | 8.40E-01 |
| NM_001277412 | RMDN1 | 0.08 | 0.13 | 8.40E-01 |
| NM_204268 | NAB1 | -0.11 | 0.18 | 8.40E-01 |
| NM_204700 | CFC1B | 0.08 | 0.14 | 8.40E-01 |
| NM_204974 | ATP6V1A | -0.05 | 0.08 | 8.40E-01 |
| NM_205041 | OASL | -0.10 | 0.17 | 8.40E-01 |
| NM_205272 | NFIB | -0.06 | 0.11 | 8.40E-01 |
| NM_001030621 | SAR1B | 0.07 | 0.11 | 8.41E-01 |
| NM_001168384 | ANO5 | -0.06 | 0.11 | 8.41E-01 |
| NM_001001767 | RNF114 | -0.06 | 0.09 | 8.42E-01 |
| NM_001008468 | TMEM184C | -0.06 | 0.10 | 8.42E-01 |
| NM_001012915 | ANKRD44 | -0.08 | 0.13 | 8.42E-01 |
| NM_001030563 | SETD6 | -0.06 | 0.10 | 8.42E-01 |
| NM_001030727 | DLD | -0.06 | 0.10 | 8.42E-01 |
| NM_204165 | FBLN1 | 0.09 | 0.15 | 8.42E-01 |
| NM_205435 | PTK2 | -0.10 | 0.18 | 8.42E-01 |
| NM_205524 | PAICS | 0.06 | 0.10 | 8.42E-01 |
| NM_001012947 | XPO4 | -0.09 | 0.16 | 8.42E-01 |
| NM_001006441 | PGRMC2 | 0.08 | 0.13 | 8.42E-01 |
| NM_001012893 | MAVS | -0.07 | 0.12 | 8.42E-01 |
| NM_001080107 | CHEK2 | 0.08 | 0.14 | 8.42E-01 |
| NM_001177357 | ARHGAP21 | -0.07 | 0.12 | 8.42E-01 |
| NM_205360 | ALPL | -0.10 | 0.16 | 8.42E-01 |
| NM_001031114 | CLIC2 | -0.07 | 0.12 | 8.43E-01 |
| NM_001199443 | CDC42BPA | 0.08 | 0.13 | 8.43E-01 |
| NM_001286261 | LDB3 | 0.08 | 0.14 | 8.43E-01 |
| NM_204500 | OAF | -0.08 | 0.13 | 8.43E-01 |
| NM_001006214 | PRKRIP1 | 0.09 | 0.16 | 8.43E-01 |
| NM_001006538 | DPH2 | -0.08 | 0.14 | 8.44E-01 |
| NM_001008467 | ANAPC10 | -0.08 | 0.13 | 8.44E-01 |
| NM_001025440 | CHMP1A | 0.06 | 0.11 | 8.44E-01 |
| NM_001030813 | C1H2ORF49 | 0.06 | 0.11 | 8.44E-01 |
| NM_001031406 | STUB1 | -0.06 | 0.11 | 8.44E-01 |
| NM_001033871 | ARFGAP2 | -0.05 | 0.08 | 8.44E-01 |
| NM_001114629 | MRPL38 | -0.07 | 0.12 | 8.44E-01 |
| NM_001305034 | RNF152 | -0.10 | 0.18 | 8.44E-01 |
| NM_001305149 | RP11-77K12.7 | -0.06 | 0.10 | 8.44E-01 |
| NM_001305160 | SORL1 | -0.06 | 0.11 | 8.44E-01 |
| NM_204336 | WNT2B | -0.10 | 0.17 | 8.44E-01 |
| NM_001030342 | IGF2 | -0.10 | 0.18 | 8.44E-01 |
| NM_204262 | PER2 | -0.08 | 0.14 | 8.44E-01 |
| NM_001031529 | CHMP6 | 0.08 | 0.13 | 8.45E-01 |
| NM_001006314 | YARS | 0.06 | 0.10 | 8.46E-01 |
| NM_001012839 | KCTD9 | -0.07 | 0.11 | 8.46E-01 |
| NM_001097532 | ATP6V0E1 | 0.09 | 0.15 | 8.46E-01 |
| NM_204756 | MAFK | 0.10 | 0.17 | 8.46E-01 |
| NM_001006135 | WDR82 | -0.05 | 0.10 | 8.46E-01 |
| NM_001277993 | SCAMP1 | -0.10 | 0.18 | 8.46E-01 |
| NM_206991 | ADIPOQ | 0.11 | 0.18 | 8.46E-01 |
| NM_001159347 | NAV3 | -0.10 | 0.17 | 8.47E-01 |
| NM_001001733 | CACNB4 | -0.10 | 0.17 | 8.48E-01 |
| NM_001006306 | CHMP7 | -0.06 | 0.11 | 8.48E-01 |
| NM_001006493 | POLE2 | 0.05 | 0.09 | 8.48E-01 |
| NM_001030544 | NRG4 | 0.08 | 0.14 | 8.48E-01 |
| NM_001030570 | N4BP1 | -0.06 | 0.10 | 8.48E-01 |
| NM_001030664 | NAA25 | -0.08 | 0.15 | 8.48E-01 |
| NM_001031226 | PPIF | 0.07 | 0.12 | 8.48E-01 |
| NM_001031592 | PELO | 0.06 | 0.11 | 8.48E-01 |
| NM_001044678 | GADD45A | -0.08 | 0.13 | 8.48E-01 |
| NM_001162406 | PLTP | 0.08 | 0.13 | 8.48E-01 |
| NM_001199576 | HHATL | -0.10 | 0.17 | 8.48E-01 |
| NM_001278060 | CHTF18 | -0.06 | 0.11 | 8.48E-01 |
| NM_001293220 | ZNF609 | -0.07 | 0.13 | 8.48E-01 |
| NM_001293321 | SNX20 | 0.08 | 0.14 | 8.48E-01 |
| NM_001302202 | CDC26 | 0.08 | 0.15 | 8.48E-01 |
| NM_204109 | PALD1 | -0.07 | 0.12 | 8.48E-01 |
| NM_204626 | CISH | -0.10 | 0.18 | 8.48E-01 |
| NM_204945 | PPP1R3E | -0.05 | 0.09 | 8.48E-01 |
| NM_001277541 | FAM192A | 0.06 | 0.11 | 8.48E-01 |
| NM_001012861 | RBFA | 0.08 | 0.15 | 8.49E-01 |
| NM_001040018 | OLFML3 | 0.07 | 0.12 | 8.49E-01 |
| NM_001277459 | HYKK | -0.08 | 0.14 | 8.49E-01 |
| NM_001277637 | MED9 | 0.09 | 0.16 | 8.49E-01 |
| NM_205454 | TGFB3 | 0.07 | 0.13 | 8.49E-01 |
| NM_001007873 | SPATA2 | -0.08 | 0.14 | 8.49E-01 |
| NM_001031169 | RNF103 | -0.06 | 0.11 | 8.49E-01 |
| NM_001079722 | DNM1L | -0.07 | 0.12 | 8.49E-01 |
| NM_205310 | CKB | 0.09 | 0.16 | 8.49E-01 |
| NM_001031268 | FAM20B | -0.05 | 0.10 | 8.50E-01 |
| NM_001039913 | MRPS26 | 0.08 | 0.14 | 8.50E-01 |
| NM_001199314 | ANKRD16 | -0.10 | 0.18 | 8.50E-01 |
| NM_001031033 | XRN2 | -0.05 | 0.09 | 8.50E-01 |
| NM_001029981 | OSER1 | 0.07 | 0.13 | 8.51E-01 |
| NM_001030715 | SMYD4 | -0.06 | 0.11 | 8.51E-01 |
| NM_001030725 | DDX52 | 0.07 | 0.13 | 8.51E-01 |
| NM_001039311 | COPS5 | 0.07 | 0.12 | 8.51E-01 |
| NM_001170344 | OSR2 | 0.09 | 0.16 | 8.51E-01 |
| NM_205282 | LPL | 0.10 | 0.18 | 8.52E-01 |
| NM_001004386 | ZYX | -0.07 | 0.12 | 8.53E-01 |
| NM_001006410 | PREP | -0.06 | 0.11 | 8.53E-01 |
| NM_001007848 | CBLL1 | -0.07 | 0.13 | 8.53E-01 |
| NM_001008459 | BTBD9 | -0.10 | 0.18 | 8.53E-01 |
| NM_001008472 | GLRX5 | 0.06 | 0.10 | 8.53E-01 |
| NM_001012690 | TIPIN | 0.07 | 0.12 | 8.53E-01 |
| NM_001012934 | PSMD2 | -0.06 | 0.10 | 8.53E-01 |
| NM_001031225 | UNC5B | -0.08 | 0.14 | 8.53E-01 |
| NM_001031320 | TRIM59 | -0.07 | 0.12 | 8.53E-01 |
| NM_001031426 | OSTF1 | -0.07 | 0.13 | 8.53E-01 |
| NM_001199410 | MKKS | 0.09 | 0.15 | 8.53E-01 |
| NM_001282522 | CHST6 | 0.06 | 0.11 | 8.53E-01 |
| NM_204245 | CRY1 | -0.08 | 0.14 | 8.53E-01 |
| NM_204885 | EZR | 0.09 | 0.16 | 8.53E-01 |
| NM_001030829 | NUP58 | 0.05 | 0.09 | 8.53E-01 |
| NM_001031254 | OLA1 | 0.06 | 0.11 | 8.53E-01 |
| NM_001199405 | TEAD1 | -0.10 | 0.18 | 8.53E-01 |
| NM_001031104 | MTR | -0.07 | 0.13 | 8.53E-01 |
| NM_001039277 | SRPR | -0.05 | 0.10 | 8.53E-01 |
| NM_001077234 | TSC22D3 | 0.09 | 0.17 | 8.53E-01 |
| NM_001293219 | RP11-152F13.10 | 0.08 | 0.15 | 8.53E-01 |
| NM_204479 | ST3GAL6 | -0.08 | 0.14 | 8.53E-01 |
| NM_001012545 | GPM6B | -0.10 | 0.18 | 8.53E-01 |
| NM_001006287 | IFT52 | 0.08 | 0.14 | 8.53E-01 |
| NM_001006324 | SIKE1 | 0.06 | 0.12 | 8.53E-01 |
| NM_001006572 | GLYR1 | -0.09 | 0.17 | 8.53E-01 |
| NM_001012604 | MAEA | 0.05 | 0.10 | 8.53E-01 |
| NM_001029849 | RHOC | 0.07 | 0.12 | 8.53E-01 |
| NM_001030908 | FLI1 | 0.05 | 0.09 | 8.53E-01 |
| NM_001031039 | LCLAT1 | -0.07 | 0.13 | 8.53E-01 |
| NM_001031040 | EHD3 | 0.06 | 0.11 | 8.53E-01 |
| NM_001031449 | BRIX1 | 0.07 | 0.13 | 8.53E-01 |
| NM_001031455 | CCPG1 | -0.07 | 0.12 | 8.53E-01 |
| NM_001031594 | DCLRE1C | -0.08 | 0.14 | 8.53E-01 |
| NM_001193575 | KPNA3 | -0.05 | 0.09 | 8.53E-01 |
| NM_001199448 | ROCK1 | -0.06 | 0.12 | 8.53E-01 |
| NM_001199705 | MPP5 | -0.10 | 0.18 | 8.53E-01 |
| NM_001277414 | MRPL44 | 0.08 | 0.15 | 8.53E-01 |
| NM_001277515 | NIPSNAP1 | -0.06 | 0.11 | 8.53E-01 |
| NM_001277641 | ZBTB6 | -0.07 | 0.13 | 8.53E-01 |
| NM_001277728 | RPL8 | 0.07 | 0.13 | 8.53E-01 |
| NM_001302145 | MANBAL | -0.10 | 0.18 | 8.53E-01 |
| NM_204435 | ALG6 | 0.08 | 0.15 | 8.53E-01 |
| NM_204810 | LECT1 | 0.08 | 0.15 | 8.53E-01 |
| NM_205009 | KRT19 | -0.10 | 0.18 | 8.53E-01 |
| NM_205292 | HRAS | 0.05 | 0.09 | 8.53E-01 |
| NM_205423 | PLA2G4A | -0.09 | 0.17 | 8.53E-01 |
| NM_001030571 | UBA2 | 0.05 | 0.10 | 8.54E-01 |
| NM_001030604 | SRGAP3 | -0.10 | 0.18 | 8.54E-01 |
| NM_001305075 | FAM195B | 0.08 | 0.14 | 8.54E-01 |
| NM_001030745 | ZDHHC17 | -0.07 | 0.13 | 8.54E-01 |
| NM_001001755 | THBS2 | 0.08 | 0.14 | 8.54E-01 |
| NM_001006546 | CCDC61 | -0.06 | 0.12 | 8.54E-01 |
| NM_001030657 | HIP1R | -0.07 | 0.13 | 8.54E-01 |
| NM_001030984 | NUPL2 | 0.06 | 0.12 | 8.54E-01 |
| NM_001031313 | PPP1R2 | 0.07 | 0.13 | 8.54E-01 |
| NM_001079734 | STXBP3 | 0.08 | 0.14 | 8.54E-01 |
| NM_001135786 | CDK5 | -0.08 | 0.15 | 8.54E-01 |
| NM_001030611 | THOC3 | 0.09 | 0.17 | 8.55E-01 |
| NM_001252158 | STX18 | 0.06 | 0.12 | 8.55E-01 |
| NM_001006354 | GTPBP4 | 0.07 | 0.13 | 8.56E-01 |
| NM_001204097 | ANKHD1 | -0.05 | 0.10 | 8.56E-01 |
| NM_205004 | GDI2 | -0.05 | 0.09 | 8.56E-01 |
| NM_001001328 | RP11-196G11.1 | 0.08 | 0.14 | 8.56E-01 |
| NM_001005825 | VWA9 | -0.05 | 0.10 | 8.56E-01 |
| NM_001005841 | DNAJA2 | 0.05 | 0.10 | 8.56E-01 |
| NM_001006220 | MYO1C | -0.10 | 0.18 | 8.56E-01 |
| NM_001006273 | SPRYD7 | 0.06 | 0.11 | 8.56E-01 |
| NM_001006366 | MRPL3 | 0.06 | 0.11 | 8.56E-01 |
| NM_001006387 | TCEA1 | 0.08 | 0.15 | 8.56E-01 |
| NM_001006465 | NADSYN1 | 0.07 | 0.14 | 8.56E-01 |
| NM_001006547 | BDH1 | -0.05 | 0.10 | 8.56E-01 |
| NM_001008469 | TMEM41B | -0.08 | 0.14 | 8.56E-01 |
| NM_001012558 | TMEM106B | -0.07 | 0.13 | 8.56E-01 |
| NM_001012870 | BUB1 | -0.05 | 0.10 | 8.56E-01 |
| NM_001012931 | MFN1 | -0.06 | 0.10 | 8.56E-01 |
| NM_001030615 | CLINT1 | 0.06 | 0.11 | 8.56E-01 |
| NM_001031385 | HNRPK | 0.05 | 0.10 | 8.56E-01 |
| NM_001031507 | TMEM65 | 0.05 | 0.09 | 8.56E-01 |
| NM_001044658 | ITGB3BP | -0.09 | 0.17 | 8.56E-01 |
| NM_001044669 | ERBB3 | -0.08 | 0.15 | 8.56E-01 |
| NM_001198639 | DCTN5 | -0.09 | 0.16 | 8.56E-01 |
| NM_001199437 | ZC3HC1 | -0.06 | 0.11 | 8.56E-01 |
| NM_001199471 | PRMT3 | 0.07 | 0.12 | 8.56E-01 |
| NM_001199529 | CISD1 | -0.06 | 0.12 | 8.56E-01 |
| NM_001252324 | FNDC3B | -0.10 | 0.18 | 8.56E-01 |
| NM_001277457 | RPL35A | 0.07 | 0.14 | 8.56E-01 |
| NM_001277639 | ZBTB26 | -0.09 | 0.17 | 8.56E-01 |
| NM_001277678 | CIDEC | -0.08 | 0.15 | 8.56E-01 |
| NM_001278029 | ERICH1 | 0.09 | 0.16 | 8.56E-01 |
| NM_001278030 | SAMM50 | 0.06 | 0.11 | 8.56E-01 |
| NM_001293322 | IPPK | -0.07 | 0.12 | 8.56E-01 |
| NM_001302106 | DPH3 | 0.07 | 0.12 | 8.56E-01 |
| NM_001305385 | HIST1H103 | 0.09 | 0.17 | 8.56E-01 |
| NM_204410 | SPARC | -0.09 | 0.17 | 8.56E-01 |
| NM_204439 | TNFRSF1B | -0.06 | 0.11 | 8.56E-01 |
| NM_204677 | BAZ2B | -0.05 | 0.10 | 8.56E-01 |
| NM_204902 | HMGB1 | 0.05 | 0.10 | 8.56E-01 |
| NM_205017 | RAC1 | -0.06 | 0.11 | 8.56E-01 |
| NM_205221 | CNGA3 | -0.09 | 0.18 | 8.56E-01 |
| NM_001006330 | LIMD2 | 0.08 | 0.15 | 8.57E-01 |
| NM_001031319 | PRKCI | -0.05 | 0.10 | 8.57E-01 |
| NM_001006155 | P4HA2 | 0.08 | 0.15 | 8.57E-01 |
| NM_001030936 | WDR18 | 0.07 | 0.14 | 8.57E-01 |
| NM_001135558 | MSRB1 | -0.07 | 0.14 | 8.57E-01 |
| NM_001282178 | CEP112 | -0.07 | 0.13 | 8.57E-01 |
| NM_001006469 | NAP1L4 | 0.05 | 0.10 | 8.57E-01 |
| NM_001006494 | PSMC6 | 0.06 | 0.12 | 8.57E-01 |
| NM_001007930 | CHP1 | -0.05 | 0.09 | 8.57E-01 |
| NM_001030779 | TNFRSF1A | -0.07 | 0.13 | 8.57E-01 |
| NM_204962 | CAMLG | -0.09 | 0.17 | 8.58E-01 |
| NM_001012803 | SCPEP1 | 0.05 | 0.10 | 8.58E-01 |
| NM_205010 | PITX2 | -0.09 | 0.17 | 8.58E-01 |
| NM_205216 | WT1 | -0.09 | 0.18 | 8.58E-01 |
| NM_001293088 | ZFHX3 | -0.07 | 0.13 | 8.58E-01 |
| NM_001199386 | GINM1 | 0.06 | 0.11 | 8.59E-01 |
| NM_001006512 | UBE2F | 0.07 | 0.14 | 8.59E-01 |
| NM_001008442 | SLC25A15 | -0.04 | 0.09 | 8.59E-01 |
| NM_001199590 | RPP38 | 0.07 | 0.14 | 8.59E-01 |
| NM_001199749 | SEMA7A | -0.08 | 0.15 | 8.59E-01 |
| NM_204702 | NR1I3 | -0.08 | 0.16 | 8.59E-01 |
| NM_001006559 | LAMTOR3 | 0.05 | 0.10 | 8.59E-01 |
| NM_001007960 | SLC25A36 | -0.06 | 0.11 | 8.59E-01 |
| NM_001031145 | AGPAT9 | 0.06 | 0.12 | 8.59E-01 |
| NM_001039256 | EFTUD1 | 0.06 | 0.12 | 8.59E-01 |
| NM_001130739 | CECR2 | 0.09 | 0.17 | 8.59E-01 |
| NM_001271932 | PRDX1 | 0.07 | 0.13 | 8.59E-01 |
| NM_001007894 | CMTM7 | 0.07 | 0.13 | 8.59E-01 |
| NM_001031111 | PDZD11 | 0.06 | 0.12 | 8.59E-01 |
| NM_001031296 | SRSF11 | 0.06 | 0.11 | 8.59E-01 |
| NM_001293305 | KIF5C | 0.06 | 0.13 | 8.59E-01 |
| NM_204385 | RGS4 | 0.09 | 0.17 | 8.59E-01 |
| NM_204537 | RGMA | 0.09 | 0.18 | 8.59E-01 |
| NM_204750 | TDG | -0.05 | 0.10 | 8.59E-01 |
| NM_001006288 | RPN2 | 0.05 | 0.10 | 8.59E-01 |
| NM_001006310 | ZDHHC18 | -0.09 | 0.18 | 8.59E-01 |
| NM_001006411 | LOC421792 | 0.08 | 0.16 | 8.59E-01 |
| NM_001007944 | KIF1BP | 0.05 | 0.09 | 8.59E-01 |
| NM_001030762 | TXNRD1 | -0.07 | 0.14 | 8.59E-01 |
| NM_204142 | VAV2 | -0.08 | 0.15 | 8.59E-01 |
| NM_204147 | MATR3 | 0.06 | 0.12 | 8.59E-01 |
| NM_204871 | IL6ST | -0.09 | 0.18 | 8.59E-01 |
| NM_205326 | RARB | -0.08 | 0.16 | 8.59E-01 |
| NM_204876 | ENO2 | 0.08 | 0.15 | 8.59E-01 |
| NM_001012831 | STAU1 | -0.04 | 0.08 | 8.59E-01 |
| NM_001012854 | VPS50 | -0.06 | 0.11 | 8.60E-01 |
| NM_001024577 | LINGO1 | -0.07 | 0.14 | 8.60E-01 |
| NM_001030988 | NGLY1 | -0.06 | 0.12 | 8.60E-01 |
| NM_001031408 | DDA1 | -0.06 | 0.11 | 8.60E-01 |
| NM_001040465 | DICER1 | 0.07 | 0.14 | 8.60E-01 |
| NM_001097531 | ZNF706 | -0.06 | 0.12 | 8.60E-01 |
| NM_001197037 | PPDPF | 0.09 | 0.17 | 8.60E-01 |
| NM_001276338 | CDCA9 | 0.08 | 0.16 | 8.60E-01 |
| NM_204984 | PXN | -0.08 | 0.17 | 8.60E-01 |
| NM_001006548 | ACAP2 | -0.05 | 0.10 | 8.60E-01 |
| NM_001006161 | CARD11 | 0.08 | 0.16 | 8.60E-01 |
| NM_001159363 | ABLIM1 | -0.09 | 0.17 | 8.62E-01 |
| NM_001006391 | YTHDF3 | 0.06 | 0.11 | 8.62E-01 |
| NM_001005842 | VPS35 | 0.04 | 0.08 | 8.62E-01 |
| NM_001012848 | TMEM70 | 0.05 | 0.11 | 8.63E-01 |
| NM_001012857 | KLHL7 | -0.08 | 0.16 | 8.64E-01 |
| NM_204551 | INVS | -0.08 | 0.17 | 8.65E-01 |
| NM_001079713 | STX2 | 0.04 | 0.08 | 8.65E-01 |
| NM_001199403 | ERGIC2 | 0.04 | 0.08 | 8.65E-01 |
| NM_204898 | TK1 | -0.08 | 0.15 | 8.65E-01 |
| NR_035298 | MIR1797 | 0.08 | 0.16 | 8.65E-01 |
| NM_001006371 | SLC12A7 | -0.05 | 0.11 | 8.65E-01 |
| NM_001030850 | NECAB3 | -0.07 | 0.13 | 8.65E-01 |
| NM_001030969 | KLF6 | 0.05 | 0.11 | 8.65E-01 |
| NM_001278454 | CHD2 | -0.05 | 0.09 | 8.65E-01 |
| NM_001006571 | ACAA2 | 0.05 | 0.10 | 8.65E-01 |
| NM_001031003 | DAP | 0.05 | 0.11 | 8.65E-01 |
| NM_205355 | RNF13 | 0.04 | 0.08 | 8.65E-01 |
| NM_001004401 | PPAT | -0.04 | 0.09 | 8.65E-01 |
| NM_001012829 | UBL3 | 0.05 | 0.10 | 8.65E-01 |
| NM_204857 | AP000295.9 | -0.07 | 0.14 | 8.65E-01 |
| NM_001001201 | HSD11B1L | 0.06 | 0.13 | 8.66E-01 |
| NM_001012825 | RASA3 | -0.06 | 0.11 | 8.66E-01 |
| NM_001031301 | SAP130 | -0.05 | 0.10 | 8.66E-01 |
| NR_035048 | MIR1564 | 0.09 | 0.17 | 8.66E-01 |
| NM_001001465 | BMPR2 | -0.09 | 0.18 | 8.66E-01 |
| NM_001006178 | SNX5 | -0.08 | 0.17 | 8.66E-01 |
| NM_001006489 | XRCC3 | 0.06 | 0.11 | 8.66E-01 |
| NM_001030592 | ATG7 | 0.07 | 0.13 | 8.66E-01 |
| NM_001031148 | METTL14 | 0.04 | 0.09 | 8.66E-01 |
| NM_001031161 | TAPT1 | 0.05 | 0.10 | 8.66E-01 |
| NM_204847 | FKBP9 | -0.05 | 0.10 | 8.66E-01 |
| NM_001012783 | KIF20A | 0.05 | 0.11 | 8.67E-01 |
| NM_001006149 | TIMD4 | -0.08 | 0.17 | 8.67E-01 |
| NM_205511 | SLC2A3 | -0.08 | 0.17 | 8.67E-01 |
| NM_001199220 | LONP2 | -0.05 | 0.11 | 8.68E-01 |
| NM_001198857 | CNEP1R1 | -0.05 | 0.10 | 8.68E-01 |
| NM_001257286 | SUPT4H1 | 0.08 | 0.16 | 8.68E-01 |
| NM_205285 | LMNB2 | -0.07 | 0.14 | 8.69E-01 |
| NM_001012902 | ARID4A | 0.07 | 0.15 | 8.70E-01 |
| NM_001293222 | TARSL2 | 0.05 | 0.11 | 8.70E-01 |
| NM_001006217 | PHKG1 | -0.06 | 0.13 | 8.70E-01 |
| NM_001252162 | CDKN3 | 0.06 | 0.12 | 8.70E-01 |
| NM_001277666 | LOC100858381 | -0.08 | 0.17 | 8.72E-01 |
| NM_204298 | TIMP2 | -0.05 | 0.10 | 8.73E-01 |
| NM_204538 | SDK2 | 0.08 | 0.16 | 8.73E-01 |
| NM_001012565 | DEGS1 | -0.05 | 0.11 | 8.73E-01 |
| NM_001006427 | ENOX2 | -0.04 | 0.09 | 8.75E-01 |
| NM_001030390 | RP11-106M3.2 | 0.05 | 0.10 | 8.75E-01 |
| NM_001030645 | NMRAL1 | 0.07 | 0.15 | 8.75E-01 |
| NM_001031391 | ATP5B | -0.05 | 0.11 | 8.75E-01 |
| NM_001031421 | HAUS6 | 0.04 | 0.09 | 8.75E-01 |
| NM_001080883 | UBE2G1 | -0.05 | 0.10 | 8.75E-01 |
| NM_001257203 | TIA1 | -0.06 | 0.12 | 8.75E-01 |
| NM_204313 | HDAC4 | 0.08 | 0.17 | 8.75E-01 |
| NM_205048 | CDC42 | 0.05 | 0.10 | 8.75E-01 |
| NM_001302196 | PIGY | -0.07 | 0.14 | 8.75E-01 |
| NM_001030955 | RNF139 | -0.04 | 0.09 | 8.75E-01 |
| NM_001033006 | CNIH1 | 0.04 | 0.09 | 8.75E-01 |
| NM_001270716 | DERL3 | 0.06 | 0.14 | 8.75E-01 |
| NM_001012551 | RAB11B | -0.04 | 0.08 | 8.76E-01 |
| NM_001031438 | ZDHHC21 | -0.07 | 0.14 | 8.76E-01 |
| NM_001195550 | AKAP17A | 0.06 | 0.13 | 8.76E-01 |
| NM_001305135 | USP37 | -0.08 | 0.18 | 8.76E-01 |
| NM_204295 | CAMK2A | 0.08 | 0.16 | 8.76E-01 |
| NM_204649 | CD4 | -0.08 | 0.17 | 8.76E-01 |
| NM_204300 | ENAH | 0.08 | 0.18 | 8.76E-01 |
| NM_204419 | RB1 | -0.06 | 0.14 | 8.76E-01 |
| NM_001031042 | LIN9 | 0.05 | 0.10 | 8.76E-01 |
| NM_206906 | ANXA1 | 0.07 | 0.15 | 8.76E-01 |
| NM_001030959 | JMJD4 | -0.06 | 0.13 | 8.77E-01 |
| NM_001031525 | PCMT1 | 0.05 | 0.11 | 8.78E-01 |
| NM_001005833 | CFAP20 | 0.07 | 0.15 | 8.78E-01 |
| NM_001008675 | MRPL51 | 0.07 | 0.15 | 8.78E-01 |
| NM_001012541 | NUP50 | 0.05 | 0.10 | 8.78E-01 |
| NM_001030568 | LPCAT2 | -0.06 | 0.12 | 8.78E-01 |
| NM_001030590 | BAP1 | -0.07 | 0.14 | 8.78E-01 |
| NM_001030713 | GBAS | 0.05 | 0.10 | 8.78E-01 |
| NM_001031328 | KIAA0907 | -0.04 | 0.09 | 8.78E-01 |
| NM_001039312 | KIF2A | 0.04 | 0.09 | 8.78E-01 |
| NM_001080211 | CRTAC1 | 0.08 | 0.18 | 8.78E-01 |
| NM_001198642 | PEBP1 | 0.05 | 0.11 | 8.78E-01 |
| NM_001278147 | GLB1 | -0.05 | 0.10 | 8.78E-01 |
| NM_001292071 | PYCR2 | -0.05 | 0.11 | 8.78E-01 |
| NM_204125 | QSOX1 | -0.05 | 0.11 | 8.78E-01 |
| NM_205027 | TMOD1 | -0.05 | 0.10 | 8.78E-01 |
| NM_205205 | NR1D2 | -0.07 | 0.16 | 8.78E-01 |
| NM_001006417 | KLF11 | 0.05 | 0.12 | 8.79E-01 |
| NM_001012780 | RBM5 | -0.06 | 0.12 | 8.79E-01 |
| NM_001031602 | METTL16 | 0.05 | 0.10 | 8.79E-01 |
| NM_001286225 | TCAIM | 0.05 | 0.11 | 8.79E-01 |
| NM_001006148 | BRD8 | -0.05 | 0.10 | 8.79E-01 |
| NM_001031281 | MMACHC | 0.08 | 0.18 | 8.79E-01 |
| NM_001080885 | PHB2 | -0.05 | 0.10 | 8.79E-01 |
| NM_001005822 | LRRC28 | 0.05 | 0.11 | 8.80E-01 |
| NM_001252031 | RP11-724O16.1 | -0.06 | 0.13 | 8.80E-01 |
| NM_205164 | NKX2-5 | 0.07 | 0.16 | 8.80E-01 |
| NM_001031241 | FAM175B | 0.04 | 0.10 | 8.80E-01 |
| NM_001031392 | SARS | -0.06 | 0.13 | 8.80E-01 |
| NM_001001778 | CMTM3 | -0.04 | 0.10 | 8.81E-01 |
| NM_001012528 | TRAF7 | -0.04 | 0.10 | 8.81E-01 |
| NM_001030960 | KLHL18 | -0.06 | 0.13 | 8.81E-01 |
| NM_001031165 | SH3BP2 | -0.07 | 0.16 | 8.81E-01 |
| NM_001031413 | DOHH | -0.07 | 0.15 | 8.81E-01 |
| NM_001031599 | IDH2 | -0.05 | 0.11 | 8.81E-01 |
| NM_001039271 | CPSF6 | -0.05 | 0.10 | 8.81E-01 |
| NM_001277788 | SYNJ2BP | 0.05 | 0.11 | 8.81E-01 |
| NM_001278120 | REXO2 | 0.06 | 0.13 | 8.81E-01 |
| NM_001305439 | SLC38A2 | 0.07 | 0.15 | 8.81E-01 |
| NM_001031329 | KLHL20 | -0.05 | 0.12 | 8.82E-01 |
| NM_204131 | LIMK1 | -0.06 | 0.13 | 8.82E-01 |
| NM_205515 | CAPZA1 | 0.04 | 0.09 | 8.82E-01 |
| NM_001031264 | NEK7 | 0.04 | 0.09 | 8.83E-01 |
| NM_001012888 | GTF2E2 | 0.05 | 0.12 | 8.84E-01 |
| NM_205174 | SKIL | -0.08 | 0.18 | 8.84E-01 |
| NM_001006476 | SRSF5A | 0.05 | 0.12 | 8.84E-01 |
| NM_001031265 | CDC73 | 0.04 | 0.09 | 8.84E-01 |
| NM_001031477 | YWHAG | -0.05 | 0.11 | 8.84E-01 |
| NM_001006165 | PRPSAP2 | 0.05 | 0.11 | 8.84E-01 |
| NM_001006534 | SH3GLB1 | 0.04 | 0.09 | 8.84E-01 |
| NM_001030859 | NSFL1C | -0.05 | 0.12 | 8.84E-01 |
| NM_001103201 | UFL1 | 0.05 | 0.11 | 8.84E-01 |
| NM_001199393 | CEP41 | 0.06 | 0.13 | 8.84E-01 |
| NM_001282200 | LOC418811 | 0.07 | 0.17 | 8.84E-01 |
| NM_204171 | CHRDL1 | 0.08 | 0.17 | 8.84E-01 |
| NM_001006444 | DCTD | 0.05 | 0.12 | 8.84E-01 |
| NM_001030550 | ITGB1BP3 | -0.06 | 0.14 | 8.84E-01 |
| NM_001031393 | KNSTRN | 0.06 | 0.15 | 8.84E-01 |
| NM_001278110 | CREBL2 | -0.06 | 0.14 | 8.84E-01 |
| NM_204286 | ATP5A1 | 0.04 | 0.10 | 8.84E-01 |
| NM_001031014 | FAM210A | 0.04 | 0.09 | 8.84E-01 |
| NM_001030696 | NDEL1 | -0.06 | 0.13 | 8.85E-01 |
| NM_001302117 | CDC42SE2 | 0.04 | 0.09 | 8.85E-01 |
| NM_001307997 | USP12P1 | 0.05 | 0.12 | 8.85E-01 |
| NM_001031383 | PGM2 | 0.04 | 0.10 | 8.85E-01 |
| NM_001001531 | FUS | -0.06 | 0.13 | 8.85E-01 |
| NM_001030817 | STK24 | -0.06 | 0.13 | 8.85E-01 |
| NM_001031021 | TMEM68 | -0.06 | 0.13 | 8.85E-01 |
| NM_001039281 | ABI1 | 0.04 | 0.08 | 8.85E-01 |
| NM_001277784 | HPGD | 0.07 | 0.16 | 8.85E-01 |
| NM_001281509 | HIST1H3H | -0.08 | 0.18 | 8.85E-01 |
| NM_001305099 | HENMT1 | -0.08 | 0.18 | 8.85E-01 |
| NM_204252 | FLT1 | 0.07 | 0.16 | 8.85E-01 |
| NM_205374 | PKIA | 0.08 | 0.18 | 8.85E-01 |
| NM_205458 | SNAP25 | 0.08 | 0.18 | 8.85E-01 |
| NM_206874 | RYR3 | 0.08 | 0.17 | 8.85E-01 |
| NM_001006440 | ABCE1 | 0.04 | 0.09 | 8.86E-01 |
| NM_204709 | CHURC1 | 0.07 | 0.16 | 8.86E-01 |
| NM_001006498 | SUPV3L1 | -0.03 | 0.08 | 8.87E-01 |
| NM_001277615 | DYNLRB2 | 0.08 | 0.18 | 8.87E-01 |
| NM_205518 | ACTB | 0.05 | 0.11 | 8.88E-01 |
| NM_001031054 | CRIPT | 0.04 | 0.09 | 8.88E-01 |
| NM_001004388 | CD47 | -0.05 | 0.11 | 8.89E-01 |
| NM_001006364 | CNOT10 | -0.04 | 0.10 | 8.89E-01 |
| NM_001012622 | GABPB1 | 0.05 | 0.12 | 8.89E-01 |
| NM_001031291 | PDE4B | -0.04 | 0.09 | 8.89E-01 |
| NM_001277702 | C1QTNF2 | 0.06 | 0.14 | 8.89E-01 |
| NM_205131 | ZEB1 | 0.06 | 0.13 | 8.89E-01 |
| NM_001012912 | MCMBP | -0.03 | 0.08 | 8.89E-01 |
| NM_001006347 | OTUD6B | 0.04 | 0.10 | 8.89E-01 |
| NM_001031012 | TUBB6 | 0.05 | 0.12 | 8.89E-01 |
| NM_001039309 | OPA1 | -0.04 | 0.09 | 8.89E-01 |
| NM_001199401 | POLR3F | 0.06 | 0.14 | 8.89E-01 |
| NM_001277735 | CD24 | -0.07 | 0.17 | 8.89E-01 |
| NM_001278088 | AASDHPPT | 0.08 | 0.18 | 8.89E-01 |
| NM_001282289 | WRN | -0.05 | 0.12 | 8.89E-01 |
| NM_001293169 | MCM8 | -0.05 | 0.11 | 8.89E-01 |
| NM_204718 | CITED4 | 0.06 | 0.13 | 8.89E-01 |
| NM_001012828 | N4BP2L2 | 0.07 | 0.16 | 8.90E-01 |
| NM_001031125 | Sep-06 | 0.05 | 0.12 | 8.91E-01 |
| NM_001006322 | EIF2D | -0.06 | 0.13 | 8.92E-01 |
| NM_001199574 | CYB561A3 | 0.07 | 0.16 | 8.92E-01 |
| NM_001256970 | ARL8BL | 0.07 | 0.17 | 8.92E-01 |
| NM_205439 | ITGAV | -0.08 | 0.18 | 8.92E-01 |
| NM_001030845 | SAMHD1 | -0.05 | 0.11 | 8.93E-01 |
| NM_001199554 | XRCC6BP1 | 0.05 | 0.13 | 8.93E-01 |
| NM_001305104 | TDRD7 | 0.07 | 0.17 | 8.93E-01 |
| NM_204531 | PCM1 | 0.04 | 0.09 | 8.93E-01 |
| NM_001031377 | FAM76A | 0.05 | 0.13 | 8.93E-01 |
| NM_001001765 | UBA5 | -0.07 | 0.18 | 8.93E-01 |
| NM_001007086 | CAV2 | 0.06 | 0.15 | 8.93E-01 |
| NM_001017414 | SIRT2 | 0.05 | 0.11 | 8.93E-01 |
| NM_001030649 | EIF4A3 | 0.04 | 0.10 | 8.93E-01 |
| NM_001030730 | NAPEPLD | -0.05 | 0.11 | 8.93E-01 |
| NM_001030785 | KIAA1524 | 0.05 | 0.12 | 8.93E-01 |
| NM_001030897 | MEAF6 | 0.06 | 0.13 | 8.93E-01 |
| NM_001031004 | NSUN2 | -0.06 | 0.15 | 8.93E-01 |
| NM_001031119 | CHM | -0.04 | 0.10 | 8.93E-01 |
| NM_001031247 | NDUFA10 | 0.05 | 0.12 | 8.93E-01 |
| NM_001031410 | TXN2 | 0.06 | 0.14 | 8.93E-01 |
| NM_001031534 | ETFDH | 0.04 | 0.09 | 8.93E-01 |
| NM_001039316 | GUSB | 0.05 | 0.13 | 8.93E-01 |
| NM_001097524 | SPRY1 | 0.07 | 0.18 | 8.93E-01 |
| NM_001195562 | PTCHD4 | -0.07 | 0.18 | 8.93E-01 |
| NM_001252280 | SMPDL3B | 0.06 | 0.14 | 8.93E-01 |
| NM_001277634 | C3H6ORF154 | 0.07 | 0.16 | 8.93E-01 |
| NM_001278154 | METTL22 | 0.05 | 0.11 | 8.93E-01 |
| NM_001308613 | LOC776816 | -0.04 | 0.09 | 8.93E-01 |
| NM_204155 | SMYD1 | 0.06 | 0.13 | 8.93E-01 |
| NM_204172 | LMBR1 | -0.07 | 0.18 | 8.93E-01 |
| NM_204642 | CBX4 | 0.05 | 0.13 | 8.93E-01 |
| NM_204826 | MAPKAP1 | -0.06 | 0.14 | 8.93E-01 |
| NM_205120 | ENO1 | 0.04 | 0.09 | 8.93E-01 |
| NM_205386 | SOHO-1 | -0.07 | 0.17 | 8.93E-01 |
| NM_001006219 | YWHAE | -0.03 | 0.08 | 8.94E-01 |
| NM_001199587 | LYVE1 | 0.07 | 0.16 | 8.94E-01 |
| NM_205013 | LEF1 | -0.06 | 0.16 | 8.94E-01 |
| NM_001030683 | GBGT1 | -0.06 | 0.13 | 8.94E-01 |
| NM_204296 | GIT1 | -0.03 | 0.08 | 8.94E-01 |
| NM_001195796 | POR | -0.04 | 0.09 | 8.95E-01 |
| NM_001277398 | SDHA | 0.04 | 0.11 | 8.95E-01 |
| NM_001031452 | NAA35 | -0.04 | 0.09 | 8.95E-01 |
| NM_001278136 | CDCA3 | -0.05 | 0.13 | 8.95E-01 |
| NM_001006215 | LIG3 | 0.04 | 0.10 | 8.95E-01 |
| NM_001006582 | TMEM175 | 0.05 | 0.12 | 8.95E-01 |
| NM_001031355 | MVB12A | -0.04 | 0.10 | 8.95E-01 |
| NM_001080892 | CHMP2A | -0.05 | 0.11 | 8.95E-01 |
| NM_204276 | BRCA2 | -0.06 | 0.16 | 8.95E-01 |
| NM_001006257 | JAM2 | 0.07 | 0.17 | 8.95E-01 |
| NM_001034819 | BMP3 | -0.07 | 0.17 | 8.95E-01 |
| NM_001278090 | CDH1-A | -0.07 | 0.17 | 8.95E-01 |
| NM_204123 | NOG | 0.07 | 0.18 | 8.95E-01 |
| NM_001006462 | IMMT | 0.04 | 0.11 | 8.96E-01 |
| NM_001031409 | EOGT | -0.07 | 0.18 | 8.96E-01 |
| NM_204157 | EEF1A1 | -0.04 | 0.09 | 8.96E-01 |
| NM_001277556 | RWDD3 | -0.07 | 0.18 | 8.96E-01 |
| NM_001030822 | TDRD3 | 0.05 | 0.11 | 8.96E-01 |
| NM_001164395 | EIF3I | 0.05 | 0.12 | 8.96E-01 |
| NM_001048077 | CYP51A1 | -0.05 | 0.13 | 8.96E-01 |
| NM_001008461 | PTP4A1 | -0.04 | 0.10 | 8.96E-01 |
| NM_001008477 | MMADHC | -0.03 | 0.08 | 8.96E-01 |
| NM_001012560 | DEK | -0.03 | 0.08 | 8.96E-01 |
| NM_001012892 | SUCLG1 | -0.04 | 0.10 | 8.96E-01 |
| NM_001031069 | EPM2A | 0.06 | 0.15 | 8.96E-01 |
| NM_001278046 | BMP10 | 0.07 | 0.18 | 8.96E-01 |
| NM_204312 | SAP18 | 0.05 | 0.14 | 8.96E-01 |
| NM_205248 | CSRP1 | 0.04 | 0.10 | 8.96E-01 |
| NM_205503 | GJC1 | -0.04 | 0.11 | 8.96E-01 |
| NM_001006384 | EXOC3 | -0.04 | 0.09 | 8.96E-01 |
| NM_204622 | ST7L | -0.05 | 0.12 | 8.96E-01 |
| NM_001001748 | CYB5A | 0.06 | 0.15 | 8.97E-01 |
| NM_001008680 | RP2 | 0.03 | 0.08 | 8.97E-01 |
| NM_001199519 | MAT1A | 0.06 | 0.15 | 8.97E-01 |
| NM_001030387 | ARIH1 | -0.06 | 0.15 | 8.97E-01 |
| NM_001030586 | TRAIP | -0.07 | 0.16 | 8.97E-01 |
| NM_001277570 | HYI | 0.06 | 0.16 | 8.97E-01 |
| NM_001006211 | SBDS | -0.04 | 0.11 | 8.97E-01 |
| NM_001006419 | LOC421975 | 0.04 | 0.10 | 8.97E-01 |
| NM_001012837 | NECAP2 | -0.05 | 0.13 | 8.97E-01 |
| NM_001030627 | FAM86A | 0.04 | 0.11 | 8.97E-01 |
| NM_001031293 | SERBP1 | 0.04 | 0.11 | 8.97E-01 |
| NM_001199612 | TSPAN13 | -0.06 | 0.14 | 8.97E-01 |
| NM_001199668 | LRRC57 | 0.04 | 0.11 | 8.97E-01 |
| NM_001205100 | TMEM189 | -0.04 | 0.11 | 8.97E-01 |
| NM_001163800 | RAB3GAP2 | -0.04 | 0.11 | 8.98E-01 |
| NM_001199652 | ZFYVE19 | -0.05 | 0.12 | 8.98E-01 |
| NM_001257332 | NCAPH | -0.04 | 0.10 | 8.98E-01 |
| NM_204476 | SGK1 | -0.04 | 0.11 | 8.98E-01 |
| NM_001031153 | UBE2D3 | 0.04 | 0.10 | 8.99E-01 |
| NM_001079757 | SNX6 | 0.04 | 0.10 | 8.99E-01 |
| NM_001302191 | ENHO | 0.05 | 0.14 | 9.00E-01 |
| NM_001031327 | RIT1 | -0.05 | 0.12 | 9.00E-01 |
| NM_204475 | SMAD3 | -0.07 | 0.17 | 9.00E-01 |
| NM_205530 | RFC2 | 0.05 | 0.14 | 9.00E-01 |
| NM_001031471 | SUDS3 | -0.04 | 0.10 | 9.01E-01 |
| NM_001142872 | RP11-894J14.5 | -0.06 | 0.15 | 9.01E-01 |
| NM_001012594 | GORASP2 | 0.03 | 0.08 | 9.02E-01 |
| NM_001031252 | RBM45 | 0.04 | 0.10 | 9.02E-01 |
| NM_001098539 | CTTNBP2 | -0.07 | 0.18 | 9.02E-01 |
| NM_001105064 | NDNL2 | -0.04 | 0.10 | 9.02E-01 |
| NM_001277480 | ZNF639 | 0.04 | 0.10 | 9.02E-01 |
| NM_001031032 | CHAC2 | -0.06 | 0.16 | 9.02E-01 |
| NM_204256 | CDKN1B | -0.04 | 0.12 | 9.02E-01 |
| NM_001006144 | HARS | 0.04 | 0.11 | 9.03E-01 |
| NM_001006454 | CNOT7 | -0.04 | 0.10 | 9.03E-01 |
| NM_001031092 | LAPTM4A | -0.04 | 0.10 | 9.03E-01 |
| NM_001031565 | LOC429115 | -0.07 | 0.17 | 9.03E-01 |
| NM_001039302 | GEMIN2 | 0.06 | 0.14 | 9.03E-01 |
| NM_001302093 | LOC426296 | 0.06 | 0.17 | 9.03E-01 |
| NM_204307 | ACTR3 | -0.04 | 0.11 | 9.03E-01 |
| NM_204592 | CASP8 | -0.06 | 0.15 | 9.03E-01 |
| NM_204135 | SUMO1 | 0.04 | 0.12 | 9.03E-01 |
| NM_001006200 | SH3GLB2 | 0.06 | 0.14 | 9.03E-01 |
| NM_001030596 | IP6K2 | -0.05 | 0.13 | 9.04E-01 |
| NM_001030993 | PDCD6IP | 0.04 | 0.10 | 9.04E-01 |
| NM_001039303 | MTHFD1 | 0.04 | 0.10 | 9.04E-01 |
| NM_001079732 | RP11-371E8.4 | -0.05 | 0.14 | 9.04E-01 |
| NM_204752 | EBF1 | 0.07 | 0.17 | 9.04E-01 |
| NM_001030805 | SH3KBP1 | -0.04 | 0.10 | 9.04E-01 |
| NM_204146 | ITGA3 | -0.04 | 0.12 | 9.04E-01 |
| NM_205441 | VCL | -0.07 | 0.18 | 9.04E-01 |
| NM_001293194 | CD276 | 0.05 | 0.14 | 9.05E-01 |
| NM_001007852 | AC113404.1 | 0.04 | 0.09 | 9.05E-01 |
| NM_001012585 | TMEM229B | -0.06 | 0.15 | 9.05E-01 |
| NM_001030748 | CRADD | 0.05 | 0.15 | 9.05E-01 |
| NM_001031064 | PDCD2 | 0.05 | 0.13 | 9.05E-01 |
| NM_001031503 | PLEKHJ1 | 0.03 | 0.09 | 9.05E-01 |
| NM_001031512 | YME1L1 | -0.03 | 0.08 | 9.05E-01 |
| NM_001039296 | LOC422249 | -0.05 | 0.13 | 9.05E-01 |
| NM_001039491 | RP11-514O12.4 | -0.06 | 0.15 | 9.05E-01 |
| NM_001145430 | SMIM20 | 0.06 | 0.17 | 9.05E-01 |
| NM_001164340 | SMCO4 | -0.05 | 0.13 | 9.05E-01 |
| NM_001199197 | ELOVL5 | -0.04 | 0.11 | 9.05E-01 |
| NM_001199221 | ARIH2 | 0.05 | 0.12 | 9.05E-01 |
| NM_001271892 | TERF2 | 0.04 | 0.11 | 9.05E-01 |
| NM_204198 | TWSG1 | 0.05 | 0.12 | 9.05E-01 |
| NM_204478 | NUF2 | -0.07 | 0.17 | 9.05E-01 |
| NM_204546 | RDM1 | 0.07 | 0.18 | 9.05E-01 |
| NM_204755 | TBPL1 | 0.04 | 0.10 | 9.05E-01 |
| NM_205082 | TOP2B | 0.04 | 0.10 | 9.05E-01 |
| NM_205433 | FGF2 | 0.06 | 0.17 | 9.05E-01 |
| NM_001001776 | GSTA | 0.06 | 0.16 | 9.05E-01 |
| NM_001030555 | FAM96A | -0.05 | 0.13 | 9.05E-01 |
| NM_001277363 | GAS2L3 | -0.06 | 0.17 | 9.05E-01 |
| NM_001277404 | PCMT1L | 0.06 | 0.17 | 9.05E-01 |
| NM_001277622 | CLDN2 | -0.06 | 0.16 | 9.05E-01 |
| NM_204222 | FZD2 | -0.04 | 0.11 | 9.05E-01 |
| NM_205032 | IGF1R | -0.07 | 0.18 | 9.05E-01 |
| NM_001079494 | AP2M1 | 0.03 | 0.08 | 9.06E-01 |
| NM_001277628 | CLN6 | -0.04 | 0.10 | 9.06E-01 |
| NM_001012859 | VPS41 | 0.03 | 0.09 | 9.07E-01 |
| NM_001277377 | MPST | 0.04 | 0.11 | 9.07E-01 |
| NM_001006540 | RNF11 | -0.03 | 0.08 | 9.08E-01 |
| NM_001305146 | RAD54L | -0.04 | 0.11 | 9.08E-01 |
| NM_204560 | ACVR1 | 0.03 | 0.09 | 9.08E-01 |
| NM_001012590 | KLHDC2 | 0.04 | 0.11 | 9.08E-01 |
| NM_001006212 | WBSCR16 | -0.04 | 0.11 | 9.08E-01 |
| NM_205496 | GLG1 | -0.05 | 0.14 | 9.08E-01 |
| NM_001007970 | ARL2BP | -0.04 | 0.12 | 9.08E-01 |
| NM_001008674 | DYX1C1 | -0.06 | 0.16 | 9.08E-01 |
| NM_001030881 | ATP13A2 | -0.06 | 0.18 | 9.08E-01 |
| NM_001277841 | SEC13 | 0.03 | 0.09 | 9.08E-01 |
| NM_001277865 | C11orf31 | 0.06 | 0.16 | 9.08E-01 |
| NM_001278149 | CBY1 | 0.06 | 0.16 | 9.08E-01 |
| NM_204505 | RGS9BP | -0.04 | 0.11 | 9.08E-01 |
| NM_001005791 | AVEN | 0.05 | 0.13 | 9.08E-01 |
| NM_001006318 | RBM7 | 0.05 | 0.14 | 9.08E-01 |
| NM_001270557 | SIGMAR1 | 0.05 | 0.13 | 9.09E-01 |
| NM_001030680 | STRBP | -0.03 | 0.09 | 9.09E-01 |
| NM_001012578 | ACSL1 | -0.04 | 0.10 | 9.09E-01 |
| NM_001001300 | TOP1MT | 0.04 | 0.11 | 9.11E-01 |
| NM_001199539 | PUS7 | 0.04 | 0.12 | 9.11E-01 |
| NM_001277087 | RTN4 | 0.04 | 0.12 | 9.11E-01 |
| NM_204863 | PLEK | -0.06 | 0.18 | 9.11E-01 |
| NM_001012929 | RASA2 | 0.05 | 0.14 | 9.11E-01 |
| NM_001006408 | SNX3 | -0.03 | 0.09 | 9.12E-01 |
| NM_001006549 | LSG1 | 0.04 | 0.12 | 9.12E-01 |
| NM_001030716 | DPH1 | -0.05 | 0.15 | 9.12E-01 |
| NM_001006537 | IPO13 | 0.04 | 0.10 | 9.13E-01 |
| NM_204741 | VDAC2 | 0.03 | 0.08 | 9.14E-01 |
| NM_001012778 | LSM14A | -0.04 | 0.13 | 9.14E-01 |
| NM_001031333 | AK6 | 0.04 | 0.11 | 9.14E-01 |
| NM_001006159 | GET4 | 0.03 | 0.09 | 9.14E-01 |
| NM_001006184 | AACS | -0.03 | 0.08 | 9.14E-01 |
| NM_001199598 | TMEM26 | 0.03 | 0.10 | 9.14E-01 |
| NM_001285869 | TICRR | -0.03 | 0.09 | 9.14E-01 |
| NM_001031588 | PGLS | 0.05 | 0.14 | 9.16E-01 |
| NM_001006142 | PPP4R2 | -0.03 | 0.10 | 9.16E-01 |
| NM_001031249 | STK17B | -0.04 | 0.11 | 9.16E-01 |
| NM_001293283 | SLC30A4 | -0.03 | 0.09 | 9.16E-01 |
| NM_001039254 | ITGB1 | 0.03 | 0.08 | 9.16E-01 |
| NM_001006225 | PSMC2 | 0.04 | 0.11 | 9.17E-01 |
| NM_001142862 | OAZ2 | 0.04 | 0.11 | 9.17E-01 |
| NM_001277811 | PDGFD | -0.05 | 0.13 | 9.17E-01 |
| NM_204480 | ST3GAL2 | -0.04 | 0.12 | 9.17E-01 |
| NM_001006486 | SETD3 | 0.04 | 0.12 | 9.18E-01 |
| NM_001030780 | RABL3 | 0.03 | 0.10 | 9.18E-01 |
| NM_001030792 | CHMP2B | 0.03 | 0.10 | 9.18E-01 |
| NM_001031099 | CENPO | 0.06 | 0.17 | 9.18E-01 |
| NM_001031547 | APIP | -0.04 | 0.11 | 9.18E-01 |
| NM_001099353 | BMA1 | -0.05 | 0.15 | 9.18E-01 |
| NM_001199412 | SLX4IP | 0.05 | 0.15 | 9.18E-01 |
| NM_001006209 | KPNA2 | 0.04 | 0.12 | 9.18E-01 |
| NM_001199604 | SLC16A7 | -0.04 | 0.12 | 9.18E-01 |
| NM_001006221 | RPA1 | 0.04 | 0.11 | 9.18E-01 |
| NM_001006400 | MCFD2 | 0.03 | 0.08 | 9.18E-01 |
| NM_001007833 | NUBP2 | -0.04 | 0.11 | 9.18E-01 |
| NM_001012873 | PPP1R21 | -0.03 | 0.10 | 9.18E-01 |
| NM_001012921 | GTDC1 | -0.03 | 0.10 | 9.18E-01 |
| NM_001030827 | UFM1 | 0.04 | 0.12 | 9.18E-01 |
| NM_001030971 | PIP4K2A | 0.04 | 0.11 | 9.18E-01 |
| NM_001031240 | ACADSB | -0.05 | 0.15 | 9.18E-01 |
| NM_001031505 | PTDSS1 | -0.03 | 0.10 | 9.18E-01 |
| NM_001039602 | SLC27A1 | 0.06 | 0.18 | 9.18E-01 |
| NM_001079717 | PEPD | -0.03 | 0.10 | 9.18E-01 |
| NM_001161801 | AHCTF1 | 0.03 | 0.10 | 9.18E-01 |
| NM_001166327 | SEPW1 | 0.05 | 0.15 | 9.18E-01 |
| NM_001199108 | SOCS4 | 0.04 | 0.13 | 9.18E-01 |
| NM_001287175 | APLF | -0.04 | 0.13 | 9.18E-01 |
| NM_204314 | PTPRG | 0.05 | 0.14 | 9.18E-01 |
| NM_204548 | HOXA3 | 0.03 | 0.09 | 9.18E-01 |
| NM_204828 | RLIM | -0.04 | 0.13 | 9.18E-01 |
| NM_204867 | TMCO3 | -0.04 | 0.12 | 9.18E-01 |
| NM_204987 | RPLP0 | -0.05 | 0.15 | 9.18E-01 |
| NM_205036 | TGFBI | 0.05 | 0.15 | 9.18E-01 |
| NM_204414 | YBX1 | 0.03 | 0.10 | 9.18E-01 |
| NM_204420 | MMP2 | 0.04 | 0.11 | 9.19E-01 |
| NM_001001464 | PPARA | -0.06 | 0.18 | 9.19E-01 |
| NM_001004406 | CFL2 | 0.04 | 0.12 | 9.19E-01 |
| NM_001005836 | AARS | -0.02 | 0.07 | 9.19E-01 |
| NM_001006295 | STX16 | 0.03 | 0.08 | 9.19E-01 |
| NM_001006406 | EIF3M | -0.03 | 0.10 | 9.19E-01 |
| NM_001006430 | LOC422214 | 0.04 | 0.13 | 9.19E-01 |
| NM_001006450 | ERI1 | -0.04 | 0.12 | 9.19E-01 |
| NM_001012291 | PNRC1 | -0.05 | 0.14 | 9.19E-01 |
| NM_001030632 | ARPC1B | -0.04 | 0.11 | 9.19E-01 |
| NM_001030686 | GPR107 | -0.03 | 0.10 | 9.19E-01 |
| NM_001030776 | MLF2 | 0.04 | 0.13 | 9.19E-01 |
| NM_001030939 | USE1 | 0.04 | 0.11 | 9.19E-01 |
| NM_001079749 | EVC2 | -0.06 | 0.18 | 9.19E-01 |
| NM_001277704 | PERP2 | 0.05 | 0.15 | 9.19E-01 |
| NM_001305152 | TMEM173 | 0.05 | 0.17 | 9.19E-01 |
| NM_204400 | TRAM1 | -0.03 | 0.10 | 9.19E-01 |
| NM_204458 | RFTN1 | -0.05 | 0.14 | 9.19E-01 |
| NM_204714 | FGF18 | 0.06 | 0.18 | 9.19E-01 |
| NM_204869 | RBPMS2 | -0.04 | 0.12 | 9.19E-01 |
| NM_205139 | SMARCA2 | -0.04 | 0.11 | 9.19E-01 |
| NM_001001463 | ARNTL | 0.03 | 0.10 | 9.20E-01 |
| NM_001004410 | PIK3CA | -0.05 | 0.17 | 9.20E-01 |
| NM_001006291 | SERINC3 | 0.02 | 0.08 | 9.20E-01 |
| NM_001006448 | POLR2B | 0.03 | 0.10 | 9.20E-01 |
| NM_001007840 | RNF185 | -0.03 | 0.10 | 9.20E-01 |
| NM_001007982 | SKP2 | 0.03 | 0.09 | 9.20E-01 |
| NM_001008451 | TRAPPC3 | -0.04 | 0.12 | 9.20E-01 |
| NM_001012596 | FAM129A | 0.06 | 0.18 | 9.20E-01 |
| NM_001030551 | MESDC2 | 0.03 | 0.10 | 9.20E-01 |
| NM_001030807 | CTPS2 | -0.05 | 0.15 | 9.20E-01 |
| NM_001039453 | RP5-877J2.1 | -0.06 | 0.18 | 9.20E-01 |
| NM_001079750 | WDFY2 | 0.05 | 0.15 | 9.20E-01 |
| NM_001177555 | TRPM7 | -0.04 | 0.13 | 9.20E-01 |
| NM_001198640 | DCTN5 | -0.04 | 0.13 | 9.20E-01 |
| NM_001199690 | SIPA1L1 | -0.04 | 0.14 | 9.20E-01 |
| NM_001277554 | JKAMP | 0.03 | 0.10 | 9.20E-01 |
| NM_001277616 | PARS2 | -0.05 | 0.14 | 9.20E-01 |
| NM_204308 | APP | 0.03 | 0.10 | 9.20E-01 |
| NM_204666 | CLEC3B | 0.05 | 0.16 | 9.20E-01 |
| NM_204703 | TLL1 | -0.06 | 0.17 | 9.20E-01 |
| NM_205398 | TPT1 | 0.03 | 0.11 | 9.20E-01 |
| NM_001001760 | CDH13 | -0.05 | 0.15 | 9.20E-01 |
| NM_001005840 | ITFG1 | 0.05 | 0.15 | 9.20E-01 |
| NM_001006544 | C9H21ORF2 | 0.04 | 0.13 | 9.20E-01 |
| NM_001031423 | ABHD17B | -0.04 | 0.13 | 9.20E-01 |
| NM_001031612 | RMI1 | 0.04 | 0.12 | 9.20E-01 |
| NM_001277546 | WDR89 | -0.04 | 0.12 | 9.20E-01 |
| NM_204322 | GLDC | 0.03 | 0.11 | 9.21E-01 |
| NM_001030990 | CDV3 | 0.04 | 0.12 | 9.21E-01 |
| NM_001031024 | SDCBP | 0.03 | 0.10 | 9.22E-01 |
| NM_001006297 | RTFDC1 | 0.04 | 0.13 | 9.22E-01 |
| NM_001006433 | CSTF2 | 0.02 | 0.08 | 9.22E-01 |
| NM_001031267 | RNASEL | 0.05 | 0.16 | 9.22E-01 |
| NM_001277424 | UEVLD | -0.04 | 0.12 | 9.22E-01 |
| NM_204348 | XIRP1 | 0.05 | 0.18 | 9.22E-01 |
| NM_204864 | MEF2A | -0.06 | 0.18 | 9.22E-01 |
| NM_204967 | FMOD | -0.05 | 0.17 | 9.22E-01 |
| NM_001080879 | FXYD6 | 0.04 | 0.12 | 9.22E-01 |
| NM_001278112 | RABL2B | 0.05 | 0.15 | 9.22E-01 |
| NM_001007953 | GCLM | 0.04 | 0.13 | 9.22E-01 |
| NM_001030710 | ORAI2 | -0.04 | 0.13 | 9.22E-01 |
| NM_001278099 | DUSP12 | -0.04 | 0.12 | 9.22E-01 |
| NM_001163651 | PAQR7 | 0.05 | 0.17 | 9.23E-01 |
| NM_001256499 | LOC420860 | -0.04 | 0.13 | 9.23E-01 |
| NM_001277385 | LRRC20 | -0.05 | 0.16 | 9.23E-01 |
| NM_204554 | WFDC1 | -0.04 | 0.13 | 9.23E-01 |
| NM_001006561 | SSR2 | 0.04 | 0.14 | 9.23E-01 |
| NM_001012943 | STX7 | -0.03 | 0.10 | 9.23E-01 |
| NM_001198620 | PDHB | -0.04 | 0.12 | 9.23E-01 |
| NM_001007877 | TMEM230 | 0.03 | 0.10 | 9.24E-01 |
| NM_001031210 | YY1 | -0.04 | 0.12 | 9.24E-01 |
| NM_001031275 | HCCS | 0.03 | 0.11 | 9.24E-01 |
| NM_001079728 | RBMX | 0.03 | 0.10 | 9.24E-01 |
| NM_206959 | HSP90AB1 | -0.03 | 0.11 | 9.24E-01 |
| NM_001005813 | TMOD3 | -0.05 | 0.18 | 9.24E-01 |
| NM_001031109 | MRPS7 | -0.03 | 0.10 | 9.24E-01 |
| NM_001293310 | LOC408180 | 0.05 | 0.15 | 9.24E-01 |
| NM_001197243 | LOC422426 | 0.04 | 0.13 | 9.24E-01 |
| NM_001012821 | ZFP92 | 0.03 | 0.11 | 9.24E-01 |
| NM_205318 | MYBL2 | 0.02 | 0.08 | 9.24E-01 |
| NM_001031288 | DHCR24 | -0.05 | 0.17 | 9.25E-01 |
| NM_001277150 | WWC1 | 0.03 | 0.11 | 9.25E-01 |
| NM_001199320 | EXOC4 | -0.03 | 0.11 | 9.25E-01 |
| NM_001005798 | SNTB1 | 0.03 | 0.11 | 9.25E-01 |
| NM_001030655 | EP400 | 0.04 | 0.13 | 9.25E-01 |
| NM_001278001 | ARMC7 | 0.04 | 0.13 | 9.25E-01 |
| NM_001034820 | PROCR | -0.05 | 0.15 | 9.26E-01 |
| NM_001006377 | SERPINB6 | 0.05 | 0.16 | 9.26E-01 |
| NM_001048266 | FAM118B | -0.05 | 0.15 | 9.26E-01 |
| NM_001044649 | CENPQ | 0.04 | 0.14 | 9.26E-01 |
| NM_001198603 | SPG21 | 0.05 | 0.16 | 9.26E-01 |
| NM_001012807 | TADA2A | -0.04 | 0.14 | 9.27E-01 |
| NM_001006453 | ASAH1 | 0.04 | 0.12 | 9.27E-01 |
| NM_001030601 | IMPDH2 | 0.03 | 0.11 | 9.27E-01 |
| NM_001030654 | Sep-05 | -0.04 | 0.12 | 9.27E-01 |
| NM_001030773 | MKLN1 | 0.03 | 0.10 | 9.27E-01 |
| NM_001030875 | LOC419429 | -0.05 | 0.17 | 9.27E-01 |
| NM_001030974 | RBM48 | -0.03 | 0.11 | 9.27E-01 |
| NM_001031344 | C10orf2 | -0.04 | 0.13 | 9.27E-01 |
| NM_001031417 | PRRC1 | -0.03 | 0.12 | 9.27E-01 |
| NM_001031434 | RNF20 | 0.03 | 0.12 | 9.27E-01 |
| NM_001031499 | YOD1 | -0.04 | 0.14 | 9.27E-01 |
| NM_001031513 | STT3B | -0.04 | 0.15 | 9.27E-01 |
| NM_001039283 | MPPE1 | -0.04 | 0.14 | 9.27E-01 |
| NM_001039331 | PRPF19 | 0.03 | 0.10 | 9.27E-01 |
| NM_001168005 | ACYP1 | 0.05 | 0.17 | 9.27E-01 |
| NM_001197288 | ACAA1 | 0.04 | 0.12 | 9.27E-01 |
| NM_001277415 | TSG101 | 0.04 | 0.13 | 9.27E-01 |
| NM_001277913 | SLC18B1 | -0.04 | 0.14 | 9.27E-01 |
| NM_204656 | DBT | 0.03 | 0.12 | 9.27E-01 |
| NM_205391 | NREP | 0.03 | 0.11 | 9.27E-01 |
| NM_206863 | VAV3 | 0.04 | 0.15 | 9.27E-01 |
| NM_001277796 | TAF9 | 0.04 | 0.13 | 9.27E-01 |
| NM_205104 | BLMH | 0.03 | 0.10 | 9.27E-01 |
| NM_001006426 | NUP85 | 0.03 | 0.11 | 9.28E-01 |
| NM_001030882 | GFPT1 | 0.03 | 0.11 | 9.28E-01 |
| NM_001142256 | CNN2 | 0.04 | 0.15 | 9.28E-01 |
| NM_001267535 | TIAM2 | 0.03 | 0.10 | 9.28E-01 |
| NM_204969 | PLA2G7 | 0.05 | 0.18 | 9.28E-01 |
| NM_204570 | CBFB | -0.03 | 0.11 | 9.29E-01 |
| NM_001006478 | SRP14 | 0.05 | 0.16 | 9.29E-01 |
| NM_001277592 | NSUN4 | -0.04 | 0.13 | 9.30E-01 |
| NM_001030685 | POMT1 | 0.05 | 0.18 | 9.30E-01 |
| NM_001079492 | FAM26E | 0.05 | 0.18 | 9.30E-01 |
| NM_001005796 | SSRP1 | -0.03 | 0.12 | 9.31E-01 |
| NM_001012901 | DLST | -0.03 | 0.11 | 9.31E-01 |
| NM_001030848 | EIF6 | 0.02 | 0.08 | 9.31E-01 |
| NM_001278017 | SET domain containing 5 | -0.04 | 0.13 | 9.31E-01 |
| NM_204954 | ENTPD2 | -0.03 | 0.12 | 9.31E-01 |
| NR_035219 | MIR1723 | 0.05 | 0.16 | 9.31E-01 |
| NM_001006130 | USP10 | -0.04 | 0.13 | 9.31E-01 |
| NM_001006480 | ZNF410 | 0.03 | 0.10 | 9.31E-01 |
| NM_001199488 | SNX16 | -0.04 | 0.13 | 9.31E-01 |
| NM_001282381 | Mar-02 | 0.03 | 0.11 | 9.31E-01 |
| NM_001012846 | ACSBG2 | 0.03 | 0.11 | 9.32E-01 |
| NM_001167728 | NAGA | -0.05 | 0.17 | 9.32E-01 |
| NM_001271144 | NAT8B | 0.05 | 0.17 | 9.32E-01 |
| NM_001006243 | MCM5 | -0.03 | 0.10 | 9.32E-01 |
| NM_204791 | TOP2A | -0.03 | 0.12 | 9.32E-01 |
| NM_204375 | SLC2A8 | 0.02 | 0.09 | 9.33E-01 |
| NM_001006562 | ABCF2 | -0.03 | 0.09 | 9.33E-01 |
| NM_001080871 | ARHGDIA | -0.02 | 0.08 | 9.33E-01 |
| NM_001030975 | FAM133B | -0.03 | 0.10 | 9.33E-01 |
| NM_001006186 | TMED2 | 0.02 | 0.08 | 9.34E-01 |
| NM_001199559 | GNS | 0.03 | 0.10 | 9.34E-01 |
| NM_001030678 | PBX3 | -0.03 | 0.11 | 9.35E-01 |
| NM_001031179 | FAR1 | 0.03 | 0.10 | 9.35E-01 |
| NM_001277420 | TMEM55A | 0.03 | 0.11 | 9.35E-01 |
| NM_001006309 | HNRNPR | 0.03 | 0.10 | 9.35E-01 |
| NM_001006424 | SLC9A3R1 | 0.04 | 0.16 | 9.35E-01 |
| NM_001199845 | C18H17ORF106 | -0.04 | 0.15 | 9.35E-01 |
| NM_204496 | HNRNPH1 | 0.02 | 0.08 | 9.35E-01 |
| NM_205024 | RBMS1 | -0.05 | 0.18 | 9.36E-01 |
| NM_001006187 | ANAPC5 | 0.03 | 0.11 | 9.36E-01 |
| NM_001006189 | RP11-87C12.2 | -0.03 | 0.12 | 9.36E-01 |
| NM_001006304 | PQLC2 | -0.04 | 0.13 | 9.36E-01 |
| NM_001006415 | YWHAQ | -0.04 | 0.14 | 9.36E-01 |
| NM_001006567 | OAT | 0.02 | 0.09 | 9.36E-01 |
| NM_001007823 | RPSA | -0.03 | 0.12 | 9.36E-01 |
| NM_001008681 | ABHD13 | -0.03 | 0.10 | 9.36E-01 |
| NM_001012882 | NCOA1 | -0.05 | 0.17 | 9.36E-01 |
| NM_001012917 | WIPF1 | 0.04 | 0.16 | 9.36E-01 |
| NM_001031600 | MEF2D | 0.03 | 0.13 | 9.36E-01 |
| NM_001031607 | N4BP3 | -0.02 | 0.09 | 9.36E-01 |
| NM_001033643 | CR1L | 0.04 | 0.15 | 9.36E-01 |
| NM_001077232 | CX3CL1 | -0.05 | 0.18 | 9.36E-01 |
| NM_001079498 | SUMO3 | 0.04 | 0.14 | 9.36E-01 |
| NM_001282395 | TTC30A | -0.04 | 0.13 | 9.36E-01 |
| NM_001293656 | ADC | -0.04 | 0.15 | 9.36E-01 |
| NM_204118 | AHR | -0.05 | 0.17 | 9.36E-01 |
| NM_204543 | MTFR1 | -0.02 | 0.08 | 9.36E-01 |
| NM_204654 | CRISPLD1 | -0.05 | 0.17 | 9.36E-01 |
| NM_204671 | SNCB | 0.04 | 0.16 | 9.36E-01 |
| NM_204917 | ETV1 | 0.04 | 0.15 | 9.36E-01 |
| NM_205263 | PARP1 | 0.03 | 0.11 | 9.36E-01 |
| NM_205321 | GOT1 | 0.03 | 0.12 | 9.36E-01 |
| NM_205429 | 17.5 | -0.04 | 0.17 | 9.36E-01 |
| NM_001277164 | RPS2 | 0.04 | 0.14 | 9.37E-01 |
| NM_205244 | CCNA2 | 0.02 | 0.09 | 9.37E-01 |
| NM_001039268 | RBM19 | -0.02 | 0.10 | 9.38E-01 |
| NM_001305065 | TLCD1 | 0.04 | 0.15 | 9.38E-01 |
| NM_001012290 | RP1-27O5.3 | -0.04 | 0.15 | 9.38E-01 |
| NM_001030860 | MAPRE1 | 0.03 | 0.10 | 9.38E-01 |
| NM_001031124 | UPRT | 0.04 | 0.14 | 9.38E-01 |
| NM_001270725 | ATAD5 | 0.03 | 0.13 | 9.38E-01 |
| NM_001277595 | C11H19ORF40 | -0.03 | 0.11 | 9.38E-01 |
| NM_001282296 | KTI12 | -0.03 | 0.11 | 9.38E-01 |
| NM_205379 | TGIF1 | 0.04 | 0.14 | 9.38E-01 |
| NR_105454 | MIR6585 | 0.05 | 0.18 | 9.38E-01 |
| NM_001006390 | LYN | -0.03 | 0.12 | 9.38E-01 |
| NM_001012802 | EXOC7 | 0.03 | 0.10 | 9.38E-01 |
| NM_001006403 | TSNAX | -0.03 | 0.11 | 9.38E-01 |
| NM_001030750 | NEDD1 | 0.02 | 0.09 | 9.38E-01 |
| NM_001030977 | ASNS | 0.03 | 0.12 | 9.38E-01 |
| NM_001114630 | RP11-552F3.12 | -0.04 | 0.14 | 9.38E-01 |
| NM_001252309 | IFT81 | 0.04 | 0.14 | 9.38E-01 |
| NM_204284 | NRP2 | -0.04 | 0.16 | 9.38E-01 |
| NM_205134 | NFKB1 | 0.03 | 0.12 | 9.38E-01 |
| NM_205329 | SRL | 0.04 | 0.15 | 9.38E-01 |
| NM_001199384 | MDM2 | -0.03 | 0.11 | 9.39E-01 |
| NM_001007831 | PHKB | -0.04 | 0.15 | 9.39E-01 |
| NM_001277275 | MED27 | 0.04 | 0.17 | 9.39E-01 |
| NM_001293093 | FLT4 | -0.03 | 0.12 | 9.39E-01 |
| NM_204188 | ACO2 | -0.03 | 0.10 | 9.39E-01 |
| NM_204806 | HAS2 | 0.04 | 0.18 | 9.39E-01 |
| NM_205286 | LMNB1 | -0.04 | 0.15 | 9.39E-01 |
| NM_001004766 | FUT8 | 0.04 | 0.17 | 9.39E-01 |
| NM_001030603 | RAD18 | 0.03 | 0.14 | 9.39E-01 |
| NM_001030793 | HSPA13 | -0.04 | 0.15 | 9.39E-01 |
| NM_001030928 | KIF18B | 0.03 | 0.13 | 9.39E-01 |
| NM_001030967 | UBE3C | -0.04 | 0.16 | 9.39E-01 |
| NM_001277835 | STAG3 | -0.03 | 0.12 | 9.39E-01 |
| NM_204173 | TBX5 | 0.03 | 0.13 | 9.39E-01 |
| NM_204246 | TGFBR1 | -0.03 | 0.11 | 9.39E-01 |
| NM_001031067 | LIN7B | -0.03 | 0.11 | 9.41E-01 |
| NM_001293095 | ETV5 | 0.04 | 0.18 | 9.41E-01 |
| NM_001031158 | SEPSECS | -0.03 | 0.13 | 9.42E-01 |
| NM_001031474 | UBAC1 | 0.02 | 0.09 | 9.42E-01 |
| NM_001197036 | TADA1L | 0.04 | 0.16 | 9.42E-01 |
| NM_001199806 | LAMA1 | -0.03 | 0.13 | 9.42E-01 |
| NM_204731 | SOX8 | 0.04 | 0.18 | 9.42E-01 |
| NM_205309 | BZFP1 | 0.03 | 0.11 | 9.42E-01 |
| NM_001006289 | YWHAB | -0.02 | 0.10 | 9.43E-01 |
| NM_001031538 | ANXA5 | 0.03 | 0.13 | 9.43E-01 |
| NM_001034827 | PRKAG1 | -0.03 | 0.13 | 9.43E-01 |
| NM_205479 | MYL4 | -0.03 | 0.10 | 9.43E-01 |
| NR_035279 | MIR1778 | 0.04 | 0.16 | 9.43E-01 |
| NM_001081705 | ARF6 | -0.03 | 0.11 | 9.43E-01 |
| NM_204303 | ME1 | 0.03 | 0.13 | 9.43E-01 |
| NM_205411 | HMGCS1 | -0.04 | 0.16 | 9.43E-01 |
| NM_001001785 | XYLT2 | -0.04 | 0.16 | 9.44E-01 |
| NM_001005834 | CIAPIN1 | -0.03 | 0.11 | 9.44E-01 |
| NM_001030764 | CHPT1 | 0.02 | 0.10 | 9.45E-01 |
| NM_001099352 | TRIM7.1 | -0.04 | 0.17 | 9.45E-01 |
| NM_001199658 | OIP5 | -0.04 | 0.18 | 9.45E-01 |
| NM_001008683 | DCLRE1A | -0.03 | 0.12 | 9.45E-01 |
| NM_001012796 | RAB14 | -0.02 | 0.09 | 9.45E-01 |
| NM_001007081 | ESRRG | -0.04 | 0.18 | 9.45E-01 |
| NM_001031318 | DCUN1D1 | -0.03 | 0.13 | 9.45E-01 |
| NM_001302141 | BLCAP | -0.03 | 0.11 | 9.45E-01 |
| NM_205161 | ZBTB14 | -0.04 | 0.15 | 9.45E-01 |
| NM_205480 | MARCKS | -0.03 | 0.13 | 9.45E-01 |
| NM_001277533 | MGST3 | 0.04 | 0.16 | 9.46E-01 |
| NM_001137649 | DEAF1 | -0.03 | 0.15 | 9.47E-01 |
| NM_001006150 | G3BP1 | 0.02 | 0.09 | 9.47E-01 |
| NM_001030766 | FAM60A | 0.03 | 0.12 | 9.47E-01 |
| NM_001031381 | MANSC1 | 0.04 | 0.16 | 9.47E-01 |
| NM_204156 | HDAC1 | 0.03 | 0.12 | 9.47E-01 |
| NM_001006520 | BCS1L | -0.03 | 0.13 | 9.47E-01 |
| NM_001031324 | SARNP | -0.02 | 0.10 | 9.47E-01 |
| NM_001174059 | ITPR1 | 0.03 | 0.12 | 9.47E-01 |
| NM_001006569 | CENPL | -0.03 | 0.12 | 9.47E-01 |
| NM_001031346 | SNX27 | -0.02 | 0.08 | 9.47E-01 |
| NM_204309 | SOX18 | -0.03 | 0.15 | 9.47E-01 |
| NM_001277373 | SERHL2 | 0.03 | 0.13 | 9.48E-01 |
| NM_001031217 | GHITM | -0.03 | 0.12 | 9.49E-01 |
| NM_001199547 | ZNF365 | 0.04 | 0.16 | 9.49E-01 |
| NM_001302158 | CTNNBIP1 | -0.04 | 0.16 | 9.49E-01 |
| NM_204456 | FGFRL1 | -0.03 | 0.13 | 9.49E-01 |
| NM_001001129 | CYP26A1 | 0.04 | 0.17 | 9.49E-01 |
| NM_001030738 | IRAK4 | 0.04 | 0.18 | 9.50E-01 |
| NM_204763 | UBE2J1 | 0.04 | 0.16 | 9.50E-01 |
| NM_001031336 | LAP3 | 0.03 | 0.15 | 9.50E-01 |
| NM_204565 | NSG1 | 0.04 | 0.17 | 9.50E-01 |
| NM_204853 | XPA | 0.03 | 0.14 | 9.50E-01 |
| NM_001030595 | ARHGEF3 | 0.04 | 0.18 | 9.51E-01 |
| NM_001031120 | HDX | 0.03 | 0.15 | 9.51E-01 |
| NM_001039262 | FBXW11 | -0.02 | 0.09 | 9.51E-01 |
| NM_001162399 | COL4A1 | 0.04 | 0.16 | 9.51E-01 |
| NM_204629 | AKR1B10 | 0.04 | 0.16 | 9.51E-01 |
| NM_205481 | LOX | 0.04 | 0.18 | 9.51E-01 |
| NM_001031224 | DNAJB12 | -0.02 | 0.11 | 9.51E-01 |
| NM_001031048 | DTL | -0.03 | 0.13 | 9.51E-01 |
| NM_001031193 | NUSAP1 | 0.02 | 0.09 | 9.51E-01 |
| NM_001199910 | PDP1 | 0.03 | 0.13 | 9.51E-01 |
| NM_001282348 | MAP2K5 | 0.03 | 0.14 | 9.51E-01 |
| NM_001290134 | XPO1 | 0.03 | 0.12 | 9.51E-01 |
| NM_001006230 | THAP5 | 0.02 | 0.10 | 9.51E-01 |
| NM_001008466 | RAB33B | -0.02 | 0.08 | 9.51E-01 |
| NM_001012524 | CLK3 | -0.03 | 0.12 | 9.51E-01 |
| NM_001110059 | THADA | 0.04 | 0.16 | 9.51E-01 |
| NM_001199546 | JMJD1C | -0.03 | 0.13 | 9.51E-01 |
| NM_001199681 | DNAL1 | -0.03 | 0.13 | 9.51E-01 |
| NM_205080 | CAPN2 | -0.04 | 0.18 | 9.51E-01 |
| NM_204345 | CHEK1 | -0.03 | 0.15 | 9.52E-01 |
| NM_001006234 | LTA4H | -0.02 | 0.09 | 9.52E-01 |
| NM_001039282 | GRB10 | 0.04 | 0.18 | 9.52E-01 |
| NM_001113761 | B3GNT5 | 0.02 | 0.12 | 9.52E-01 |
| NM_001044665 | COLEC11 | 0.04 | 0.18 | 9.52E-01 |
| NR_031456 | MIRLET7D | -0.03 | 0.15 | 9.52E-01 |
| NM_001006247 | STRAP | -0.02 | 0.09 | 9.52E-01 |
| NM_204204 | UGT8 | -0.04 | 0.18 | 9.52E-01 |
| NM_001030775 | COPS7A | -0.02 | 0.10 | 9.52E-01 |
| NM_001199402 | DTD1 | 0.02 | 0.11 | 9.52E-01 |
| NM_001006198 | WDR5 | 0.03 | 0.13 | 9.52E-01 |
| NM_001031419 | SLC30A5 | 0.02 | 0.11 | 9.52E-01 |
| NM_001031458 | NUDT19 | -0.03 | 0.13 | 9.52E-01 |
| NM_001195570 | SCAF11 | -0.02 | 0.09 | 9.52E-01 |
| NM_001199100 | FANCB | 0.04 | 0.18 | 9.52E-01 |
| NM_001277097 | ANK3 | -0.02 | 0.12 | 9.52E-01 |
| NM_001277144 | BHLHE40 | -0.04 | 0.17 | 9.52E-01 |
| NM_001277474 | FBLN5 | 0.04 | 0.17 | 9.52E-01 |
| NM_001277517 | GLTP | 0.03 | 0.15 | 9.52E-01 |
| NM_001277606 | RRAD | -0.03 | 0.16 | 9.52E-01 |
| NM_001006233 | METAP2 | -0.02 | 0.09 | 9.52E-01 |
| NM_001006449 | SRP72 | 0.02 | 0.09 | 9.52E-01 |
| NM_001012798 | EXOSC2 | 0.03 | 0.14 | 9.52E-01 |
| NM_001031049 | LPGAT1 | 0.04 | 0.17 | 9.52E-01 |
| NM_001031051 | DIEXF | -0.02 | 0.12 | 9.52E-01 |
| NM_001031311 | PIK3CB | -0.03 | 0.13 | 9.52E-01 |
| NM_001127314 | SOCS5 | -0.03 | 0.15 | 9.52E-01 |
| NM_001199452 | RGS6 | -0.03 | 0.17 | 9.52E-01 |
| NM_001206510 | TCF7L2 | -0.03 | 0.13 | 9.52E-01 |
| NM_001277156 | RRN3 | 0.02 | 0.11 | 9.52E-01 |
| NM_001277277 | C1H11ORF54 | -0.03 | 0.15 | 9.52E-01 |
| NM_001302197 | STRA13 | 0.03 | 0.16 | 9.52E-01 |
| NM_204402 | RIPK1 | 0.02 | 0.10 | 9.52E-01 |
| NM_204494 | DPYSL2 | 0.03 | 0.12 | 9.52E-01 |
| NM_204900 | CLU | 0.02 | 0.10 | 9.52E-01 |
| NM_001031358 | CCNE1 | -0.03 | 0.14 | 9.53E-01 |
| NM_204618 | NFIL3 | -0.04 | 0.18 | 9.53E-01 |
| NM_001289888 | CSNK1D | -0.02 | 0.07 | 9.53E-01 |
| NM_205437 | CAPZB | -0.03 | 0.14 | 9.53E-01 |
| NM_204646 | BCHE | 0.04 | 0.18 | 9.54E-01 |
| NM_001006232 | POC1B | -0.02 | 0.10 | 9.54E-01 |
| NM_001006565 | ANKRA2 | 0.02 | 0.10 | 9.54E-01 |
| NM_001277448 | TSTA3 | -0.03 | 0.13 | 9.54E-01 |
| NM_001030572 | PDCD2L | -0.02 | 0.12 | 9.54E-01 |
| NM_213575 | MID1IP1 | -0.03 | 0.17 | 9.54E-01 |
| NM_001030920 | BAK1 | -0.02 | 0.12 | 9.54E-01 |
| NM_001006397 | IARS2 | 0.02 | 0.11 | 9.54E-01 |
| NM_001006327 | MED20 | 0.03 | 0.13 | 9.55E-01 |
| NM_001030647 | UBE2H | -0.02 | 0.12 | 9.55E-01 |
| NM_001030815 | ANKRD10 | -0.02 | 0.12 | 9.55E-01 |
| NM_001030883 | ARHGAP25 | -0.03 | 0.16 | 9.55E-01 |
| NM_001012858 | ITGA9 | 0.03 | 0.17 | 9.56E-01 |
| NM_001159370 | LRRC16A | -0.03 | 0.13 | 9.56E-01 |
| NM_204553 | SFRP1 | -0.02 | 0.12 | 9.56E-01 |
| NM_001002242 | CSNK2A1 | -0.02 | 0.13 | 9.56E-01 |
| NM_001007869 | SDC4 | 0.03 | 0.15 | 9.56E-01 |
| NM_001030689 | DOLPP1 | -0.02 | 0.12 | 9.56E-01 |
| NM_204704 | RP11-426L16.10 | -0.02 | 0.10 | 9.56E-01 |
| NM_001012540 | TTLL12 | 0.02 | 0.10 | 9.56E-01 |
| NM_001030731 | CD36 | 0.03 | 0.18 | 9.56E-01 |
| NM_204988 | MERTK | -0.03 | 0.14 | 9.57E-01 |
| NM_001030757 | ST13 | 0.02 | 0.12 | 9.57E-01 |
| NM_001293299 | SLC7A6OS | 0.02 | 0.12 | 9.57E-01 |
| NM_001039329 | PRDX6 | -0.02 | 0.12 | 9.58E-01 |
| NM_204102 | RECQL5 | 0.03 | 0.13 | 9.59E-01 |
| NM_001006152 | PPP2CA | -0.01 | 0.08 | 9.59E-01 |
| NM_001006285 | UQCC | 0.02 | 0.11 | 9.59E-01 |
| NM_001006508 | PMS1 | -0.02 | 0.12 | 9.59E-01 |
| NM_001012569 | AMD1 | 0.02 | 0.10 | 9.59E-01 |
| NM_001030866 | SLMO2 | 0.02 | 0.11 | 9.59E-01 |
| NM_204393 | RECQL | 0.03 | 0.17 | 9.59E-01 |
| NM_001031190 | PSMC3 | 0.02 | 0.13 | 9.59E-01 |
| NM_001030662 | ZCCHC8 | 0.02 | 0.09 | 9.59E-01 |
| NM_001006517 | ORC2 | -0.03 | 0.15 | 9.59E-01 |
| NM_204331 | DPF2 | 0.03 | 0.18 | 9.59E-01 |
| NM_001030594 | TSEN2 | 0.02 | 0.11 | 9.60E-01 |
| NM_001031008 | TEX10 | -0.02 | 0.09 | 9.60E-01 |
| NM_001031485 | WDR3 | -0.02 | 0.12 | 9.60E-01 |
| NM_001137648 | SOCS1 | 0.03 | 0.17 | 9.60E-01 |
| NM_001185051 | PNOC | -0.03 | 0.16 | 9.60E-01 |
| NM_001199701 | PIGH | -0.03 | 0.18 | 9.60E-01 |
| NM_001276384 | TDP2 | 0.02 | 0.11 | 9.60E-01 |
| NM_204324 | PAFAH1B1 | -0.01 | 0.07 | 9.60E-01 |
| NM_001006267 | ARGLU1 | 0.02 | 0.09 | 9.61E-01 |
| NM_001012936 | ASB8 | -0.03 | 0.14 | 9.61E-01 |
| NM_001030618 | CNOT8 | -0.02 | 0.10 | 9.61E-01 |
| NM_001030979 | ANKMY2 | 0.02 | 0.12 | 9.61E-01 |
| NM_001031201 | PRKD1 | 0.03 | 0.18 | 9.61E-01 |
| NM_001031302 | PLEKHB2 | -0.03 | 0.15 | 9.61E-01 |
| NM_001031397 | IKBKB | 0.02 | 0.10 | 9.61E-01 |
| NM_001290201 | CUX1 | 0.02 | 0.11 | 9.61E-01 |
| NM_001006238 | L3MBTL2 | 0.02 | 0.14 | 9.61E-01 |
| NM_001006241 | RPL3 | 0.02 | 0.11 | 9.61E-01 |
| NM_001006431 | PLS3 | -0.02 | 0.14 | 9.61E-01 |
| NM_001007836 | TANGO2 | 0.02 | 0.12 | 9.61E-01 |
| NM_001024829 | TIRAP | -0.02 | 0.14 | 9.61E-01 |
| NM_001030545 | FBXO22 | -0.02 | 0.10 | 9.61E-01 |
| NM_001030569 | HEATR3 | -0.02 | 0.10 | 9.61E-01 |
| NM_001030684 | GTF3C5 | -0.03 | 0.15 | 9.61E-01 |
| NM_001030880 | SZRD1 | 0.02 | 0.10 | 9.61E-01 |
| NM_001031196 | C5H14ORF169 | -0.02 | 0.13 | 9.61E-01 |
| NM_001031331 | CHIR-IG1-5 | -0.03 | 0.17 | 9.61E-01 |
| NM_001031340 | CERK | -0.02 | 0.09 | 9.61E-01 |
| NM_001039261 | FANCD2 | 0.02 | 0.10 | 9.61E-01 |
| NM_001039332 | WBSCR22 | 0.03 | 0.15 | 9.61E-01 |
| NM_001271159 | TRAPPC5 | 0.02 | 0.13 | 9.61E-01 |
| NM_001277369 | BORCS7 | -0.02 | 0.11 | 9.61E-01 |
| NM_001277564 | COG7 | -0.02 | 0.10 | 9.61E-01 |
| NM_001278095 | TWF1 | -0.02 | 0.12 | 9.61E-01 |
| NM_001302175 | SMIM4 | 0.03 | 0.17 | 9.61E-01 |
| NM_204927 | XRCC6 | -0.02 | 0.14 | 9.61E-01 |
| NM_205107 | COL6A1 | -0.02 | 0.13 | 9.61E-01 |
| NR_105493 | MIR6625 | -0.03 | 0.16 | 9.61E-01 |
| NM_205373 | SEMA3D | 0.03 | 0.18 | 9.61E-01 |
| NM_001006298 | ICMT | -0.02 | 0.10 | 9.61E-01 |
| NM_001008446 | ACTR5 | -0.03 | 0.17 | 9.61E-01 |
| NM_001030808 | PIGA | 0.02 | 0.13 | 9.61E-01 |
| NM_001277881 | LOC419390 | -0.03 | 0.16 | 9.61E-01 |
| NM_001006137 | C12H3ORF37 | -0.02 | 0.14 | 9.62E-01 |
| NM_001012794 | TPST2 | 0.02 | 0.15 | 9.62E-01 |
| NM_001031335 | MORN4 | 0.01 | 0.08 | 9.62E-01 |
| NM_204839 | PDLIM4 | 0.02 | 0.15 | 9.62E-01 |
| NM_001006181 | TOP3B | 0.02 | 0.10 | 9.62E-01 |
| NM_001006185 | DDX55 | 0.02 | 0.12 | 9.62E-01 |
| NM_001006279 | TMEM123 | -0.02 | 0.14 | 9.62E-01 |
| NM_001006389 | TGS1 | -0.02 | 0.11 | 9.62E-01 |
| NM_001031127 | VMA21 | 0.02 | 0.10 | 9.62E-01 |
| NM_001079760 | PFN2 | -0.02 | 0.13 | 9.62E-01 |
| NM_001277337 | PBLD | 0.03 | 0.17 | 9.62E-01 |
| NM_204965 | HAND1 | 0.03 | 0.17 | 9.62E-01 |
| NM_205323 | ACTN2 | -0.03 | 0.17 | 9.63E-01 |
| NM_001305165 | RPL15 | 0.02 | 0.13 | 9.63E-01 |
| NM_001006398 | EPRS | -0.02 | 0.12 | 9.63E-01 |
| NM_001012923 | RABGAP1L | -0.02 | 0.12 | 9.63E-01 |
| NM_001039307 | CNPPD1 | -0.02 | 0.12 | 9.63E-01 |
| NM_001257267 | QRICH1 | 0.02 | 0.11 | 9.63E-01 |
| NM_204469 | AIP | -0.02 | 0.11 | 9.63E-01 |
| NM_001006128 | GPI | 0.01 | 0.10 | 9.63E-01 |
| NM_001006202 | NELFB | 0.02 | 0.14 | 9.63E-01 |
| NM_001006275 | SUPT20H | 0.01 | 0.09 | 9.63E-01 |
| NM_001006452 | RPL7L1 | -0.02 | 0.11 | 9.63E-01 |
| NM_001006516 | BZW1 | -0.01 | 0.09 | 9.63E-01 |
| NM_001006551 | MCCC1 | 0.02 | 0.11 | 9.63E-01 |
| NM_001006568 | RP11-529K1.3 | 0.02 | 0.12 | 9.63E-01 |
| NM_001007854 | ADIPOR2 | -0.02 | 0.16 | 9.63E-01 |
| NM_001012694 | TMEM131 | 0.01 | 0.09 | 9.63E-01 |
| NM_001030556 | CASC4 | -0.02 | 0.10 | 9.63E-01 |
| NM_001030578 | KLHDC4 | 0.02 | 0.10 | 9.63E-01 |
| NM_001030733 | ING3 | -0.02 | 0.11 | 9.63E-01 |
| NM_001030858 | TPD52L2 | -0.01 | 0.10 | 9.63E-01 |
| NM_001030878 | TARDBP | 0.02 | 0.12 | 9.63E-01 |
| NM_001030944 | RBM12B | -0.01 | 0.09 | 9.63E-01 |
| NM_001031334 | RRP1B | -0.02 | 0.10 | 9.63E-01 |
| NM_001031460 | RP11-403P17.5 | 0.02 | 0.12 | 9.63E-01 |
| NM_001031500 | DCLRE1B | -0.03 | 0.17 | 9.63E-01 |
| NM_001031575 | RALBP1 | -0.02 | 0.11 | 9.63E-01 |
| NM_001037269 | WNT5B | -0.03 | 0.18 | 9.63E-01 |
| NM_001044637 | SPECC1L | -0.02 | 0.16 | 9.63E-01 |
| NM_001178144 | PID1 | -0.03 | 0.18 | 9.63E-01 |
| NM_001199665 | GANC | -0.02 | 0.12 | 9.63E-01 |
| NM_001257296 | DENND2C | 0.02 | 0.13 | 9.63E-01 |
| NM_001272017 | FAM114A1 | -0.02 | 0.15 | 9.63E-01 |
| NM_001276354 | DBX2 | -0.02 | 0.15 | 9.63E-01 |
| NM_001277587 | COX7A2L | 0.02 | 0.16 | 9.63E-01 |
| NM_001277736 | PRRG1 | 0.02 | 0.14 | 9.63E-01 |
| NM_001278050 | RAB40B | -0.03 | 0.18 | 9.63E-01 |
| NM_001293203 | SORD | 0.02 | 0.12 | 9.63E-01 |
| NM_001305673 | MRRF | 0.02 | 0.12 | 9.63E-01 |
| NM_204201 | CLDN5 | 0.03 | 0.18 | 9.63E-01 |
| NM_204488 | NEBL | 0.03 | 0.17 | 9.63E-01 |
| NM_204514 | SLC35B1 | -0.02 | 0.10 | 9.63E-01 |
| NM_204558 | IRF9 | -0.03 | 0.18 | 9.63E-01 |
| NM_204773 | SFRP2 | -0.02 | 0.14 | 9.63E-01 |
| NM_204941 | CHD1 | 0.02 | 0.14 | 9.63E-01 |
| NM_205124 | PPP2CB | -0.01 | 0.08 | 9.63E-01 |
| NM_205232 | MYBL1 | -0.03 | 0.18 | 9.63E-01 |
| NM_205273 | NFIA | 0.02 | 0.16 | 9.63E-01 |
| NM_205470 | PCK2 | -0.02 | 0.12 | 9.63E-01 |
| NM_001007842 | FBXW2 | 0.01 | 0.08 | 9.64E-01 |
| NM_001012582 | RELL1 | 0.02 | 0.15 | 9.64E-01 |
| NM_001030736 | GXYLT1 | -0.02 | 0.10 | 9.64E-01 |
| NM_001039320 | SIRT6 | 0.02 | 0.13 | 9.64E-01 |
| NM_001199678 | BET1 | -0.02 | 0.11 | 9.64E-01 |
| NM_001006445 | AGA | 0.02 | 0.15 | 9.64E-01 |
| NM_001030930 | MLX | 0.02 | 0.10 | 9.64E-01 |
| NM_001030950 | RAD21 | 0.01 | 0.09 | 9.64E-01 |
| NM_001039274 | MTMR2 | -0.02 | 0.14 | 9.64E-01 |
| NM_001244882 | ARHGAP29 | 0.02 | 0.12 | 9.64E-01 |
| NM_204421 | NR2F2 | -0.02 | 0.10 | 9.64E-01 |
| NM_001012919 | DNPEP | 0.01 | 0.10 | 9.64E-01 |
| NM_204557 | EMB | 0.02 | 0.15 | 9.64E-01 |
| NM_001006362 | HIBADH | 0.01 | 0.09 | 9.65E-01 |
| NM_001012797 | PSMD5 | 0.02 | 0.11 | 9.65E-01 |
| NM_001281486 | SMIM7 | 0.01 | 0.08 | 9.65E-01 |
| NM_001286169 | MYO9B | 0.02 | 0.12 | 9.65E-01 |
| NM_204725 | CASP3 | 0.02 | 0.12 | 9.65E-01 |
| NM_205506 | S100A10 | -0.02 | 0.16 | 9.65E-01 |
| NM_001006482 | TMED10 | -0.02 | 0.12 | 9.65E-01 |
| NM_001012292 | CLP1 | -0.01 | 0.10 | 9.65E-01 |
| NM_001030787 | CD200 | -0.02 | 0.15 | 9.65E-01 |
| NM_001031212 | CDCA4 | 0.02 | 0.11 | 9.65E-01 |
| NM_001199273 | ETV6 | -0.02 | 0.11 | 9.65E-01 |
| NM_001199459 | RDH10 | 0.02 | 0.18 | 9.65E-01 |
| NM_204513 | SLC35A1 | 0.02 | 0.13 | 9.65E-01 |
| NM_204942 | CLIP1 | -0.02 | 0.14 | 9.65E-01 |
| NM_205002 | ID2 | 0.02 | 0.14 | 9.65E-01 |
| NM_001079751 | MBD3 | -0.02 | 0.12 | 9.66E-01 |
| NM_001081505 | MRPL37 | -0.02 | 0.16 | 9.66E-01 |
| NM_001159982 | CPPED1 | -0.02 | 0.17 | 9.66E-01 |
| NM_001199594 | SPTY2D1 | 0.02 | 0.12 | 9.66E-01 |
| NM_001012805 | C19H17ORF85 | 0.01 | 0.11 | 9.67E-01 |
| NM_001030337 | FZD1 | -0.02 | 0.18 | 9.67E-01 |
| NM_001030575 | TLDC1 | -0.01 | 0.10 | 9.67E-01 |
| NM_001030639 | PLK1 | 0.02 | 0.14 | 9.67E-01 |
| NM_001079765 | PPP6C | 0.02 | 0.11 | 9.67E-01 |
| NM_001199540 | MAGI2 | -0.02 | 0.17 | 9.67E-01 |
| NM_001277545 | FTSJ2 | 0.02 | 0.16 | 9.67E-01 |
| NM_001277629 | CALML4 | 0.02 | 0.16 | 9.67E-01 |
| NM_001277734 | DYNLT3 | -0.02 | 0.14 | 9.67E-01 |
| NM_001293241 | ATP6V1B2 | 0.02 | 0.12 | 9.67E-01 |
| NM_204301 | UFD1L | 0.02 | 0.12 | 9.67E-01 |
| NM_204723 | ATP6V0A2 | -0.01 | 0.10 | 9.67E-01 |
| NM_204728 | PPARD | -0.01 | 0.10 | 9.67E-01 |
| NM_001006466 | RRAS2 | -0.01 | 0.11 | 9.67E-01 |
| NM_001030658 | ORAI1 | -0.02 | 0.14 | 9.67E-01 |
| NM_001031380 | PUF60 | 0.01 | 0.08 | 9.68E-01 |
| NM_204787 | VCAN | 0.02 | 0.12 | 9.68E-01 |
| NM_001030798 | C1H21ORF33 | -0.02 | 0.14 | 9.68E-01 |
| NM_001006265 | CDC16 | 0.01 | 0.11 | 9.68E-01 |
| NM_001024832 | DNMT3A | 0.02 | 0.13 | 9.68E-01 |
| NM_001030549 | MAPK6 | 0.02 | 0.14 | 9.68E-01 |
| NM_001199387 | PTPN2 | 0.01 | 0.11 | 9.68E-01 |
| NM_001145042 | C5H11orf96 | 0.02 | 0.17 | 9.69E-01 |
| NM_205116 | MYBPC3 | -0.02 | 0.15 | 9.69E-01 |
| NM_001006523 | ERCC3 | -0.01 | 0.10 | 9.70E-01 |
| NM_001079742 | STARD4 | 0.02 | 0.15 | 9.70E-01 |
| NR_036657 | C14H16orf59 | 0.02 | 0.15 | 9.70E-01 |
| NM_204376 | HZGJ | 0.01 | 0.09 | 9.70E-01 |
| NM_001048007 | COPS8 | -0.01 | 0.12 | 9.70E-01 |
| NM_001006363 | RAB5A | -0.01 | 0.11 | 9.70E-01 |
| NM_001205005 | GNG10 | 0.02 | 0.13 | 9.70E-01 |
| NM_001025304 | BCL2L1 | -0.02 | 0.15 | 9.70E-01 |
| NM_001012872 | PELI1 | 0.02 | 0.12 | 9.71E-01 |
| NM_001030791 | GPR89B | -0.02 | 0.14 | 9.71E-01 |
| NM_001006268 | UBAC2 | -0.02 | 0.13 | 9.72E-01 |
| NM_001006332 | BECN1 | 0.01 | 0.11 | 9.72E-01 |
| NM_001006532 | RALGPS2 | -0.01 | 0.11 | 9.72E-01 |
| NM_001007825 | BLOC1S6 | 0.01 | 0.12 | 9.72E-01 |
| NM_001013395 | ASS1 | -0.01 | 0.12 | 9.72E-01 |
| NM_001030574 | WWOX | -0.02 | 0.17 | 9.72E-01 |
| NM_001031022 | SDR16C5 | -0.01 | 0.11 | 9.72E-01 |
| NM_001031274 | SLC35A3 | -0.02 | 0.14 | 9.72E-01 |
| NM_001031586 | CDK5RAP3 | -0.02 | 0.13 | 9.72E-01 |
| NM_001199557 | TMEM5 | -0.02 | 0.15 | 9.72E-01 |
| NM_001204389 | ARHGAP8 | 0.02 | 0.18 | 9.72E-01 |
| NM_001277141 | HDAC11 | -0.02 | 0.13 | 9.72E-01 |
| NM_205007 | H2AFY | 0.02 | 0.13 | 9.72E-01 |
| NM_205532 | EIF5A2 | -0.01 | 0.10 | 9.73E-01 |
| NM_001012905 | EXOC6 | -0.02 | 0.14 | 9.74E-01 |
| NM_001006497 | DNA2 | 0.01 | 0.09 | 9.74E-01 |
| NM_001127312 | SOCS6 | -0.02 | 0.15 | 9.74E-01 |
| NM_001006434 | APOOL | 0.02 | 0.18 | 9.75E-01 |
| NM_001008440 | LCP1 | -0.01 | 0.11 | 9.75E-01 |
| NM_001012818 | PEX5 | -0.01 | 0.12 | 9.75E-01 |
| NM_205396 | INHBA | 0.02 | 0.18 | 9.75E-01 |
| NM_001039258 | CDH1 | -0.02 | 0.14 | 9.75E-01 |
| NM_001031299 | SLC35G2 | -0.01 | 0.12 | 9.75E-01 |
| NM_001012653 | SURF1 | 0.02 | 0.14 | 9.75E-01 |
| NM_001031262 | CCDC93 | -0.01 | 0.10 | 9.75E-01 |
| NM_205290 | HSPB1 | 0.01 | 0.12 | 9.75E-01 |
| NM_001031209 | CCNK | 0.01 | 0.13 | 9.75E-01 |
| NM_001007892 | CDK6 | -0.01 | 0.12 | 9.77E-01 |
| NM_001079763 | CRYZ | -0.01 | 0.10 | 9.77E-01 |
| NM_001252099 | UGGT2 | -0.01 | 0.11 | 9.77E-01 |
| NM_205252 | HHEX | 0.01 | 0.14 | 9.77E-01 |
| NM_204310 | QKI | -0.02 | 0.18 | 9.77E-01 |
| NM_001006194 | COQ5 | 0.01 | 0.10 | 9.77E-01 |
| NM_001006378 | VPS4B | -0.01 | 0.08 | 9.77E-01 |
| NM_001012557 | C1GALT1 | 0.01 | 0.09 | 9.77E-01 |
| NM_001012926 | Sep-15 | 0.01 | 0.10 | 9.77E-01 |
| NM_001080880 | TMEM170A | 0.01 | 0.10 | 9.77E-01 |
| NM_001198652 | ECHDC3 | 0.02 | 0.15 | 9.77E-01 |
| NM_204888 | FGF12 | 0.02 | 0.15 | 9.77E-01 |
| NM_001001472 | NFKBIA | 0.01 | 0.12 | 9.77E-01 |
| NM_001006360 | TRA2A | 0.01 | 0.08 | 9.77E-01 |
| NM_001006467 | COPB1 | -0.01 | 0.10 | 9.77E-01 |
| NM_001001604 | ST8SIA2 | 0.02 | 0.17 | 9.77E-01 |
| NM_001012405 | EDARADD | -0.02 | 0.15 | 9.77E-01 |
| NM_001025578 | MAD2L2 | -0.01 | 0.12 | 9.77E-01 |
| NM_001199231 | SMIM15 | 0.01 | 0.12 | 9.77E-01 |
| NM_001257279 | TRPC3 | -0.02 | 0.16 | 9.77E-01 |
| NM_001008470 | CD82 | -0.01 | 0.11 | 9.77E-01 |
| NM_001012566 | YIPF3 | 0.01 | 0.14 | 9.77E-01 |
| NM_001030806 | REPS2 | 0.02 | 0.16 | 9.77E-01 |
| NM_001162376 | LYRM1 | -0.01 | 0.12 | 9.77E-01 |
| NM_001278061 | ACAD8 | 0.01 | 0.09 | 9.77E-01 |
| NM_001031531 | ANKRD40 | -0.01 | 0.13 | 9.78E-01 |
| NM_204838 | DUSP4 | 0.02 | 0.17 | 9.78E-01 |
| NM_001030597 | COPG1 | -0.01 | 0.09 | 9.78E-01 |
| NM_001006587 | FANCC | -0.01 | 0.11 | 9.78E-01 |
| NM_001030906 | SRSF10 | 0.01 | 0.08 | 9.78E-01 |
| NM_001167766 | ODC1 | 0.01 | 0.13 | 9.78E-01 |
| NM_205529 | ADSL | -0.01 | 0.11 | 9.79E-01 |
| NM_001199481 | RP11-463D19.2 | 0.01 | 0.11 | 9.80E-01 |
| NM_001006201 | CDK9 | -0.01 | 0.10 | 9.80E-01 |
| NM_001278084 | PDXK | -0.01 | 0.12 | 9.80E-01 |
| NM_001101034 | IGFBP3 | 0.02 | 0.16 | 9.80E-01 |
| NM_001008746 | GNAL | -0.01 | 0.15 | 9.81E-01 |
| NM_001030560 | GFOD2 | -0.01 | 0.14 | 9.81E-01 |
| NM_001030795 | CBR1 | 0.01 | 0.11 | 9.81E-01 |
| NM_001030844 | AAR2 | 0.01 | 0.11 | 9.81E-01 |
| NM_001031129 | CTSO | 0.01 | 0.11 | 9.81E-01 |
| NM_001031442 | DTWD2 | -0.01 | 0.14 | 9.81E-01 |
| NM_001031484 | PTPN6 | 0.01 | 0.16 | 9.81E-01 |
| NM_001034813 | CAMK4 | 0.01 | 0.16 | 9.81E-01 |
| NM_001109939 | CDK10 | -0.01 | 0.12 | 9.81E-01 |
| NM_001110060 | RAB23 | -0.02 | 0.18 | 9.81E-01 |
| NM_001145490 | SLC7A1 | -0.02 | 0.18 | 9.81E-01 |
| NM_001195594 | TCEANC2 | -0.01 | 0.11 | 9.81E-01 |
| NM_001199558 | TBK1 | -0.01 | 0.12 | 9.81E-01 |
| NM_001199748 | UBL7 | 0.01 | 0.12 | 9.81E-01 |
| NM_001277585 | ECI1 | 0.01 | 0.13 | 9.81E-01 |
| NM_001278080 | LOC425137 | -0.01 | 0.14 | 9.81E-01 |
| NM_204411 | GRB2 | -0.01 | 0.14 | 9.81E-01 |
| NM_205348 | COL6A2 | 0.01 | 0.15 | 9.81E-01 |
| NM_001045830 | MED22 | 0.01 | 0.15 | 9.81E-01 |
| NM_001012550 | PSME3 | 0.01 | 0.09 | 9.81E-01 |
| NM_204413 | NFKB2 | -0.01 | 0.12 | 9.81E-01 |
| NM_001006560 | HP1BP3 | 0.01 | 0.09 | 9.81E-01 |
| NM_001006570 | GOLGA7 | 0.01 | 0.13 | 9.81E-01 |
| NM_001012880 | PRIM2 | 0.01 | 0.12 | 9.81E-01 |
| NM_001030698 | STX8 | -0.01 | 0.13 | 9.81E-01 |
| NM_001030761 | PWP1 | -0.01 | 0.10 | 9.81E-01 |
| NM_001031220 | ARID5B | 0.01 | 0.16 | 9.81E-01 |
| NM_001031286 | ORC1 | -0.01 | 0.13 | 9.81E-01 |
| NM_001033641 | TNFRSF11B | 0.01 | 0.17 | 9.81E-01 |
| NM_001079723 | MINA | 0.01 | 0.11 | 9.81E-01 |
| NM_001199599 | RNLS | -0.01 | 0.18 | 9.81E-01 |
| NM_001277139 | MPHOSPH6 | 0.01 | 0.11 | 9.81E-01 |
| NM_001277580 | TMEM53 | 0.01 | 0.15 | 9.81E-01 |
| NM_001278045 | UBXN10 | -0.01 | 0.16 | 9.81E-01 |
| NM_001302165 | SMIM14 | 0.01 | 0.18 | 9.81E-01 |
| NM_001305168 | LOXL2 | 0.01 | 0.10 | 9.81E-01 |
| NM_204258 | SEMA3F | 0.01 | 0.12 | 9.81E-01 |
| NM_204729 | RGN | -0.01 | 0.15 | 9.81E-01 |
| NM_205283 | LAMP1 | 0.01 | 0.07 | 9.81E-01 |
| NM_205289 | ITGA6 | 0.02 | 0.18 | 9.81E-01 |
| NM_001006416 | TAF1B | -0.01 | 0.10 | 9.82E-01 |
| NM_001006222 | GOSR1 | -0.01 | 0.10 | 9.82E-01 |
| NM_001012894 | SMYD5 | 0.01 | 0.09 | 9.82E-01 |
| NM_001030800 | DDX3X | -0.01 | 0.12 | 9.82E-01 |
| NM_001030934 | ANP32B | -0.01 | 0.12 | 9.82E-01 |
| NM_001099358 | CYP21A2 | 0.01 | 0.14 | 9.82E-01 |
| NM_204726 | CASP6 | -0.01 | 0.11 | 9.82E-01 |
| NM_001030836 | RPS3 | -0.01 | 0.12 | 9.83E-01 |
| NM_001030843 | SRSF6 | 0.01 | 0.09 | 9.83E-01 |
| NM_204468 | TERF2IP | -0.01 | 0.13 | 9.83E-01 |
| NM_001097646 | FAM3C | 0.01 | 0.16 | 9.83E-01 |
| NM_205040 | DGCR6 | 0.01 | 0.13 | 9.83E-01 |
| NM_001006399 | INTS7 | -0.01 | 0.13 | 9.83E-01 |
| NM_001008474 | SPATS2L | -0.01 | 0.12 | 9.83E-01 |
| NM_001030659 | TMEM120B | 0.01 | 0.11 | 9.83E-01 |
| NM_001080891 | RABEPK | -0.01 | 0.11 | 9.83E-01 |
| NM_001198537 | ODZ3 | -0.01 | 0.14 | 9.83E-01 |
| NM_001198654 | PEX10 | -0.01 | 0.11 | 9.83E-01 |
| NM_001257290 | TIMMDC1 | 0.01 | 0.14 | 9.83E-01 |
| NM_001277393 | AKR1D1 | 0.01 | 0.14 | 9.83E-01 |
| NM_204817 | ANGPT2 | 0.01 | 0.16 | 9.83E-01 |
| NM_205115 | LIMK2 | -0.01 | 0.16 | 9.83E-01 |
| NM_205136 | CTNNA2 | 0.01 | 0.17 | 9.83E-01 |
| NM_204199 | SMN | 0.01 | 0.10 | 9.83E-01 |
| NM_001006263 | TRAPPC2 | 0.01 | 0.10 | 9.84E-01 |
| NM_001006329 | METTL2A | -0.01 | 0.14 | 9.84E-01 |
| NM_001006343 | TNFAIP8L1 | 0.01 | 0.10 | 9.84E-01 |
| NM_001006507 | PPP2R2D | 0.01 | 0.09 | 9.84E-01 |
| NM_001012564 | EPCAM | -0.01 | 0.16 | 9.84E-01 |
| NM_001012608 | F2RL1 | 0.01 | 0.16 | 9.84E-01 |
| NM_001012793 | GNB1L | 0.01 | 0.12 | 9.84E-01 |
| NM_001030557 | GALK2 | 0.01 | 0.13 | 9.84E-01 |
| NM_001030847 | RP1-309K20.6 | -0.01 | 0.16 | 9.84E-01 |
| NM_001030868 | RAE1 | -0.01 | 0.11 | 9.84E-01 |
| NM_001031016 | B4GALT6 | -0.01 | 0.11 | 9.84E-01 |
| NM_001031070 | ZBTB2 | -0.01 | 0.13 | 9.84E-01 |
| NM_001031325 | ORMDL2 | -0.01 | 0.14 | 9.84E-01 |
| NM_001031573 | ARMC1 | -0.01 | 0.09 | 9.84E-01 |
| NM_001031580 | HPS1 | -0.01 | 0.17 | 9.84E-01 |
| NM_001168007 | CEP63 | 0.01 | 0.12 | 9.84E-01 |
| NM_001202462 | CFLAR | 0.01 | 0.16 | 9.84E-01 |
| NM_001271157 | FARSA | 0.01 | 0.12 | 9.84E-01 |
| NM_001276303 | NACA | -0.01 | 0.15 | 9.84E-01 |
| NM_001278083 | NDUFAF5 | -0.01 | 0.15 | 9.84E-01 |
| NM_001305422 | RMND5A | 0.01 | 0.13 | 9.84E-01 |
| NM_001006401 | RASGRP3 | 0.01 | 0.14 | 9.84E-01 |
| NM_204427 | POFUT1 | 0.01 | 0.09 | 9.84E-01 |
| NM_204638 | FSTL1 | -0.01 | 0.08 | 9.84E-01 |
| NM_001031357 | BORCS5 | -0.01 | 0.13 | 9.84E-01 |
| NM_001031521 | ADSS | -0.01 | 0.13 | 9.84E-01 |
| NM_205085 | ITM2B | 0.01 | 0.10 | 9.84E-01 |
| NM_001030343 | CHST10 | 0.01 | 0.13 | 9.84E-01 |
| NM_001277716 | HMBOX1 | -0.01 | 0.15 | 9.84E-01 |
| NM_001006519 | DYNC1I2 | 0.00 | 0.08 | 9.85E-01 |
| NM_001030634 | DRG2 | -0.01 | 0.13 | 9.85E-01 |
| NM_001115081 | LPAR2 | 0.01 | 0.17 | 9.85E-01 |
| NM_001195557 | CYP2D6 | -0.01 | 0.15 | 9.85E-01 |
| NM_001267075 | SLIT2 | 0.01 | 0.13 | 9.85E-01 |
| NM_204425 | CRIM1 | -0.01 | 0.18 | 9.85E-01 |
| NM_204830 | ELAVL4 | -0.01 | 0.18 | 9.85E-01 |
| NM_001198710 | P4HB | 0.00 | 0.08 | 9.86E-01 |
| NM_001305648 | STXBP1 | -0.01 | 0.17 | 9.86E-01 |
| NM_001031517 | ASB3 | 0.01 | 0.12 | 9.86E-01 |
| NM_001278496 | MTMR14 | -0.01 | 0.13 | 9.86E-01 |
| NM_204735 | MYO6 | 0.01 | 0.12 | 9.86E-01 |
| NM_001006172 | METTL9 | 0.01 | 0.13 | 9.86E-01 |
| NM_001007886 | AP1M1 | -0.01 | 0.11 | 9.86E-01 |
| NM_001030365 | ERBB4 | -0.01 | 0.18 | 9.86E-01 |
| NM_001001766 | LIMS1 | 0.01 | 0.18 | 9.86E-01 |
| NM_001302110 | TIMM8A | -0.01 | 0.12 | 9.86E-01 |
| NM_001030865 | SLC35C2 | 0.01 | 0.14 | 9.86E-01 |
| NM_001031017 | MAPRE2 | -0.01 | 0.15 | 9.86E-01 |
| NM_001097645 | BCAP29 | -0.01 | 0.12 | 9.86E-01 |
| NM_001277104 | UBAP2 | -0.01 | 0.15 | 9.86E-01 |
| NM_001004411 | SERPINI1 | -0.01 | 0.18 | 9.86E-01 |
| NM_001006193 | ACADS | 0.01 | 0.14 | 9.86E-01 |
| NM_001012819 | ZDHHC23 | -0.01 | 0.14 | 9.86E-01 |
| NM_001277407 | LACTB2 | -0.01 | 0.12 | 9.86E-01 |
| NM_205499 | DBN1 | -0.01 | 0.11 | 9.87E-01 |
| NM_001006175 | TRAP1 | 0.01 | 0.14 | 9.87E-01 |
| NM_001012887 | NKRF | -0.01 | 0.12 | 9.87E-01 |
| NM_001030652 | SNAP29 | 0.00 | 0.09 | 9.87E-01 |
| NM_001030751 | SCYL2 | -0.01 | 0.15 | 9.87E-01 |
| NM_001031175 | IGHMBP2 | 0.01 | 0.12 | 9.87E-01 |
| NM_001031354 | NCLN | -0.01 | 0.13 | 9.87E-01 |
| NM_001031467 | EARS2 | -0.01 | 0.14 | 9.87E-01 |
| NM_001031596 | COPS2 | 0.00 | 0.10 | 9.87E-01 |
| NM_001135572 | C1H12ORF73 | -0.01 | 0.16 | 9.87E-01 |
| NM_001277336 | SLIT1 | -0.01 | 0.18 | 9.87E-01 |
| NM_001277419 | UBE2G2 | -0.01 | 0.11 | 9.87E-01 |
| NM_001277843 | SLC25A26 | 0.01 | 0.16 | 9.87E-01 |
| NM_204398 | LHFPL5 | 0.01 | 0.16 | 9.87E-01 |
| NM_204944 | AXIN1 | 0.01 | 0.12 | 9.87E-01 |
| NM_205081 | CTNNB1 | 0.00 | 0.10 | 9.87E-01 |
| NM_205105 | EPHA5 | -0.01 | 0.17 | 9.87E-01 |
| NM_205224 | ACTR2 | 0.00 | 0.10 | 9.87E-01 |
| NM_001012529 | PDPK1 | 0.00 | 0.09 | 9.87E-01 |
| NM_001025354 | POLE3 | -0.01 | 0.15 | 9.87E-01 |
| NM_001030972 | RUNDC3B | 0.01 | 0.17 | 9.87E-01 |
| NM_001031146 | SARAF | 0.00 | 0.11 | 9.87E-01 |
| NM_204278 | TLR2-1 | 0.01 | 0.14 | 9.87E-01 |
| NM_205071 | IGF2BP1 | -0.01 | 0.13 | 9.87E-01 |
| NM_001031041 | TRMT6 | -0.01 | 0.13 | 9.87E-01 |
| NM_001006139 | MCM2 | 0.00 | 0.09 | 9.87E-01 |
| NM_001006226 | PHTF2 | -0.01 | 0.15 | 9.87E-01 |
| NM_205123 | PPP1R12A | -0.01 | 0.15 | 9.87E-01 |
| NM_001277555 | NARFL | 0.00 | 0.10 | 9.88E-01 |
| NM_205477 | MYH9 | 0.01 | 0.14 | 9.89E-01 |
| NM_001006342 | C28H19ORF10 | 0.00 | 0.09 | 9.89E-01 |
| NM_001007594 | WNT6 | 0.01 | 0.18 | 9.89E-01 |
| NM_205278 | MYL9 | 0.00 | 0.12 | 9.89E-01 |
| NM_001044662 | PRKAB2 | 0.00 | 0.13 | 9.89E-01 |
| NM_001012562 | ANKH | -0.01 | 0.14 | 9.90E-01 |
| NM_001171164 | PARP4 | 0.01 | 0.15 | 9.90E-01 |
| NM_001198651 | CD99 | 0.01 | 0.16 | 9.90E-01 |
| NM_001199561 | IVD | 0.00 | 0.14 | 9.90E-01 |
| NM_001293173 | C4orf48 | -0.01 | 0.17 | 9.90E-01 |
| NM_204209 | OGN | 0.01 | 0.15 | 9.90E-01 |
| NM_001031236 | SHOC2 | -0.01 | 0.17 | 9.91E-01 |
| NM_001031131 | SLC10A7 | 0.00 | 0.13 | 9.91E-01 |
| NM_001105664 | CAV1 | 0.01 | 0.18 | 9.91E-01 |
| NM_001031079 | USP45 | 0.00 | 0.15 | 9.92E-01 |
| NM_204430 | FUT10 | 0.00 | 0.14 | 9.92E-01 |
| NM_001005831 | PRMT7 | 0.00 | 0.11 | 9.92E-01 |
| NM_001031609 | SEPP1 | 0.00 | 0.15 | 9.92E-01 |
| NM_001079746 | FAM105A | 0.00 | 0.11 | 9.92E-01 |
| NM_001277774 | EFCAB14 | 0.00 | 0.11 | 9.92E-01 |
| NM_204706 | BOK | 0.00 | 0.10 | 9.92E-01 |
| NM_001031005 | RPRD1A | -0.01 | 0.17 | 9.92E-01 |
| NM_001277568 | MED8 | 0.00 | 0.12 | 9.92E-01 |
| NM_001031239 | FAM45A | 0.00 | 0.14 | 9.92E-01 |
| NM_001199624 | BTD | 0.00 | 0.14 | 9.92E-01 |
| NM_001198540 | GPATCH3 | 0.00 | 0.14 | 9.93E-01 |
| NM_001276306 | PTGES3 | 0.00 | 0.11 | 9.94E-01 |
| NM_001277490 | ELP6 | 0.00 | 0.14 | 9.94E-01 |
| NM_204872 | WSB1 | 0.00 | 0.09 | 9.94E-01 |
| NM_205213 | MST1 | 0.00 | 0.12 | 9.94E-01 |
| NM_001012581 | UGDH | 0.00 | 0.13 | 9.94E-01 |
| NM_204291 | P2RX4 | 0.00 | 0.15 | 9.94E-01 |
| NM_001031269 | SEC16B | 0.00 | 0.14 | 9.94E-01 |
| NM_204663 | SLC16A3 | 0.00 | 0.18 | 9.95E-01 |
| NM_001271528 | AAGAB | 0.00 | 0.14 | 9.95E-01 |
| NM_001199533 | NRBF2 | 0.00 | 0.11 | 9.96E-01 |
| NM_001006141 | SUCLG2 | 0.00 | 0.12 | 9.96E-01 |
| NM_001006261 | AC025048.1 | 0.00 | 0.09 | 9.96E-01 |
| NM_001008475 | PPIL3 | 0.00 | 0.14 | 9.96E-01 |
| NM_001012864 | CLPTM1L | 0.00 | 0.11 | 9.96E-01 |
| NM_001012884 | WDR44 | 0.00 | 0.11 | 9.96E-01 |
| NM_001030338 | IRX1 | 0.00 | 0.18 | 9.96E-01 |
| NM_001030564 | VAC14 | 0.00 | 0.12 | 9.96E-01 |
| NM_001030613 | UBLCP1 | 0.00 | 0.08 | 9.96E-01 |
| NM_001030726 | TNFAIP1 | 0.00 | 0.10 | 9.96E-01 |
| NM_001030970 | CUL2 | 0.00 | 0.09 | 9.96E-01 |
| NM_001031277 | SAMD13 | 0.00 | 0.15 | 9.96E-01 |
| NM_001031418 | SGTB | 0.00 | 0.10 | 9.96E-01 |
| NM_001039279 | PHF20L1 | 0.00 | 0.09 | 9.96E-01 |
| NM_001079488 | DUT | 0.00 | 0.15 | 9.96E-01 |
| NM_001199472 | PMPCB | 0.00 | 0.11 | 9.96E-01 |
| NM_001256151 | ATRIP | 0.00 | 0.11 | 9.96E-01 |
| NM_001276313 | CENPT | 0.00 | 0.09 | 9.96E-01 |
| NM_001277589 | CAMKMT | 0.00 | 0.17 | 9.96E-01 |
| NM_001277715 | IMP4 | 0.00 | 0.13 | 9.96E-01 |
| NM_001277820 | KLHL2 | 0.00 | 0.10 | 9.96E-01 |
| NM_001293185 | PTPN9 | 0.00 | 0.15 | 9.96E-01 |
| NM_204367 | RHBG | 0.00 | 0.13 | 9.96E-01 |
| NM_204775 | LY6E | 0.00 | 0.08 | 9.96E-01 |
| NM_205241 | ST6GAL1 | 0.00 | 0.14 | 9.96E-01 |
| NM_205488 | MSX1 | 0.00 | 0.16 | 9.96E-01 |
| NM_001012589 | EXOC5 | 0.00 | 0.10 | 9.96E-01 |
| NM_001030835 | PAAF1 | 0.00 | 0.12 | 9.96E-01 |
| NM_001302174 | SPTSSA | 0.00 | 0.13 | 9.96E-01 |
| NM_001006369 | KIAA1143 | 0.00 | 0.12 | 9.97E-01 |
| NM_001199426 | KIF18A | 0.00 | 0.11 | 9.97E-01 |
| NM_001199446 | DACH1 | 0.00 | 0.13 | 9.97E-01 |
| NM_001277418 | ITM2C | 0.00 | 0.16 | 9.97E-01 |
| NM_204280 | ERG | 0.00 | 0.18 | 9.97E-01 |
| NM_204378 | FANCG | 0.00 | 0.15 | 9.97E-01 |
| NM_001031394 | ALG10 | 0.00 | 0.13 | 9.97E-01 |
| NM_001007851 | RASSF3 | 0.00 | 0.12 | 9.97E-01 |
| NM_204975 | KIF4A | 0.00 | 0.10 | 9.97E-01 |
| NM_001006468 | NUCB2 | 0.00 | 0.15 | 9.98E-01 |
| NM_001277557 | TMEM56 | 0.00 | 0.17 | 9.98E-01 |
| NM_001006522 | PECR | 0.00 | 0.11 | 9.98E-01 |
| NM_001006545 | TBCCD1 | 0.00 | 0.13 | 9.98E-01 |
| NM_001277539 | PSMG3 | 0.00 | 0.15 | 9.98E-01 |
| NM_204225 | DCXR | 0.00 | 0.11 | 9.98E-01 |
| NM_204995 | ALDH1A2 | 0.00 | 0.16 | 9.98E-01 |
| NM_001006418 | FAM49A | 0.00 | 0.10 | 1.00E+00 |
| NM_001201455 | SMAD1 | 0.00 | 0.15 | 1.00E+00 |
| NM_001277712 | ATOX1 | 0.00 | 0.17 | 1.00E+00 |
| NM_001006245 | WASH1 | 0.00 | 0.12 | 1.00E+00 |
| NM_001044645 | CD320 | 0.00 | 0.14 | 1.00E+00 |
| NM_001271527 | DUSP28 | 0.00 | 0.13 | 1.00E+00 |
| NM_001276364 | SIRT5 | 0.00 | 0.14 | 1.00E+00 |
| NM_001277569 | ERO1L | 0.00 | 0.16 | 1.00E+00 |
